# C=O methylenation mediated by organo-alkali metal reagents: metal identity and ligand effects†

**DOI:** 10.1039/d5sc02313k

**Published:** 2025-05-19

**Authors:** Xiao Yang, Nathan Davison, Matthew E. Lowe, Paul G. Waddell, Roly J. Armstrong, Claire L. McMullin, Matthew N. Hopkinson, Erli Lu

**Affiliations:** a School of Chemistry, University of Birmingham Edgbaston Birmingham B15 2TT UK n.davison@bham.ac.uk e.lu@bham.ac.uk; b Chemistry – School of Natural and Environmental Sciences, Newcastle University Newcastle Upon Tyne NE1 7RU UK roly.armstrong@newcastle.ac.uk; c Department of Chemistry, University of Bath Claverton Down Bath BA2 7AY UK cm2025@bath.ac.uk

## Abstract

C=O methylenation mediated by α-silyl organo-alkali metal reagents, namely Peterson methylenation, is a textbook organic reaction that has been widely employed in synthetic chemistry for over 50 years. The process is performed over two steps, by isolating the β-silyl alcohol intermediate generated *via* nucleophilic addition and then subjecting it to elimination. The choices of alkali metal and external Lewis base ligand play a critical role in the elimination step, but the reasons remain poorly understood. In this work, we have systematically investigated the metal identity and ligand effects in C=O methylenation reactions mediated by MCH_2_SiMe_3_ (M = Li; Na; K). We observed pronounced alkali metal cation and ligand effects on the methylenation performance, with K^+^ and tetrahydrofuran (THF) being optimal. Based upon these learnings, a straightforward new methylenation method has been designed involving carbonyl addition with LiCH_2_SiMe_3_, followed by *in situ* addition of KO*t*Bu in THF, facilitating facile transmetallation-enabled elimination. This strategy enables the methylenation to be achieved in one pot, whilst circumventing the use of KCH_2_SiMe_3_. Excellent yields have been achieved for a range of ketones (including enolizable examples) and aldehydes. The method uses commercial solvents and reagents, and can be performed without any requirement for stringent drying or deoxygenation.

## Abstract 2

A systematic study of alkali metal identity and ligand effects allows us to develop a Li–K transmetallation-enabled facile C=O methylenation method.

## Graphical abstract

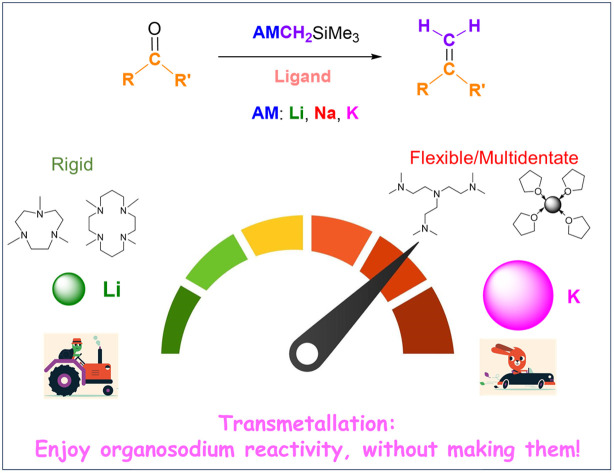

## 1 Introduction

**Fig. 1:**
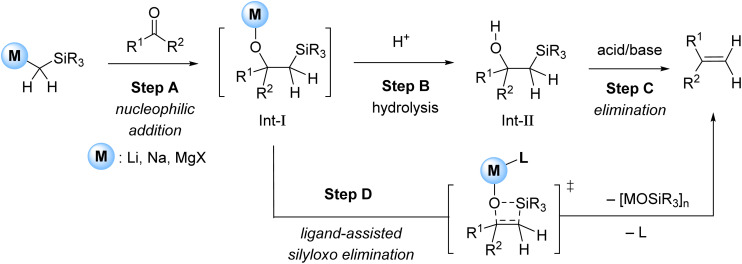
Traditional Peterson methylenation (steps A to C) and one-pot ligand-catalysed methylenation.^13^

Methylenation of a C=O bond (C=O to C=CH_2_) is a widely used functional group interconversion in organic synthesis. Over the decades, many reagents have been developed to effect this transformation, including textbook named reactions such as Wittig,^1,2^ Tebbe^3^ (including zirconium modification^4^), Julia^5^/Julia-Kocieński^6,7^ and Peterson.^8–10^ However, most of these methods suffer from the drawback of requiring toxic and hazardous reagents. In this context, Peterson methylenation is highly attractive because it employs relatively abundant and less toxic group-1 and -2 organometallic reagents, such as α-silyl organolithium,^8^ organosodium^8,9^ or Grignard reagents.^11,12^ However, traditional Peterson methylenation is a stepwise process, requiring hydrolysis of the intermediate β-silyl alkoxide (Int-I) generated after carbonyl addition followed by treatment with acid or base to afford the final olefin product (Fig. 1, steps B–C).^8–12^ The inconvenience of this two-step sequence, combined with the requirement for hazardous reagents to effect elimination in step C (*e.g.* strong mineral acids or pyrophoric potassium hydride), has meant that the Peterson method has generally been overlooked in favour of other methylenation methods.

In 2023, we reported a one-pot Peterson methylenation mediated by an organosodium reagent, NaCH_2_SiMe_3_, which in the presence a tetradentate amine ligand, tris-2-(dimethylamino)ethyl amine (Me_6_Tren), enabled direct carbonyl methylenation without any requirement to isolate the β-silyl alcohol intermediate.^13^ The key to success in this case is the ability of the *in situ* generated β-silyl sodium alkoxide to undergo spontaneous *syn*-elimination (Fig. 1, step-D). It was established that both the identity of the metal cation and ligand play crucial roles in this elimination. Specifically: (1) NaCH_2_SiMe_3_ enables efficient reaction but not LiCH_2_SiMe_3_; (2) the ligand (Me_6_Tren in this case) is also essential, but could be employed catalytically (5 mol%) whilst still maintaining efficient reactivity.

Despite the promise of this chemistry, it is not practical from a synthetic organic chemistry perspective because organosodiums, or organo-heavy-alkali-metal reagents (AM-R, AM = K, Rb, Cs; R = alkyl, aryl), are not commercially available and are challenging to prepare and handle, requiring specialized glovebox techniques. For these reasons, despite recent advances,^13–16^ organo-heavy-alkali-metal reagents have found limited applications in organic synthesis. We rationalized that if this problem could be overcome, it might be possible to develop a new method for Peterson methylenation that operates in one-pot, with commercially available reagents, without any challenging glovebox manipulations. Herein, we describe the successful realization of this goal, which was facilitated by performing in-depth studies of the coordination chemistry and reactivity of β-silyl alkoxide intermediates, including the identity of the metal and ligand effects. Based on these learnings, we have been able to develop a new one-pot method for Peterson-type olefination employing a transmetallation-enabled approach, involving carbonyl addition with commercially available LiCH_2_SiMe_3_ followed by addition of KO*t*Bu in THF to facilitate *in situ* transmetallation and elimination. This operationally simple method has been employed to methylenate a wide range of C=O substrates, including aldehydes and ketones in high yields at 5 mmol scale.

## 2 Results and discussion

### 2.1 Metal identity effect

**Fig. 2:**
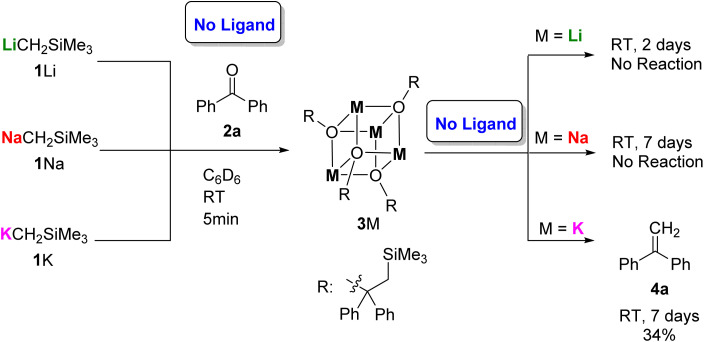
Reactions between 1M and 2a without ligand (M = Li, Na, K).

We commenced our study by evaluating reactions between 1M (M = Li, Na, K) and one equivalent of benzophenone (2a) in *d*_6_-benzene without any ligand (Fig. 2). In all three cases, rapid nucleophilic addition was observed, leading to formation of alkoxide tetramers 3M, which were all characterized by single-crystal X-ray diffraction studies (3Li & 3Na in our previous work,^13^3K herein^17^). The three alkoxides 3M feature similar tetrameric cubic structure, but exhibited different reactivity, with 3Li and 3Na indefinitely stable at room temperature in *d*_6_-benzene, but 3K slowly converting into the methylenation product 4a over the course of one week (Fig. 2).

**Fig. 3:**
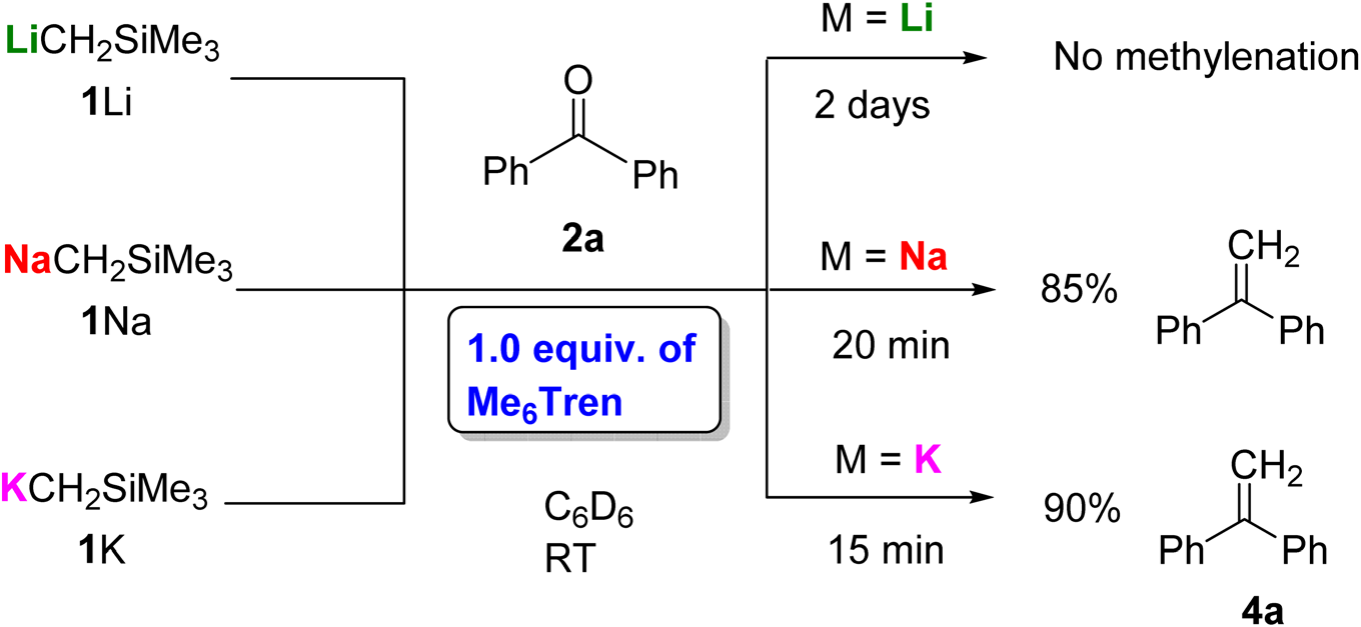
Benzophenone methylenation promoted by one equivalent of Me_6_Tren and 1M.

We found that adding an exogenous ligand to these β-silyl alkoxide complexes had a significant impact on the rate of this elimination process, and upon addition of one equivalent of the flexible polydentate ligand Me_6_Tren, 1K and 1Na both underwent rapid elimination to form methylenation product 4a (Fig. 3). In contrast, 1Li underwent no conversion to 4a, even with extended reaction times.

Based upon these results, we concluded that the first nucleophilic addition step is fast for 1Li/Na/K, but the subsequent elimination step is slow and depends on the identity of the alkali metal cation, following the reactivity trend K > Na ≫ Li. With the presence of Lewis basic ligand Me_6_Tren, the elimination is substantially accelerated for K and Na, while Li remains unreactive. In light of this, we set out to perform a systematic study to further explore how the choice of ligand influences this key rate-determining elimination step.

### 2.2 Ligand effects: donor atom, flexibility and denticity

We chose the selection of eight ligands shown in Table 1 that would allow the impact of the following key structural features to be evaluated: (1) ligand donor atom identity, *e.g.*, N-donor *vs.* O-donor; (2) ligand denticity, *e.g.*, tridentate, bidentate, *etc.*; (3) ligand flexibility, *i.e.*, flexible *vs.* rigid.

**Table 1:** Ligands studied in this work, described by three factors: (1) N- *vs.* O-donors (table *y*-axis); (2) denticity (table *x*-axis); (3) rigidity (colour code)

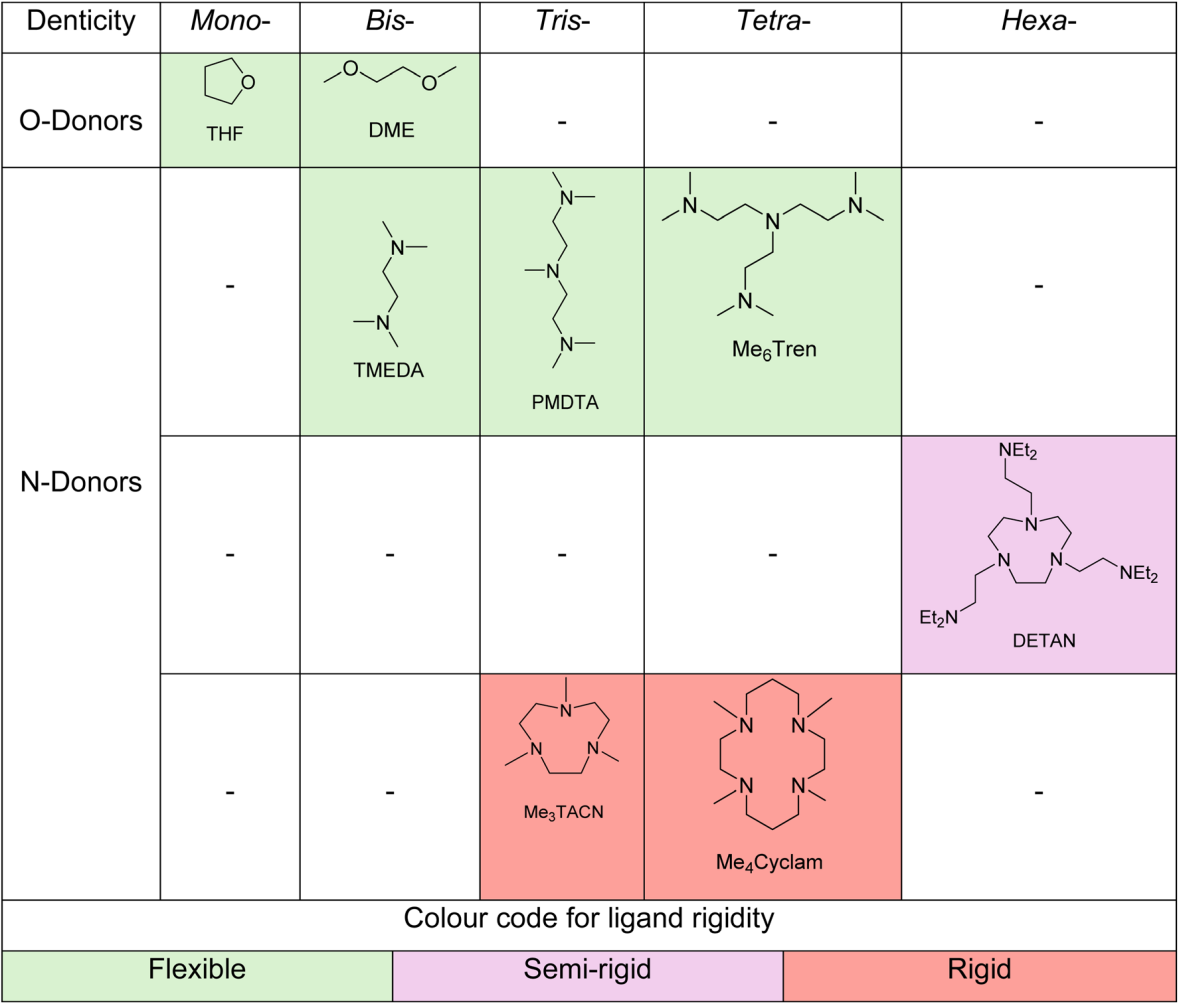

All eight of these ligands (1 equivalent for all, except THF, which was used in large excess quantity as solvent) were explored in model methylenation reactions of benzophenone (4a) with LiCH_2_SiMe_3_, NaCH_2_SiMe_3_ and KCH_2_SiMe_3_. The reactions were performed at 0.04 mmol scale in *d*_6_-benzene (or *d*_8_-THF for entry 2, Table 2) allowing *in situ*^1^H NMR analysis to assess the ratio of ligated β-silyl alkoxide intermediates and methylenated product 4 at various time intervals. The results are shown below in Table 2 along with the data from control experiments performed in the absence of ligand (Table 2 entry 1). Detailed comparison and discussion are presented below, grouped according to the impacts of donor atom identity, denticity and flexibility.

**Table 2:** Systematic studies to explore ligand effects on benzophenone methylenation. The colour codes qualitatively indicate the reaction outcome

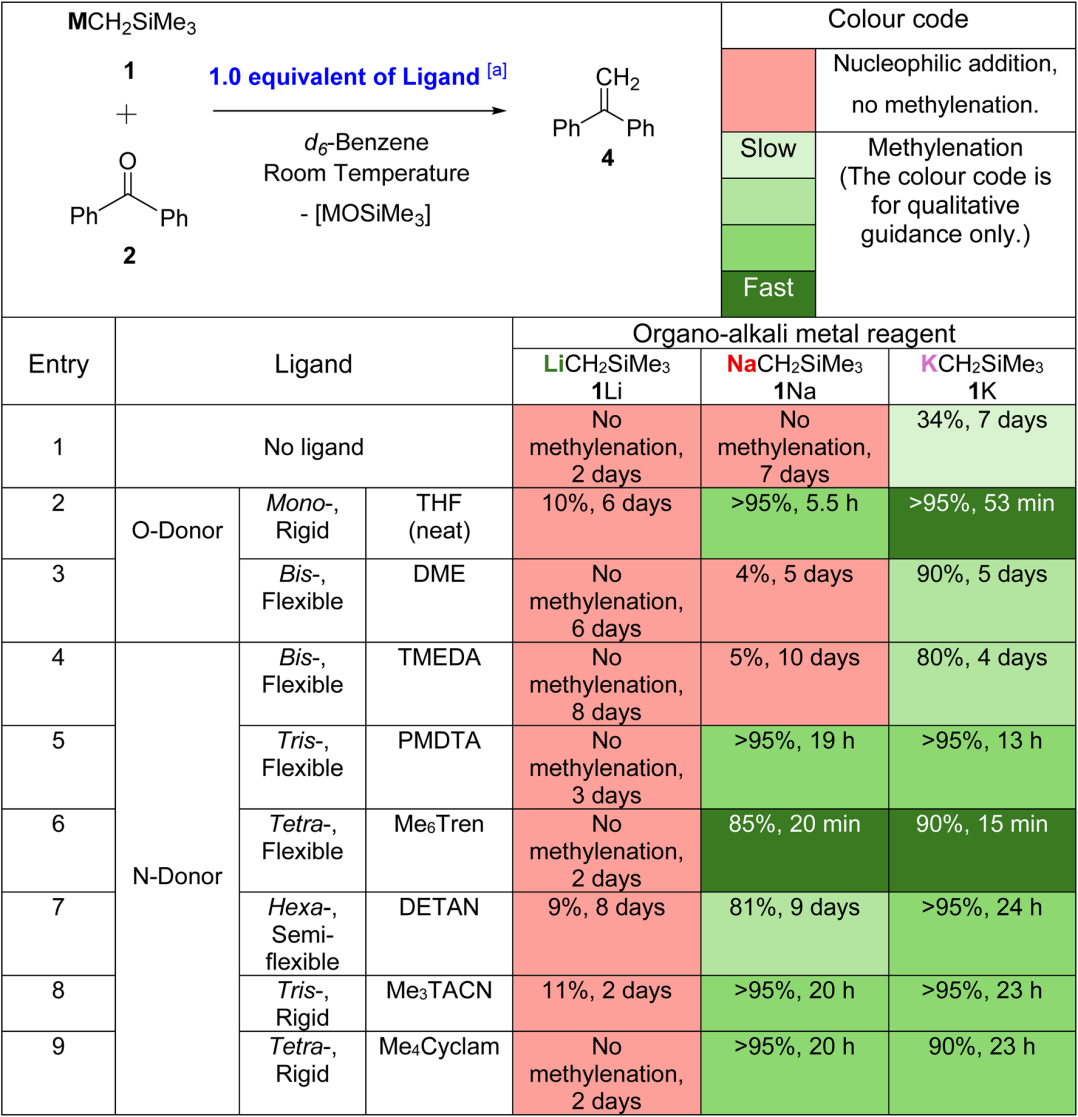

a Solvent quantity of THF (neat) was employed in these reactions.

#### 2.2.1 N- *vs.* O-donors

Comparing the like-for-like cases of DME (bidentate O-donor; Table 2 entry 3) and TMEDA (bidentate N-donor; Table 2 entry 4), which are both bidentate flexible ligands, indicates that little difference, if any, exists between the O-donor and N-donor ligands irrespective of the identity of the alkali metal cation. Note that the reaction with THF is not suitable for such a comparison, because: (1) it was used as a bulk solvent (*ergo* large excess quantity); (2) our studies do not include a suitable monodentate N-donor ligand for comparison.

#### 2.2.2 Ligand denticity

The N-donor ligands TMEDA (Table 2 entry 4), PMDTA (Table 2 entry 5) and Me_6_Tren (Table 2 entry 6) provide an ideal platform to probe the effect of ligand denticity upon reactivity. As all three are flexible, acyclic ligands, any trend observed can be separated from competing effects arising from rigidity (discussed in the following Section 2.2.3). Comparing methylenations mediated by 1Na, it can be clearly seen that the more coordination sites on the ligand, the faster the rate of elimination – with bidentate TMEDA, elimination is incomplete after 10 days (Table 2 entry 4), whereas with tridentate PMDTA, >95% conversion to 4a was achieved after 19 hours (Table 2 entry 5). Tetradentate Me_6_Tren further accelerates the rate of elimination, giving 85% conversion to 4a within 20 minutes (Table 2 entry 6). The same trend can be observed in the 1K mediated methylenations, where the reaction rates follow the trend TMEDA < PMDTA < Me_6_Tren.

On first inspection, this trend appears reversed for O-donor ligands THF and DME (Table 2, entries 2–3), with THF (monodentate) promoting more rapid elimination than DME (bidentate). However, it should be noted that THF is used as bulk solvent and is present in large excess, whereas a stoichiometric quantity of DME was used. We hypothesize that, in a similar manner to a flexible multidentate ligand, the alkali metal cation is coordinated by several THF molecules, which act collectively in a similar manner to a sizeable flexible multidentate ligand.

#### 2.2.3 Rigid *vs.* flexible

In contrast with the clear preference for multidentate ligands, the ligand rigidity effect was more ambiguous. For example, tridentate ligands Me_3_TACN (rigid) (Table 2 entry 8) and PMDTA (flexible) (Table 2 entry 5) led to similar reactivity with 1Na, but PMDTA was slightly more efficient at promoting elimination in reactions performed with 1K (PMDTA, >95% 13 h *vs.* Me_3_TACN, >95% 23 h). This trend was much more obvious for the tetradentate ligands Me_6_Tren (Table 2 entry 6) and Me_4_Cyclam (Table 2 entry 9): with the flexible Me_6_Tren able to promote elimination of both the sodium and potassium alkoxides within 20 minutes, and Me_4_Cyclam resulting in much more sluggish elimination, especially in the case of the potassium alkoxide, which did not fully convert after 23 hours. We conclude that, in general, flexible ligands are superior to rigid ones. An interesting case is the hexadentate semi-flexible ligand DETAN^18^ (Table 2 entry 7), which seems less effective than both the corresponding flexible and rigid analogues. We speculate that the poor performance of DETAN could be due to either the presence of too many coordination sites (six), which “over-saturate” the metal center, or competition between the macrocyclic N-donors and the flexible amine sidearms which deteriorate the overall performance.

### 2.3 Computational studies to understand the metal identity effect in Me_6_Tren system

In our previous report,^13^ we calculated the pathways for the reactions between the ligated MCH_2_SiMe_3_ monomer [M(Me_6_Tren)(CH_2_SiMe_3_)] (M = Li, Na), and benzophenone, and postulated that a series of lower kinetic barriers for the Na-system *cf.* the Li-system are accountable for the observed Na-only methylenation. In the previous study, we considered the Me_6_Tren ligand coordinated to Li/Na metal centers as a monomer, as well as species that were unsupported by a ligand, highlighting the need to factor ligand-metal coordination into the mechanistic model. Regardless of the presence of Me_6_Tren ligand, the polymeric aggregates [LiCH_2_SiMe_3_]_6_ (1Li) or [NaCH_2_SiMe_3_]_∞_ (1Na) conduct fast nucleophilic addition towards the C=O bond of benzophenone (2a), producing tetrameric alkoxide complexes [MOC(CH_2_SiMe_3_)Ph_2_]_4_ (3Li/Na), which have been isolated and unequivocally characterized (*vide supra*). To further elucidate the mechanism behind the metal-dependent methylenation, our computational investigations now focused on the potentially key role the tetrameric alkoxide complexes 3M play in the observed reactivity, with Me_6_Tren as the chosen model ligand (L) to keep in line with already reported data.^13^ A full mechanistic pathway for methylenation at a hypothetical organopotassium monomer [(κ^4^-Me_6_Tren)K(CH_2_SiMe_3_)] (Fig. S61†) and at a hypothetical ligand-free KCH_2_SiMe_3_ (1K) (Fig. S62) are provided in the ESI† for comparison to the existing Li and Na studies, alongside the computational methodology details.

Previously, a large free energy stabilization for the tetrameric compounds 3M, Δ*G* = −123.0 and −142.5 kcal mol^−1^ for Na and Li respectively was reported.^13^ Similarly, for the K system, the tetrameric cluster 3K is calculated to be Δ*G* = −129.1 kcal mol^−1^, which is lower than the monomer [(κ^4^-Me_6_Tren)K(CH_2_SiMe_3_)], and has all four N donor atoms of the Me_6_Tren ligand coordinated at the K center (a κ^3^-N conformer was also explored and is 7.4 kcal mol^−1^ higher in free energy). A more appropriate ranking of these 3M species relative to 1M is to consider only a quarter of the cluster, essentially the coordinated product of nucleophilic addition “M(OC(CH_2_SiMe_3_)Ph_2_)”, modifying the free energies to be −30.7 and −35.6 kcal mol^−1^ for ¼ 3Na and ¼ 3Li respectively, and for the computed potassium tetramer, ¼ 3K, −32.3 kcal mol^−1^.

**Fig. 4:**
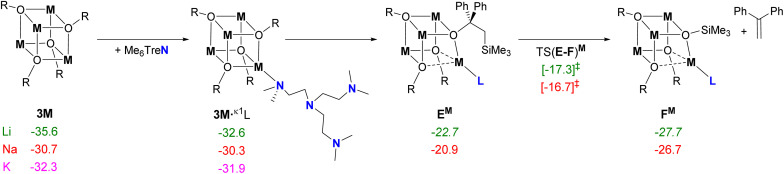
DFT computed free energies for addition of Me_6_TreN (L) to 3M and subsequent silyl abstraction, in kcal mol^−1^ (BP86-D3^BJ^(C_6_H_6_)/BS2//BP86/BS1). Free energy values are relative to 1M and benzophenone using ¼ 3M. R = OC(CH_2_SiMe_3_)Ph_2_, L = Me_6_Tren.

These alkoxide tetramers would likely not exist in isolation in solution, and it is reasonable to consider association of a ligand (or two) to 3M. A range of conformers of 3M·(Me_6_Tren)_*x*_ were computationally explored (see ESI† for full details), with a ligand binding to one of the four M corners of the alkoxide cube seeing a minimal increase in free energy (see Fig. 4, 3M· κ^1^L). After the initial κ^1^ coordination of one Me_6_Tren ligand (L) to 3M, the R group –C(CH_2_SiMe_3_)Ph_2_ needs to rotate about the O–C–C–Si bond so that the SiMe_3_ group is *syn* to the original O–C carbonyl bond (in the plane; O–C–C–Si ∼ 0°) and β-silyl abstraction is possible. This pre-silyl-abstraction intermediate, E^M^, was located for both Li and Na systems, with the rotation of the SiMe_3_ group raising the free energy by *ca.* 10 kcal mol^−1^, as decomposition of the M_4_O_4_ cuboid structure begins with elongation of the other two O–M bonds. Abstraction through TS(E-F)^M^ gives free energy barriers of 18.3 and 14.0 kcal mol^−1^ respectively from 3Li and 3Na, to form the Ph_2_C = CH_2_ product, 4a, and broken tetramer, F^M^, which is higher in free energy than 3M.

Silyl abstraction could not be isolated for 3K·κ^1^L with a Me_6_Tren ligand coordinated. However, a ligand-free silyl abstraction at a “naked” 3K′′ was attempted and located, with a notably lower free energy barrier of 8.5 kcal mol^−1^ (see ESI, Fig. S62,† again, the product species sees the decomposition of the tetramer core with a Δ*G* = −36.8 kcal mol^−1^). Similar ligand-free silyl abstraction at 3Li and 3Na were investigated, but could not be found. This notable change in mechanism for the different group-1 tetramers matches experimental data, which saw methylenation for the potassium system in the absence of the Me_6_Tren ligand (∼50% in two weeks, Fig. 3), yet not for the sodium or lithium systems. This lower barrier for the non-ligated 3K system could also explain the outstanding methylenation performance of potassium in the ligand study overall.

Often, attempts to computationally coordinate a ligand to the tetrameric cluster would facilitate disassembly into lower aggregates, which was more pronounced for 3Li and 3K. Only 3Na could support a κ^2^ conformer (3Na·κ^2^L; Δ*G* = −26.8 kcal mol^−1^, see ESI†), with many attempts to locate equivalent intermediates failing repeatedly for 3Li and 3K. Further inspection of the alkoxide clusters suggests that steric considerations are the main argument for this behavior – albeit for different reasons. With the lithium system, a second arm of the Me_6_Tren ligand is unable to coordinate to an Li atom due to the smaller metal radius. Whilst it could be assumed that a larger group-1 atom in 3K has more available space to support κ^2^ binding, this capacity has instead established a strong π-interaction with one of the phenyl groups from R of the original benzophenone substrate, blocking the potential second ligand coordination site at the K atom.

Full disaggregation is calculated to be highly endergonic (4 × C; Δ*G* = +40.0 kcal mol^−1^, relative to 3Na and 4 Me_6_TreN ligands), suggesting that a level of aggregation may remain during the silyl abstraction. Ultimately, the rate determining step (regardless of metal identity) is found to be silyl elimination, where the Li system features the higher kinetic barrier.

### 2.4 Transmetallation strategy

**Fig. 5:**
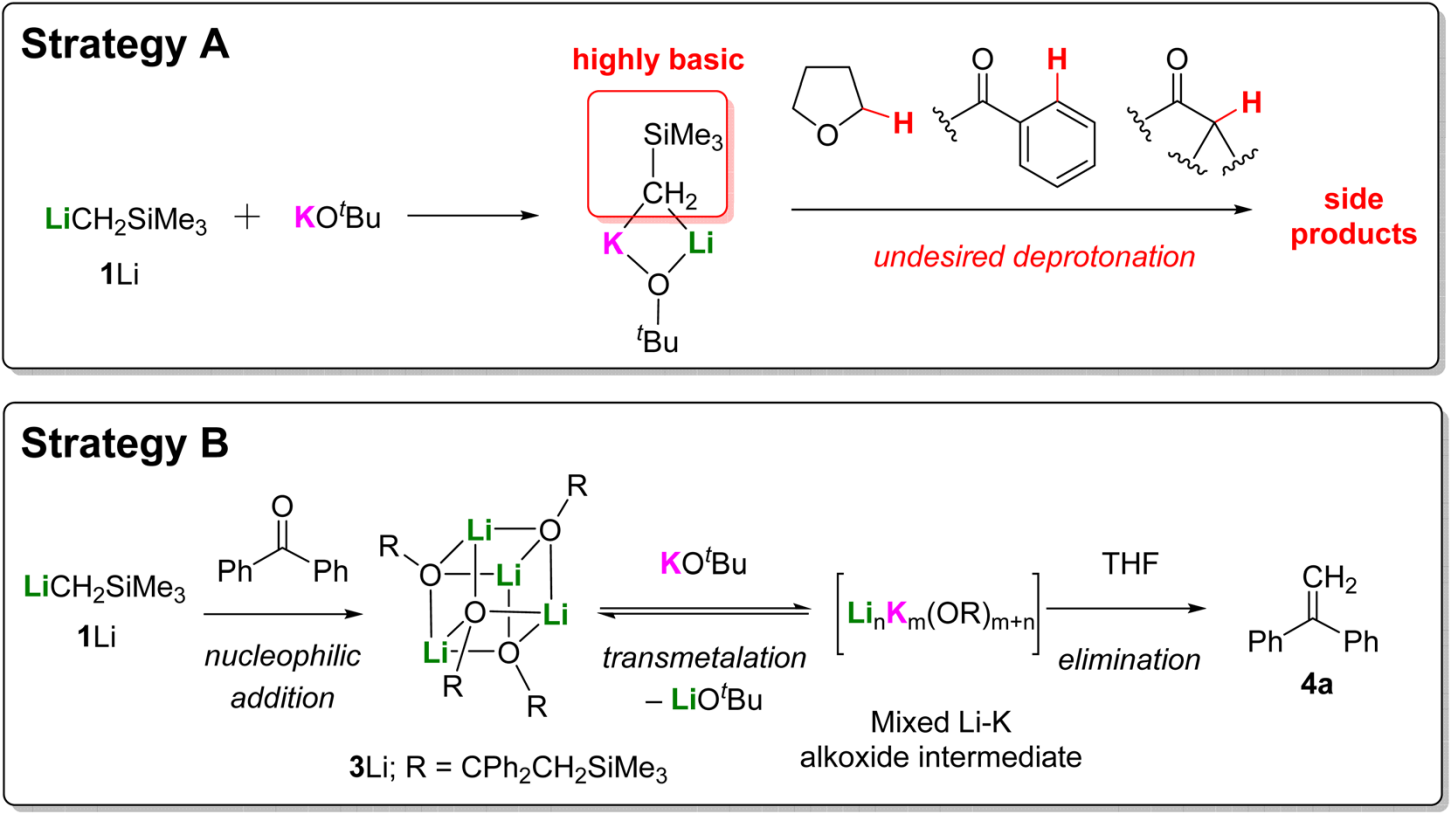
Schematic representations of the two transmetallation strategies: (A) transmetallation before nucleophilic addition; (B) transmetallation after nucleophilic addition.

Our survey of ligand and metal studies had revealed that rapid and efficient one-pot Peterson methylenation of benzophenone can be achieved with KCH_2_SiMe_3_ (1K) in THF, but this was still far from a practical solution. 1K is not commercially available and is even more challenging to synthesize and handle than 1Na, requiring preparation in a glovebox and storage in a −35 °C freezer. With this in mind, we speculated that it might be possible to avoid the isolation of the key organopotassium reagent 1K by forming it *in situ via* transmetallation of the corresponding commercially available organolithium 1Li with a potassium salt such as KO*t*Bu (Fig. 5, strategy A). However, thoughtful consideration soon rules out this approach, as the formation of heterobimetallic species (*c.f.* LIC-KOR superbases pioneered by Lochmann and Schlosser) would likely lead to many potential undesired side reactions. The presence of THF could further enhance the Brønsted basicity of such species,^19^ which could lead to undesired deprotonation of THF or of substrates containing aromatic ketones with C(sp^2^)–H bonds,^20^ or enolizable ketones/aldehydes with α-protons.^21^ To avoid such side reactions, we wondered if an alternative approach might be possible (Fig. 5, strategy B), in which the carbonyl addition step is performed with commercially available LiCH_2_SiMe_3_ (1Li) to generate the elimination-inert lithium alkoxide 3Li. Subsequent addition of KO*t*Bu could then effect transmetallation to access the key elimination-enabled potassium alkoxide species, which would spontaneously deliver the olefin product 4. This approach represents the best of both worlds, allowing the use of the commercially available LiCH_2_SiMe_3_ (1Li), yet capturing the propensity for facile elimination of the THF-ligated potassium alkoxide identified in our studies above.

**Fig. 6:**
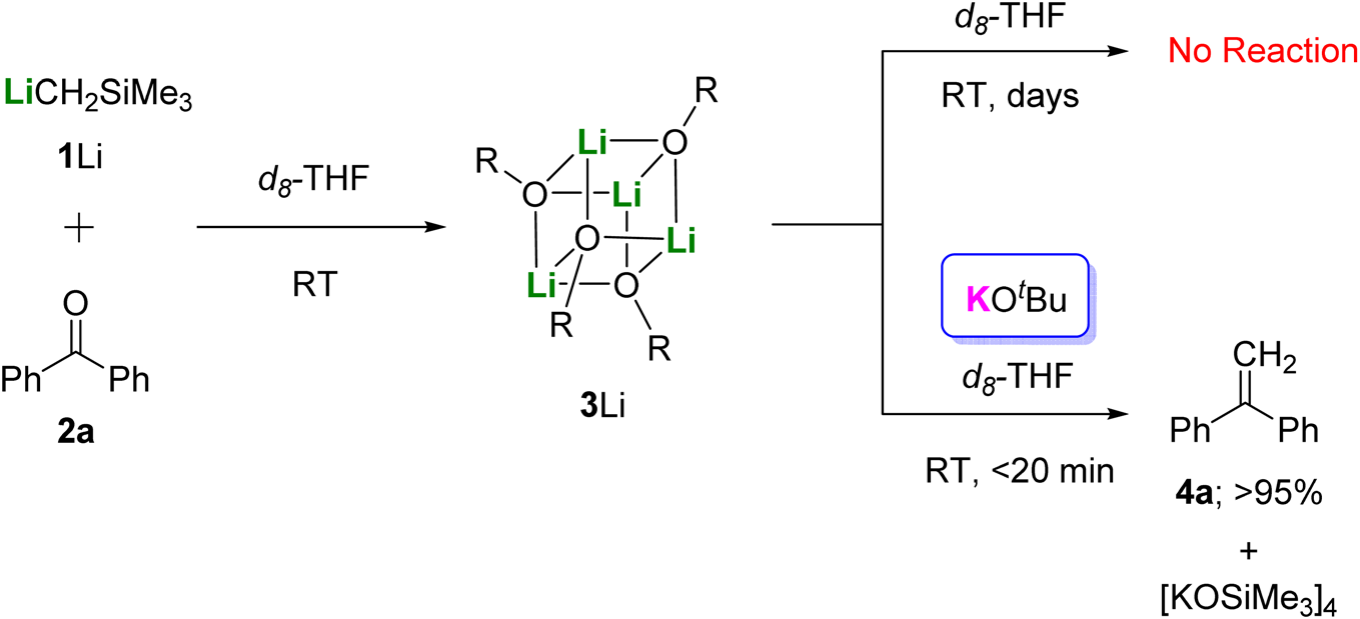
The transmetallation strategy to promote methylenation using 1Li and KO*t*Bu.

To our gratification, we found that *in situ* formation of 3Li by reaction of a commercially available solution of LiCH_2_SiMe_3_ with 2a, followed by addition of one equivalent of KO*t*Bu in *d*_8_-THF led to full conversion into 4a at room temperature within 20 minutes (Fig. 6). The by-product of the elimination step [KOSiMe_3_]_4_, was confirmed by monitoring the ^1^H NMR spectrum and comparing with that of the SCXRD-characterized authenticated sample. Readers should note that the single crystals of [KOSiMe_3_]_4_ were isolated in a separated reaction of 1K, 2a, and Me_6_Tren, rather than from this transmetallation reaction. In contrast, without KO*t*Bu, 3Li did not promote the conversion from 2a to 4a even after several days (Fig. 6).

#### 2.4.1 Generality of transmetallation-enabled peterson methylenation

**Fig. 7:**
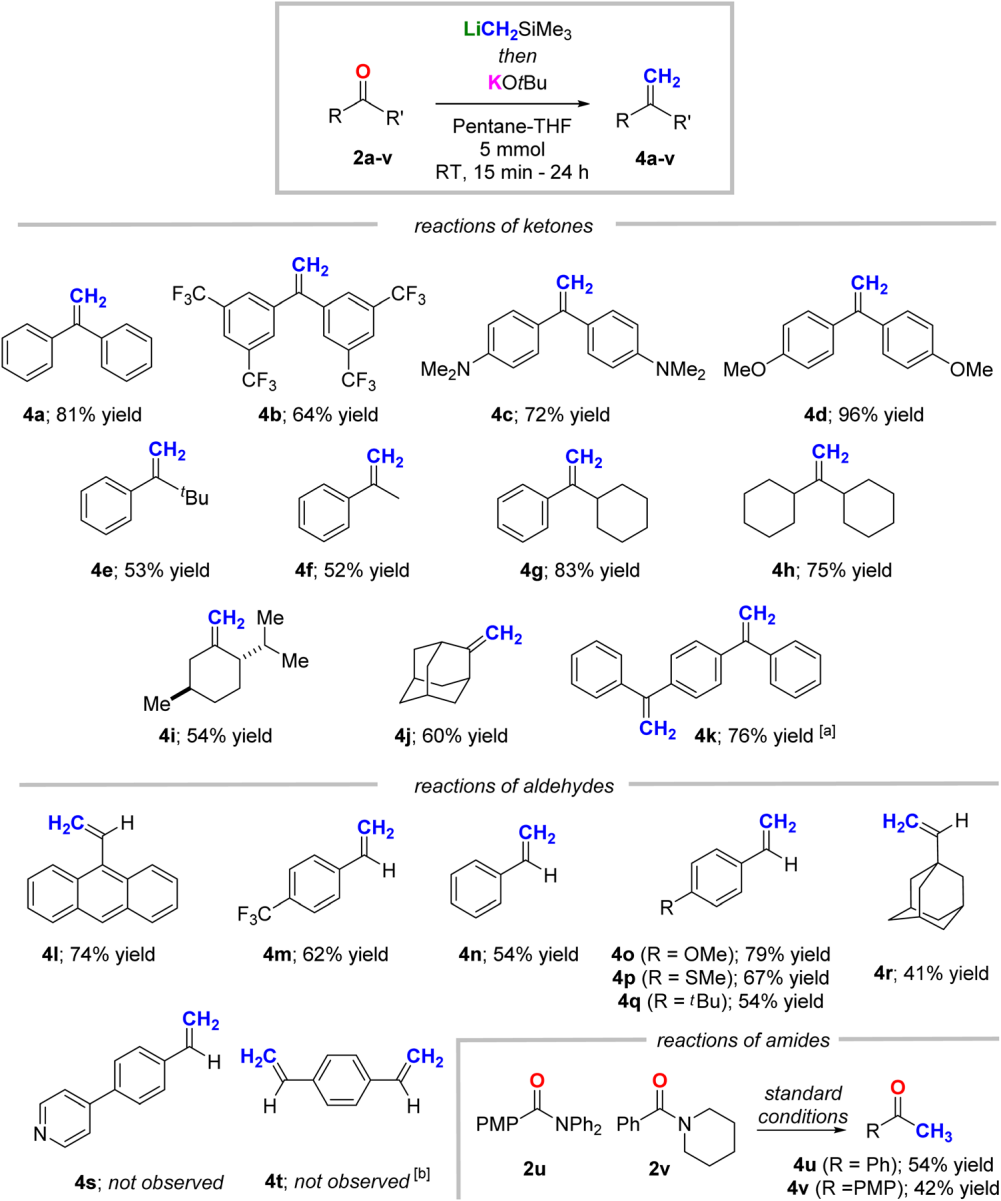
Transmetallation methylenation of substrates 2a–t. Reaction conditions: 5 mmol of substrates 2a–t; reaction temperature and time: (i) nucleophilic addition: 0 °C to room temperature 1–2 hours; (ii) elimination: −78 °C to room temperature, 1.5 hours to 3 days. Yields are based upon isolated product obtained by column chromatography. ^*a*^Reaction carried out with LiCH_2_SiMe_3_ (3.0 equiv.) and KO*t*Bu (2.0 equiv.); attempts to mono-methylenate failed. ^*b*^Polymerization was observed. PMP = *para*-methoxyphenyl.

With an efficient one-pot method for transmetallation-enabled Peterson methylenation established, we set out to explore its generality (Fig. 7). We were delighted to find that a variety of electron deficient and electron rich benzophenone analogues were well-tolerated, delivering the corresponding products (4a–d) in good to excellent yields. Sterically congested ketones were also effective substrates, with a reaction of *tert*-butyl phenyl ketone affording methylenated product 4e in 53% yield. Of a particular note, enolizable ketones were also well tolerated, undergoing olefination with minimal evidence of deprotonation to deliver products 4f–h in yields of 52–83%. Likewise, a reaction of menthone afforded 4i in 54% yield without any erosion of relative stereochemistry. The ability of this new method to methylenate enolizable substrates is a major benefit over our previous method employing 1Na-Me_6_Tren, which was incompatible with ketones containing acidic α-protons.^13^ We hypothesize that the suppression of the undesired enolization process is due to the weaker Brønsted basicity and the stronger nucleophilicity of LiCH_2_SiMe_3_ (1Li) *cf.* NaCH_2_SiMe_3_ (1Na).^15^ The reaction was also successfully applied to prepare bridged bicyclic olefin 4j and double methylenation of 1,4-dibenzoylbenzene delivered the corresponding product 4k in 76% yield.

We were also pleased to discover that aromatic aldehydes could also be smoothy methylenated under our optimized conditions to access a variety of styrene analogues, including examples with sterically encumbered (4l), electron poor (4m), electron neutral (4n) and electron rich (4o–q) aromatic rings. Aromatic aldehydes bearing thioethers and aliphatic substituents were also well tolerated, with the corresponding styrenes 4p and 4q isolated in yields of 67% and 54%, respectively. Beyond aromatic aldehydes, adamantyl alkene (4r) was obtained in 41% yield from 1-adamantanecarboxaldehyde (2r). Not all the examples we tested were successful – for example, pyridine containing product 4s was not formed under the reaction conditions. This could be a result of nucleophilic dearomatization of the pyridine group mediated by 1Li: organo-alkali metal reagents are well-known to undergo such pyridine nucleophilic dearomatization.^22^ Likewise, an attempted double methylenation reaction of terephthalaldehyde (2t) to access 4t was unsuccessful, with only insoluble materials produced which we attribute to formation of polymeric products.

We also explored reactions with two amides, *N*-benzoylpiperidine (2u) and 4-methoxy-*N*,*N*-diphenylbenzamide (2v). Instead of the methylenation products (vinyl amines), the corresponding methyl ketones 2u and 2v were isolated as the major products in 54% and 42% yields, respectively. These ketone products could theoretically arise *via* hydrolysis of the desired enamine products upon aqueous workup, but given that these intermediates could not be observed by *in situ* NMR experiments, direct addition–elimination followed by desilylation or silicon-assisted amine elimination seems more probable.^23^ We chose not to test esters, as in our previous study, an ester was observed to undergo a silyl ether elimination to form an enolate intermediate following the initial nucleophilic addition with organosodiums.^13^

#### 2.4.2 Preliminary mechanistic studies of the transmetallation step

To gain a better understanding of the key transmetallation step, we conducted preliminary mechanistic studies. An NMR-scale reaction between isolated 3Li and KO*t*Bu in non-coordinative solvent *d*_6_-benzene generated a complex ^1^H NMR spectrum, which was different from both 3Li and 3K (Fig. S30).† This observation suggests that there may be more than one heterometallic Li–K species co-existing in a number of equilibria.

To further explore this hypothesis computationally, we calculated the relative free energies of 3Li, 3K and three heterometallic tetrameric species, namely Li_3_K, Li_2_K_2_ and LiK_3_, representing different levels of transmetallation. The results are summarized in Table 3. Although 3Li is more stable than 3K, the free energy difference between the two pure clusters is only 3.3 kcal mol^−1^, while free energy differences between the heterometallic species are even smaller. This data suggests a series of equilibrium processes are likely.

**Table 3:** Calculated relative free energies in kcal mol^−1^ for 3Li, Li_3_K, Li_2_K_2_, LiK_3_ and 3K (BP86-D3^BJ^(C_6_H_6_)/BS2//BP86/BS1)

	Relative free energy [in kcal mol^−1^]
3Li	−35.6
Li_3_K	−34.6
Li_2_K_2_	−33.3
LiK_3_	−33.2
3K	−32.3

**Fig. 8:**
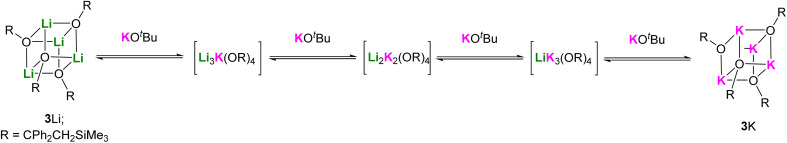
Equilibria of Li–K transmetallation.

Hence, we hypothesize that the transmetallated intermediate obtained from 3Li and KO*t*Bu likely adopts a series of equilibria, as schematically presented in Fig. 8. Readers should be reminded that, though our calculations modelled tetrameric structures (which are based on the SCXRD structures of 3Li and 3K), the presence of different aggregate sizes is still possible. Encouraged by the success of the transmetallation strategy in Section 2.4.1, a comprehensive mechanistic study is underway in our groups.

## 3 Conclusion

In this work we describe the development of a highly efficient new transmetallation-enabled Peterson-type methylenation reaction, which was enabled by an in-depth study of the fundamental coordination chemistry of the key β-silyl alkoxide intermediates involved in the process. This work clearly demonstrates that, both alkali metal identity and ligand have profound influences over reaction patterns: understanding these factors has allowed us to achieve precise control over the key elimination step in Peterson methylenation. Future efforts in our groups are being directed towards using alkali metal identity and ligands as tuning handles to conduct other highly desirable yet challenging organic reactions, such as transition-metal-free, chemo- and enantioselective non-polar C–E bond activation and functionalization (E: H, C, N, O).

## Author contributions

X. Y., N. D. and M. E. L. conducted the syntheses and experimental characterisations. C. L. M. designed and conducted the computational studies. P. G. W. collected and refined the single-crystal X-ray diffraction data. E. L. and N. D. conceptualised the project with input from R. J. A. and M. N. H. E. L., R. J. A. and C. L. M. wrote the manuscript with input from all the authors.

## Conflicts of interest

The authors declare no competing financial interest.

## Supplementary Material

SC-016-D5SC02313K-s001

SC-016-D5SC02313K-s002

## Data Availability

CCDC 2374573 ([KOSiMe_3_]_4_) and 2374574 (3 K) contain the supplementary crystallographic data for the corresponding complexes, respectively. This data can be obtained free of charge *via*https://www.ccdc.cam.ac.uk/data_request/cif, or by emailing data_request@ccdc.cam.ac.uk, or by contacting The Cambridge Crystallographic Data Centre, 12 Union Road, Cambridge CB2 1EZ, UK; fax: +44 1223 336033.
