# Supplementary material for: C[double bond, length as m-dash]O methylenation mediated by organo-alkali metal reagents: metal identity and ligand effects
Source: Chem Sci. 2025 May 19;16(24):11151–60. doi: 10.1039/d5sc02313k (PMC12101075; doi:10.1039/d5sc02313k)
Supplement: SC-016-D5SC02313K-s001 [file SC-016-D5SC02313K-s001.pdf]

Supplementary Information (SI) for Chemical Science.
This journal is © The Royal Society of Chemistry 2025

Supplementary Materials for

C=O Methylenation mediated by organo-alkali metal reagents: metal identity and ligand effects

Xiao Yang,[1] Nathan Davison,*[1] Matthew Lowe,[2] Paul G. Waddell,[2] Roly J. Armstrong,*[2] Claire L. McMullin*[3] Matthew Hopkinson[2] and Erli Lu* [1]

Affiliation:

1 School of Chemistry, University of Birmingham, Edgbaston, Birmingham, B15 2TT, U. K.

2 Chemistry–School of Natural and Environmental Sciences, Newcastle University, Newcastle upon Tyne NE1 7RU, U. K.

3 Department of Chemistry, University of Bath, Claverton Down, Bath BA2 7AY, U.K.

*Corresponding authors: n.davison@bham.ac.uk (N. D.), roly.armstrong@newcastle.ac.uk (R. J. A.), cm2025@bath.ac.uk (C. L. M), e.lu@bham.ac.uk (E. L.),

This PDF file includes:

Materials and Methods
Supplementary Text
Figures S1 to S64
Tables S1 to S5

Section 1. Experimental methods and data---S2
Section 2. Computational details and data---S54
Section 3. References--S122

Materials and Methods

Section 1. Experimental methods and data

1.1 General procedures

All manipulations were carried out in a Vigor™ glovebox equipped with a −35 ˚C freezer and a cold well, under an atmosphere of dry argon. The solvents were dried with sodium press, sodium-potassium alloy and distilled under reduced pressure, and kept in the glovebox. Chemicals were purchased from Merck, Fluorochem, Tokyo Chemical Industry, Fisher scientific, Apollo or Alfa Aesar, Acros Organics, and dried under dynamic vacuum for several hours, or over activated 4Å molecular sieves, prior to use. All glassware, including pipettes, vials and ampoules, was silylated by treating with trimethylsilyl chloride (Me_3SiCl), rinsing with water, and dried in a 150 °C oven for 12 hours prior to use.

1Li was prepared as previously described.[1] $NaCH_2SiMe_3$ and KCH_2SiMe_3 were prepared by a modified procedure of that described in the literature.[2,3,4]

Solution-state ^{1}H spectra were recorded on Bruker 300 Avance III spectrometer, Bruker 400 Avance III spectrometer and Bruker 400 Avance NEO spectrometer operating at 300 MHz, 400 MHz and 400 MHz, respectively. ^{19}F NMR spectra were recorded on a Bruker Avance NEO spectrometer operating at 377 MHz.

Chemical shifts are quoted in ppm and are relative to $SiMe_4$ (^{1}H).

1.2 Methylenation of Benzophenone

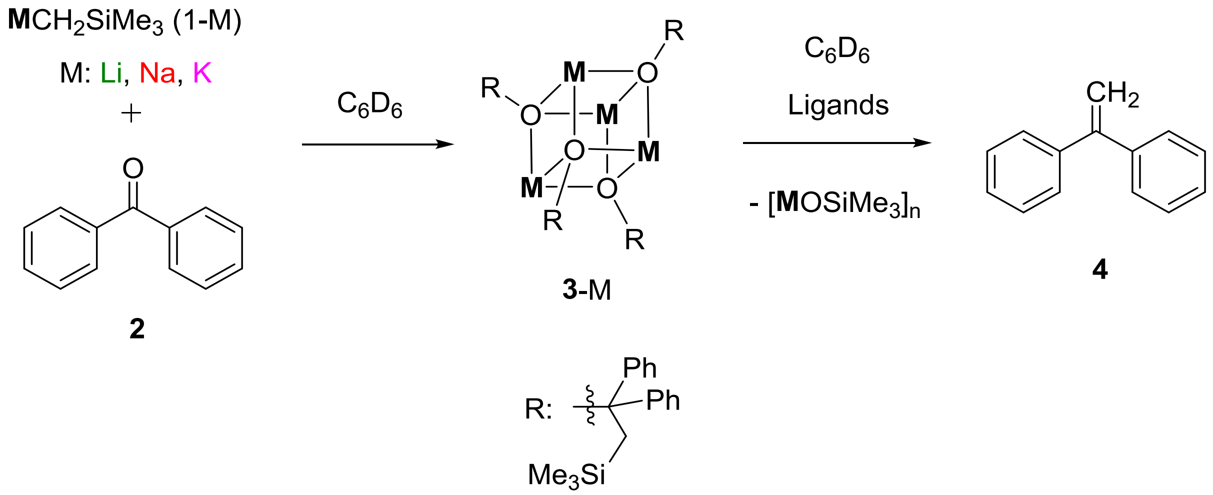

General procedure for MCH_2SiMe_3 + benzophenone in C_6D_6 (no ligand) (Main text Table 2 Entry 1)

Adamantane (internal standard, 0.0054g, 0.04 mmol) was dissolved in C_6D_6 (0.5 mL). The solution was added to benzophenone (0.0073 g, 0.04 mmol). The resulting solution was added to MCH_2SiMe_3 (M = Li, Na, K) (0.04 mmol) in a one-portion manner at room temperature. The resulting solution was transferred to a J. Young NMR tube. The reactions were monitored at room temperature by NMR spectroscopy for conversion to 1,1-diphenylethylene. Conversion was calculated by comparing the internal standard adamantane signals with the =$\mathbf{C}\boldsymbol{H_2}$ signal from 1,1-diphenylethylene. The results are summarised in Main text Table 2.

Single-crystals of 3K for SCXRD study

Benzophenone (0.0911 g, 0.5 mmol) was dissolved in benzene (0.5 mL). The resulting colourless solution was added to a suspension of KCH_2SiMe_3 (0.0632 g, 0.5 mmol) in benzene (1 mL). The resulting blue solution was left at room temperature for 5 minutes before the volatiles were removed *in vacuo*. A pale blue solid resulted. The solid was dissolved in a mixture of *n*-hexane (2.5 mL) and methylcyclohexane (0.75 mL). The solution was filtered and placed in a -35 °C freezer. Colourless crystals suitable for SCXRD resulted after approximately 4 weeks. ^{1}H NMR of the single crystals matched the NMR scale reaction.

^{1}H NMR of **3K** (300 MHz, C_6D_6, 25 °C): δ (ppm) 7.39-7.30 (m, 4H, Ar*H*), 7.28-7.18 (m, 4H, Ar*H*), 7.12-7.03 (m, 2H, Ar*H*), 1.37 (s, 2H C*H*$_2$Si), -0.18 (s, 9H, Si*Me*$_3$).

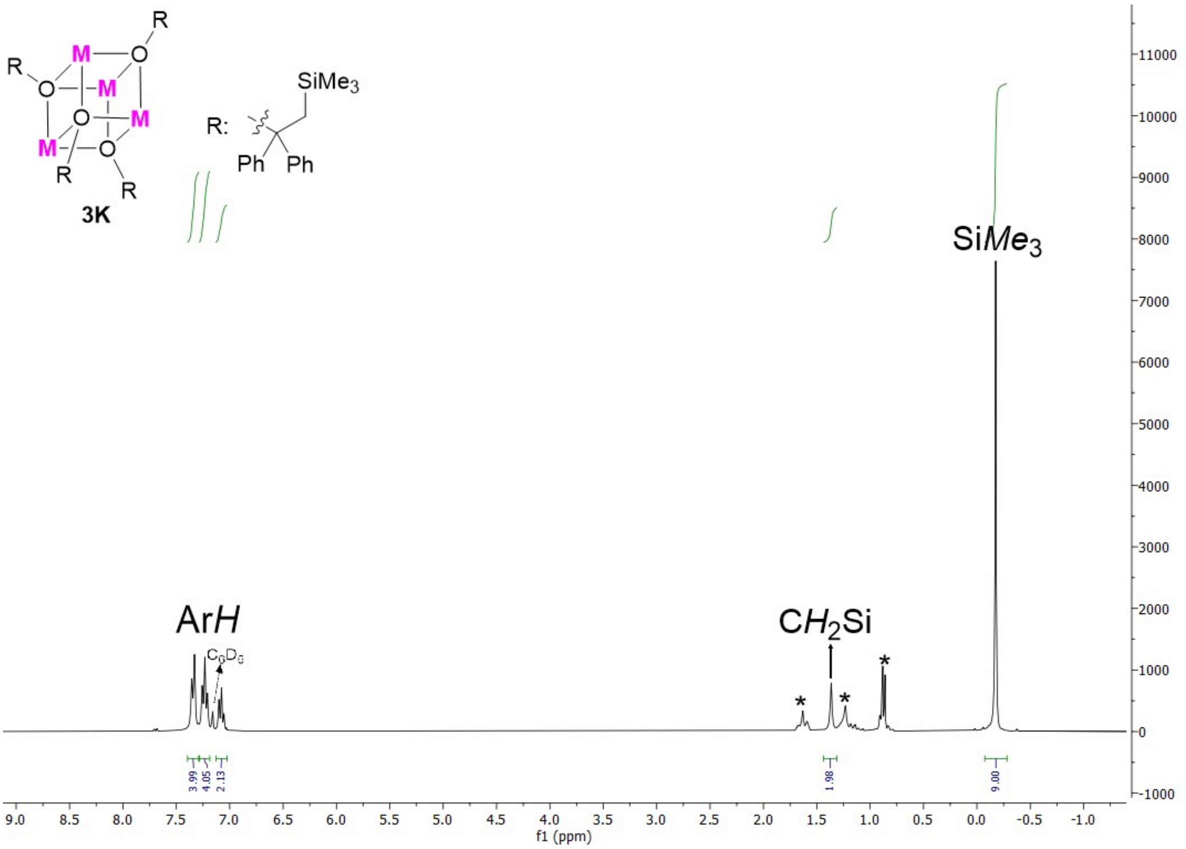

Figure S1: ^{1}H NMR (d_6-benzene, 25 ˚C, 300 MHz) of crystals of **3K** (* = residual crystallisation solvent signals n-hexane/methylcyclohexane).

General procedure for MCH_2SiMe_3 + benzophenone in neat d_8-THF (Main text Table 2 Entry 2)
Adamantane (internal standard, 0.0054g, 0.04 mmol) was dissolved in d_8-THF (0.5 mL). The solution was added to benzophenone (0.0073 g, 0.04 mmol). The resulting solution was added to MCH_2SiMe_3 (M = Li, Na, K) (0.04 mmol) in a one-portion manner at room temperature. The resulting solution was transferred to a J. Young NMR tube. The reactions were monitored by NMR spectroscopy at room temperature for conversion to 1,1-diphenylethylene. Conversion was calculated by comparing the internal standard adamantane signals with the =C***H*₂** signal from 1,1-diphenylethylene. The results are summarised in Main text Table 2.

General procedure for MCH_2SiMe_3 + benzophenone + ligand in C_6D_6 (Main text Table 2 Entries 3–9)

Benzophenone (0.0073 g, 0.04 mmol) was dissolved in C_6D_6 (0.5 mL). The solution was added to MCH_2SiMe_3 (M = Li, Na, K) (0.04 mmol) in a one-portion manner at room temperature. The resulting solution was added to one equivalent the ligand (L = DME, TMEDA, PMDTA, Me_6Tren, DETAN, Me_3TACN, Me_4Cyclam) (0.04 mmol) at room temperature. The resulting solution was transferred to a J. Young NMR tube. The reactions were monitored by NMR spectroscopy for conversion to 1,1-diphenylethylene. Conversion was calculated using by comparing the integration of the ligand signals and the =**C$\boldsymbol{H_2}$** signal from 1,1-diphenylethylene. The results are summarised in Main text Table 2.

Single-crystals of $[KOSiMe_3]_4$ for SCXRD study

Colourless single crystals of the potassium silyloxo cluster $[KOSiMe_3]_4$, resulted after 1 week from the NMR scale reaction of KCH_2SiMe_3 + benzophenone + Me_6Tren in C_6D_6.

^{1}H NMR of 1,1-diphenylethylene (**4a**) (400 MHz, d_6-benzene, 25 °C): δ (ppm) 7.33 – 7.27 (m, 4H, Ar*H*), 7.12 – 7.07 (m, 6H, Ar*H*), 5.36 (s, 2H, =CH_2).

NMR data are consistent with the literature.[5]

^{1}H NMR of 1,1-diphenylethylene (**4a**) (400 MHz, d_8-THF, 25 °C): δ (ppm) 7.30 (s, 10H, Ar*H*), 5.43 (s, 2H, =CH_2).

NMR data are consistent with the literature[5]

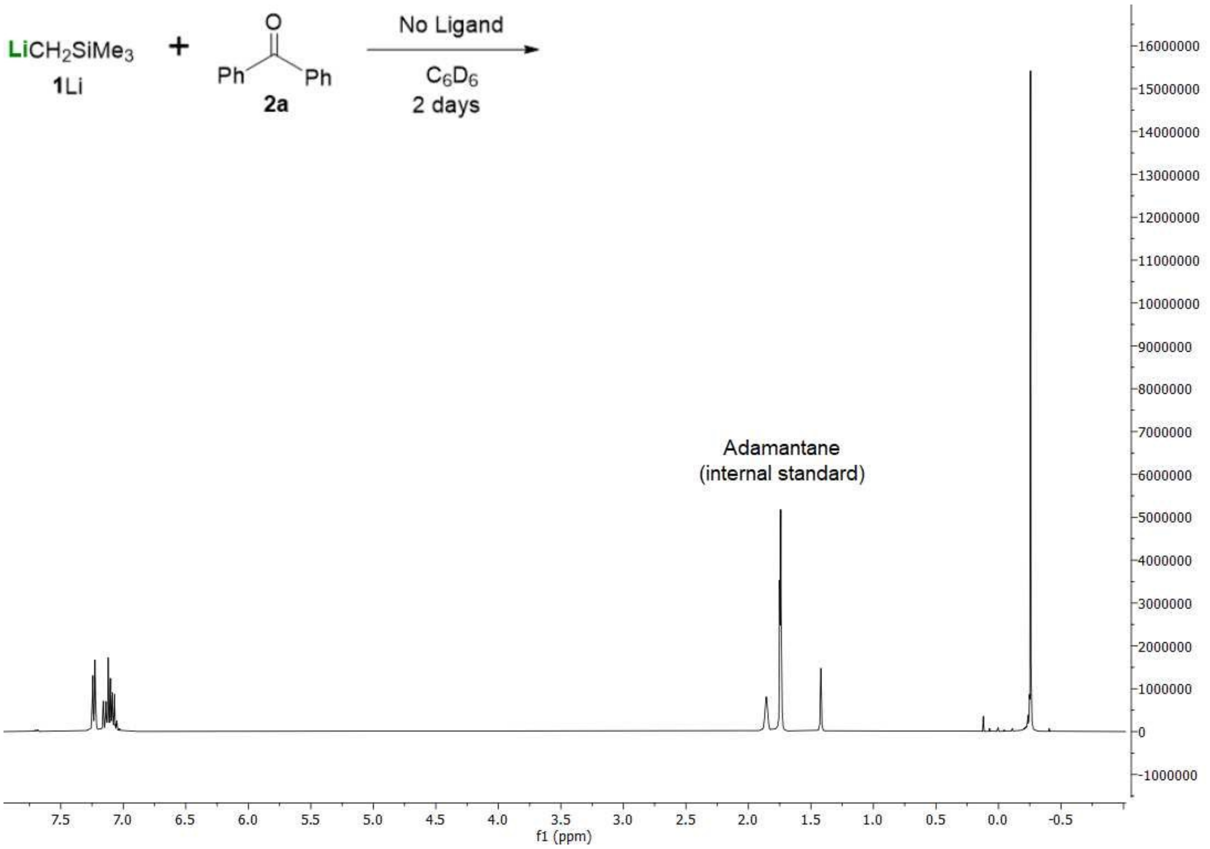

Figure S2: ^{1}H NMR (d_6-benzene, 25 ˚C, 400 MHz) of an NMR scale reaction between $LiCH_2SiMe_3$ and benzophenone (**2a**) (RT, 2 day).

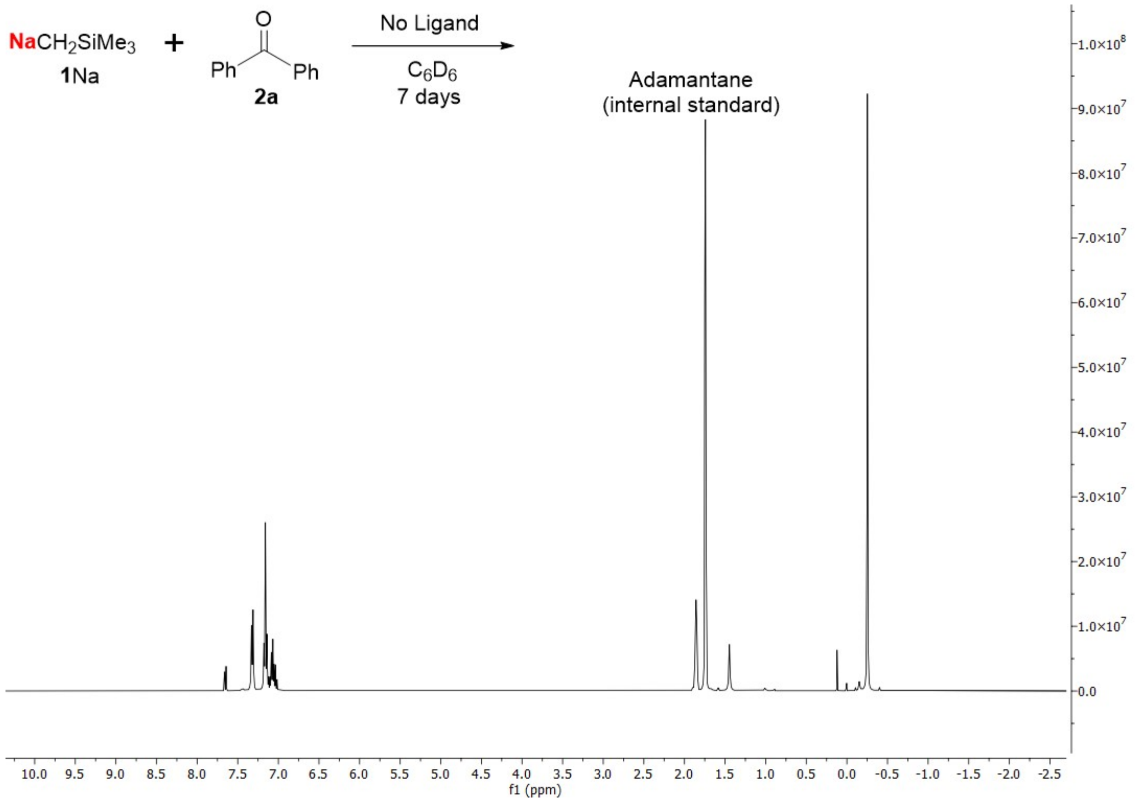

Figure S3: ^{1}H NMR (d_6-benzene, 25 ˚C, 400 MHz) of an NMR scale reaction between $NaCH_2SiMe_3$ and benzophenone (**2a**) (RT, 7 days).

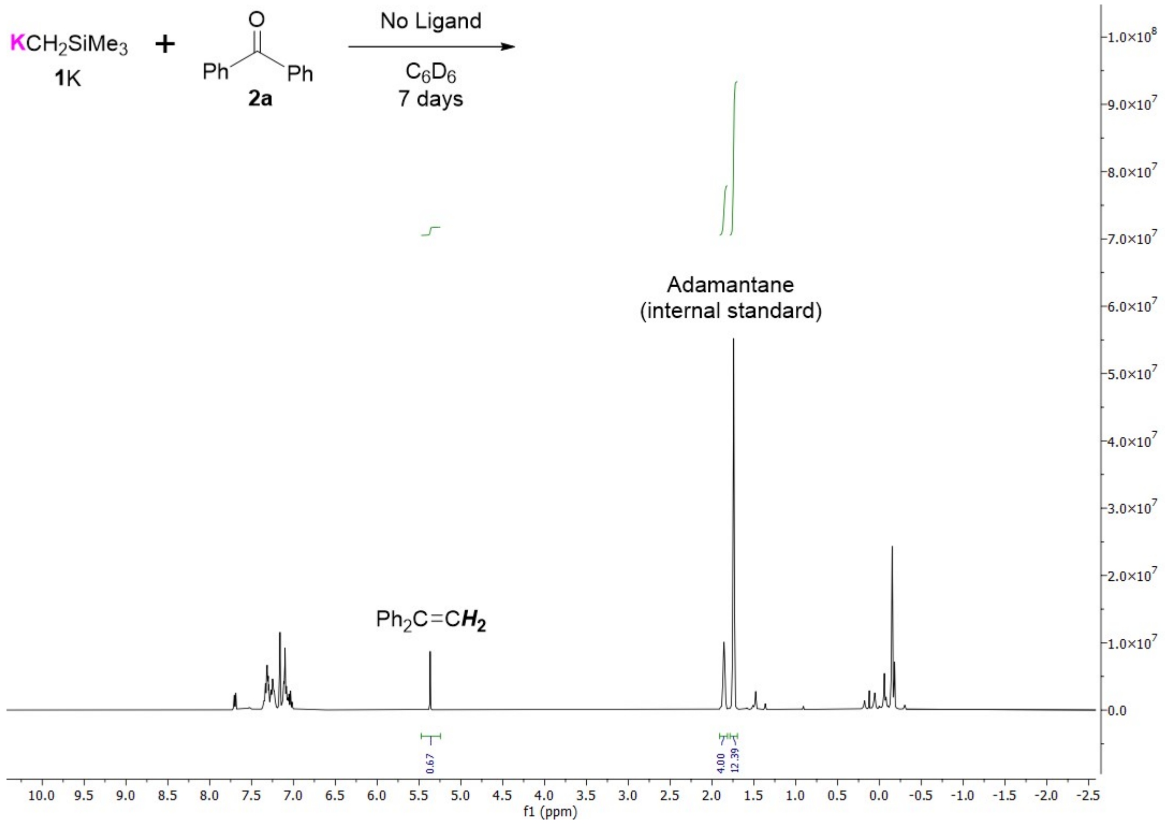

Figure S4: ^{1}H NMR (d_6-benzene, 25 ˚C, 400 MHz) of an NMR scale reaction between KCH_2SiMe_3 and benzophenone (**2a**) (RT, 7 days).

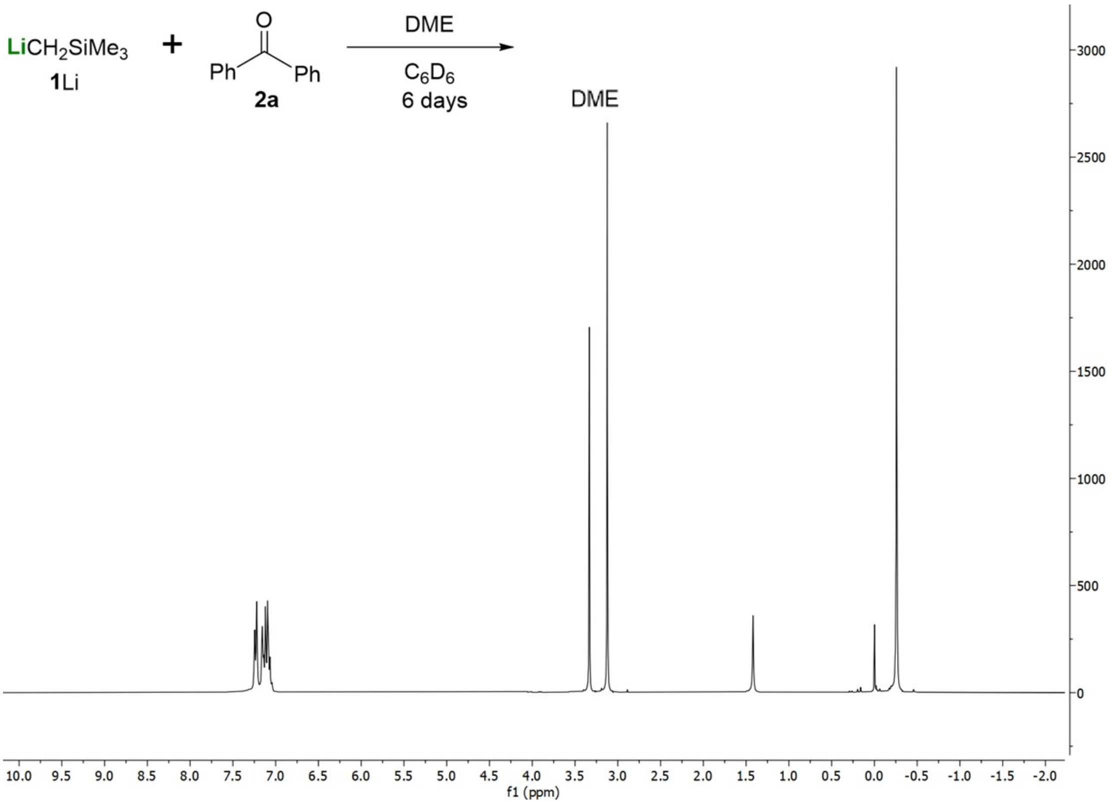

Figure S5: ^{1}H NMR (d_6-benzene, 25 ˚C, 400 MHz) of an NMR scale reaction between $LiCH_2SiMe_3$, benzophenone (**2a**) and DME (RT, 6 days).

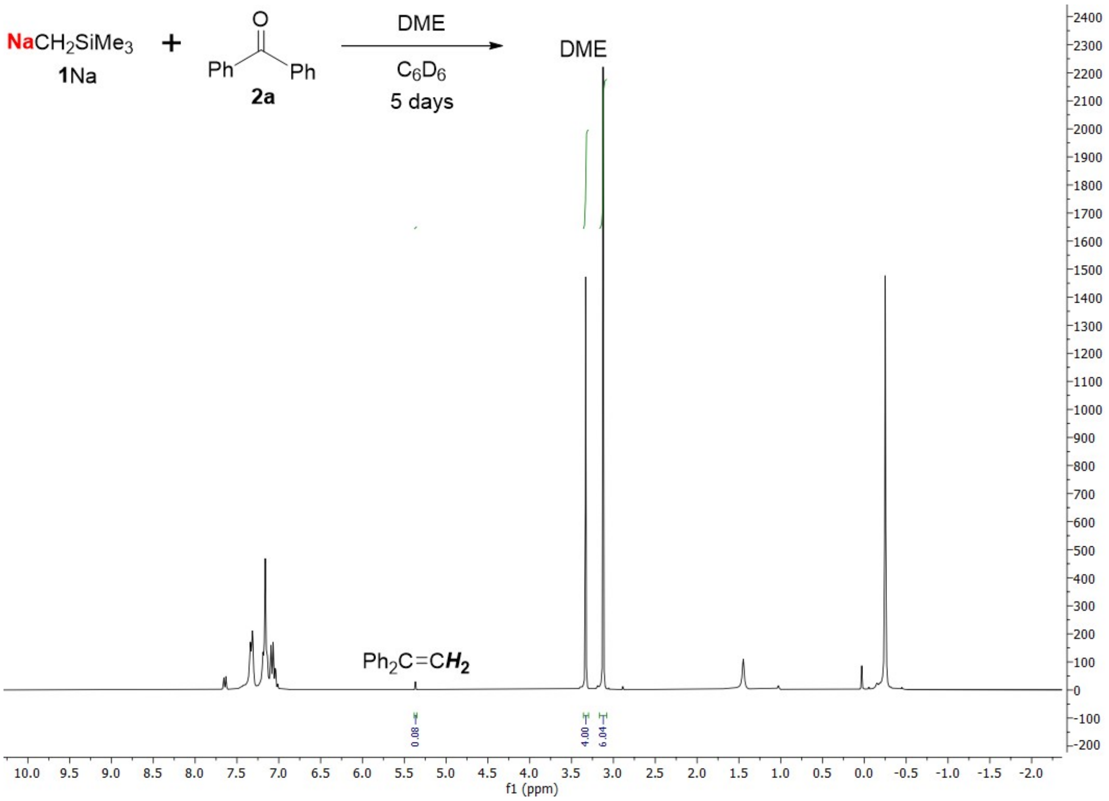

Figure S6: ^{1}H NMR (d_6-benzene, 25 ˚C, 400 MHz) of an NMR scale reaction between $NaCH_2SiMe_3$, benzophenone (**2a**) and DME (RT, 5 days).

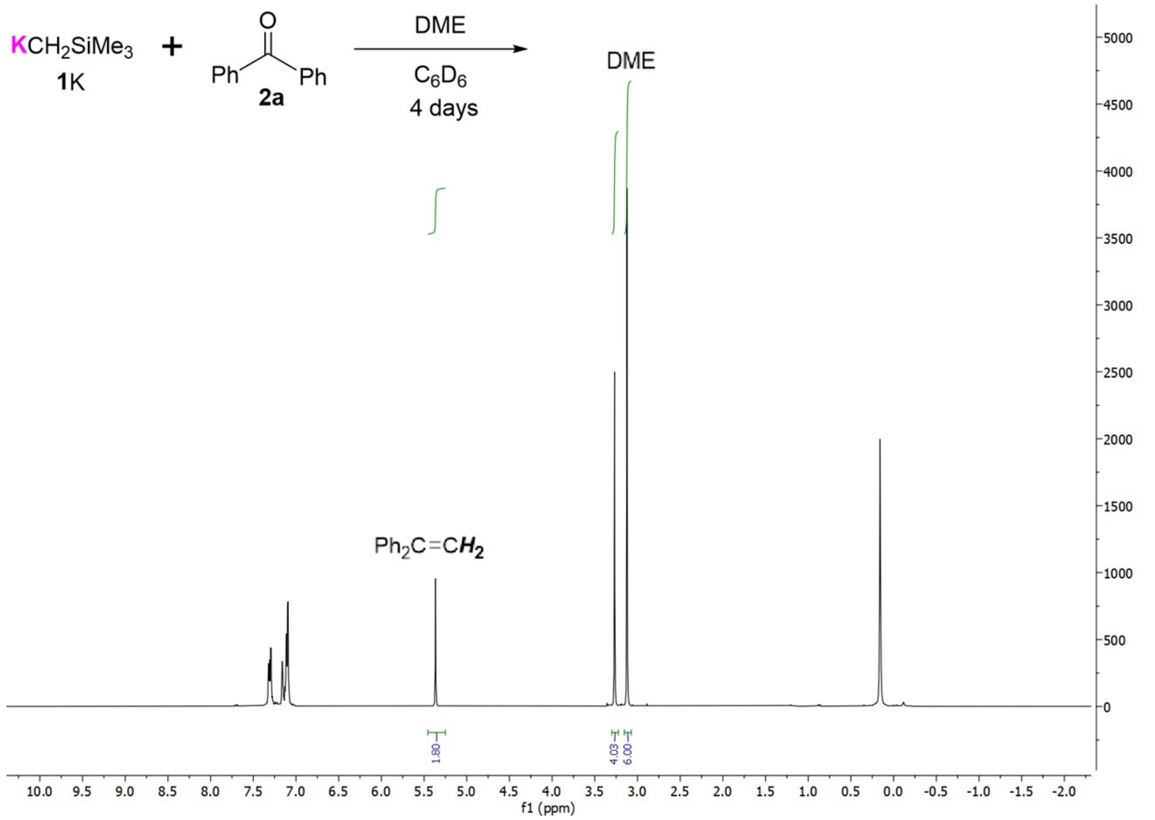

Fi
gure S7: ^{1}H NMR (d_6-benzene, 25 ˚C, 400 MHz) of an NMR scale reaction between KCH_2SiMe_3, benzophenone (**2a**) and DME (RT, 4 days).

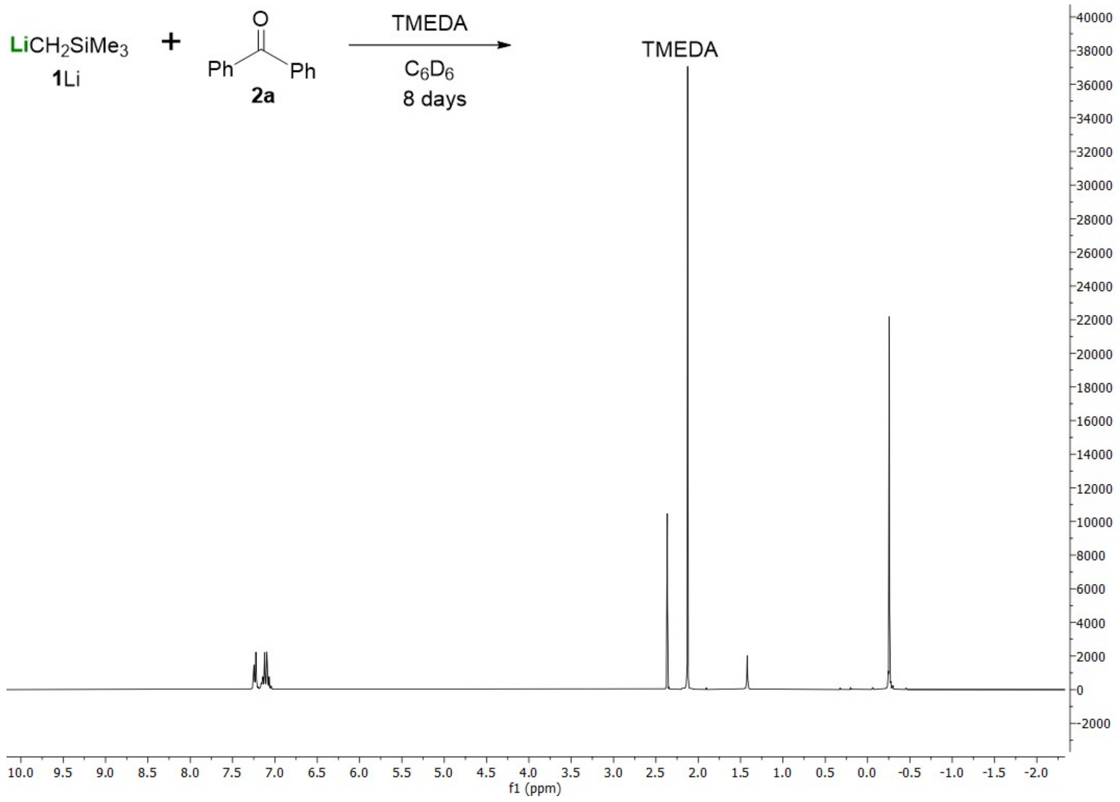

Figure S8: ^{1}H NMR (d_6-benzene, 25 ˚C, 400 MHz) of an NMR scale reaction between $LiCH_2SiMe_3$, benzophenone (**2a**) and TMEDA (RT, 8 days).

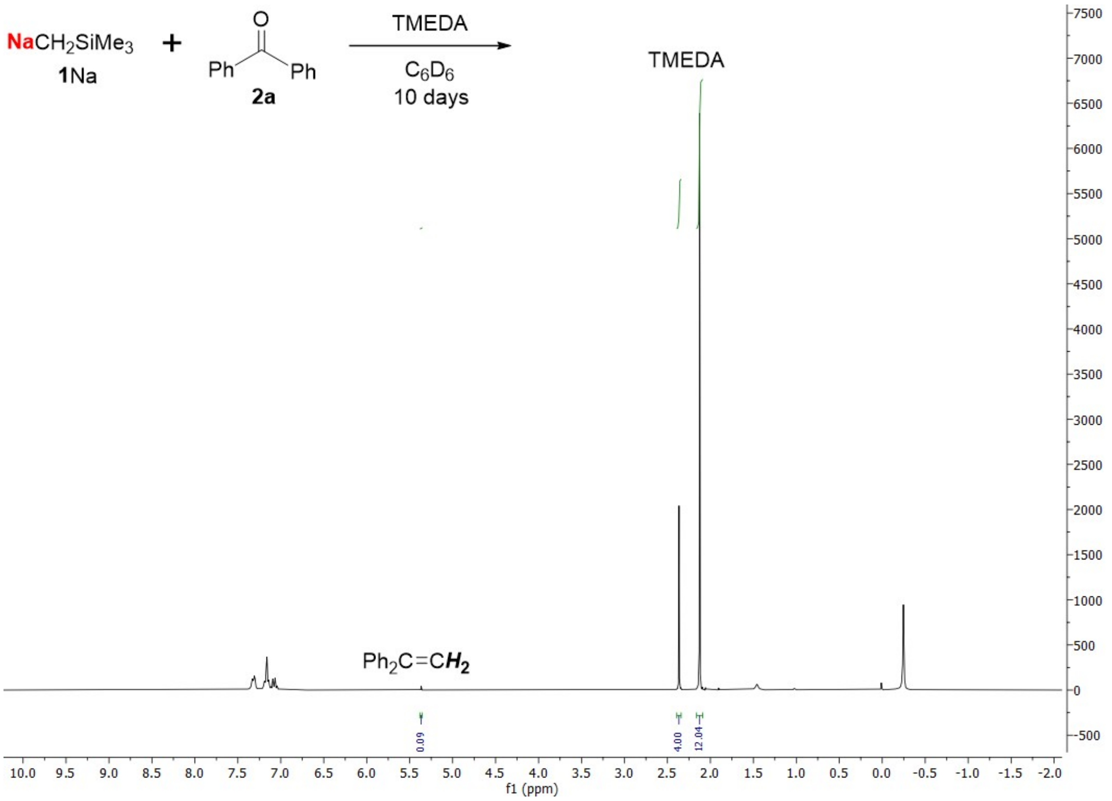

Figure S9: ^{1}H NMR (d_6-benzene, 25 ˚C, 400 MHz) of an NMR scale reaction between $NaCH_2SiMe_3$, benzophenone (**2a**) and TMEDA (RT, 10 days).

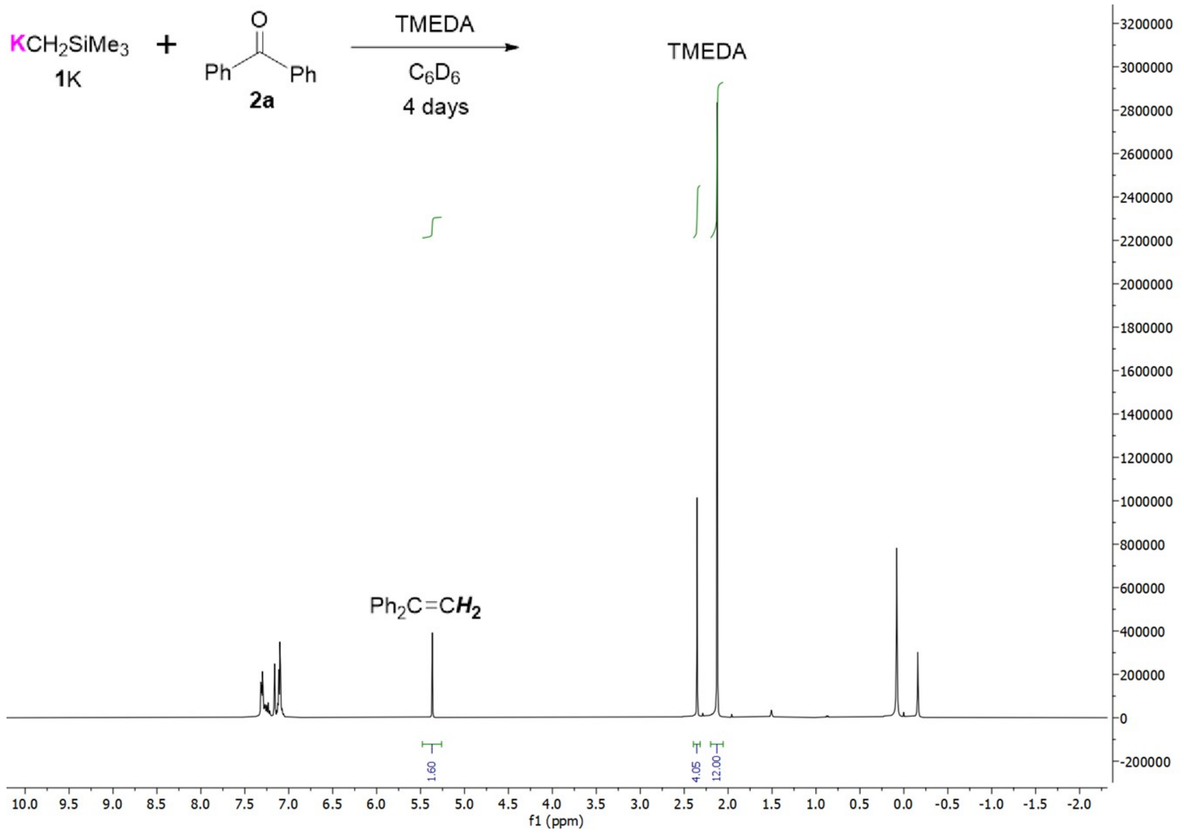

F

igure S10: ^{1}H NMR (d_6-benzene, 25 ˚C, 400 MHz) of an NMR scale reaction between KCH_2SiMe_3, benzophenone (**2a**) and TMEDA (RT, 4 days).

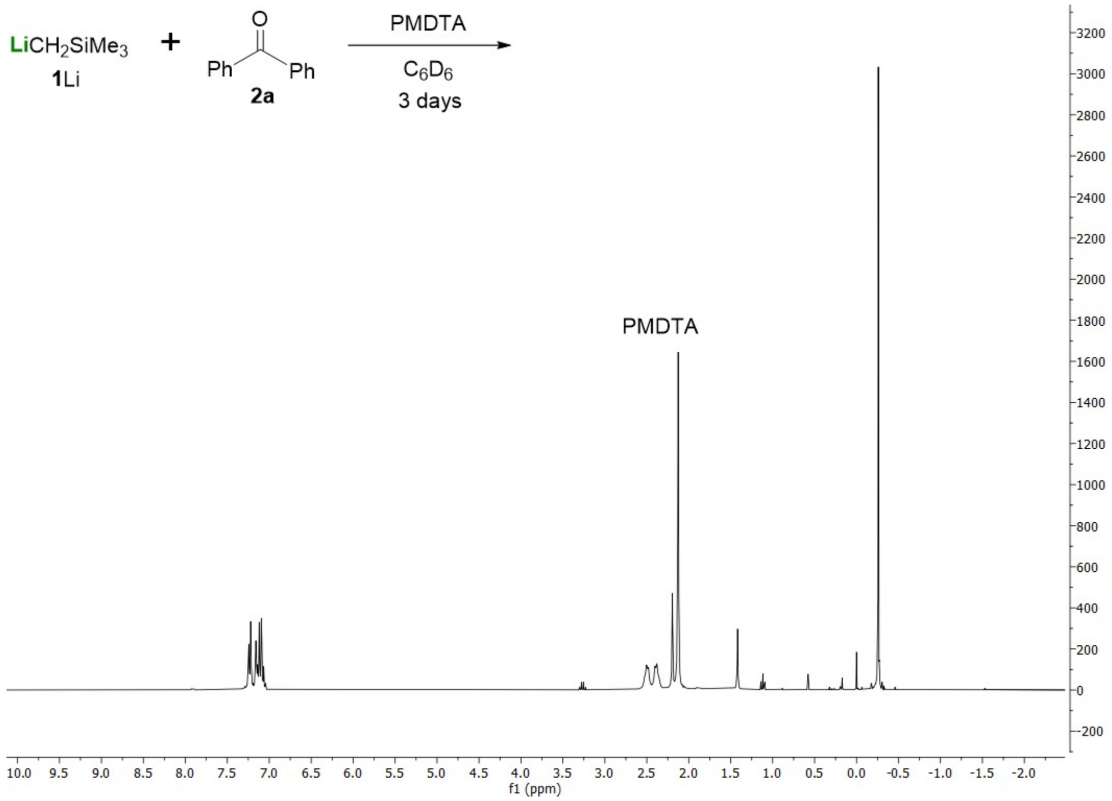

Figure S11: ^{1}H NMR (d_6-benzene, 25 ˚C, 400 MHz) of an NMR scale reaction between $LiCH_2SiMe_3$, benzophenone (**2a**) and PMDTA (RT, 3 days).

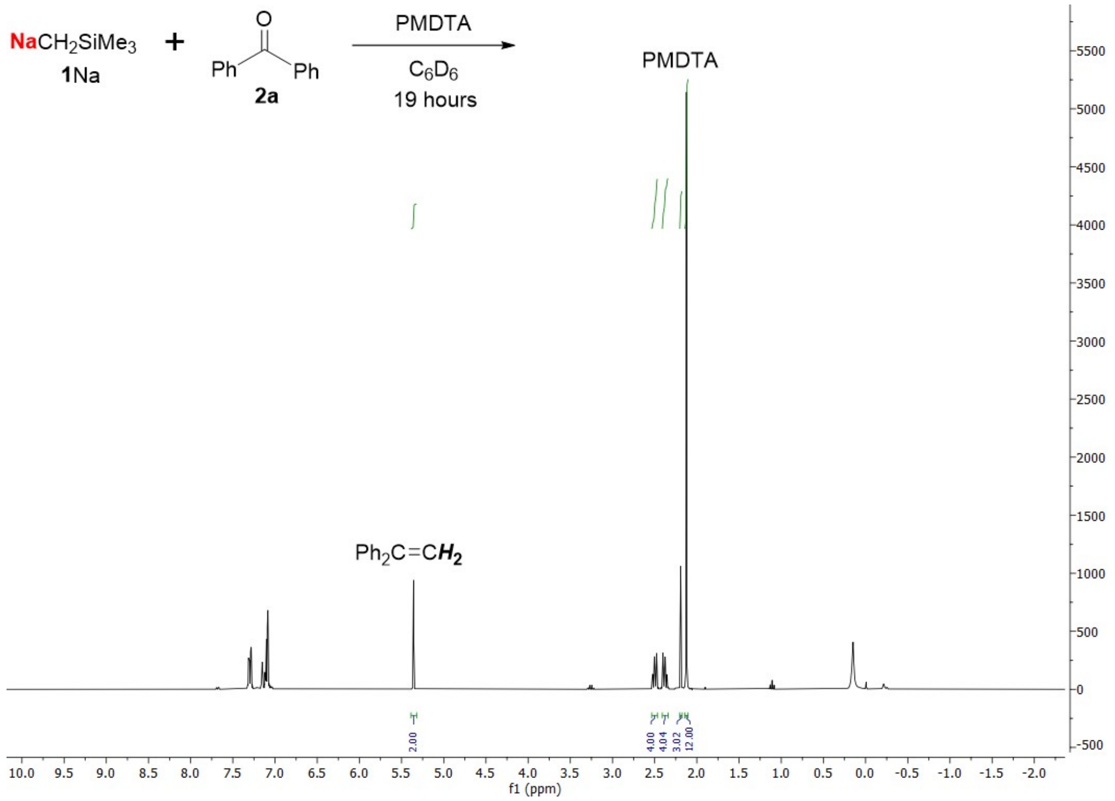

Figure S12: ^{1}H NMR (d_6-benzene, 25 ˚C, 400 MHz) of an NMR scale reaction between $NaCH_2SiMe_3$, benzophenone (**2a**) and PMDTA (RT, 19 hours).

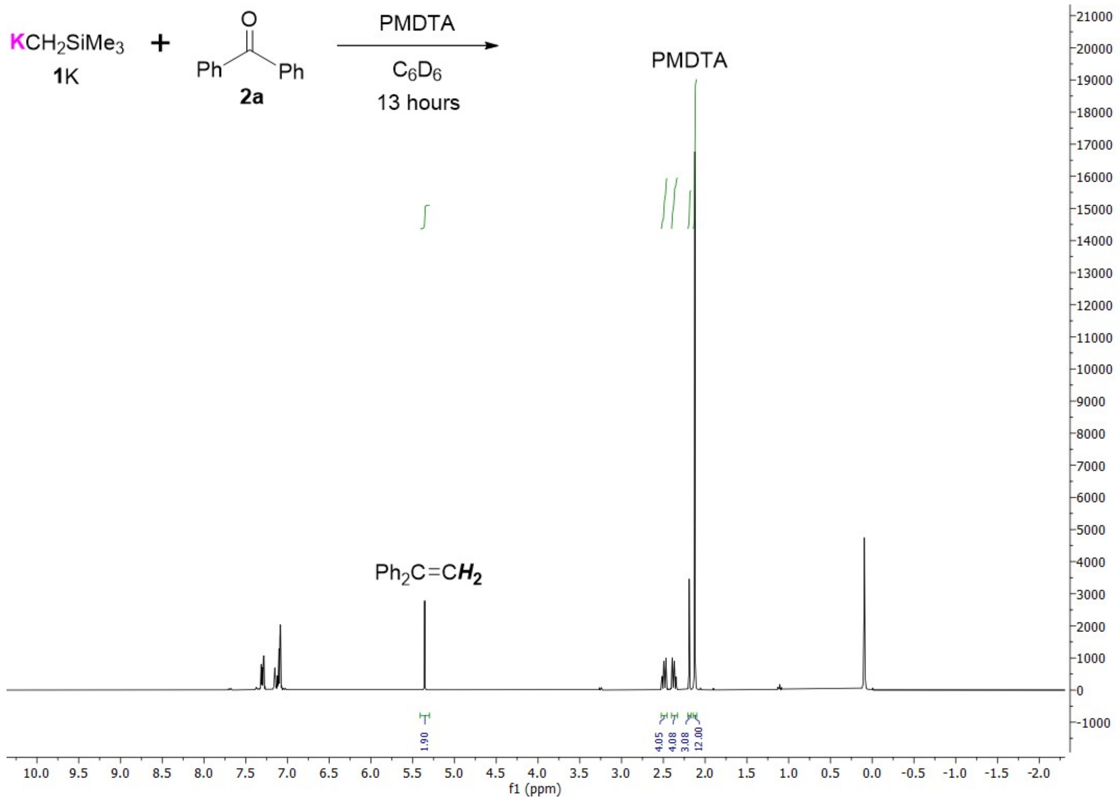

Figure S13: ^{1}H NMR (d_6-benzene, 25 ˚C, 400 MHz) of an NMR scale reaction between KCH_2SiMe_3, benzophenone (**2a**) and PMDTA (RT, 13 hours).

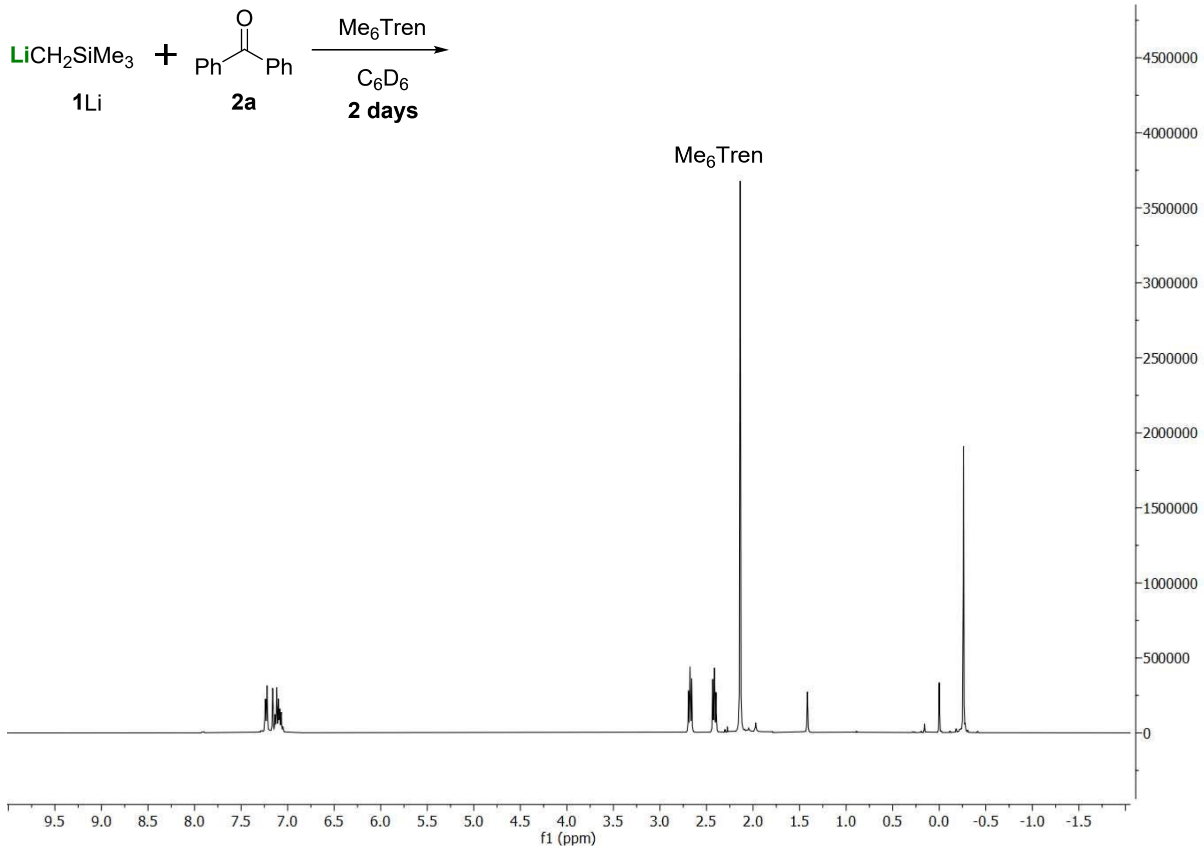

Figure S14: ^{1}H NMR (d_6-benzene, 25 ˚C, 400 MHz) of an NMR scale reaction between $LiCH_2SiMe_3$, benzophenone (**2a**) and Me_6Tren (RT, 2 days).

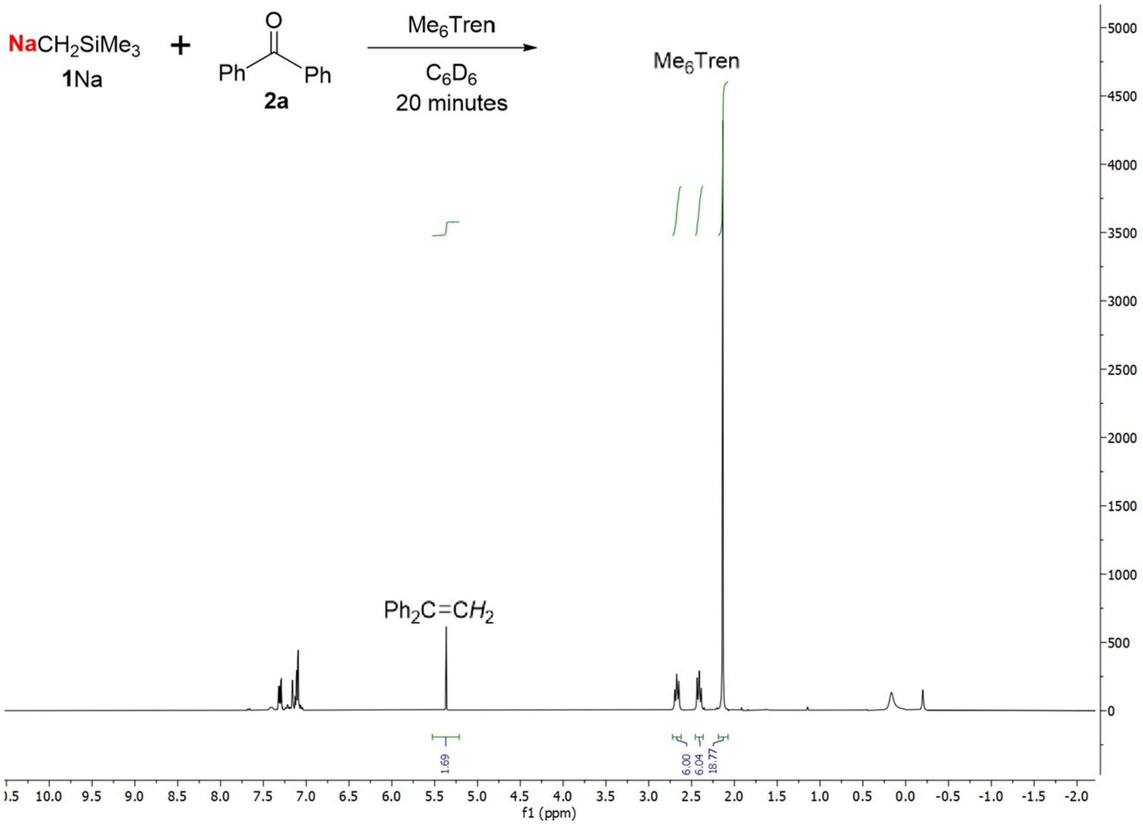

Figure S15: ^{1}H NMR (d_6-benzene, 25 ˚C, 400 MHz) of an NMR scale reaction between $NaCH_2SiMe_3$, benzophenone (**2a**) and Me_6Tren (RT, 20 mins).

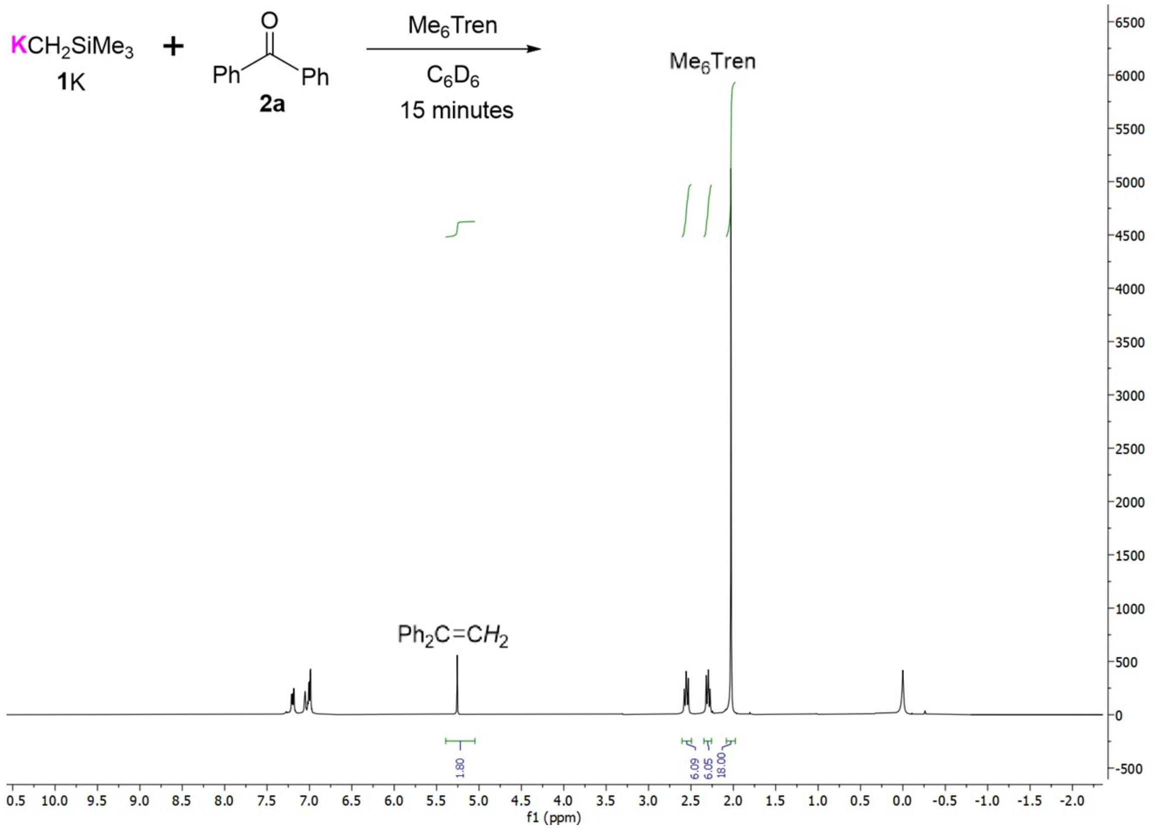

Figure S16: ^{1}H NMR (d_6-benzene, 25 ˚C, 400 MHz) of an NMR scale reaction between KCH_2SiMe_3, benzophenone (**2a**) and Me_6Tren (RT, 15 mins).

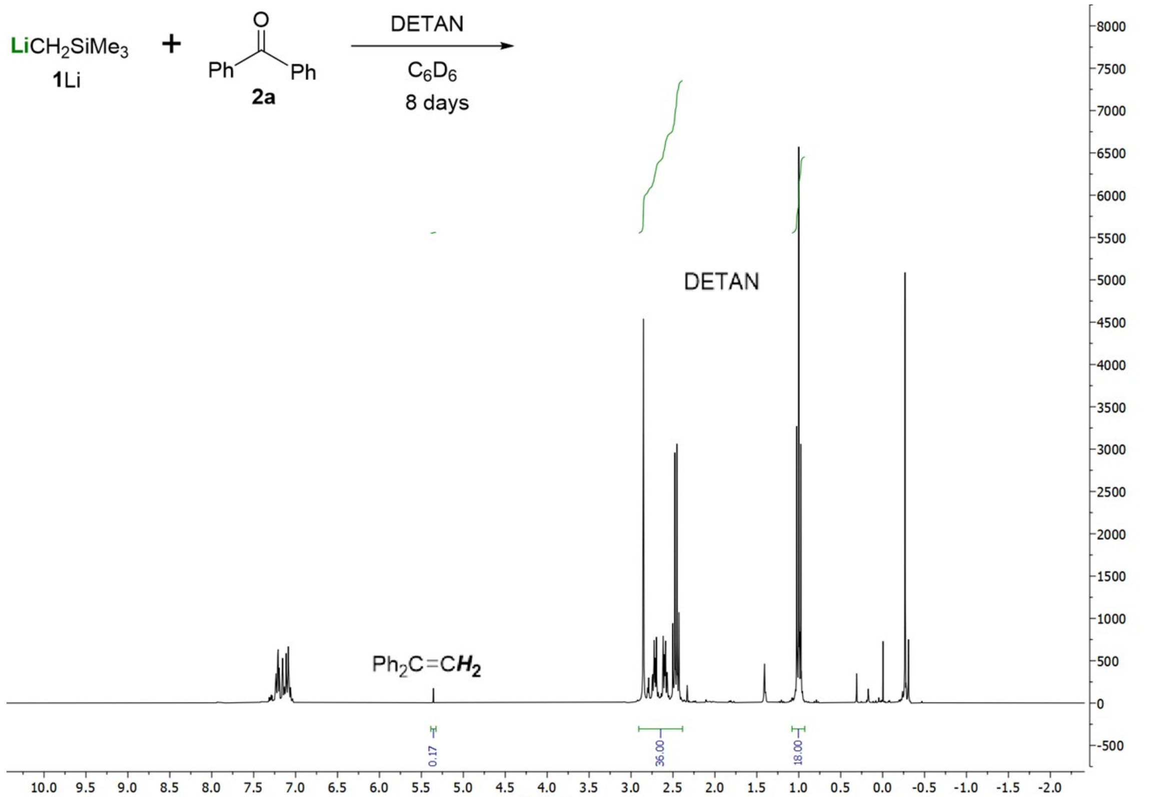

Figure S17: ^{1}H NMR (d_6-benzene, 25 ˚C, 400 MHz) of an NMR scale reaction between $LiCH_2SiMe_3$, benzophenone (**2a**) and DETAN (RT, 8 days).

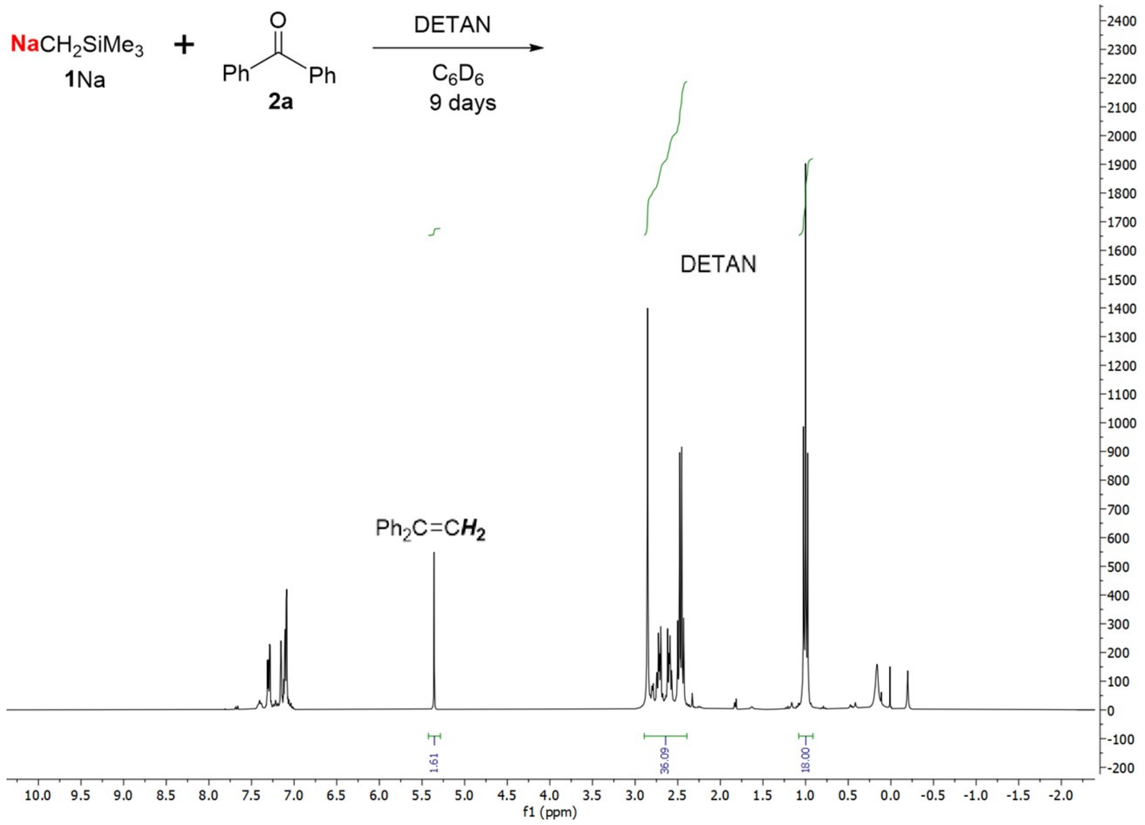

Figure S18: ^{1}H NMR (d_6-benzene, 25 ˚C, 400 MHz) of an NMR scale reaction between $NaCH_2SiMe_3$, benzophenone (**2a**) and DETAN (RT, 9 days).

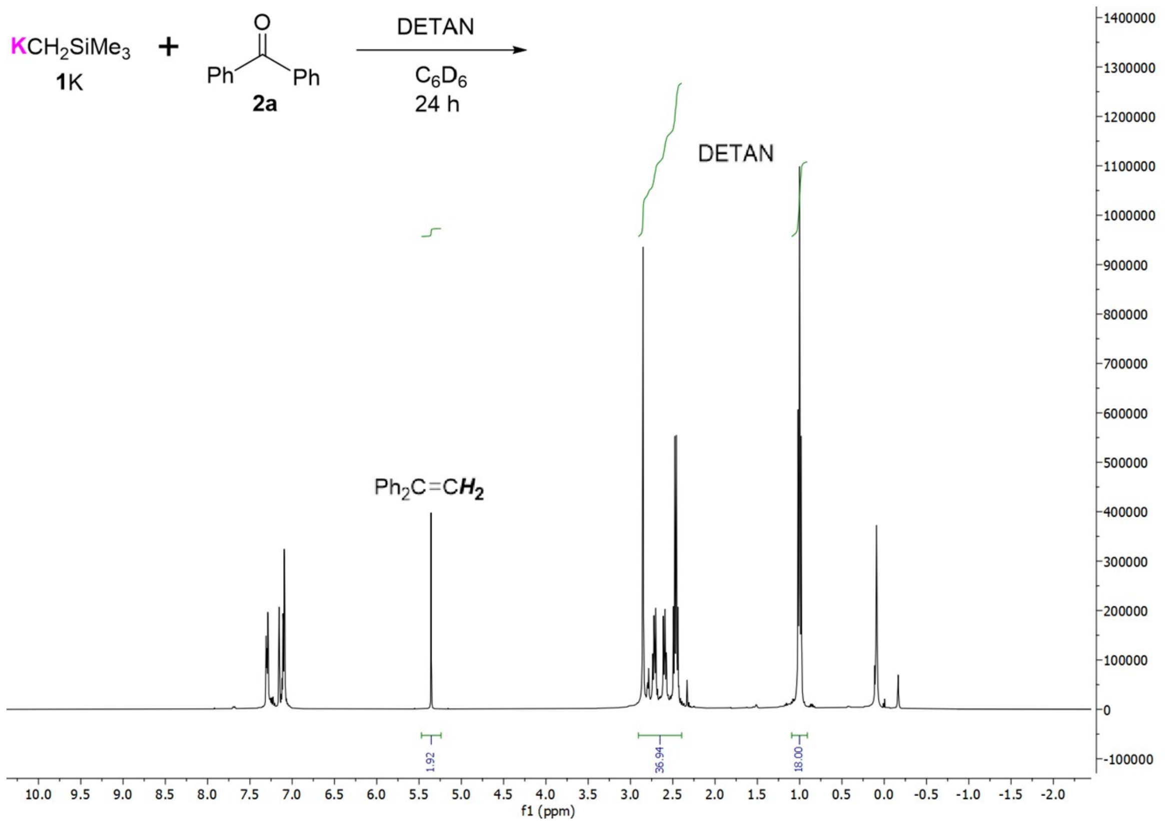

Figure S19: ^{1}H NMR (d_6-benzene, 25 ˚C, 400 MHz) of an NMR scale reaction between KCH_2SiMe_3, benzophenone (**2a**) and DETAN (RT, 24 h).

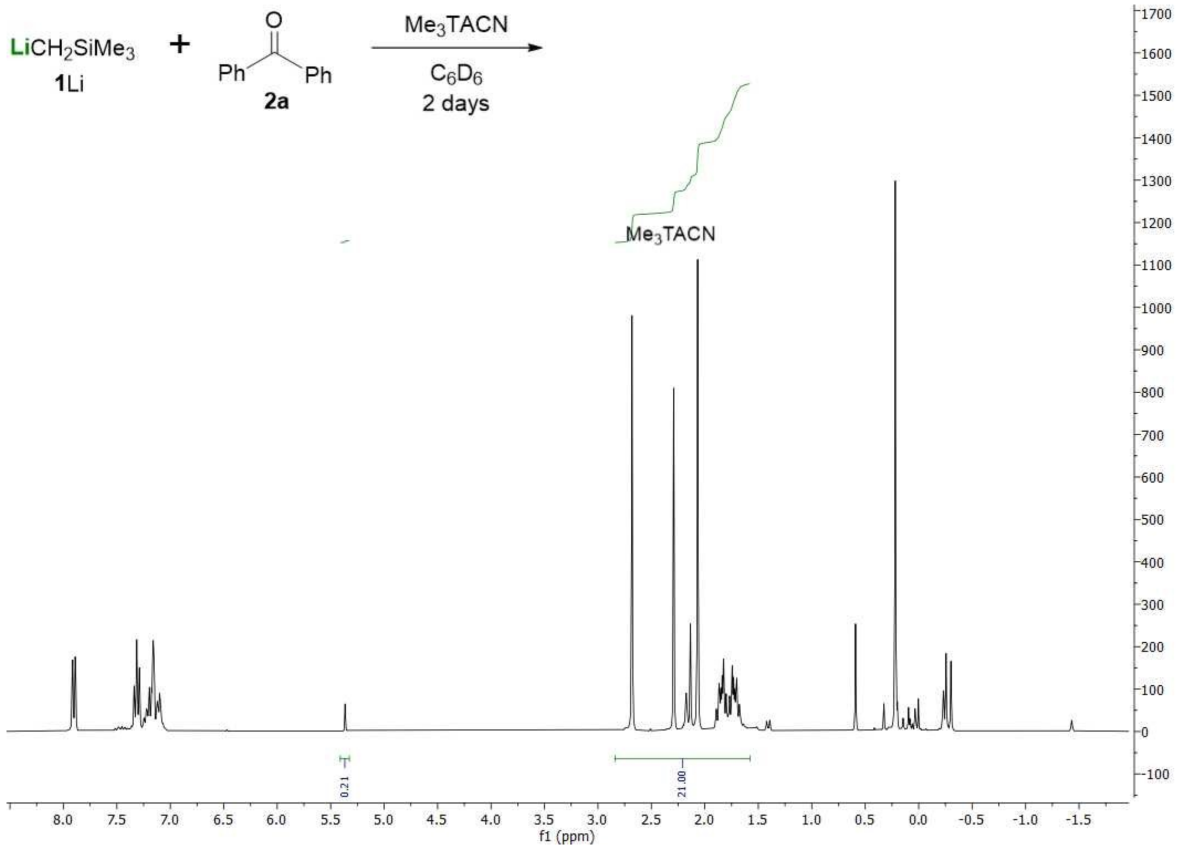

Figure S20: ^{1}H NMR (d_6-benzene, 25 ˚C, 300 MHz) and ^{7}Li NMR (d_6-benzene, 25 ˚C, 76 MHz) of an NMR scale reaction between $LiCH_2SiMe_3$, benzophenone (**2a**) and Me_3TACN (RT, 2 days).

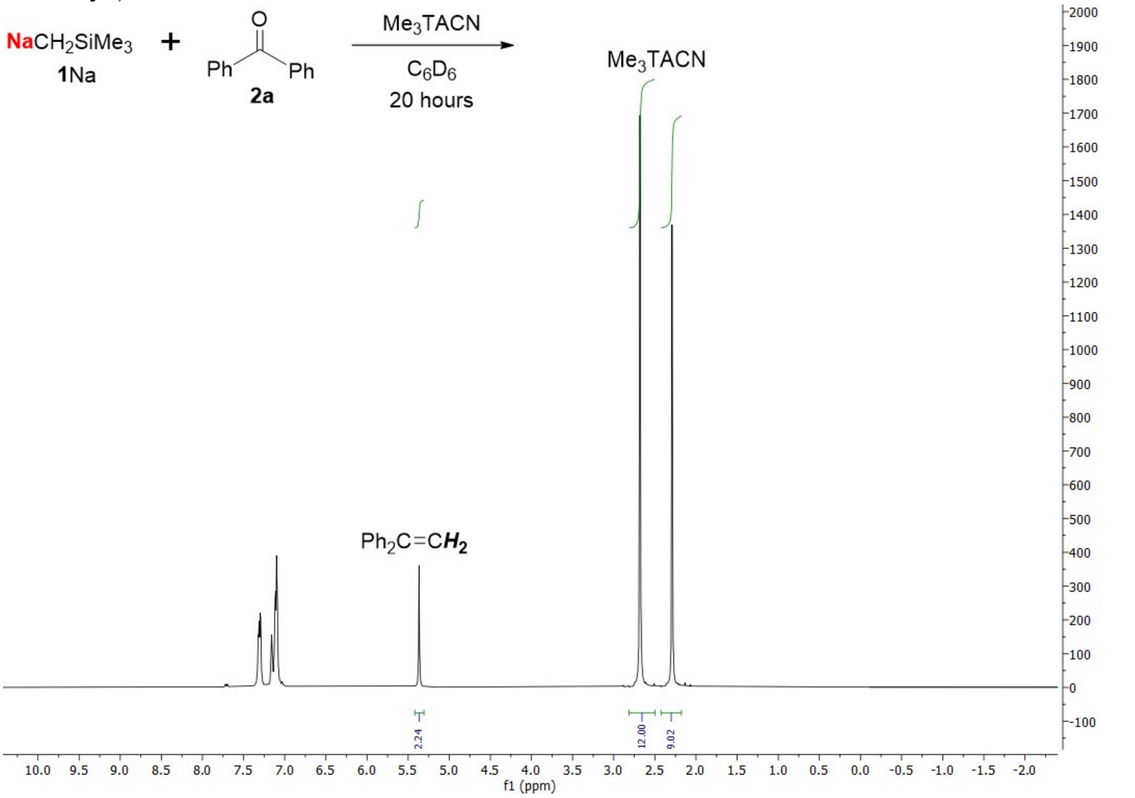

Figure S21: ^{1}H NMR (d_6-benzene, 25 ˚C, 400 MHz) of an NMR scale reaction between $NaCH_2SiMe_3$, benzophenone (**2a**) and Me_3TACN (RT, 20 hours).

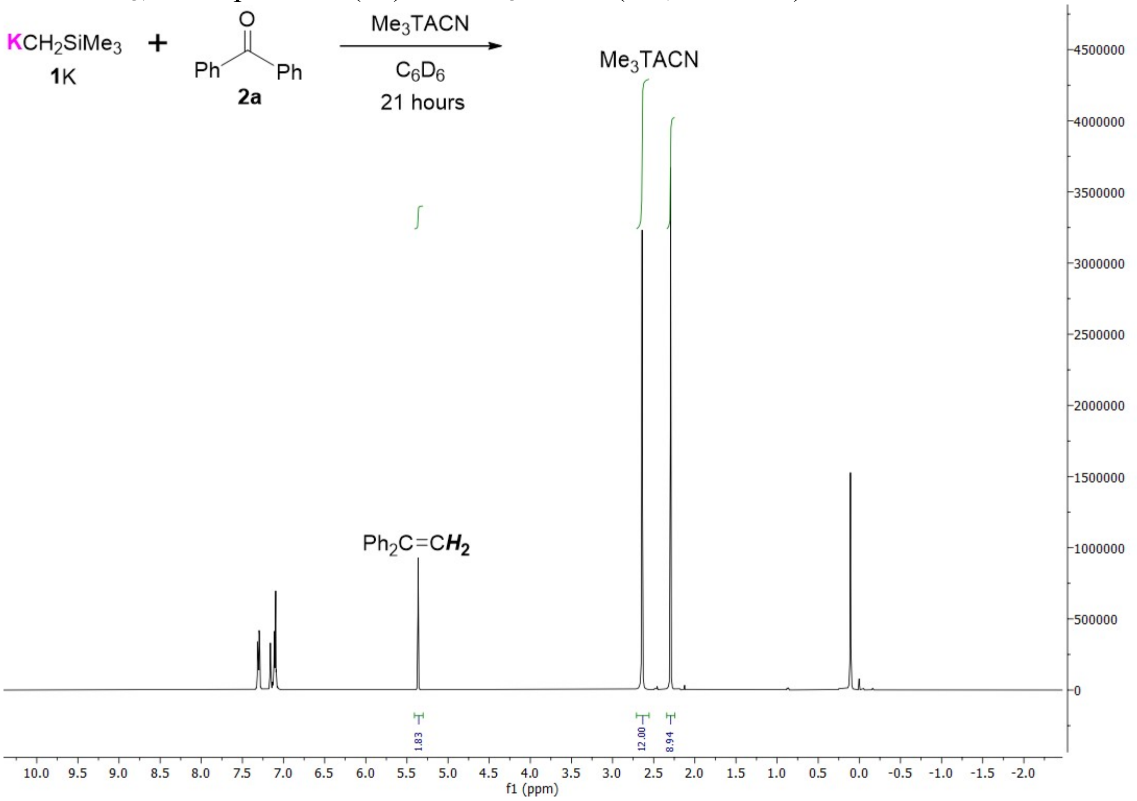

Figure S22: ^{1}H NMR (d_6-benzene, 25 ˚C, 400 MHz) of an NMR scale reaction between KCH_2SiMe_3, benzophenone (**2a**) and Me_3TACN (RT, 21 hours).

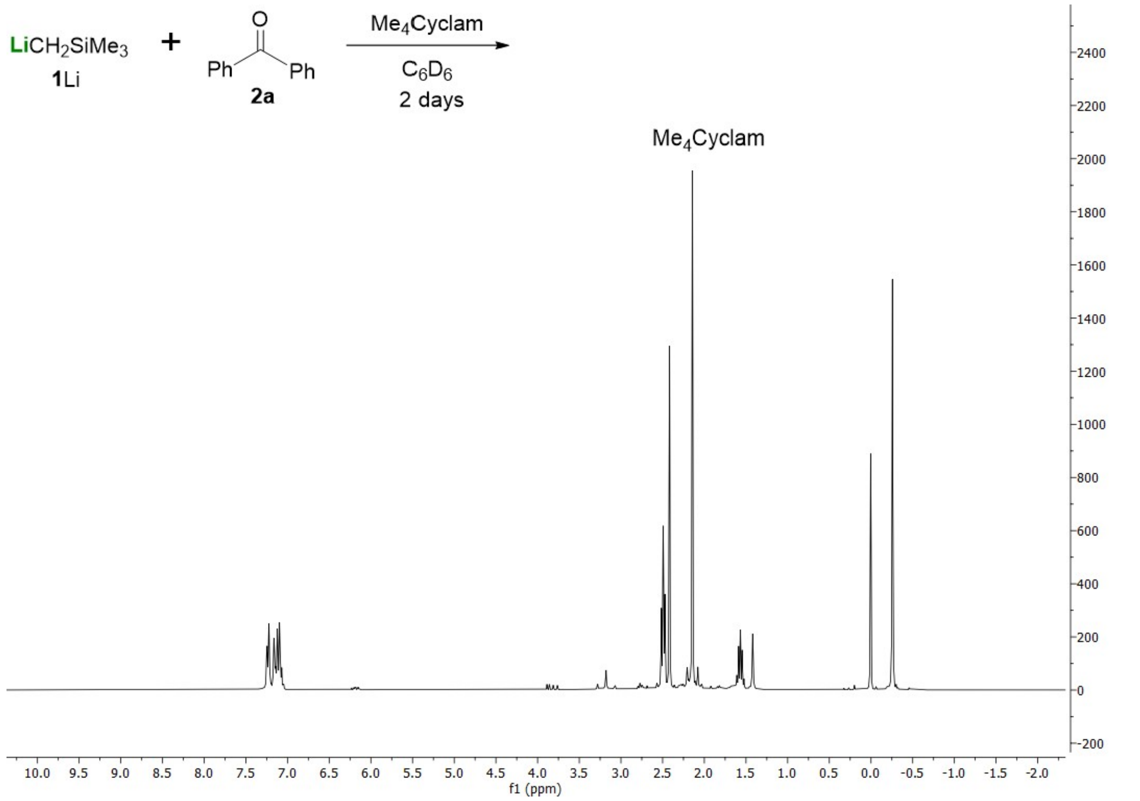

Figure S23: ^{1}H NMR (d_6-benzene, 25 ˚C, 400 MHz) of an NMR scale reaction between $LiCH_2SiMe_3$, benzophenone (**2a**) and $Me_4Cyclam$ (RT, 2 days).

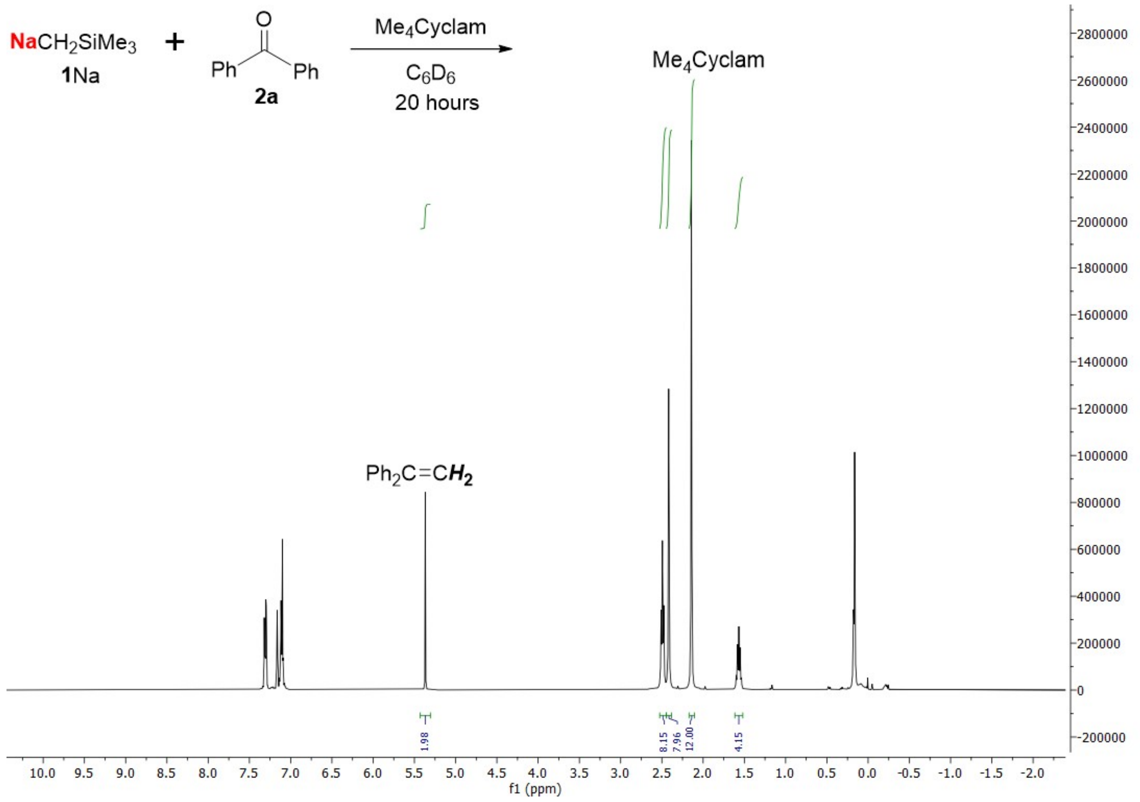

Figure S24: ^{1}H NMR (d_6-benzene, 25 ˚C, 400 MHz) of an NMR scale reaction between $NaCH_2SiMe_3$, benzophenone (**2a**) and Me_4Cyclam (RT, 20 hours).

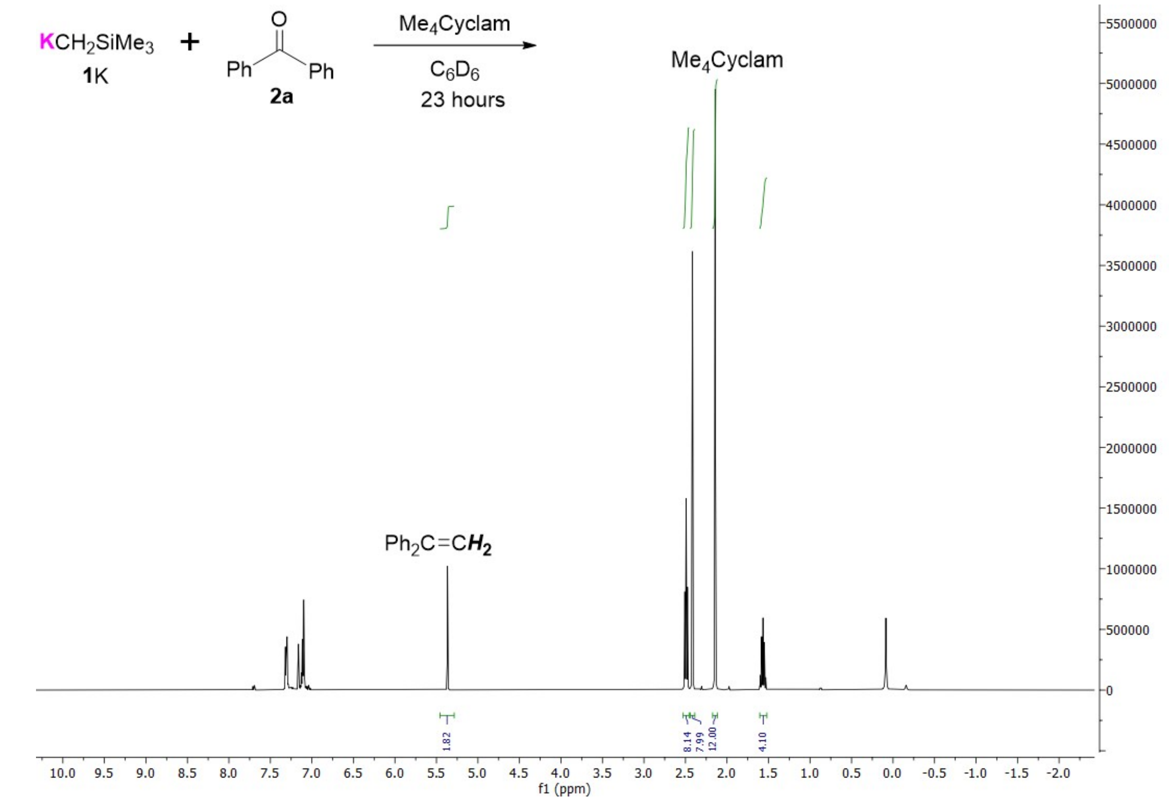

Figure S25: ^{1}H NMR (d_6-benzene, 25 ˚C, 400 MHz) of an NMR scale reaction between KCH_2SiMe_3, benzophenone (**2a**) and Me_4Cyclam (RT, 23 hours).

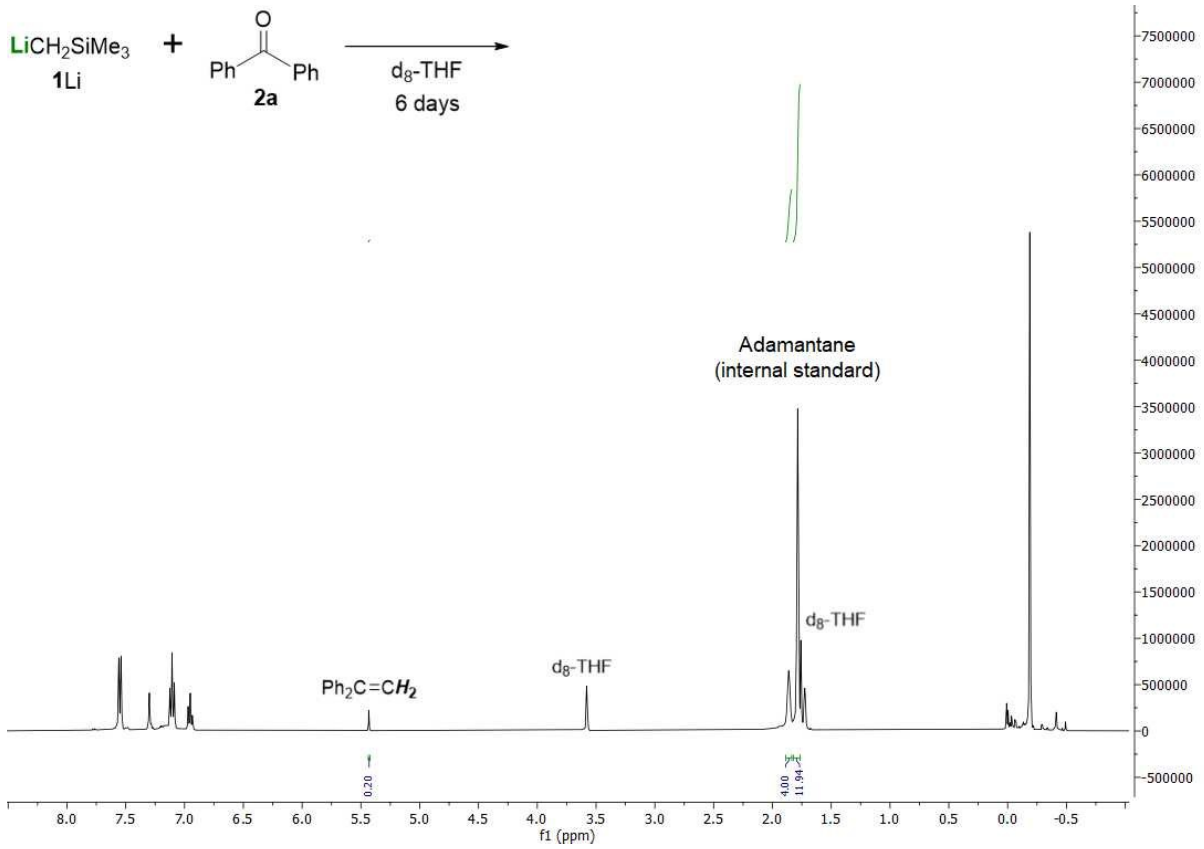

Figure S26: ^{1}H NMR (d_8-THF, 25 ˚C, 400 MHz) of an NMR scale reaction between $LiCH_2SiMe_3$ and benzophenone (**2a**) (RT, 6 days).

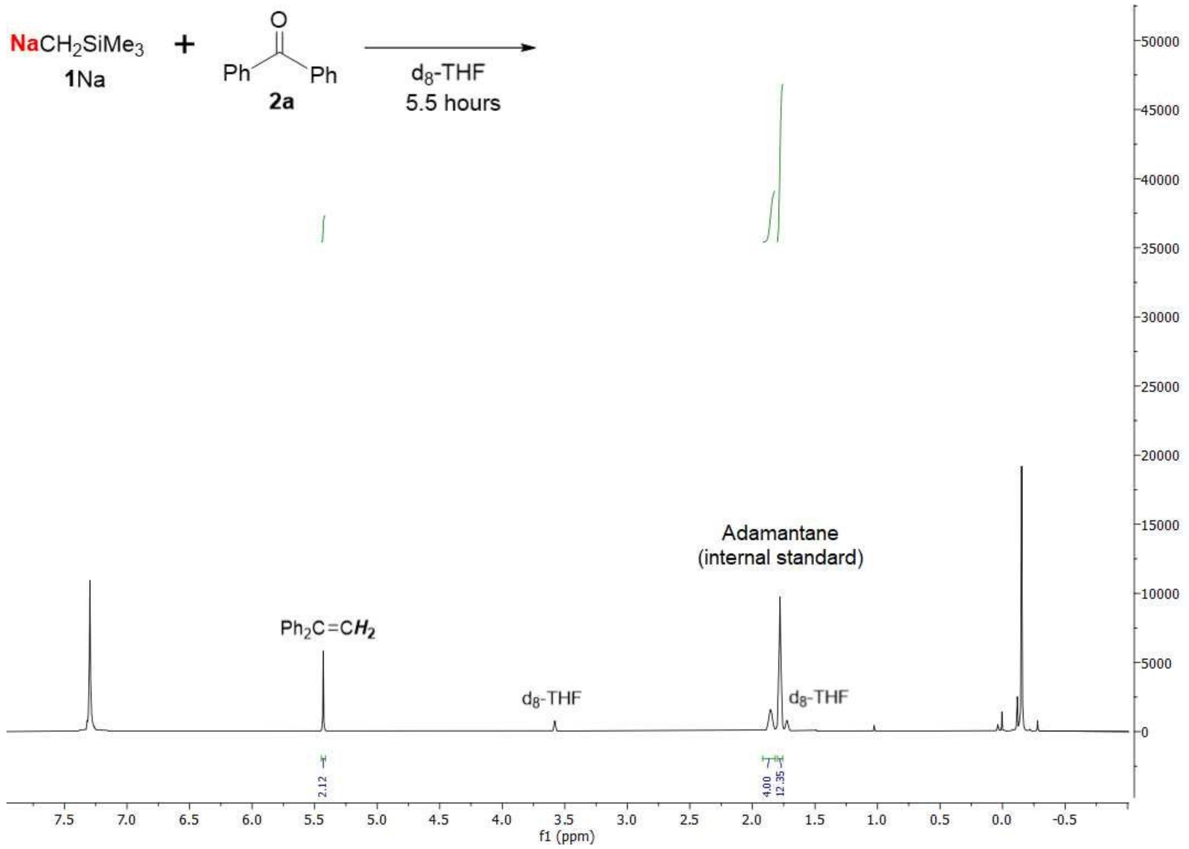

Figure S27: ^{1}H NMR (d_8-THF, 25 ˚C, 300 MHz) of an NMR scale reaction between $NaCH_2SiMe_3$ and benzophenone (**2a**) (RT, 5.5 hours).

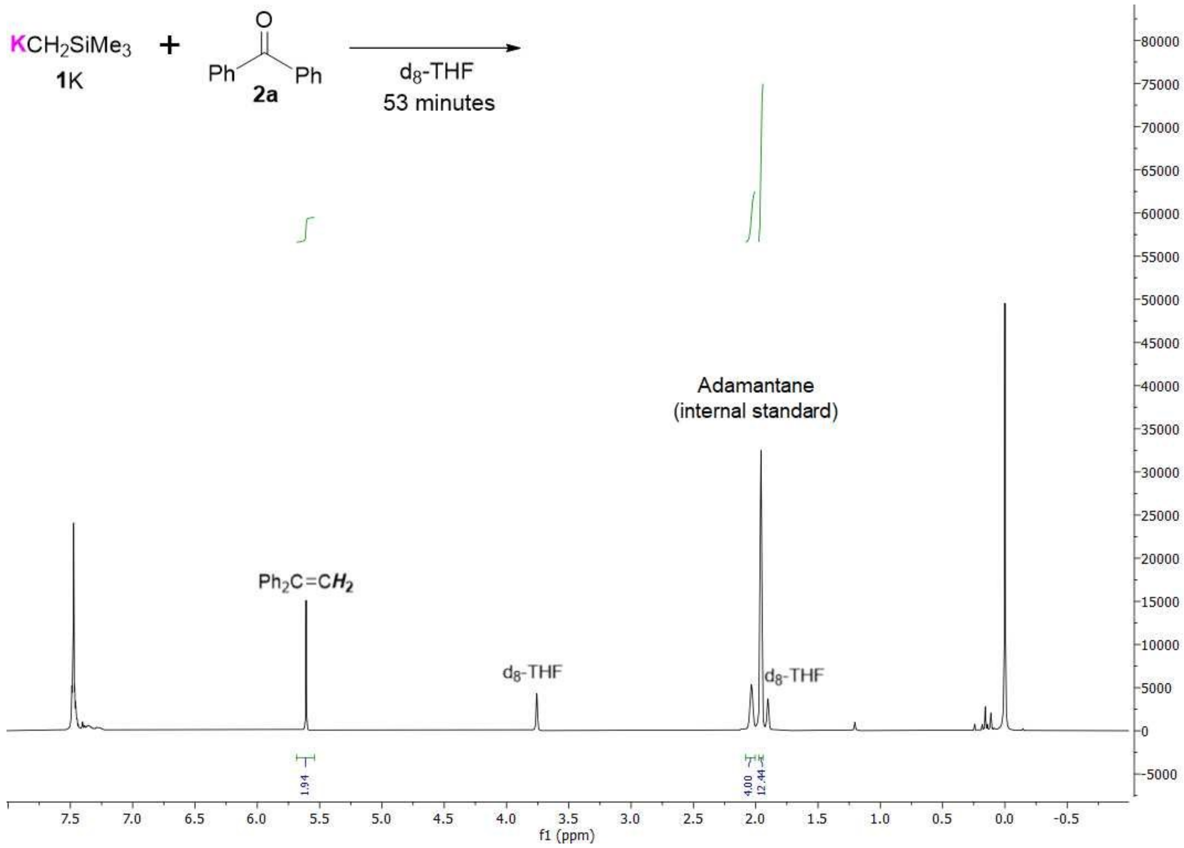

Figure S28: ^{1}H NMR (d_8-THF, 25 ˚C, 400 MHz) of an NMR scale reaction between KCH_2SiMe_3 and benzophenone (**2a**) (RT, 53 mins).

1.3 NMR-scale Transmetallation Reactions

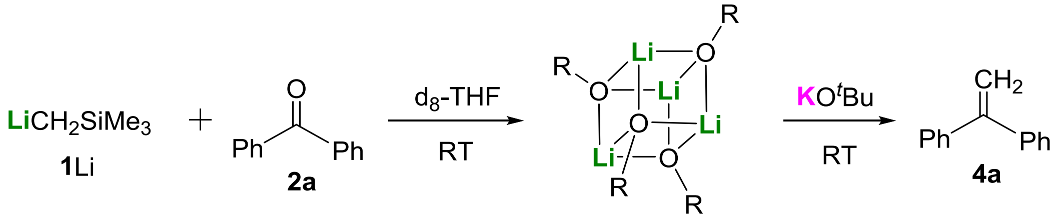

3Li; R = $CPh_2CH_2SiMe_3$

Procedure for the synthesis of 3 Li

Benzophenone (0.3644 g, 2.0 mmol) was dissolved in benzene (2.0 mL). The resulting colourless solution was added to $LiCH_2SiMe_3$ (0.1883 g, 2.0 mmol) in a one-portion manner at room temperature. The resulting transparent pale green solution was left at room temperature for 19 hours before the volatiles were removed *in vacuo*. A pale green to white solid was obtained and characterised by ^{1}H NMR, which is matched with our previous work.[1]

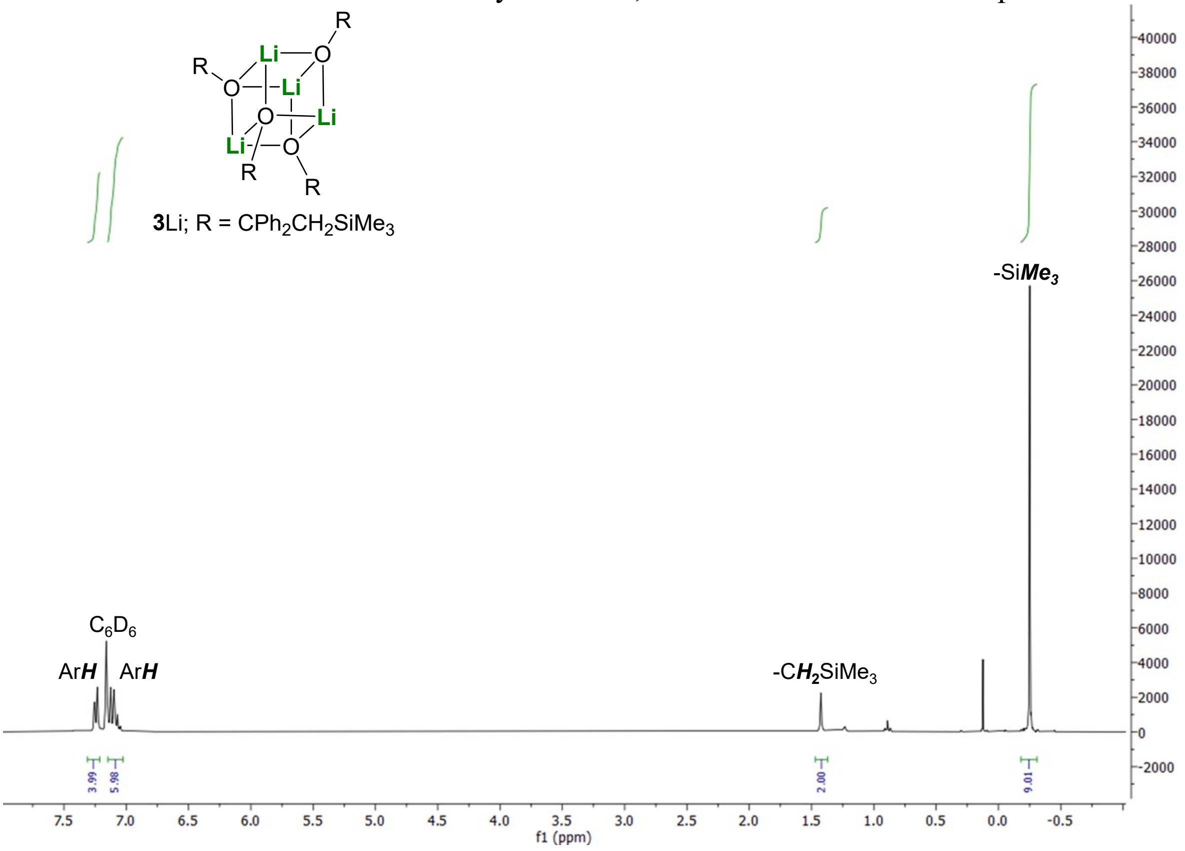

Figure S29: ^{1}H NMR (d_6-benzene, 25 ˚C, 300 MHz) of solid of **3**Li.

Procedure for benzophenone + $LiCH_2SiMe_3$ + KO^{t}Bu in C_6D_6

Benzophenone (0.0073 g, 0.04 mmol) was dissolved in C_6D_6 (0.5 ml). The solution was added to $LiCH_2SiMe_3$ (0.0038 g, 0.04 mmol) in a one-portion manner at room temperature. The resulting solution was added to KO^{t}Bu (0.0045 g, 0.04 mmol) in a one-portion manner at room temperature.

The resulting solution was transferred to a J. Young NMR tube. The reaction was monitored by NMR spectroscopy.

Procedure for 3Li + KO^{t}Bu in C_6D_6

3Li (0.0111 g, 0.04 mmol) was dissolved in C_6D_6 (0.5 ml). The solution was added to KO^{t}Bu (0.0045 g, 0.04 mmol) in a one-portion manner at room temperature.

The resulting solution was transferred to a J. Young NMR tube. The reaction was monitored by NMR spectroscopy.

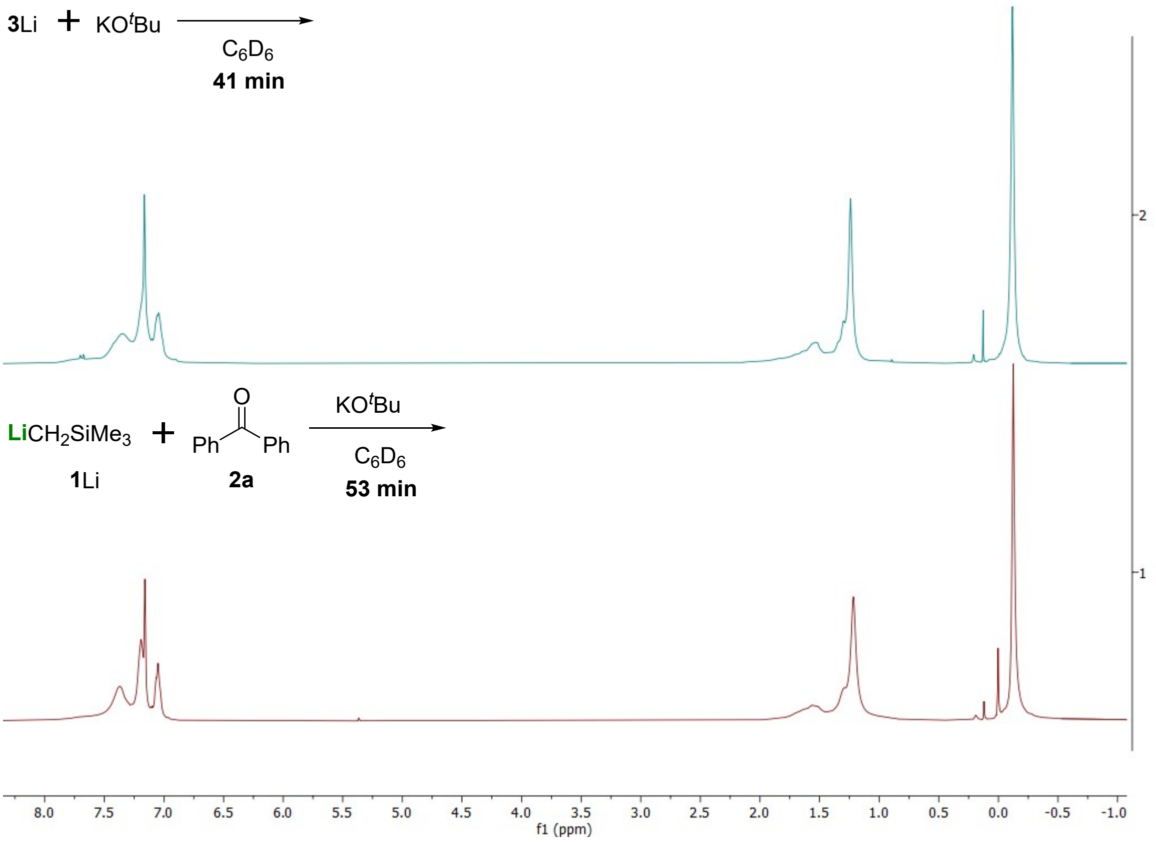

Figure S30: ^{1}H NMR (d$_6$-benzene, 25 ˚C, 300 MHz) of reaction between **3**Li and KO^{t}Bu (41 min), top; ^{1}H NMR (d$_6$-benzene, 25 ˚C, 400 MHz) of reaction between **1**Li, benzophenone and KO^{t}Bu (53 min), bottom.

Procedure for benzophenone + $LiCH_2SiMe_3$ + KO^{t}Bu in d_8-THF + cyclohexane (internal standard)

Benzophenone (0.0073 g, 0.04 mmol) was dissolved in a solution of cyclohexane (internal standard, 0.04 mmol) in d_8-THF (0.5 ml). The solution was added to $LiCH_2SiMe_3$ (0.0038 g, 0.04 mmol) in a one-portion manner at room temperature. The resulting solution was added to KO^{t}Bu (0.0045 g, 0.04 mmol) in one-portion manner at room temperature.

The resulting solution was transferred to a J. Young NMR tube. The reactions were monitored by NMR spectroscopy for methylenation (conversion: > 95%).

Procedure for control reaction benzophenone + $LiCH_2SiMe_3$ + cyclohexane (internal standard) in d_8-THF (no KO^{t}Bu)

Benzophenone (0.0073 g, 0.04 mmol) was dissolved in a solution of cyclohexane (internal standard, 0.04 mmol) in d_8-THF (0.5 ml). The solution was added to $LiCH_2SiMe_3$ (0.0038 g, 0.04 mmol) in a one-portion manner at room temperature.

The resulting solution was transferred to a J. Young NMR tube. The reactions were monitored by NMR spectroscopy for methylenation (conversion: < 5%).

^{1}H NMR of 1,1-diphenylethylene (**4a**) (400 MHz, d_8-THF, 25 °C): δ (ppm) 7.30 (s, 10H, Ar*H*), 5.43 (s, 2H, =C*H*$_2$).

NMR data are consistent with the literature.[5]

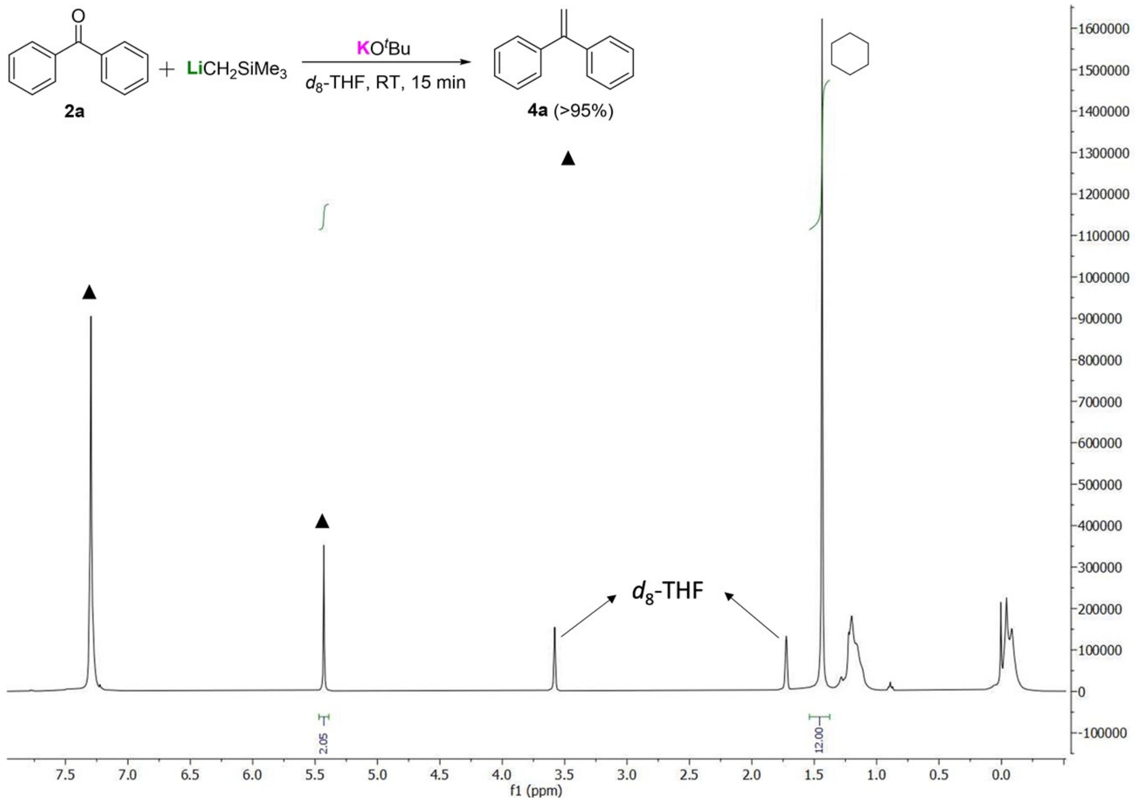

Figure S31: ^{1}H NMR (d_8-THF, 25 ˚C, 400 MHz) of an NMR scale transmetallation reaction between benzophenone (**2a**), $LiCH_2SiMe_3$ and KO^{t}Bu.

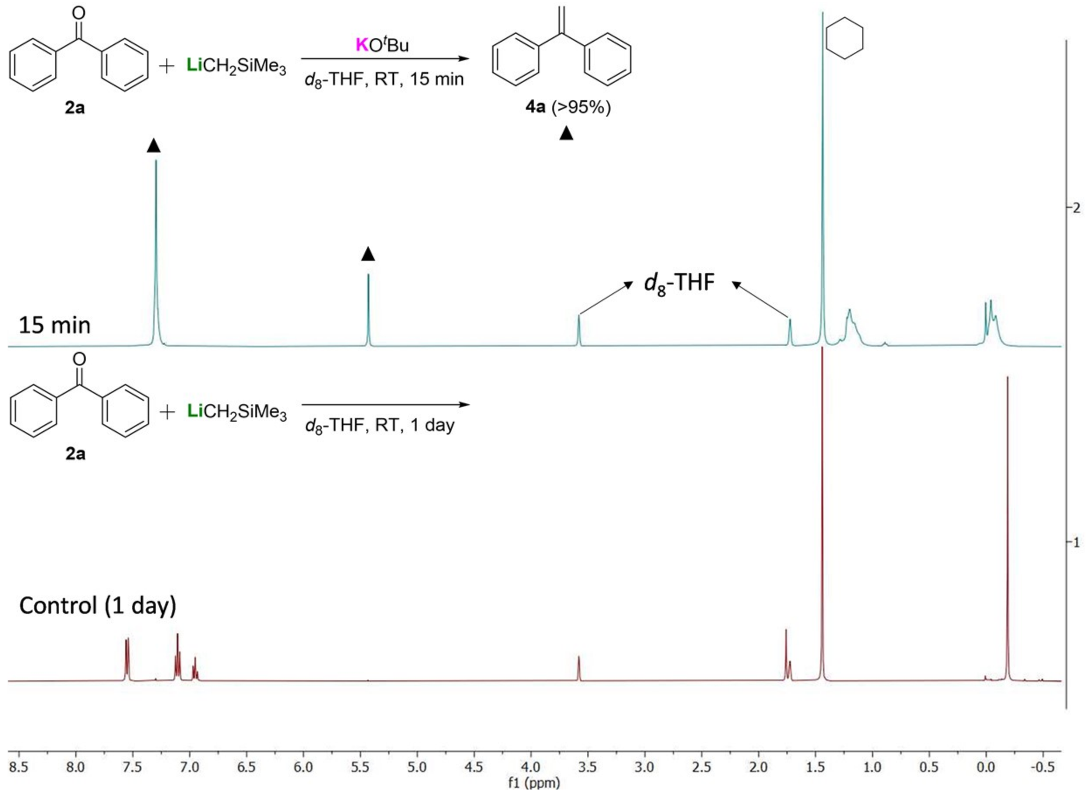

Figure S32: Stacked ^{1}H NMR (d_8-THF, 25 ˚C, 400 MHz) of an NMR scale transmetallation reaction between benzophenone (**2a**), $LiCH_2SiMe_3$ and KO^{t}Bu (top); the control reaction between benzophenone (**2a**) and $LiCH_2SiMe_3$ (bottom).

1.4 Scale-up Transmetallation

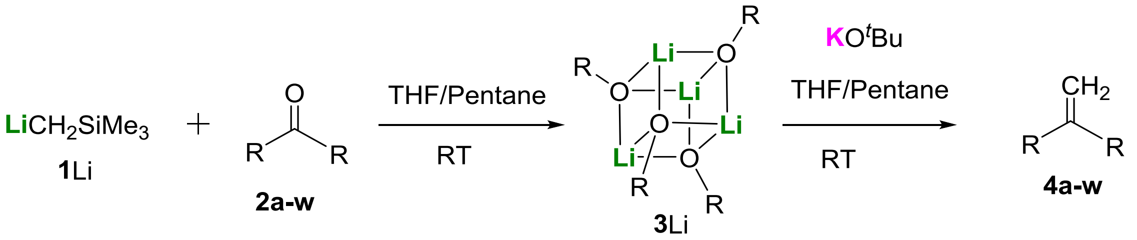

General procedure for scale-up reactions of organocarbonyls+ $LiCH_2SiMe_3$ + KO^{t}Bu in THF and pentane

An organocarbonyls (**2a-w**) (5.0 mmol, 1.0 equiv.) was dissolved in THF (15 ml) (extra dry over molecular sieve, stabilised, under N_2 (AcroSeal), Thermo Scientific) in a 100 mL Schlenk flask under N_2. The solution of $LiCH_2SiMe_3$ (Merck) (1.0 M in pentane, 7.5 mmol, 7.5 mL, 1.5 equiv.) was added dropwisely into the organocarbonyl solution at 0 ºC with stirring. The ice-water bath was removed after the addition. The mixture was stirred at room temperature for 1 – 2 h (the reaction time will be specified in the specific reaction as Step-A Nucleophilic Addition). Then the mixture was transferred dropwisely into the KO^{t}Bu (5.0 mmol, 0.5610 g, 1.0 equiv.) suspension in the extra dry THF (15 mL) at 0 ºC *via* a cannula. After removing the ice-water bath, the reaction was stirred at room temperature for 1.5 – 4.5 h (the reaction time will be specified in the specific reaction as Step-B Methylenation). All volatiles were removed under vacuum, the residue was quenched by deionised water (30 mL). The aqueous phase was extracted by diethyl ether, ethyl acetate or dichloromethane (30 mL × 4). The organic phase

was combined and washed with brine (30 mL) and dried over $MgSO_4$ for several hours to overnight. The organic phase was filtered by filter paper and the $MgSO_4$ was washed by the solvent used for extraction. All volatiles were removed *in vacuo* from the combined organic phase on rotary evaporator. The crude product was purified by column chromatography.

Transmetallation for ketones

Benzophenone (2a) to 1,1-Diphenylethylene (4a)
Reaction time:
Step-A Nucleophilic Addition: 1h 30min
Step-B Methylenation: 2h
4a was synthesized according to the general procedure. The crude product was extracted by diethyl ether and purified by column chromatography (hexanes) with isolated yield of 81% (0.7322 g, colourless liquid).

^{1}H NMR (400 MHz, $CDCl_3$, 25 °C): δ(ppm) 7.41-7.35 (m, 10H, Ar*H*), 5.51 (s, 2H, =CH_2).

NMR data are consistent with the literature.[6]

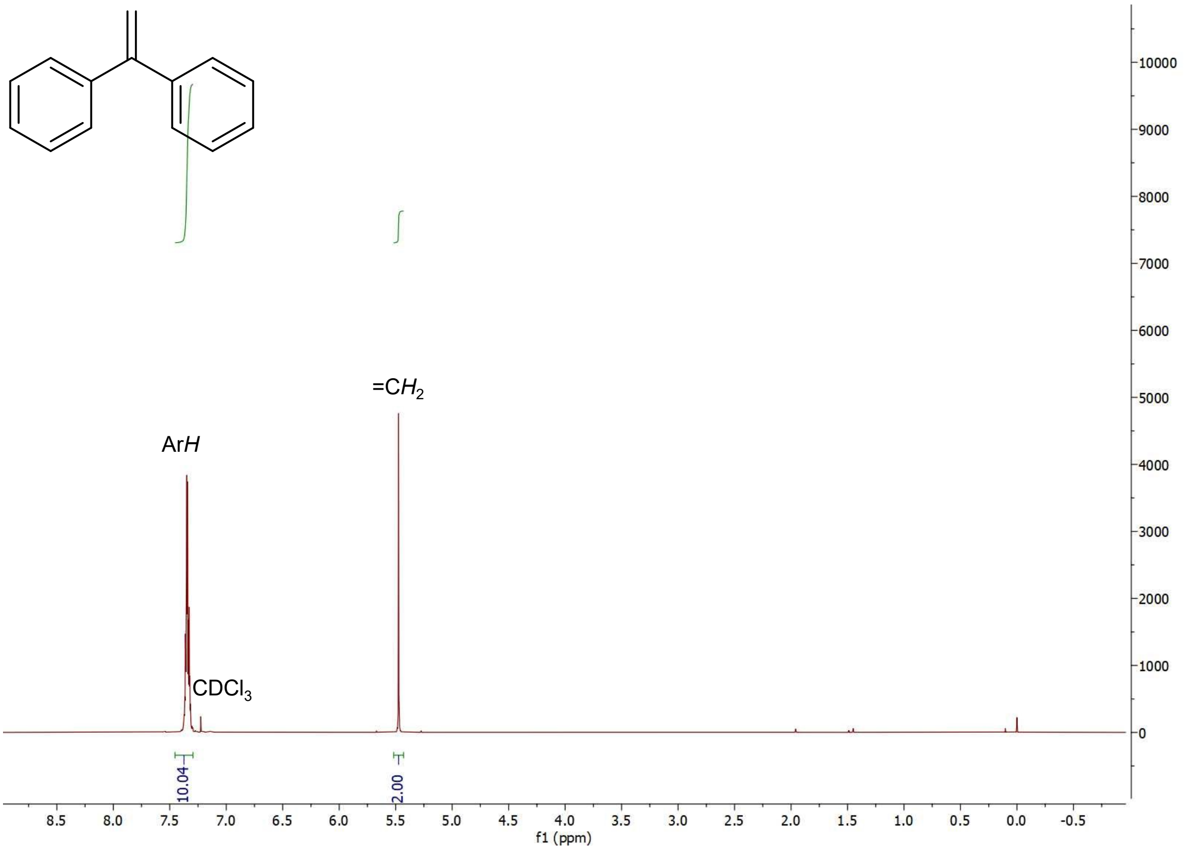

Figure S33: ^{1}H NMR ($CDCl_3$, 25 ˚C, 400 MHz) of 1,1-diphenylethylene (**4a**).

Bis(3,5-bis(trifluromethyl)phenyl) ketone (2b) to 1,1-bis(3,5-bis(trifluromethyl)phenyl)ethylene (4b)
Reaction time:
Step-A Nucleophilic Addition: 1h

Step-B Methylenation: 1h 30min

4b was synthesized according to the general procedure. The crude product was extracted by diethyl ether and purified by column chromatography (hexanes) with isolated yield of 64% (1.4321 g, white solid).

^{1}H NMR (400 MHz, $CDCl_3$, 25 °C): δ (ppm) 7.91 (s, 2H, Ar*H*), 7.74 (s, 4H, Ar*H*) 5.79 (s, 2H, =CH_2).

^{19}F NMR (377 MHz, $CDCl_3$, 25 °C): δ (ppm) -63.0 (s, -CF_3)

NMR data are consistent with the literature.[5]

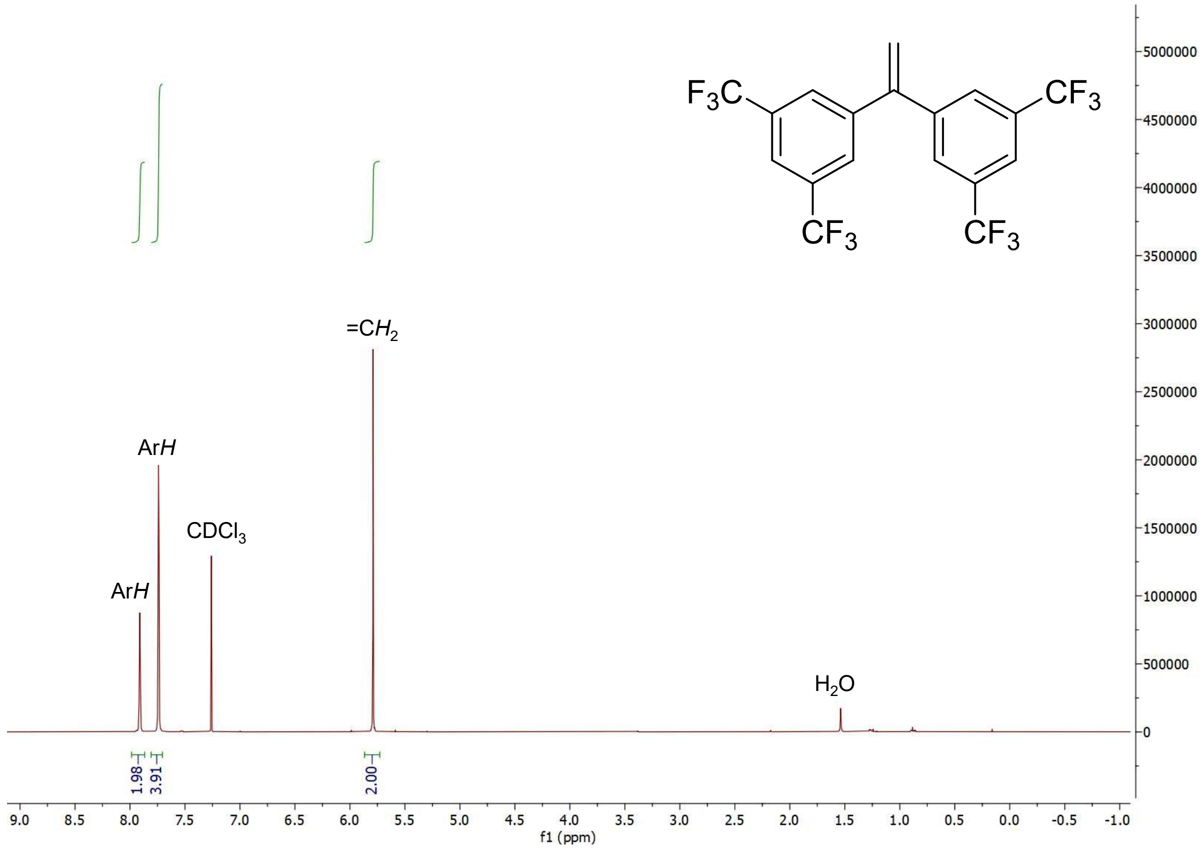

Figure S34: ^{1}H NMR ($CDCl_3$, 25 ˚C, 400 MHz) of 1,1-bis(3,5-bis(trifluromethyl)phenyl)ethylene (**4b**).

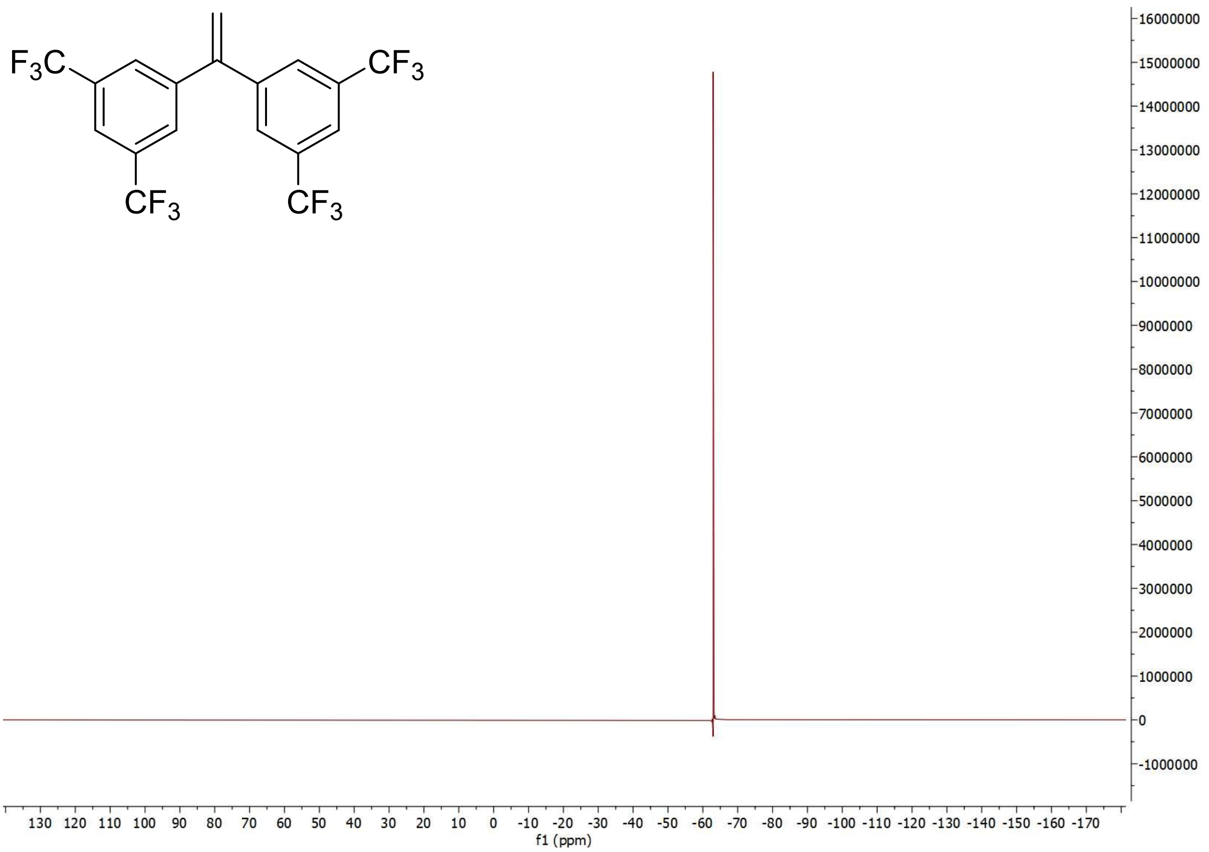

Figure S35: ^{19}F NMR ($CDCl_3$, 25 ˚C, 377 MHz) of 1,1-bis(3,5-bis(trifluromethyl)phenyl)ethylene (**4b**).

Bis(*p*-(dimethylamino)phenyl) ketone (2c) to 1,1-bis(*p*-(dimethylamino)phenyl)ethylene (4c)

Reaction time:

Step-A Nucleophilic Addition: 1h 20min

Step-B Methylenation: 3h

4c was synthesized according to the general procedure. The crude product was extracted by diethyl ether and purified by column chromatography (hexanes : ethyl acetate = 10:1) with isolated yield of 72 % (0.9596 g, white solid).

^1H NMR (400 MHz, $CDCl_3$, 25 °C): δ (ppm) 7.28 (d, $^3J_{HH}$ = 8.7 Hz, 4H, Ar*H*), 6.71(d, $^3J_{HH}$ = 8.3 Hz, 4H, Ar*H*), 5.20 (s, 2H, =CH_2), 2.98 (s, 12H, -N(CH_3)$_2$).

NMR data are consistent with the literature.[7]

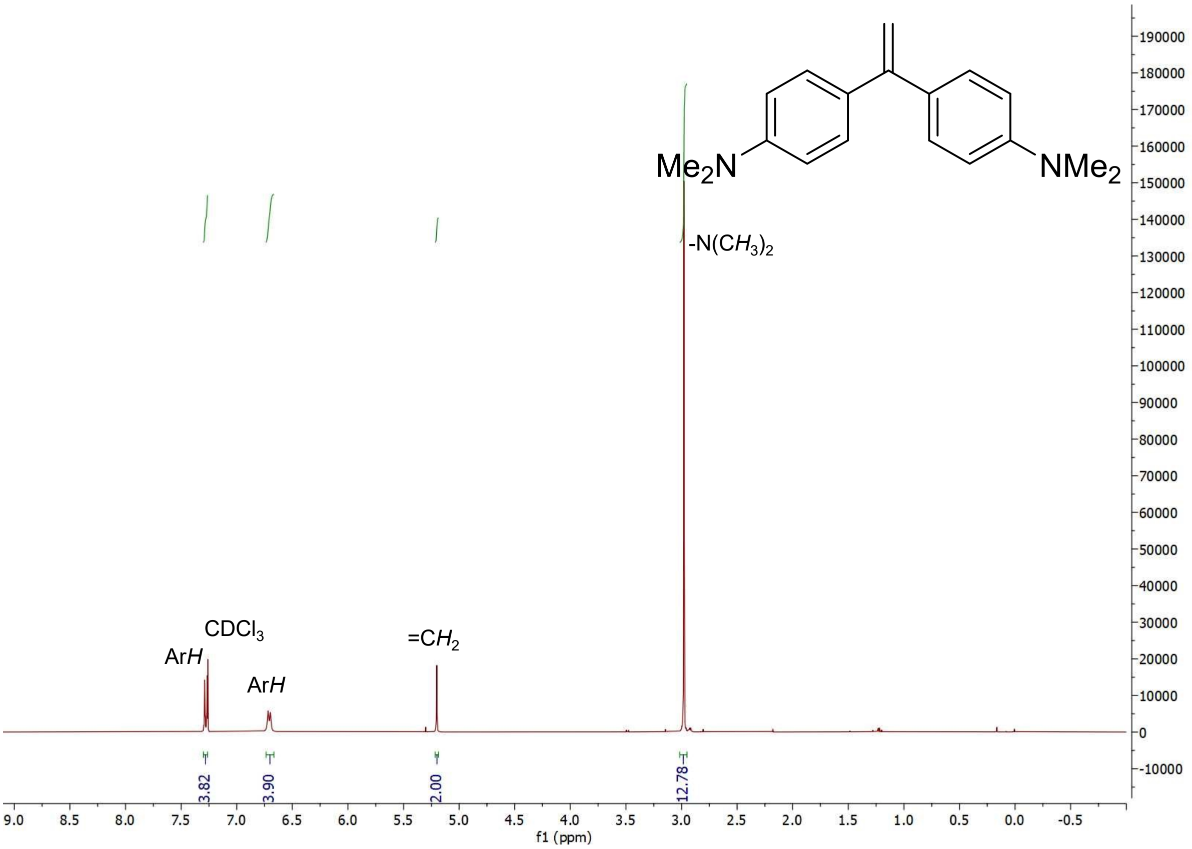

Figure S36: ^{1}H NMR ($CDCl_3$, 25 ˚C, 400 MHz) of 1,1-bis(*p*-(dimethylamino)phenyl)ethylene (**4c**).

Bis(4-methoxyphenyl) ketone (2d) to 1,1-bis(4-methoxyphenyl)ethylene (4d)
Reaction time:
Step-A Nucleophilic Addition: 1h 10min
Step-B Methylenation: 3h 50min

4d was synthesized according to the general procedure. The crude product was extracted by dichloromethane, removal of all volatile under vacuum afforded **4d** as a white solid in 96% yield (1.1575 g). Column chromatography was not needed in this case.

^{1}H NMR (400 MHz, $CDCl_3$, 25 °C): δ (ppm) 7.31-7.27 (m, 4H, Ar*H*), 6.89-6.86 (m, 4H, Ar*H*), 5.31 (s, 2H, =CH_2), 3.84(s, 6H, -CH_3).

NMR data are consistent with the literature.[8]

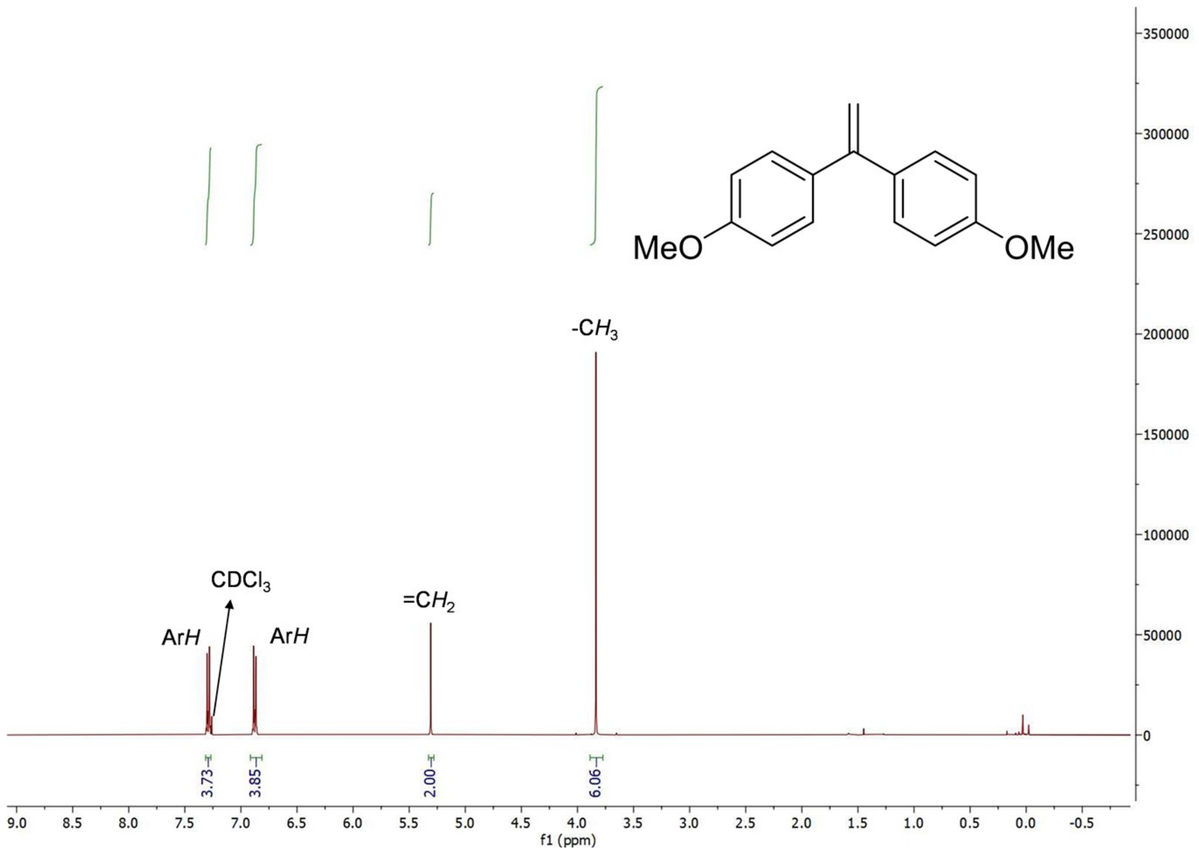

Figure S37: ^{1}H NMR ($CDCl_3$, 25 ˚C, 400 MHz) of 1,1-bis(4-methoxyphenyl)ethylene (**4d**).

Phenyl *tert*-butyl ketone (2e) to 1-phenyl-1-*tert*-butyl ethylene (4e)
Reaction time:
Step-A Nucleophilic Addition: 1h
Step-B Methylenation: 4h 30min

4e was synthesized according to the general procedure. The crude product was extracted by diethyl ether and purified by column chromatography (hexanes) with isolated yield of 53 % (0.4374 g, pale yellow oil).

^{1}H NMR (300 MHz, $CDCl_3$, 25 °C): δ (ppm) 7.30-7.22 (m, 3H, Ar*H*), 7.16-7.12(m, 2H, Ar*H*), 5.17 (d, $^{2}J_{HH}$ = 1.7 Hz, 1H, =CH_2), 4.76 (d, $^{2}J_{HH}$ = 1.7 Hz, 1H, =CH_2), 1.12 (s, 9H, -CH_3).

NMR data are consistent with the literature.[9]

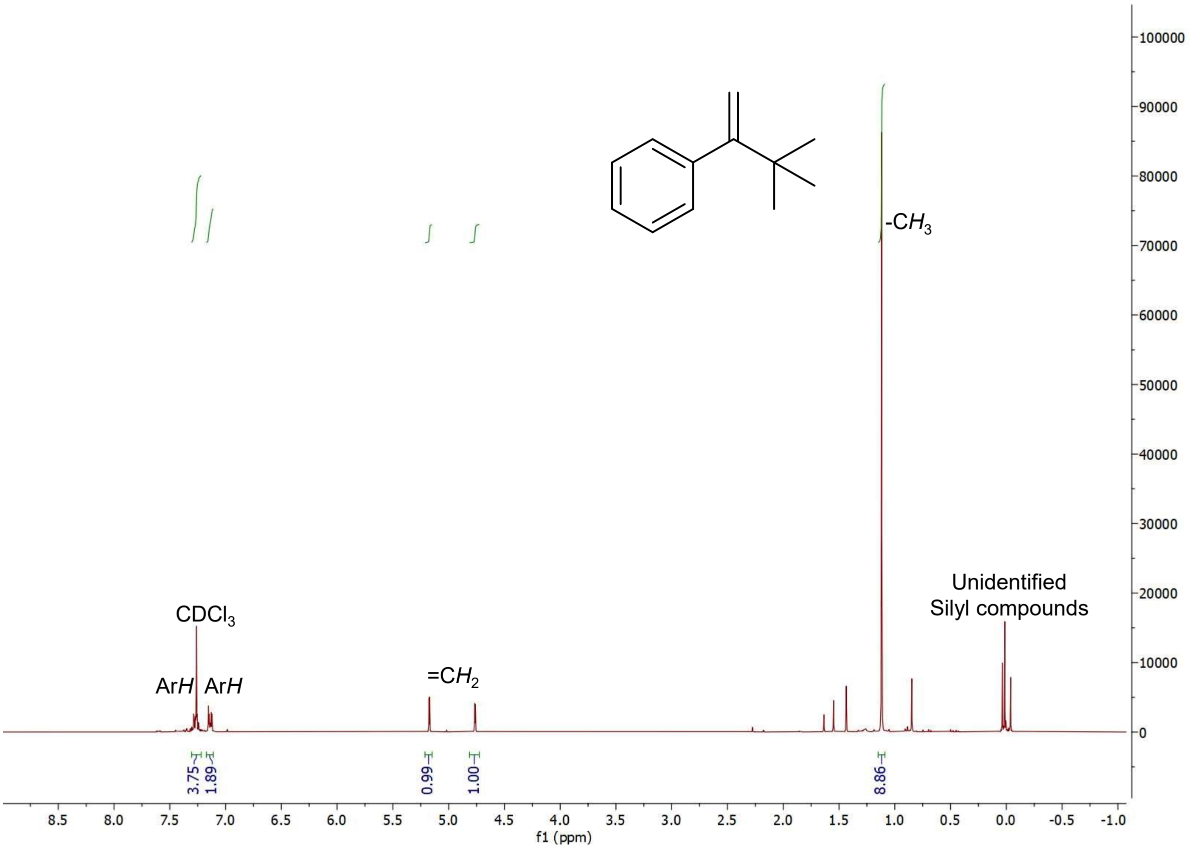

Figure S38: ^{1}H NMR ($CDCl_3$, 25 ˚C, 300 MHz) of 1,1'-phenyl *tert*-butyl ethylene (**4e**).

Acetophenone (2f) to isopropenylbenzene (4f)
Reaction time:
Step-A Nucleophilic Addition: 1h
Step-B Methylenation: 3 days

4f was synthesized according to the general procedure but the reaction was last for 3 days. The crude product was extracted by diethyl ether and purified by column chromatography (hexanes : ethyl acetate = 1:0 to 20:1) with isolated yield of 52 % (0.3069 g, colourless oil).

^{1}H NMR (400 MHz, $CDCl_3$, 25 °C): δ (ppm) 7.51-7.46 (m, 2H, Ar*H*), 7.38-7.32 (m, 2H, Ar*H*), 7.31-7.25 (m, 1H, Ar*H*), 5.39 (m, 1H, =CH_2), 5.10 (m, 1H, =CH_2), 2.17 (dd, $^4J_{HH}$ (*trans*) = 1.5 Hz, $^4J_{HH}$ (*cis*) = 0.8 Hz, 1H, -CH_3).

NMR data are consistent with the literature.[10]

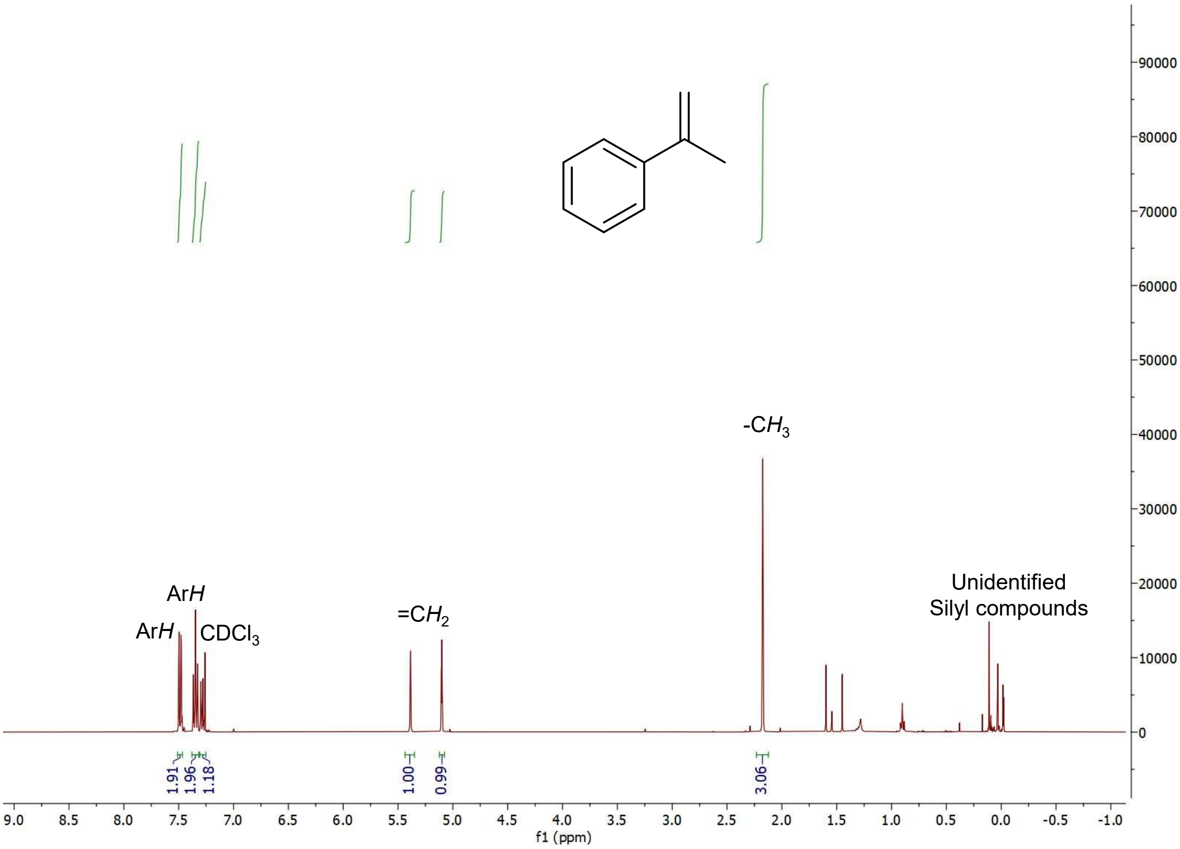

Figure S39: ^{1}H NMR ($CDCl_3$, 25 ˚C, 400 MHz) of isopropenylbenzene (**4f**).

Phenyl-cyclohexyl ketone (2g) to 1-phenyl-1-cyclohexylethylene (4g)
Reaction time:
Step-A Nucleophilic Addition: 1h
Step-B Methylenation: 1h 45min

4g was synthesized according to the general procedure. The crude product was extracted by diethyl ether and purified by column chromatography (hexanes : ethyl acetate = 1:0 to 20:1) with isolated yield of 83% (0.7675 g, colourless liquid).

^{1}H NMR (400 MHz, $CDCl_3$, 25 °C): δ (ppm) 7.37-7.29 (m, 4H, Ar*H*), 7.23-7.29 (m, 1H, Ar*H*) 5.14 (d, $^2J_{HH}$ = 1.2 Hz, 1H, =CH_2), 5.01 (t, $^2J_{HH}$ = 1.2 Hz, 1H, =CH_2), 2.43 (t, $^3J_{HH}$ = 11.5 Hz, 1H, -CH_2C*H*(C(Ph)CH_2)CH_2-), 1.90-1.75 (m, 4H, -CH_2-), 1.75-1.67(m, 1H, -CH_2-), 1.41-1.11(m, 5H, -CH_2-).

NMR data are consistent with the literature.[11]

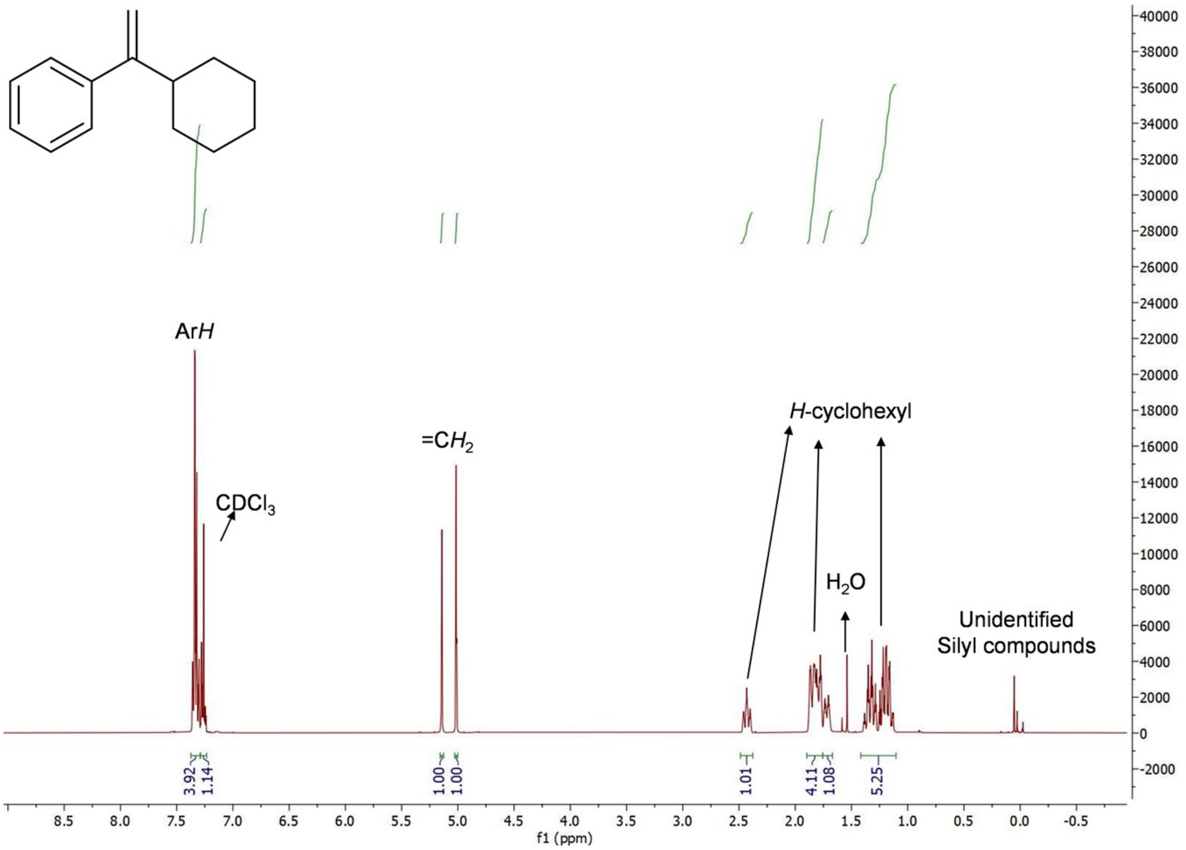

Figure S40: ^{1}H NMR (CDCl$_3$, 25 ˚C, 400 MHz) of 1-phenyl-1-cyclohexylethylene (**4g**).

Dicyclohexyl ketone (1h) to ethene-1,1-diyldicyclohexane (4h)
Reaction time:
Step-A Nucleophilic Addition: 1h 10min
Step-B Methylenation: 2h 45min

4h was synthesized according to the general procedure. The crude product was extracted by diethyl ether and purified by column chromatography (hexanes) with isolated yield of 75% (0.7203 g, colourless liquid).

^{1}H NMR (400 MHz, CDCl$_3$, 25 °C): δ(ppm) 4.69 (s, 2H, =C*H*$_2$), 1.89-1.64 (m, 12H, *H*-cyclohexyl), 1.34-1.08 (m, 10H, *H*-cyclohexyl).

NMR data are consistent with the literature.[12]

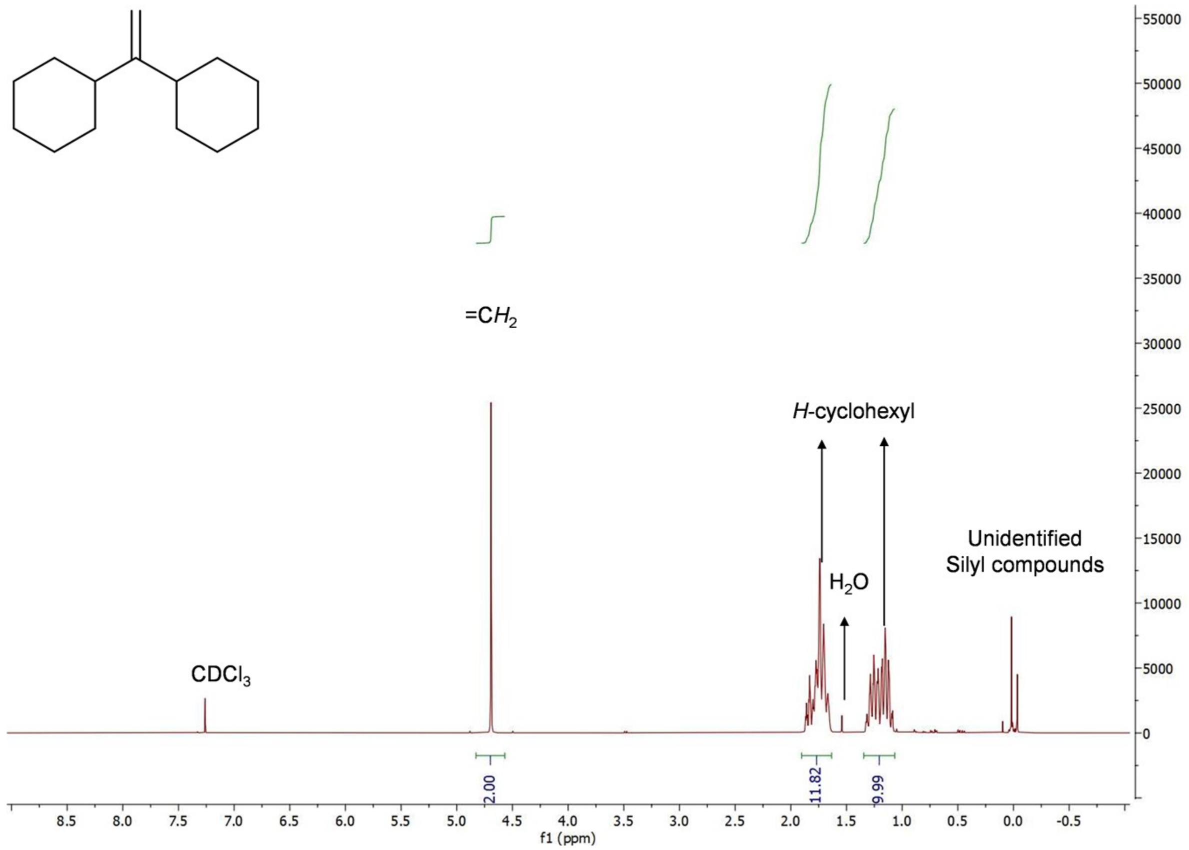

Figure S41: ^{1}H NMR ($CDCl_3$, 25 ˚C, 400 MHz) of ethene-1,1-diyldicyclohexane (**4h**).

***L*-Menthone (2i) to *(1S,4R)*-1-Isopropyl-4-methyl-2-methylenecyclohexane (4i)**
Reaction time:
Step-A Nucleophilic Addition: 1h 30min
Step-B Methylenation: 3h 20min

4i was synthesized according to general procedure. The crude product was extracted by diethyl ether and purified by column chromatography (hexanes) with isolated yield of 43.8 % (0.3335 g, colourless oil).

^{1}H NMR (300 MHz, $CDCl_3$, 25 °C): δ (ppm) 4.70 (m, 1H, =CH_2), 4.58 (m, 1H, =CH_2), 2.33-2.23 (m, 1H), 2.05-1.88 (m, 1H), 1.83-1.51 (m, 5H), 1.30-1.04 (m, overlap with impurities, 2H), 0.95-0.86 (m, 9H).

NMR data are consistent with the literature.[13]

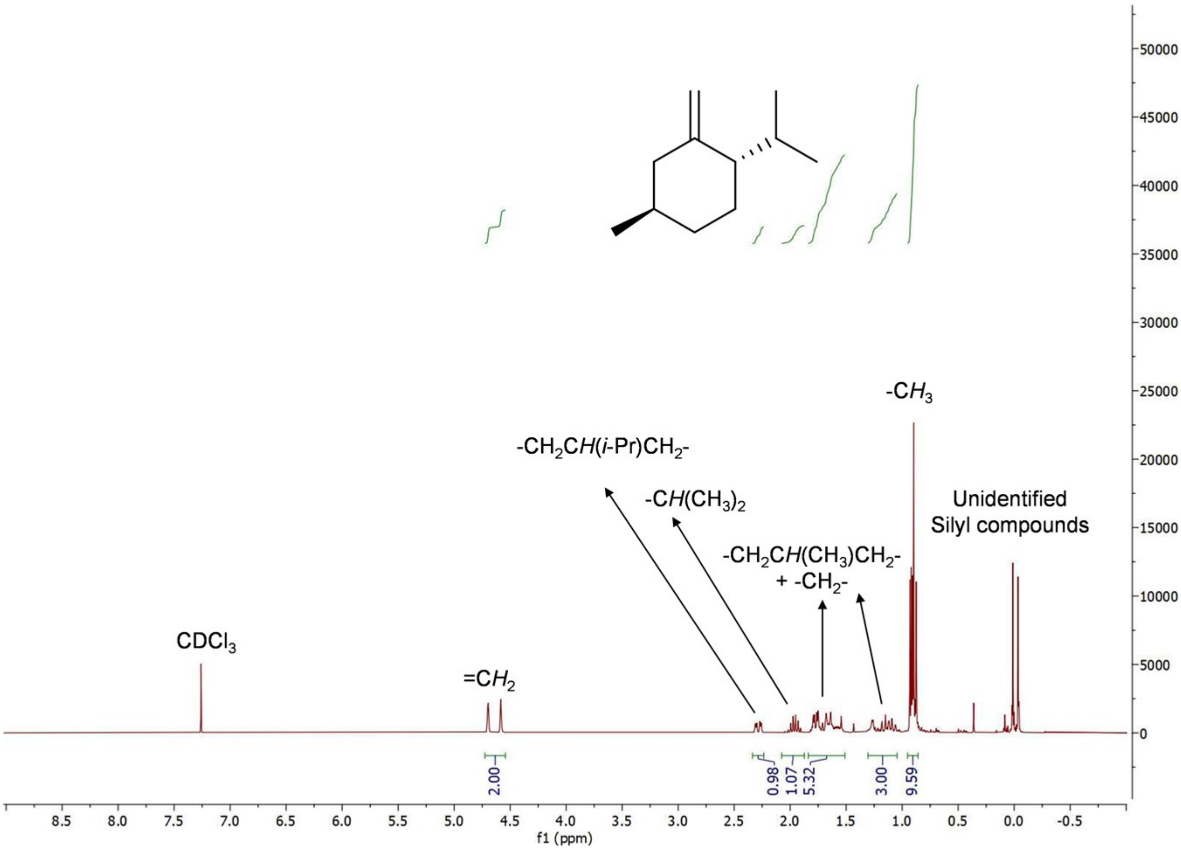

Figure S42: ^{1}H NMR ($CDCl_3$, 25 ˚C, 300 MHz) of *(1S,4R)*-1-Isopropyl-4-methyl-2-methylenecyclohexane (**4i**).

2-Adamantanone(2j) to 2-methylideneadamantane (4j)
Reaction time:
Step-A Nucleophilic Addition: 1h 10min
Step-B Methylenation: 2h 20min

4j was synthesized according to general procedure. The crude product was extracted by ethyl acetate and purified by column chromatography (diluent: hexanes) with isolated yield of 59 % (0.4361, white solid).

^{1}H NMR (300 MHz, $CDCl_3$, 25 °C): δ (ppm) 4.50 (s, 2H, =C*H*$_2$), 2.48 (br, 2H, -C*H*(CH_2-)-), 1.98-1.70 (m, 12H, -C*H*(CH_2-)-, -CH_2-).

NMR data are consistent with the literature.[14]

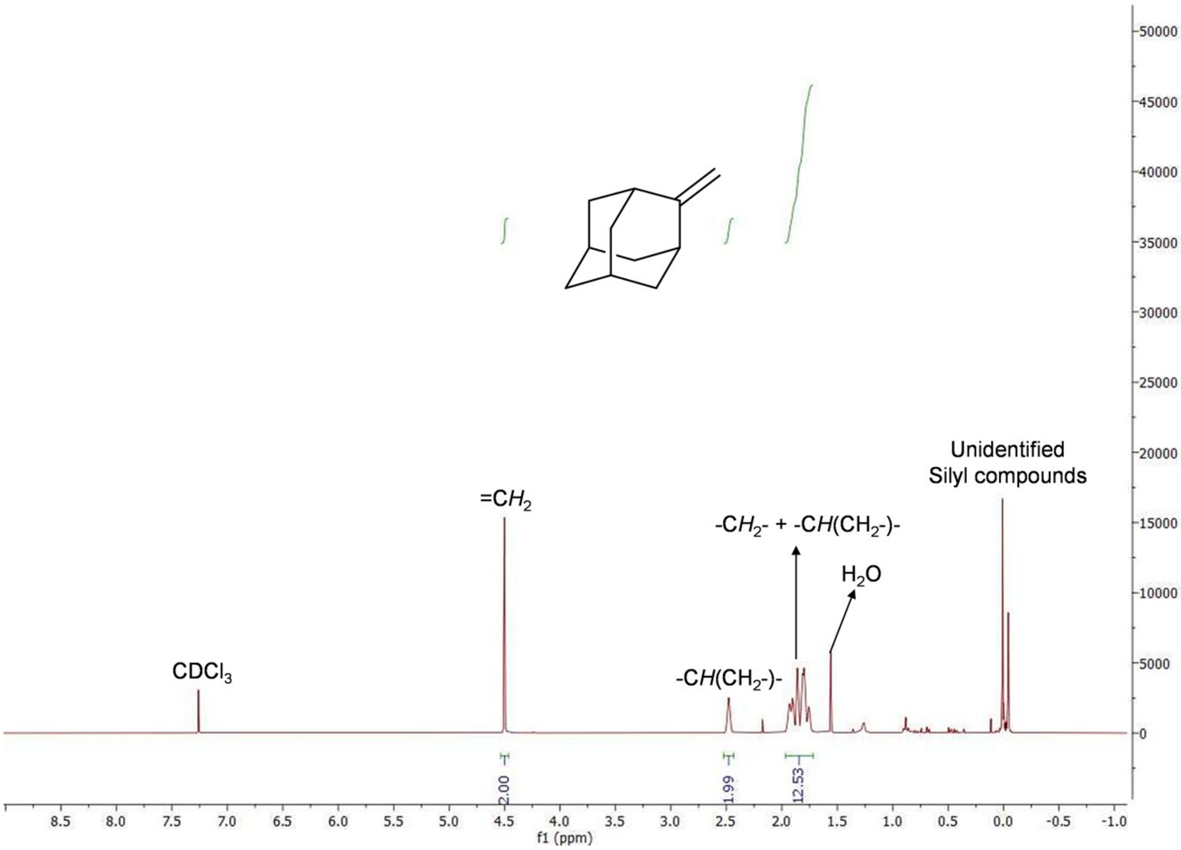

Figure S43: ^{1}H NMR ($CDCl_3$, 25 ˚C, 300 MHz) of 2-methylideneadamantane **(4j)**.

1,4-Phenylenebis(phenylmethanone) (2k) to 1,4-bis(1-phenylvinyl)benzene (4k)
Reaction time:
Step-A Nucleophilic Addition: 1h 15min
Step-B Methylenation: 2h 20min

4k was synthesized according to the general procedure, but with two modifications: (1) 3.0 equiv. of **1**Li and 2.0 equiv. of KO^{t}Bu were used; (2) KO^{t}Bu-THF suspension was added into the suspension of the nucleophilic addition mixture. The reasons for these modifications are: (1) the extra **1**Li and KO^{t}Bu are needed to convert both carbonyls; (2) after the nucleophilic addition, this reaction forms a suspension, in comparison to homogenous solutions in other cases.
The crude product was extracted by ethyl acetate. After removing all volatiles, the crude product was purified by washing with hexanes (4 mL × 3) with isolated yield of 76 % (1.0778 g, pale yellow-to-white solids). No column chromatography was needed for this reaction.

^{1}H NMR (300 MHz, $CDCl_3$, 25 °C): δ (ppm) 7.44-7.31 (m, 14H, Ar*H*), 5.53 (d, $^2J_{HH}$ = 1.2 Hz, 2H, =CH_2), 5.49 (d, $^2J_{HH}$ = 1.2 Hz, 2H, =CH_2).

NMR data are consistent with the literature.[15]

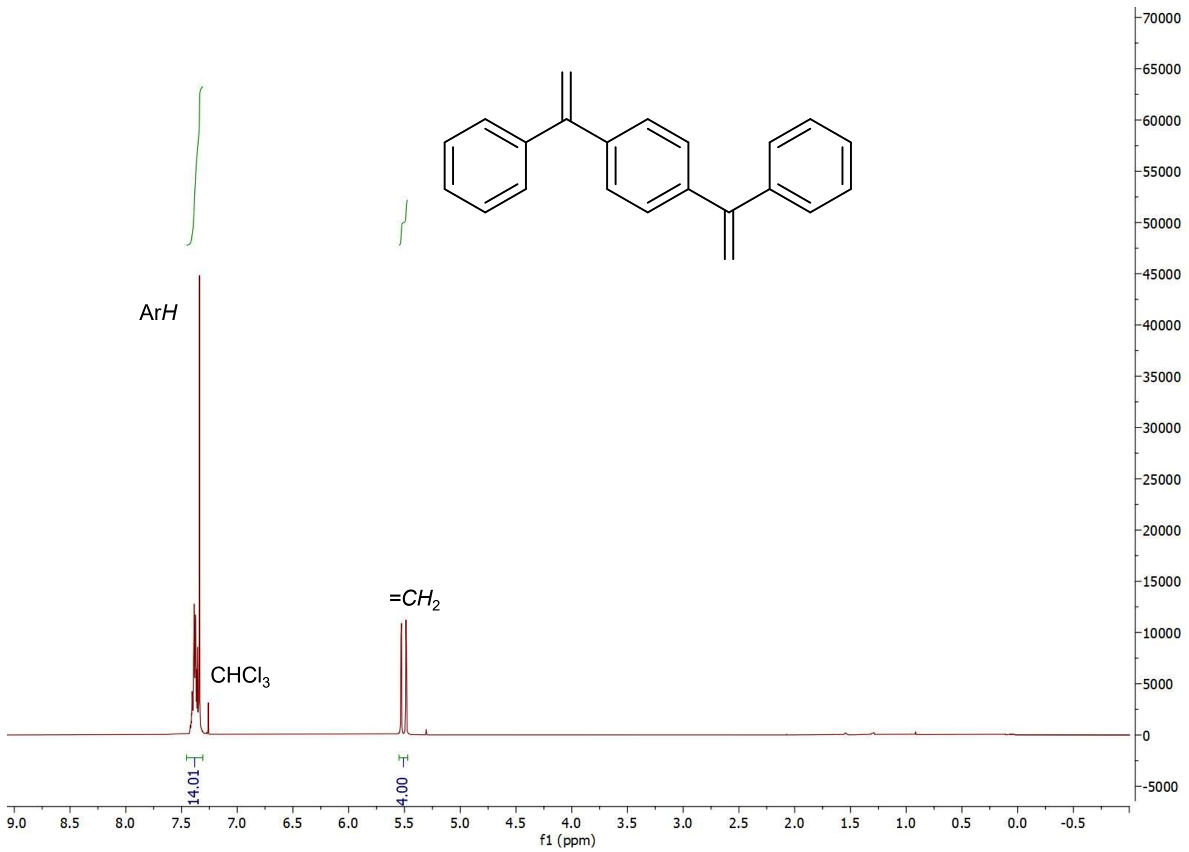

Figure S44: ^{1}H NMR ($CDCl_3$, 25 ˚C, 300 MHz) of 1,4-bis(1-phenylvinyl)benzene (**4k**).

Attempts to mono-methylenate 2k:

We have attempted twice to mono-methylenate **2k**, by controlling the stoichiometric ration between **2k** and **1**Li, as well as temperature. But only a small amount of the mono-methylenated compound[16] (yellow square) was found in the crude product. The rest components were **2k** starting material (red frame) and **4k** (blue sphere).

Attempt 1: we followed the general procedure except changing the ratio of **1**Li to 1.2 equivalents.

Reaction time:

Step-A Nucleophilic Addition: 1h

Step-B Methylenation: 3h

Attempt 2: besides changing the ratio of **1**Li to 1.05 equivalent, we kept the first step reaction at −78 ± 4 °C for 1 h.

Reaction time:

Step-A Nucleophilic Addition: (i) −78 °C for 1h; (ii) RT for 15min

Step-B Methylenation: 3h

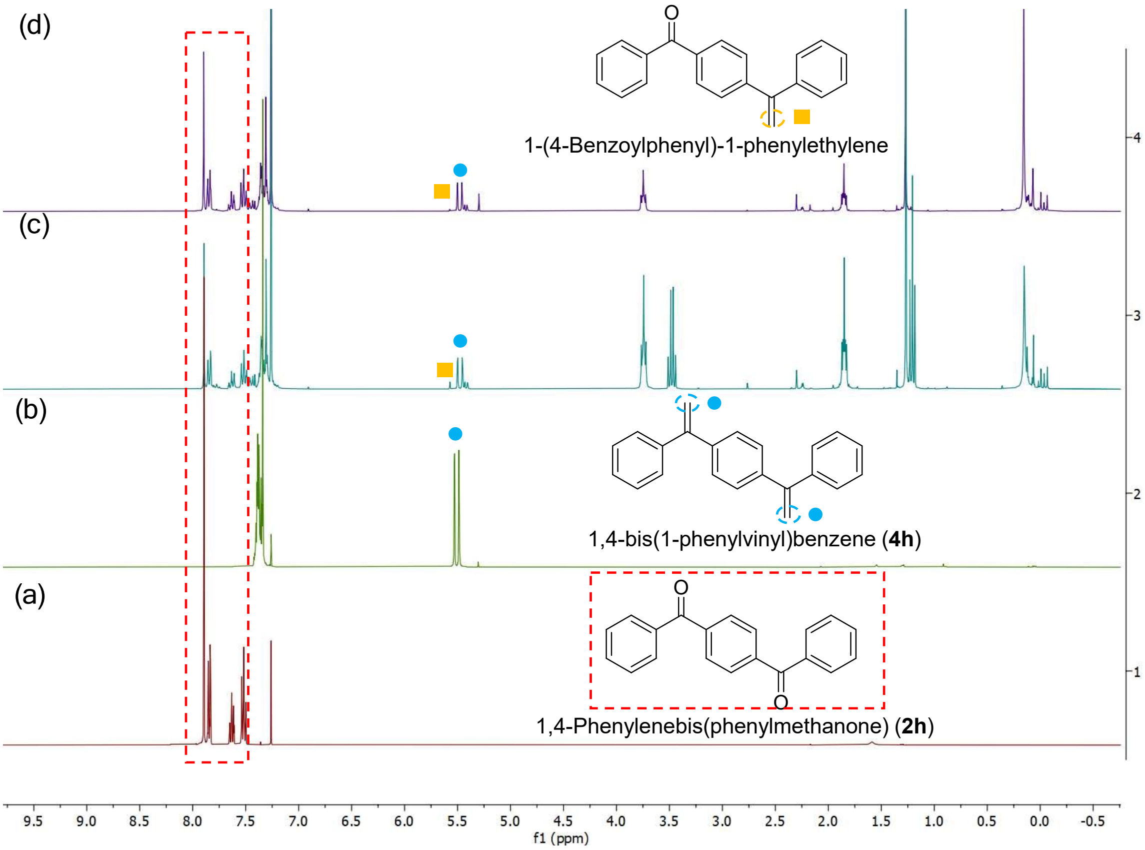

Figure S45: ^{1}H NMR ($CDCl_3$, 25 ˚C, 300 MHz) of (a) **2k**, (b) **4k**, (c) crude products before extraction in Attempt 1 and (d) crude products before extraction in Attempt 2. (red frame: all proton peaks of **2k**; blue sphere: characteristic =CH_2 peaks of **4k**; yellow square: characteristic =CH_2 peaks of 1-(4-Benzoylphenyl)-1-phenylethylene).

Transmetallation for Aldehydes

9-Anthracene aldehyde (2l) to 9-vinylanthracene (4l)
Reaction time:
Step-A Nucleophilic Addition: 1h 30min
Step-B Methylenation: 2h 30min

4l was synthesized according to the general procedure. The crude product was extracted by diethyl ether and purified by column charmatography (hexanes : ethyl acetate = 1:0, then 20:1, finally 10:1) with isolated yield of 74 % (0.7586 g, yellow crystals).

^{1}H NMR (400 MHz, $CDCl_3$, 25 °C): δ (ppm) 8.41 (s, 1H, 9-H anthracenyl), 8.38-8.33 (m, 2H, Ar*H*), 8.05-7.99 (m, 2H, Ar*H*), 7.56-7.46 (m, 5H, Ar*H* and -C*H*=), 6.04 (dd, $^3J_{HH}$ (*cis*) = 11.5, $^2J_{HH}$ = 2.1 Hz, 1H, =CH_2), 5.66 (dd, $^3J_{HH}$ (trans) = 17.9, $^2J_{HH}$ = 2.1 Hz, 1H, =CH_2).

NMR data are consistent with the literature.[17]

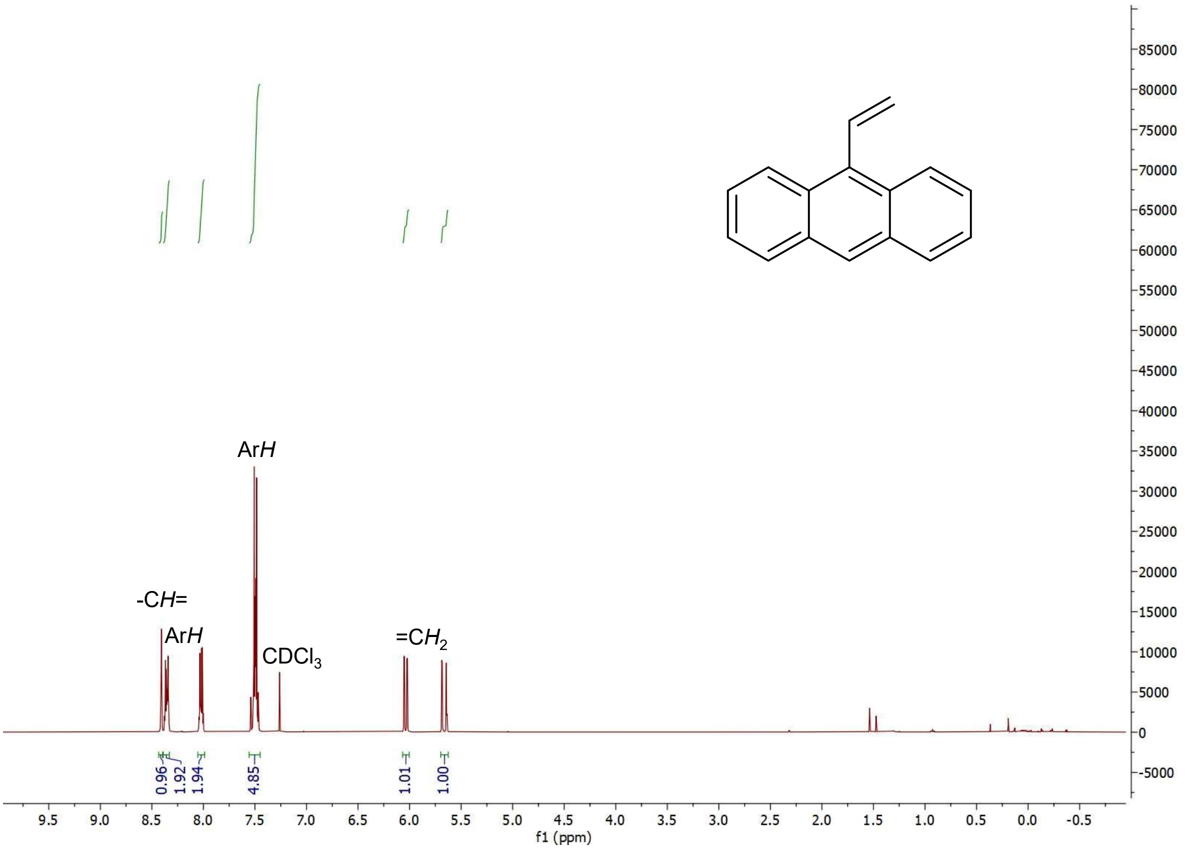

Figure S46: ^{1}H NMR ($CDCl_3$, 25 ˚C, 400 MHz) of 9-vinylanthracene (**4l**).

4-Triflurometyl-benaylaldehyde (2m) to 1-trifluoromethyl-4-vinyl-benzene (4m)
Reaction time:
Step-A Nucleophilic Addition: 1h 30min
Step-B Methylenation: 3h

4m was synthesized according to the general procedure. The crude product was extracted by diethyl ether and purified by column chromatography (diluent: hexanes) with isolated yield of 62 % (0.5309 g, colourless oil).

^{1}H NMR (400 MHz, $CDCl_3$, 25 °C): δ (ppm) 7.56 (d, $^{3}J_{HH}$ = 8.2 Hz, 2H, Ar*H*), 7.48 (d, $^{3}J_{HH}$ = 8.2 Hz, 2H, Ar*H*), 6.73 (dd, $^{3}J_{HH}$ (trans) = 17.6 Hz, $^{3}J_{HH}$ (cis) = 10.9 Hz, 1H, -C*H*=), 5.83 (d, $^{3}J_{HH}$ (trans) = 17.6 Hz, 1H, =CH_2), 5.37 (d, $^{3}J_{HH}$ (cis) = 10.9 Hz, 1H, =CH_2).
^{19}F NMR (377 MHz, $CDCl_3$, 25 °C): δ (ppm) -62.5 (s, -CF_3).

NMR data are consistent with the literature.[17]

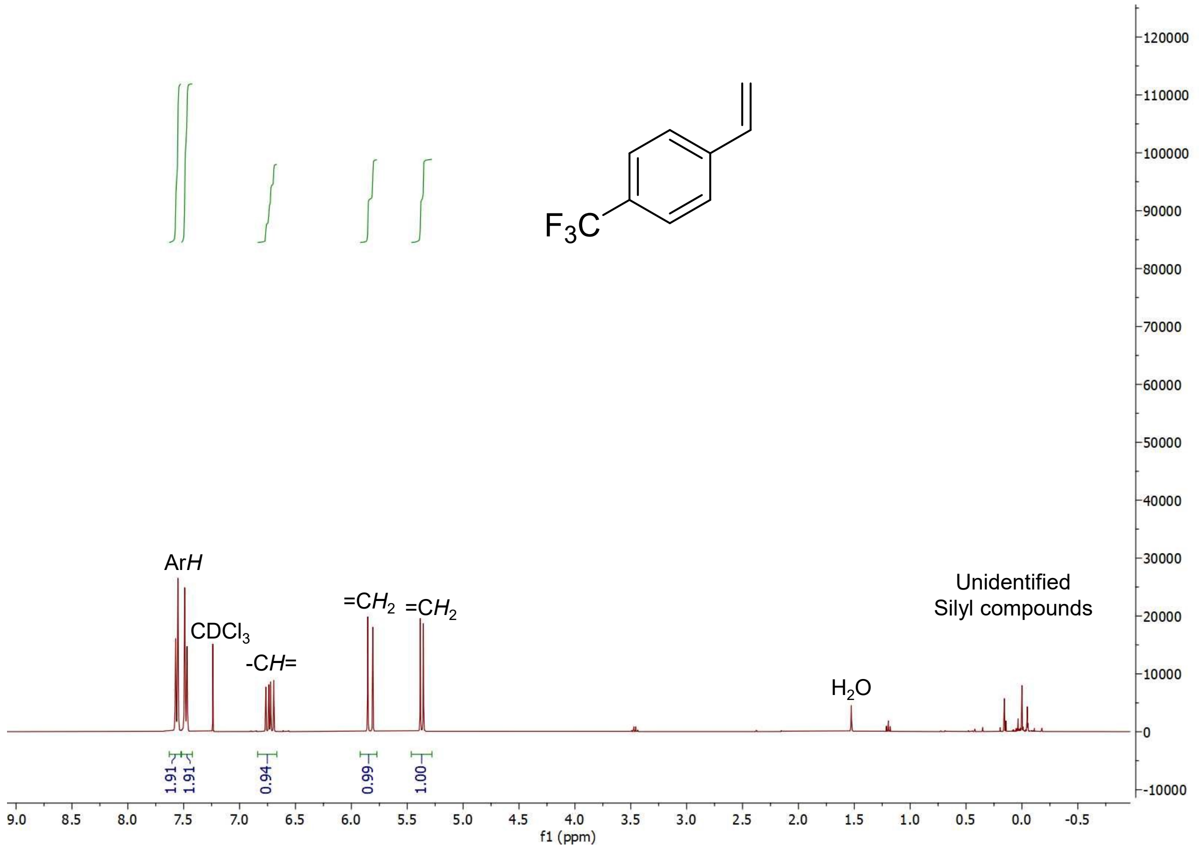

Figure S47: ^{1}H NMR ($CDCl_3$, 25 ˚C, 400 MHz) of 1-trifluoromethyl-4-vinyl-benzene (**4m**).

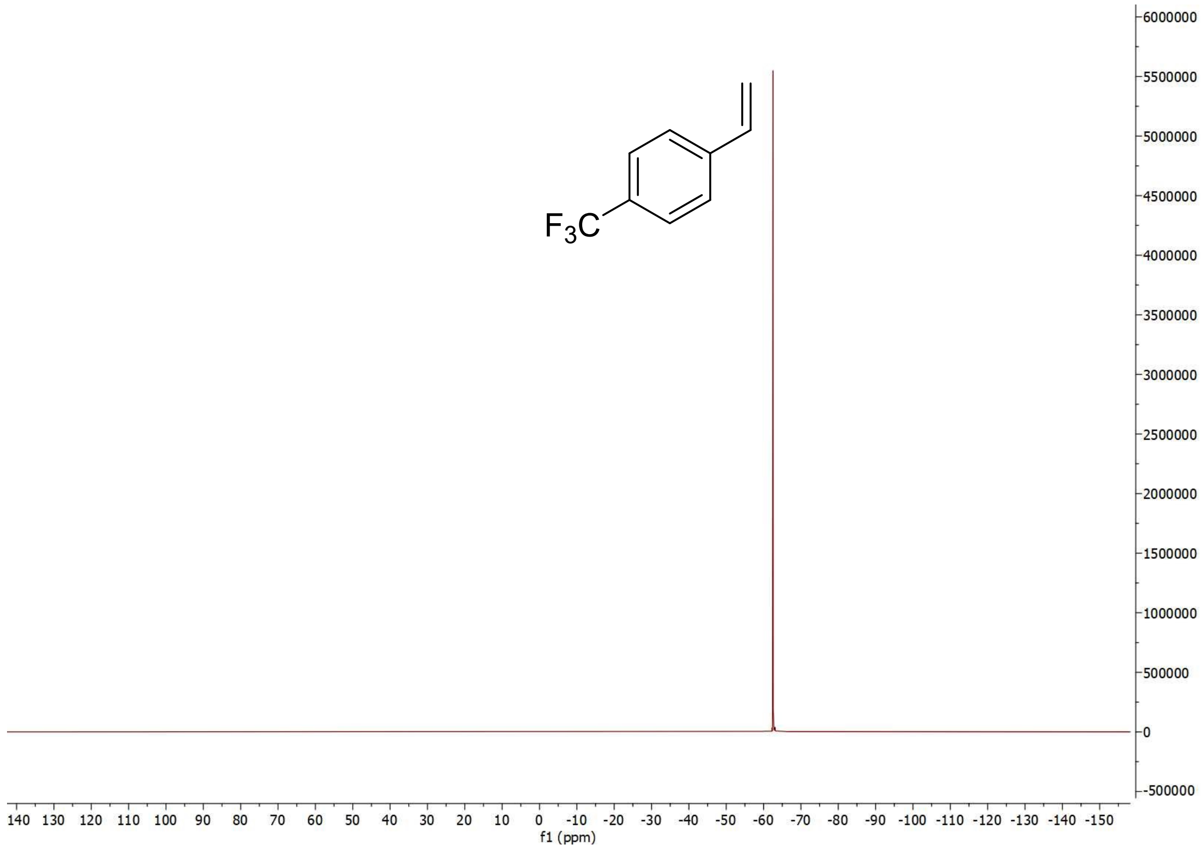

Figure S48: ^{19}F NMR ($CDCl_3$, 25 ˚C, 377 MHz) of 1-trifluoromethyl-4-vinyl-benzene (**4m**).

Benzaldehyde (2n) to styrene (4n)
Reaction time:
Step-A Nucleophilic Addition: 1h
Step-B Methylenation: 4h 15min

4n was synthesized according to the general procedure. The crude product was extracted by diethyl ether and purified by column chromatography (hexanes) with isolated yield of 54 % (0.2831 g, colourless liquid).

^{1}H NMR (400 MHz, $CDCl_3$, 25 °C): δ (ppm) 7.44-7.40 (m, 2H, Ar*H*), 7.36-7.31 (m, 2H, Ar*H*), 7.28-7.24 (m, 1H, Ar*H*), 6.73 (dd, $^3J_{HH}$ (trans) = 17.6 Hz, $^3J_{HH}$ (cis) = 10.9 Hz, 1H, -C*H*=), 5.76 (dd, $^3J_{HH}$ (trans) = 17.6 Hz, $^2J_{HH}$ = 0.9 Hz, 1H, =CH_2), 5.25 (dd, $^3J_{HH}$ (cis) =10.9 Hz, $^2J_{HH}$ = 0.9 Hz, 1H, =CH_2).

NMR data are consistent with the literature.[18]

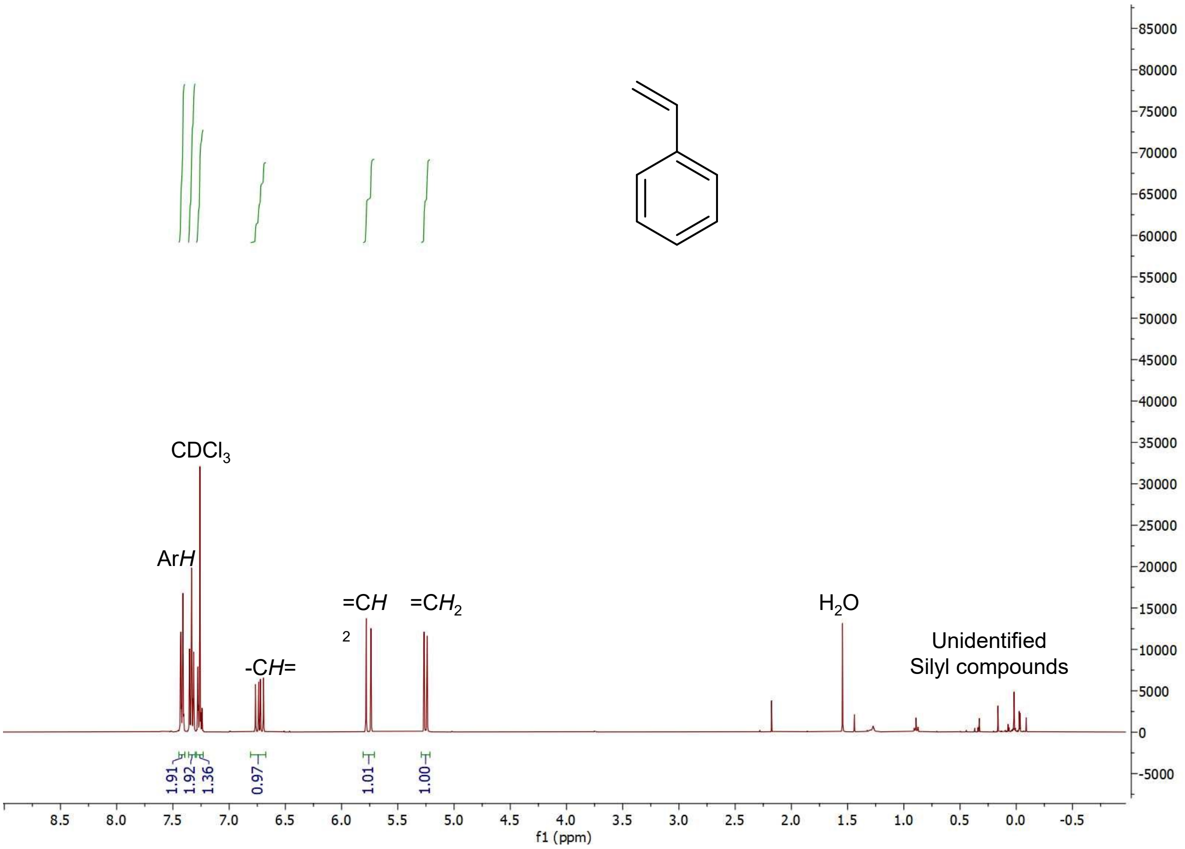

Figure S49: ^{1}H NMR ($CDCl_3$, 25 ˚C, 400 MHz) of Styrene (**4n**).

4-Methoxybenzaldehyde (2o) to 4-methoxystyrene (4o)
Reaction time:
Step-A Nucleophilic Addition: 1h 15min
Step-B Methylenation: 43h

4o was synthesized according to the general procedure. The crude product was extracted by diethyl ether and purified by column chromatography (hexanes) with isolated yield of 79 % (0.5319 g, colourless oil).

^{1}H NMR (300 MHz, CDCl$_3$, 25 °C): δ (ppm) 7.40-7.31 (m, 2H, Ar*H*), 6.92-6.82 (m, 2H, Ar*H*), 6.67 (dd, $^3J_{HH}$ (trans) = 17.6 Hz, $^2J_{HH}$ (cis) = 10.9 Hz, 1H, -C*H*=), 5.62 (d, $^3J_{HH}$ (trans) = 17.6 Hz, 1H, =CH_2), 5.13 (d, $^3J_{HH}$ (cis) = 10.9 Hz, 1H, =CH_2), 3.82 (s, 3H, -CH_3).

NMR data are consistent with the literature.[19]

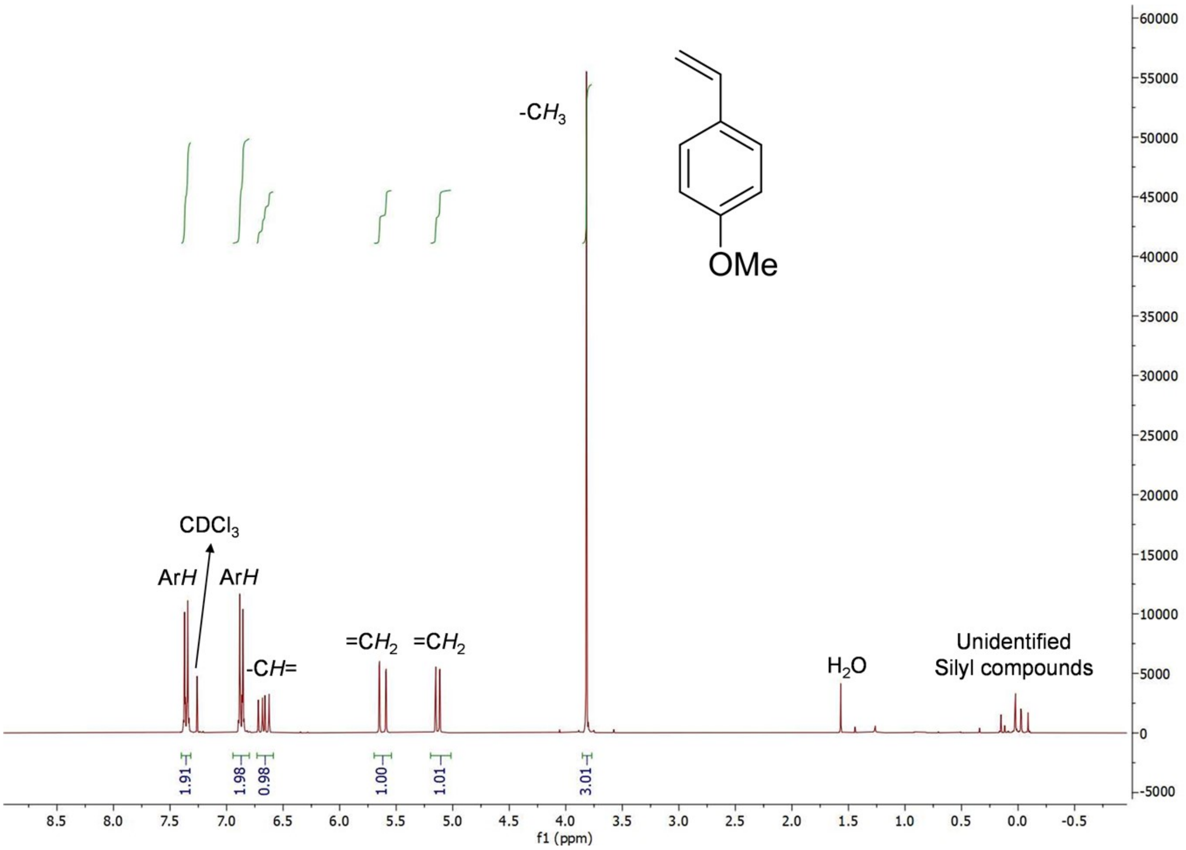

Figure S50: ^{1}H NMR (CDCl$_3$, 25 ˚C, 300 MHz) of 4-methoxystyrene (**4o**).

4-(Methylthio)benzaldehyde (2p) to 4-methythiostyrene (4p)
Reaction time:
Step-A Nucleophilic Addition: 1h
Step-B Methylenation: 2h 10min

4p was synthesized according to the general procedure. The crude product was extracted by diethyl ether and purified by column chromatography (hexanes : dichloromethane = 1:0 to 10:1) with isolated yield of 67 % (0.4991 g, colourless oil).

^{1}H NMR (400 MHz, CDCl$_3$, 25 °C): δ (ppm) 7.36-7.31 (m, 2H, Ar*H*), 7.23-7.19 (m, 2H, Ar*H*), 6.67 (dd, $^3J_{HH}$ (trans) = 17.6 Hz, $^3J_{HH}$ (cis) = 10.9 Hz, 1H, -C*H*=), 5.71 (d, $^3J_{HH}$ (trans) = 17.6 Hz, 1H, =CH_2), 5.21 (d, $^3J_{HH}$ (cis) = 10.9 Hz, 1H, =CH_2), 2.49 (s, 3H, -CH_3).

NMR data are consistent with the literature.[20]

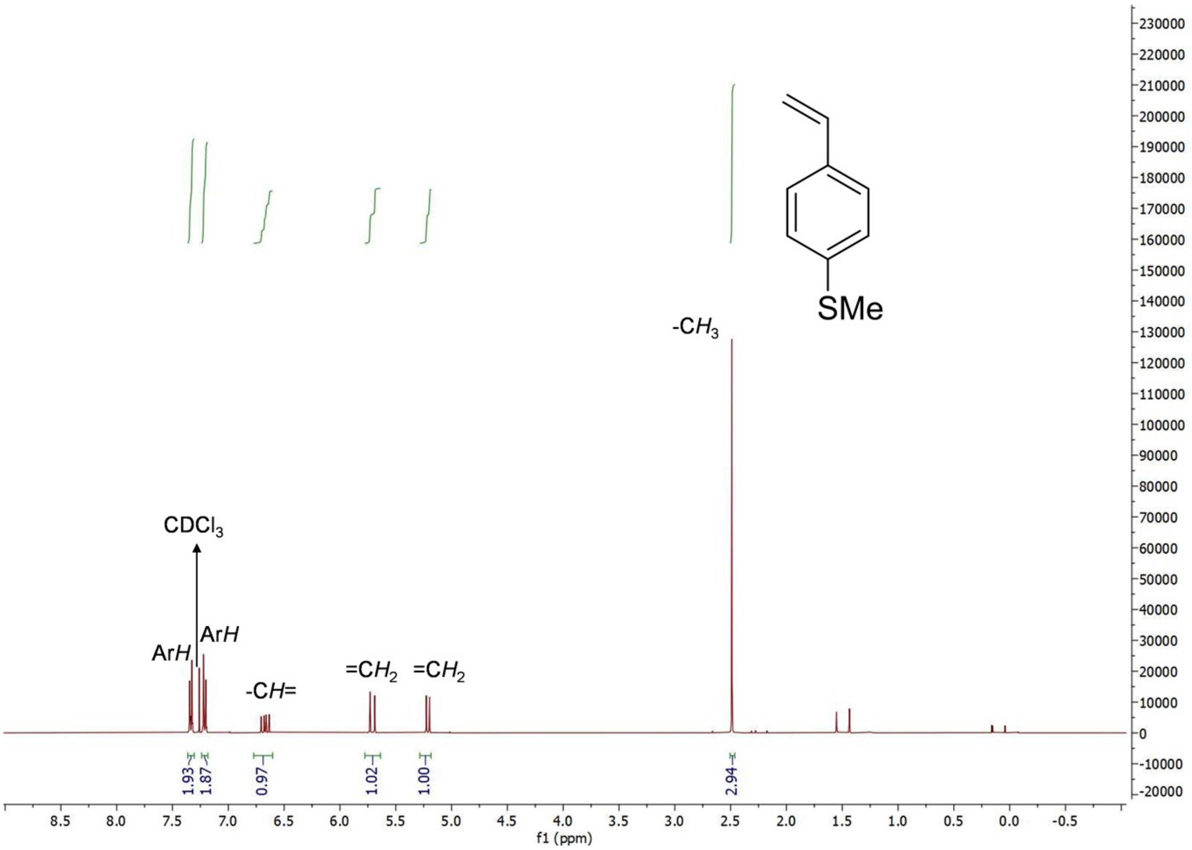

Figure S51: ^{1}H NMR ($CDCl_3$, 25 ˚C, 400 MHz) of 4-methythiostyrene (**4p**).

4-*tert*-Butyl benzaldehyde (2q) to 4-*tert*-butylstyrene (4q)
Reaction time:
Step-A Nucleophilic Addition: 1h 15min
Step-B Methylenation: 20h

4q was synthesized according to the general procedure. The crude product was extracted by diethyl ether and purified by column chromatography (hexanes : ethyl acetate= 1:0 to 20:1) with isolated yield of 54 % (0.4292 g, colourless liquid).

^{1}H NMR (400 MHz, $CDCl_3$, 25 °C): δ (ppm) 7.39 (s, 4H, Ar*H*), 6.73 (dd, $^3J_{HH}$ (trans) = 17.6 Hz, $^3J_{HH}$ (cis) = 10.8 Hz, 1H, -C*H*=), 5.74 (d, $^3J_{HH}$ (trans) = 17.6, Hz, 1H, =CH_2), 5.23 (d, $^3J_{HH}$ (cis) = 10.8, 1.0 Hz, 1H, =CH_2), 1.35 (s, 9H, -^{t}Bu).

NMR data are consistent with the literature.[21]

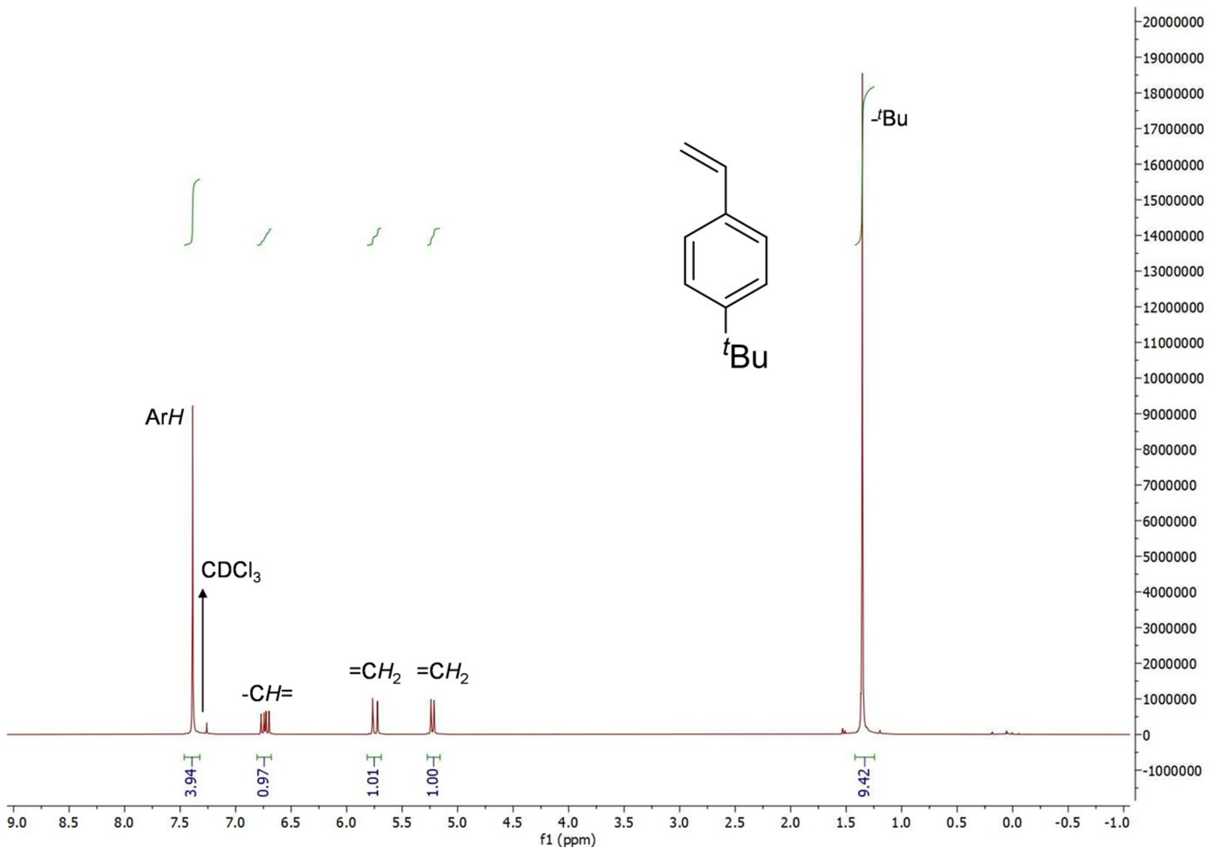

Figure S52: ^{1}H NMR ($CDCl_3$, 25 ˚C, 400 MHz) of 4-*tert*-butylstyrene (**4q**).

Adamantane-1-carbaldehyde (2r) to 1-vinyladamantane (4r)
Reaction time:
Step-A Nucleophilic Addition: 1h 30min
Step-B Methylenation: 2h 15min

4r was synthesized according to the general procedure. The crude product was extracted by diethyl ether and purified by column chromatography (hexanes : ethyl acetate= 1:0 to 20:1) with isolated yield of 41 % (0.3299 g, colourless oil).

^{1}H NMR (400 MHz, $CDCl_3$, 25 °C): δ (ppm) 5.71 (dd, $^{3}J_{HH}$ (trans) = 17.4 Hz, $^{3}J_{HH}$ (cis) = 10.9 Hz, 1H, -C*H*=), 4.88-4.85 (m, 1H, =CH_2), 4.83 (s, 1H, =CH_2), 1.99 (s, 3H, -C*H*(CH_2-)-), 1.77-1.62 (m, 6H, -CH_2-), 1.60-1.56 (m, 6H, -CH_2-).

NMR data are consistent with the literature.[22]

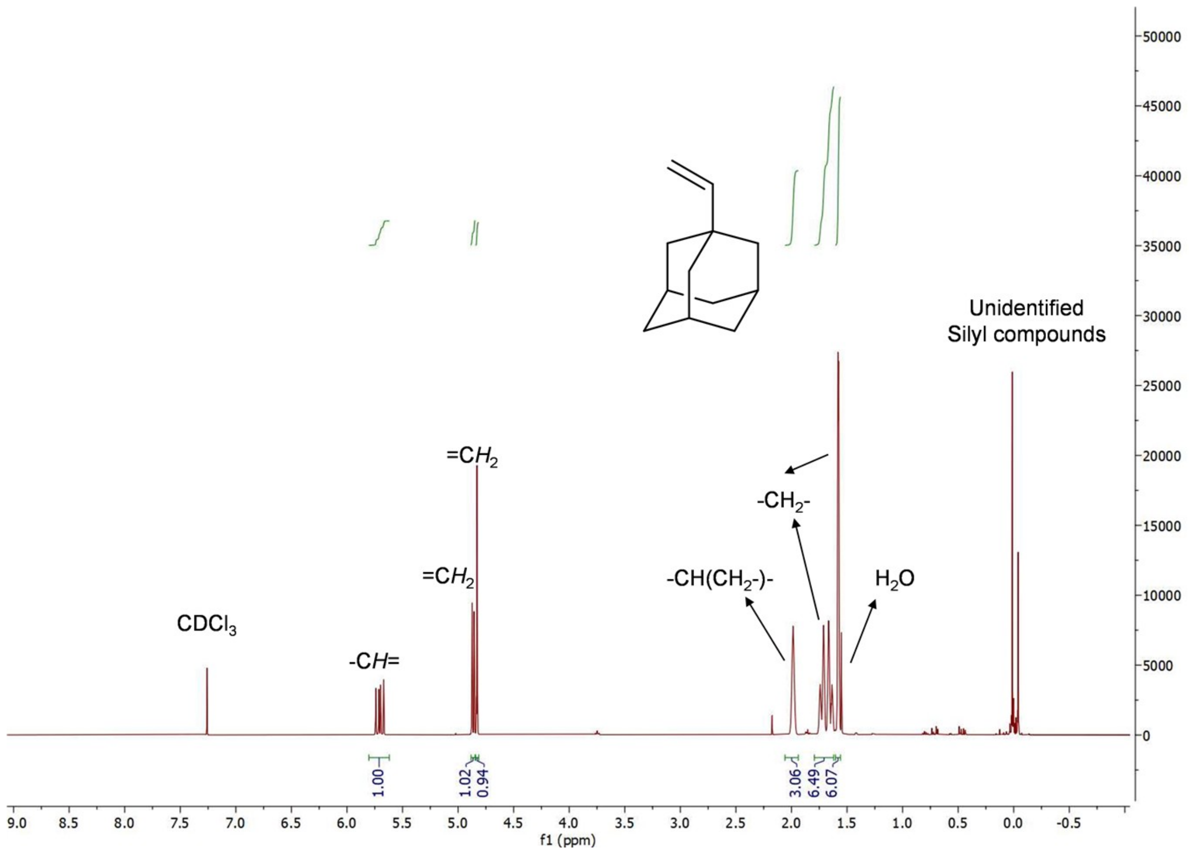

Figure S53: ^{1}H NMR ($CDCl_3$, 25 ˚C, 400 MHz) of 1-vinyladamantane (**4r**).

4-(pyridin-4-yl)benzaldehyde (2s) to 4-(4-Vinylphenyl)pyridine (4s)
Reaction time:
Step-A Nucleophilic Addition: 1h 15min
Step-B Methylenation: 4h 30min

The attempt to synthesise **4s** failed. No characteristic peaks of the methylenation product, **4s**, were found in NMR spectrum of the crude product.[23]

Terephthalaldehyde (2t) to 1,4-divinylbenzene (4t)
The attempt to synthesise **4t** failed. No characteristic peaks of the bis-methylenation product, **4t**, were found in NMR spectrum of the crude product.[24]

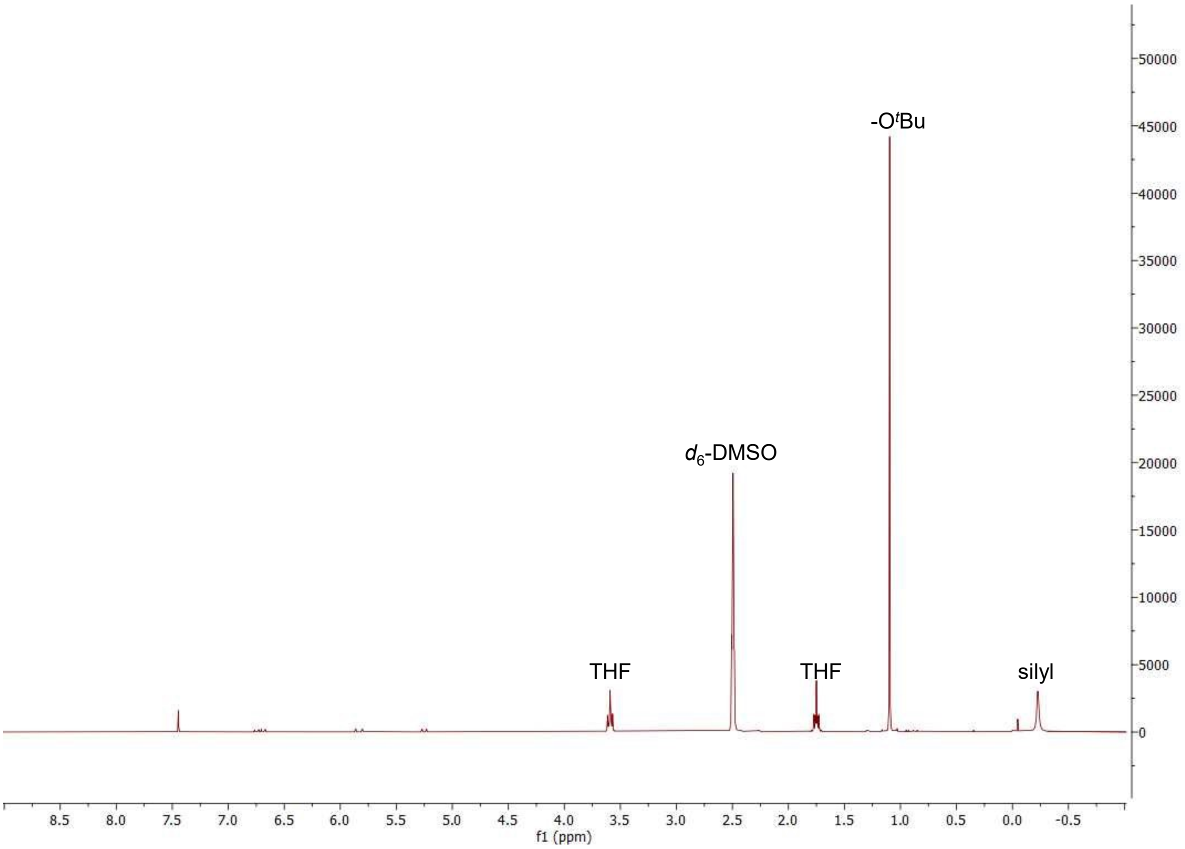

Figure S54: ^{1}H NMR (d_6-DMSO, 25 °C, 300 MHz) of the crude product before extraction in the attempt to synthesize 1,4-divinylbenzene **(4t)**.

Transmetallation for amides
4-Methoxy-*N*,*N*-diphenylbenzamide (2u) to 4-Methoxyacetophenone (4u)
Reaction time:
Step-A Nucleophilic Addition: 20 h 10 min
Step-B Methylenation: 4 h 30 min

4u was also synthesized according to general procedure but the reaction time of step-A was 20 h. The crude product was extracted by diethyl ether and purified by column chromatography (hexanes : ethyl acetate= 1:0 to 20:1, then 10:1, finally 5:1) with isolated yield of 42.0 % (0.3154 g, colourless oil).

^{1}H NMR (300 MHz, $CDCl_3$, 25 °C): δ (ppm) 7.98-7.90 (m, 2H, Ar*H*), 6.97-6.90 (m, 2H, Ar*H*), 3.87 (s, 3H, -COCH_3), 2.56 (s, 3H, -OCH_3).

NMR data are consistent with the literature.[25]

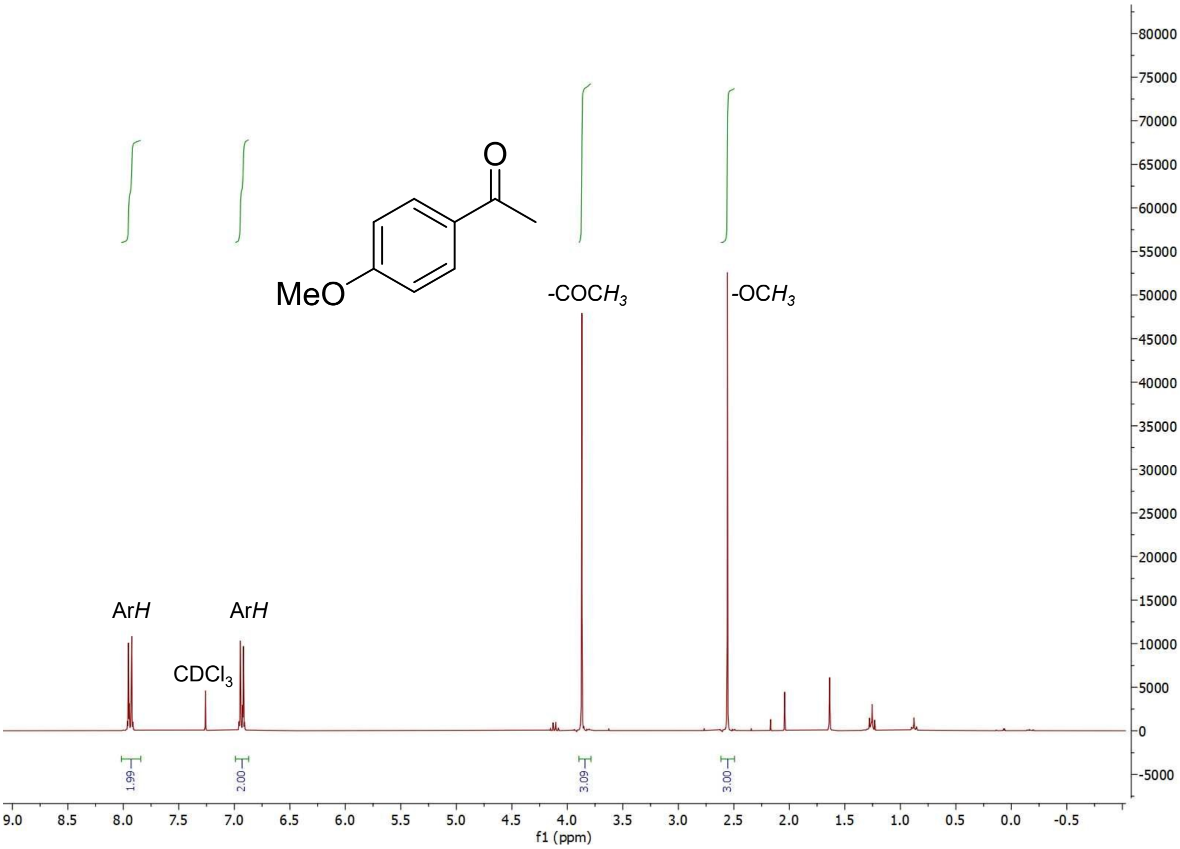

Figure S55: ^{1}H NMR ($CDCl_3$, 25 ˚C, 300 MHz) of 4-methoxyacetophenone (**4u**).

***N*-Benzoylpiperidine (2v) to acetonphenone (4v)**
Reaction time:
Step-A Nucleophilic Addition: 1h 30 min
Step-B Methylenation: 1 d

4v was also synthesized according to general procedure but the reaction time of step-B was elongated to 1 day. There was different reaction mechanism for formation of **4v** from corresponding amide, *N*-benzoylpiperidine (**2v**). The crude product was extracted by diethyl ether and purified by column chromatography (hexanes : ethyl acetate= 1:0 to 20:1) with isolated yield of 54 % (0.3216 g, colourless oil).

^{1}H NMR (400 MHz, $CDCl_3$, 25 °C): δ (ppm) 8.00-7.93 (m, 2H, Ar*H*), 7.60-7.54 (m, 1H, Ar*H*), 7.51-7.44 (m, 2H, Ar*H*), 2.61 (s, 3H, -C*H*$_3$).

NMR data are consistent with the literature.[26]

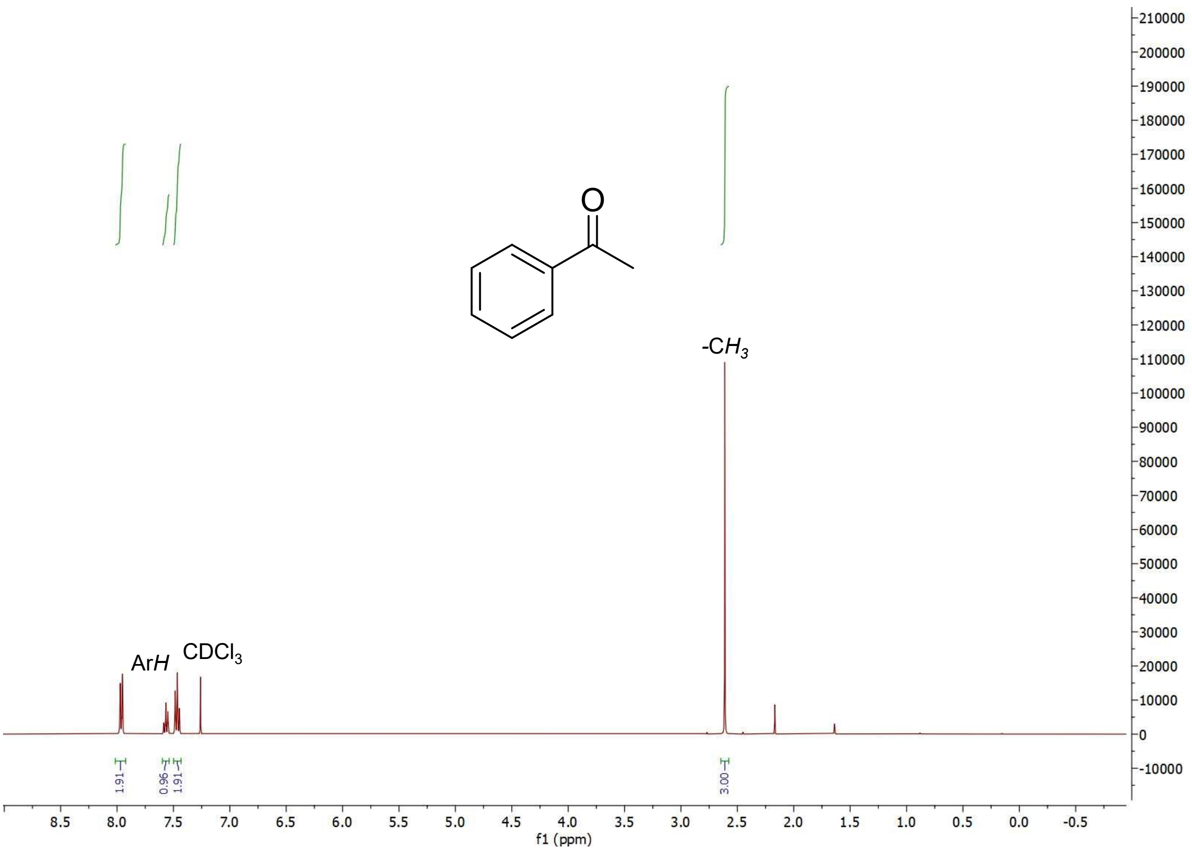

Figure S56: ^{1}H NMR ($CDCl_3$, 25 ˚C, 400 MHz) of acetonphenone (**4v**).

1.5 Single Crystal X-Ray Diffraction

Crystal structure data were collected on a XtaLAB Synergy, Single source at home/near, HyPix-Arc 100 diffractometer equipped with a fine-focus sealed X-ray tube ($\lambda_{CuK\alpha}$ = 1.54184 Å) at 150 K using an Oxford Cryosystems CryostreamPlus open-flow N_2 cooling device. Cell refinement, data collection and data reduction were undertaken via software CrysAlisPro (Rigaku OD, 2023). Intensities were corrected for absorption using a multifaceted crystal model based on expressions derived by Clark & Reid (Clark & Reid, 1995).

Using Olex2 (Dolomanov, 2009), the structure was solved using SHELXT (Sheldrick, 2018) and refined by SHELXL (Sheldrick, 2015). All non-hydrogen atoms were refined anisotropically and hydrogen atoms were positioned with idealised geometry, with displacement parameters constrained using a riding model with $U_{(H)}$ set to be an appropriate multiple of the U_{eq} value of the parent atom.

CrysAlisPro, Rigaku Oxford Diffraction, Tokyo, Japan.
Clark, R. C. & Reid, J. S. (1995). Acta Cryst. A51, 887-897
Dolomanov, O.V., Bourhis, L.J., Gildea, R.J, Howard, J.A.K. & Puschmann, H. (2009), J. Appl. Cryst. 42, 339-341.

Sheldrick, G.M. (2008). Acta Cryst. A64, 112-122.
Sheldrick, G.M. (2015). Acta Cryst. A71, 3-8.

Table S1. Crystal Structure Refinement Details for Complexes **3K** and $[KOSiMe_3]_4$.

Compound	**3K**	$[KOSiMe_3]_4$
Empirical formula	$C_{82}H_{112}K_4O_4Si_4$	$C_{12}H_{36}K_4O_4Si_4$
Formula weight	1430.47	513.17
Temperature/K	150.0(2)	150.0(2)
Crystal system	triclinic	cubic
Space group	P-1	F-43c
a/Å	12.8781(2)	17.5314(3)
b/Å	16.8307(2)	17.5314(3)
c/Å	19.5417(2)	17.5314(3)
α/°	100.8590(10)	90
β/°	90.4230(10)	90
γ/°	94.8410(10)	90
Volume/Å^3	4143.76(9)	5388.3(3)
Z	2	8
ρ_{calc}g/cm^3	1.146	1.265
μ/mm^{-1}	2.806	7.691
F(000)	1536.0	2176.0
Crystal size/mm^3	0.16 × 0.06 × 0.04	0.2 × 0.18 × 0.1
Radiation	CuKα (λ = 1.54184)	CuKα (λ = 1.54184)
2Θ range for data collection/°	4.606 to 155.562	10.092 to 146.824
Index ranges	$-15 \leq h \leq 16$, $-21 \leq k \leq 21$, $-20 \leq l \leq 23$	$-20 \leq h \leq 18$, $-20 \leq k \leq 21$, $-19 \leq l \leq 13$
Reflections collected	76812	2632
Independent reflections	16585 [R_{int} = 0.0380, R_{sigma} = 0.0288]	458 [R_{int} = 0.0270, R_{sigma} = 0.0245]
Data/restraints/parameters	16585/1767/990	458/0/21
Goodness-of-fit on F^2	1.062	1.120
Final R indexes [I>=2σ (I)]	R_1 = 0.0486, wR_2 = 0.1157	R_1 = 0.0185, wR_2 = 0.0510
Final R indexes [all data]	R_1 = 0.0596, wR_2 = 0.1210	R_1 = 0.0194, wR_2 = 0.0517
Largest diff. peak/hole / e Å^{-3}	0.44/-0.34	0.16/-0.14
Flack parameter	n/a	0.026(16)

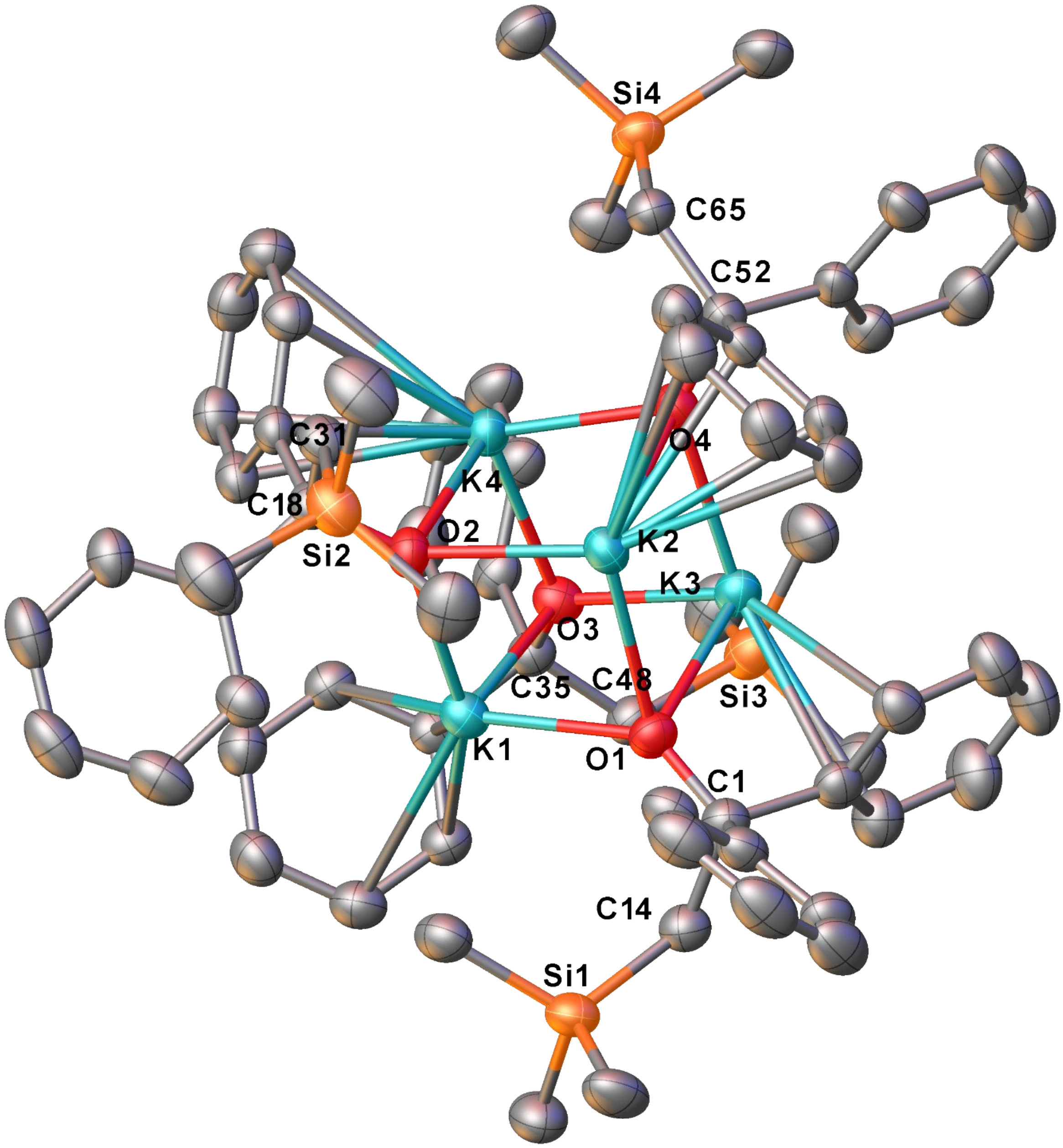

Figure S57: Single crystal structure of **3K** with ellipsoids drawn at the 50% probability level. Key bond lengths (Å): K1–O1 2.5929(15), K1–O2 2.6207(15), K1–O3 2.7756(15), K2–O1 2.6502(15), K2–O2 2.5690(15), K2–O4 2.8410(15), K3–O1 2.7568(15), K3–O3 2.6071(15), K3–O4 2.6284(15), K4–O2 2.8737(15), K4–O3 2.6201(15), K4–O4 2.5769(14), O1–C1 1.387(3), O2–C18 1.387(2), O3–C35 1.392(2), O4–C52 1.392(2), C1–C14 1.563(3), C18–C31 1.559(3), C35–C48 1.563(3), C52–C65 1.559(3), Si1–C14 1.881(2), Si2–C31 1.880(2), Si3–C48 1.880(2), Si4–C65 1.882(2). Hydrogen atoms and two molecules of disordered methylcyclohexane are omitted for clarity. Key: grey: carbon; teal: potassium; blue: nitrogen; orange: silicon: red: oxygen.

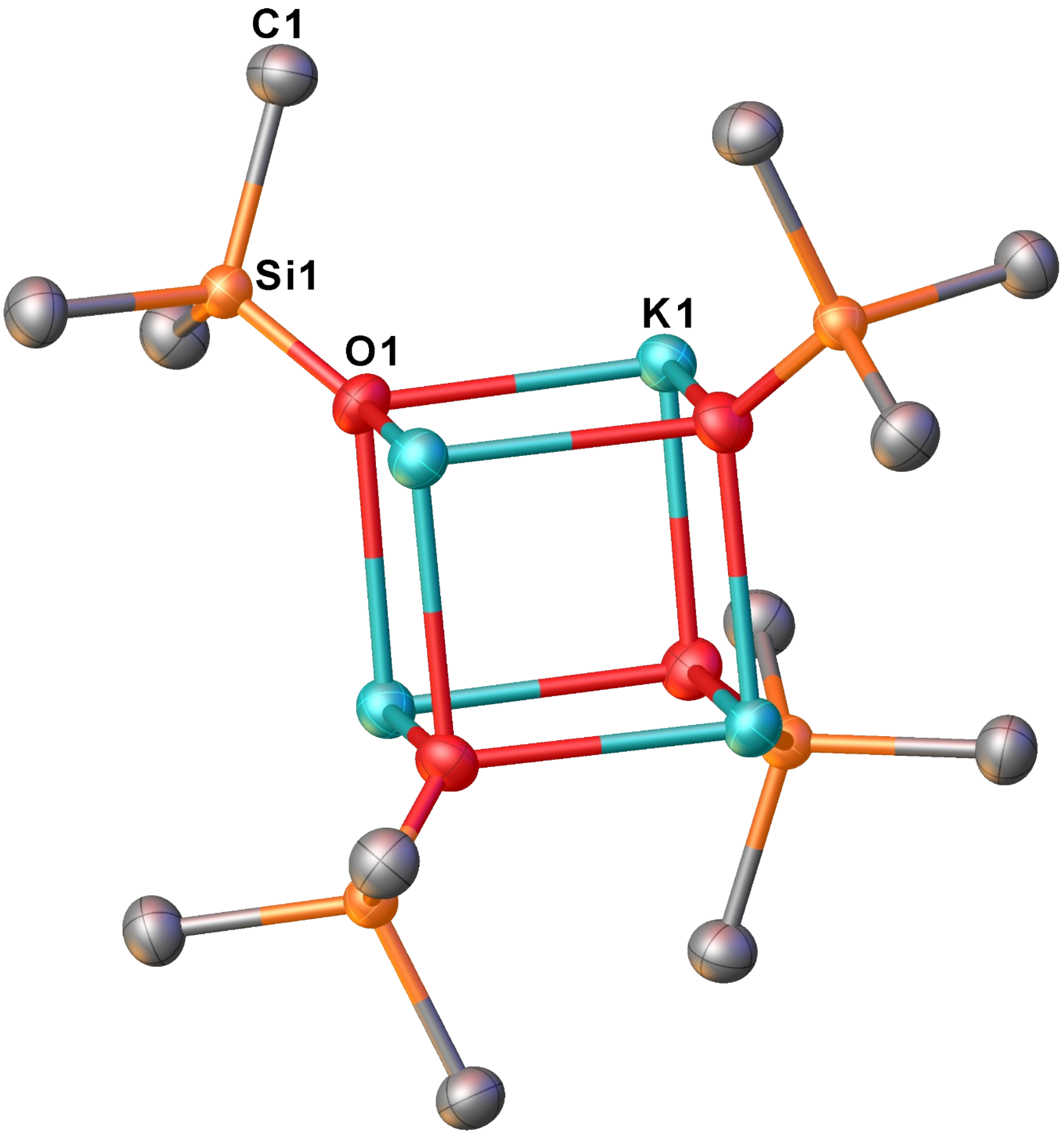

Figure S58: Single crystal structure of $[KOSiMe_3]_4$ with ellipsoids drawn at the 50% probability level. Key bond lengths (Å): K1–O1 2.6257(12), Si1–O1 1.584(2), Si1–C1 1.881(2). Hydrogen atoms are omitted for clarity. Only the crystallographically independent atoms are labelled. Key: grey: carbon; teal: potassium; orange: silicon: red: oxygen.

Section 2. Computational Details and Data

2.1 Computational Methodology

DFT calculations were run with Gaussian 16 (C.01).[27] The K, Na and Si centres were described with the Stuttgart RECPs and associated basis sets,[28] and 6-31G** basis sets were used for all other atoms (BS1).[29] A polarization function was also added to K (ζ_d = 1.000) and Si (ζ_d = 0.284). Initial BP86[30] optimizations were performed using the 'grid = ultrafine' option, with all stationary points being fully characterized via analytical frequency calculations as minima (all positive eigenvalues) or a transition state (one negative eigenvalue). All energies were recomputed with a larger basis set featuring 6-311++G** on all atoms. Corrections for the effect of benzene (ε = 2.2706) solvent were run using the polarizable continuum model and BS1.[31] Single-point dispersion corrections to the BP86 results employed Grimme's D3 parameter set with Becke-Johnson damping as implemented in Gaussian.[32]

2.2 Breakdown of Energy Contributions

The following tables detail the evolution of the relative energies as the successive corrections to the initial SCF energy are included. Terms used are:

ΔE_{BSI}	SCF energy computed with the BP86 functional with BS1
ΔH_{BSI}	Enthalpy at 0 K with BS1
ΔG_{BSI}	Free energy at 298.15 K and 1 atm with BS1
$\Delta G_{BSI/bnz}$	Free energy corrected for benzene solvent with BS1
$\Delta G_{BSI/bnz+D3BJ}$	Free energy corrected for benzene and dispersion effects with BS1
ΔE_{BS2}	SCF energy computed with the BP86 functional with BS2
ΔG_{bnz}	Free energy corrected for basis set (BS2), dispersion effects and benzene solvent

In each case the final data used in the main article are highlighted in bold.

Table S2. Relative energies for computed structures. Data in bold are those used in the main text. Free energies are quoted relative to complex **1**-K and benzophenone at 0.0 kcal mol^{-1}.

	ΔE_{BSI}	ΔH_{BSI}	ΔG_{BSI}	$\Delta G_{BSI/bnz}$	$\Delta G_{BSI/bnz + D3BJ}$	ΔE_{BS2}	ΔG_{bnz}
**1**K (κ^4)	0.0	0.0	0.0	0.0	0.0	0.0	**0.0**
**1**K (κ^3)	5.4	5.7	4.9	3.5	8.2	4.7	**7.4**
**A**-K	-4.9	3.8	5.1	7.3	3.5	-3.6	**4.8**
**TS(A-B)**-K	-1.6	7.0	11.1	12.2	5.2	-0.8	**6.0**
**B**-K	-30.7	-20.3	-12.0	-9.1	-23.0	-29.0	**-21.4**
**TS(B-C)**-K	-25.9	-16.2	-6.0	-3.1	-17.9	-24.2	**-16.2**
**C**-K	-31.3	-21.0	-13.7	-11.0	-25.8	-29.9	**-24.3**
**TS(C-D)**-K	-10.8	-2.2	5.4	7.0	-9.9	-10.0	**-9.1**
**D**-K	-50.3	-39.7	-33.4	-30.8	-45.9	-51.3	**-46.9**
**3**-K	-158.4	-170.9	-85.6	-65.9	-137.1	-150.4	**-129.1**

The reaction profile for **1**K is similar in shape to the equivalent Na and Li free energy surfaces that were previously reported[28]. The rate limiting step remains unchanged, with the β-silyl abstraction step of **TS(C-D)**-K having a barrier of 15.2 kcal mol^{-1} ($\Delta\Delta G^{\ddagger}$ = 17.7 (Na) and 20.3 (Li) kcal mol^{-1}).

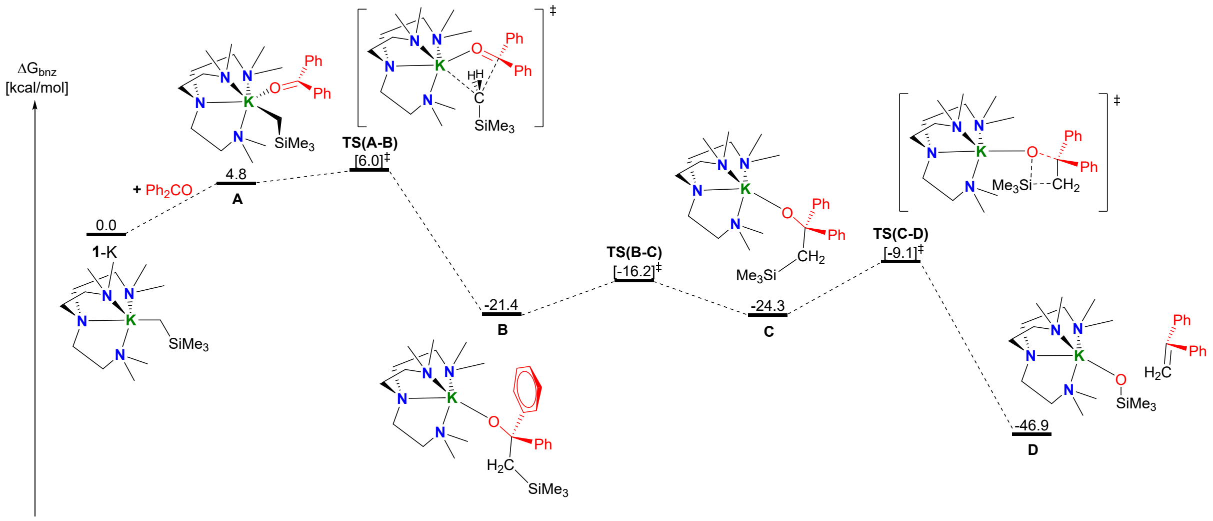

Figure S59: DFT calculated free energy profile (BP86-D3BJ(C_6H_6)/6-311++G**//BP86/6-31G**&SDDALL, in kcal mol^{-1}) for the reaction of **1**K with benzophenone.

Table S3. Relative energies for computed structures. Data in bold are those used in the main text. Free energies are quoted relative to complex $K(CH_2SiMe_3)$ and benzophenone at 0.0 kcal mol^{-1}.

	ΔE_{BSI}	ΔH_{BSI}	ΔG_{BSI}	$\Delta G_{BSI/bnz}$	$\Delta G_{BSI/bnz + D3BJ}$	ΔE_{BS2}	ΔG_{bnz}
^{K}**A**	-13.6	-5.2	-5.3	-1.2	-5.2	-12.3	**–3.9**
^{K}**TS(A-B)**	-2.3	5.7	10.8	11.4	1.5	-2.6	**1.1**
^{K}**B**	-26.6	-17.0	-9.7	-9.1	-22.1	-30.4	**–25.9**
^{K}**TS(B-C)**	-21.4	-12.5	-4.8	-4.5	-17.7	-23.8	**–20.2**
^{K}**C**	-30.1	-20.5	-13.2	-11.4	-25.0	-31.7	**–26.7**
^{K}**TS(C-D)**	-11.6	-3.5	4.2	5.3	-10.2	-13.0	**–11.6**
^{K}**D**	-52.5	-42.7	-40.5	-39.0	-46.0	-57.0	**–50.4**

Again, the reaction profile for $K(CH_2SiMe_2)$ is similar in shape to the equivalent Na and Li free energy surfaces that were previously reported[28]. However, the profile is ~20 kcal mol^{-1} lower, beginning with ^{K}**A** at -3.9 kcal mol^{-1} (when ^{Na}**A** = 16.3 and ^{Li}**A** = 16.1 kcal mol^{-1}). The rate limiting step remains unchanged, with the β-silyl abstraction step of ^{K}**TS(C-D)** having a barrier of 15.1 kcal mol^{-1} ($\Delta\Delta G^{\ddagger}$ = 15.8 (Na) and 18.5 (Li) kcal mol^{-1}).

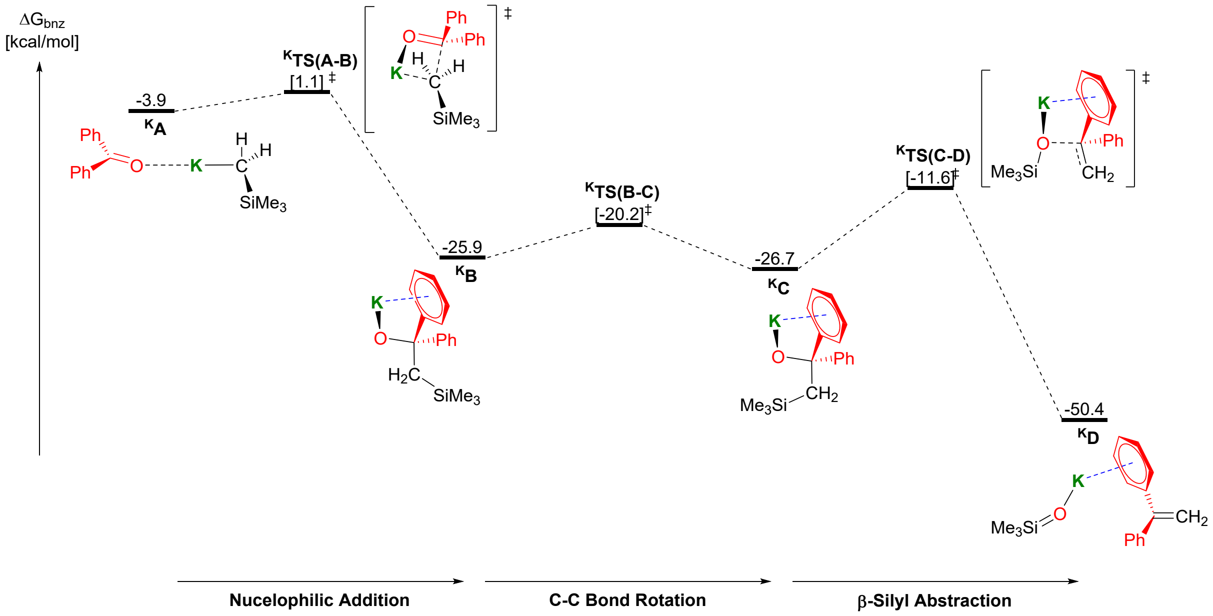

Figure S60: DFT calculated free energy profile (BP86-D3BJ(C_6H_6)/6-311++G**//BP86/6-31G**&SDDALL, in kcal mol^{-1}) for the reaction of $K(CH_2SiMe_3)$ with benzophenone.

Table S4. Relative energies for computed structures. Data in bold are those used in the main text. Free energies are quoted relative to the monomer **1**M and benzophenone at 0.0 kcal mol^{-1}, therefore, the relative energies of the tetrameric species (**3**M, $\mathbf{E^M}$ and $\mathbf{F^M}$) are calculated by taking a quarter of the M_4 species and adding in equivalent numbers of the ligand Me_6Tren (L) to balance.

	ΔE_{BSI}	ΔH_{BSI}	ΔG_{BSI}	$\Delta G_{BSI/bnz}$	$\Delta G_{BSI/bnz + D3BJ}$	ΔE_{BS2}	ΔG_{bnz}
**1**Na (κ^4)	0.0	0.0	0.0	0.0	0.0	0.0	**0.0**
**3**Na	-35.4	-17.0	-19.1	-15.2	-34.4	-31.7	**–30.7**
**3**Na·κ^{1}L	-33.1	-11.1	-14.1	-10.2	-34.7	-28.7	**–30.3**
**3**Na·κ^{2}L	-27.9	-5.9	-8.8	-5.8	-31.1	-23.6	**–26.8**
**3**Na·2κ^{1}L	-31.9	-6.3	-10.2	-6.2	-35.2	-26.9	**–30.2**
$\mathbf{E}^{Na}$	-26.7	-23.6	-9.3	-6.9	-23.9	-23.7	**–20.9**
**TS(E-F)**^{Na}	-21.5	-18.7	-4.3	-2.0	-19.7	-18.6	**–16.7**
$\mathbf{F}^{Na}$	-31.9	-28.9	-14.9	-12.6	-29.1	-29.5	**–26.7**
(κ^2-Me_6Tren) Na(μ-OR) $Na_3(OR)_3$	-28.2	-25.0	-9.7	-6.7	-29.2	-24.3	**–25.3**
$\mathbf{C}^{Na}$ (κ^2)	-26.5	-23.5	-12.5	-10.3	-20.7	-24.4	**-18.7**
**TS(C-D)**^{Na} (κ^2)	1.0	2.1	14.5	14.3	1.5	1.7	**2.2**
$\mathbf{D}^{Na}$ (κ^2)	-39.9	-38.2	-30.3	-30.4	-33.5	-42.3	**-35.9**
**1**Li (κ^4)	0.0	0.0	0.0	0.0	0.0	0.0	**0.0**
**3**Li	-38.6	-20.9	-23.6	-20.6	-39.7	-34.5	**–35.6**
**3**Li·κ^{1}L	-34.9	-31.6	-16.3	-13.2	-37.4	-30.1	**–32.6**
$\mathbf{E}^{Li}$	-33.5	-30.6	-19.1	-17.2	-26.0	-30.1	**–22.7**
**TS(E-F)**^{Li}	-27.0	-24.3	-12.0	-9.8	-20.4	-23.9	**–17.3**
$\mathbf{F}^{Li}$	-38.8	-36.1	-24.8	-22.8	-30.3	-36.3	**–27.7**
**1**K (κ^3)	0.0	0.0	0.0	0.0	0.0	0.0	**0.0**
**3**K	-39.6	-42.7	-21.4	-16.5	-34.3	-37.6	**–32.3**
**3**K·κ^{1}L	-38.2	-56.6	-17.0	-12.0	-34.7	-35.4	**–31.9**
**3**K·2κ^{1}L	-37.3	-55.8	-13.8	-8.8	-35.5	-34.0	**–32.2**
**3**K''	-38.4	-56.8	-20.1	-15.2	-33.8	-36.2	**–31.6**
**3**K''-TS(c-d)	-30.5	-49.1	-12.0	-7.3	-26.1	-28.2	**–23.8**
**3**K''-d	-44.8	-63.5	-28.5	-24.0	-37.6	-43.9	**–36.8**

2.2 Additional Computational Mechanism Discussion

3M – alternative conformations:

Upon closer inspection of **3**M, it should be noted that of the four R groups, two are in an *anti*-configuration ($\tau_{O\text{-}C\text{-}C\text{-}Si} = -170$ °), whilst the other two could be considered as *gauche*, with O-C-C-Si dihedrals of 73 °. A fully "*anti*" **3**M conformer was computed to be higher in free energy by 0.6 kcal mol^{-1} for all three metal tetramers, and are labelled as **3**M'.

Table S5 Relative energies for computed structures. Data in bold are those used in the main text. Free energies are quoted relative to the monomer **1**M and benzophenone at 0.0 kcal mol^{-1}, therefore, the relative energies of the tetrameric species (**3**M) are calculated by taking a quarter of the M_4 species and adding in equivalent numbers of the ligand Me_6Tren (L) to balance.

	ΔE_{BSI}	ΔH_{BSI}	ΔG_{BSI}	$\Delta G_{BSI/bnz}$	$\Delta G_{BSI/bnz\,+\,D3BJ}$	ΔE_{BS2}	ΔG_{bnz}
**1**Na (κ^4)	0.0	0.0	0.0	0.0	0.0	0.0	**0.0**
**3**Na	-35.4	-17.0	-19.1	-15.2	-34.4	-31.7	**−30.7**
**3**Na'	-35.4	-32.2	-20.3	-16.8	-33.3	-32.1	**−29.9**
**1**Li (κ^4)	0.0	0.0	0.0	0.0	0.0	0.0	**0.0**
**3**Li	-38.6	-20.9	-23.6	-20.6	-39.7	-34.5	**−35.6**
**3**Li′	-39.7	-36.6	-25.6	-22.8	-38.9	-35.8	**−35.0**
**1**K (κ^3)	0.0	0.0	0.0	0.0	0.0	0.0	**0.0**
**3**K	-39.6	-42.7	-21.4	-16.5	-34.3	-37.6	**−32.3**
**3**K′	-40.0	-58.4	-22.0	-17.3	-33.4	-38.2	**−31.7**
$Li_3K(OR)_4$	-39.1	-41.3	-23.1	-19.8	-38.7	-35.1	**-34.6**
$Li_2K_2(OR)_4$	-38.9	-46.5	-22.0	-18.3	-36.9	-35.4	**-33.3**
$LiK_3(OR)_4$	-39.2	-52.2	-21.6	-17.1	-36.1	-36.4	**-33.2**

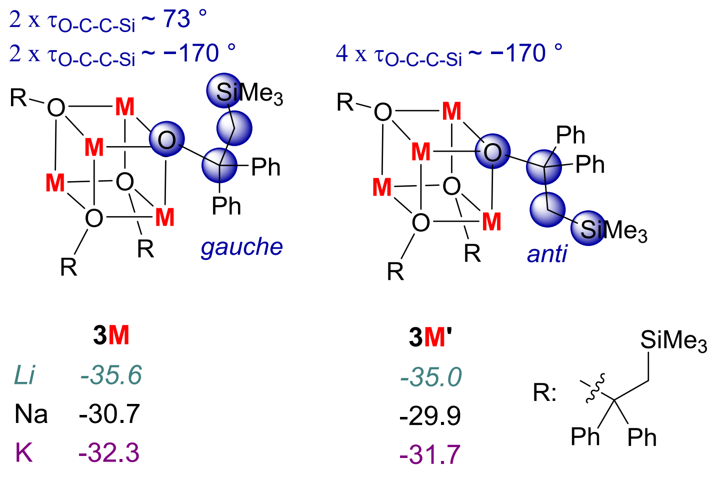

Figure S61: DFT computed free energies of **3**M and **3**M'in kcal mol^{-1} (BP86-D3BJ(C_6H_6)/BS2//BP86/BS1). Free energy values are relative to **1**M and benzophenone using ¼ **3**M. R = $OC(CH_2SiMe_3)Ph_2$, L = Me_6Tren.

3K'' ligandless silyl abstraction:

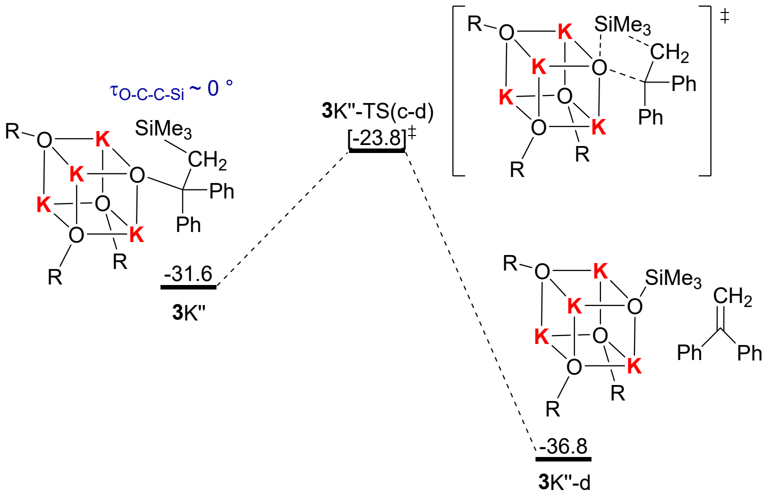

Figure S62: DFT computed free energies for silyl abstraction at **3**K, in kcal mol^{-1} (BP86-D3BJ(C_6H_6)/BS2//BP86/BS1). Free energy values are relative to **1**K and benzophenone using ¼ **3**K. R = OC(CH_2SiMe_3)Ph_2, L = Me_6Tren.

3M + 2 × Me_6Tren :

There is little impact on the free energy of the tetramer as two ligands are coordinated to two different metal centers when forming the intermediate **3**M·2(κ^{1}L), changing by +0.5 and +0.1 kcal mol^{-1}, for M = Na and K respectively (Figure S70).

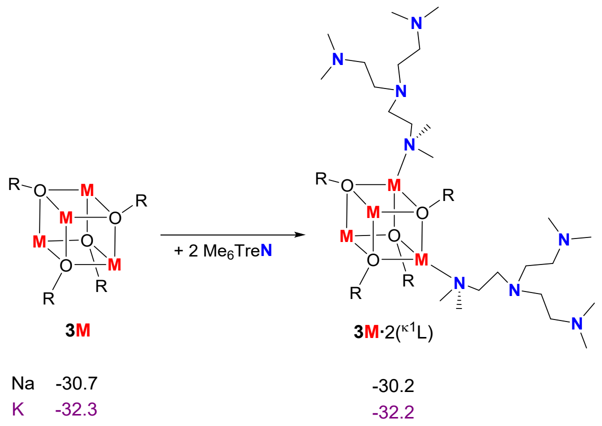

Figure S63: DFT computed free energies for addition of two Me_6TreN (L) to **3**M, in kcal mol^{-1} (BP86-D3BJ(C_6H_6)/BS2//BP86/BS1). Free energy values are relative to **1**M and benzophenone using ¼ **3**M. R = $OC(CH_2SiMe_3)Ph_2$, L = Me_6Tren.

Coordination of a second arm of the Me_6Tren ligand was only successfully optimised for **3**Na, with intermediate **3**Na·$^{\kappa^2}L$ (ΔG = −26.8 kcal mol^{-1}), with disaggregation of the tetrametallic cube observed as the coordinated Na center has significantly elongated Na···O distances of 4.775 and 4.057 Å to two of it's three oxygen contacts (see Figure S71). Further elongation along these bonds as a monomeric "**1**Na" unit, [(κ^2-Me_6Tren)Na(OR)], detaches from the cluster to give a $Na_3(OR)_3$ trimer adduct connected through a bridging "OR" ligand ((κ^2-Me_6Tren)Na(μ-OR) $Na_3(OR)_3$; ΔG = −25.3 kcal mol^{-1}).

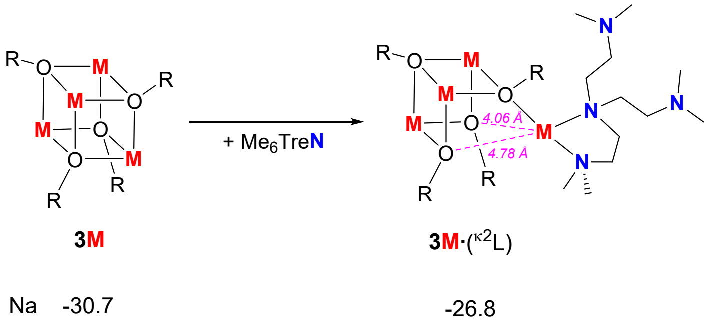

Figure S64: DFT computed free energies for addition of one Me_6TreN (L) to **3**Na, in kcal mol^{-1} (BP86-D3BJ(C_6H_6)/BS2//BP86/BS1). Free energy values are relative to **1**Na and benzophenone using ¼ **3**Na. R = OC(CH_2SiMe_3)Ph_2, L = Me_6Tren.

<u>Dissociation of a monomeric unit from **3**Na:</u>

Logically, following on from elongation of the "Na(OR)L" monomeric unit as discussed above, the monomeric species can rejoin the monomer reaction pathway, such as which we previously reported[28]. Attempts to coordinate a third arm of the Me_6Tren ligand to the departing monomer unit were unsuccessful, however, silyl abstraction was modelled from **C**^{Na} (κ^2), which at −18.7 kcal mol^{-1} is 2.0 kcal mol^{-1} higher in free energy than **C**^{Na} (κ^3)[28]. A barrier of 20.9 kcal mol^{-1} was located (**TS(C-D)**^{Na} (κ^2); ΔG = 2.2 kcal mol^{-1}) affording the methylenated substrate and **D**^{Na} (κ^2) at −35.9 kcal mol^{-1}. The silyl abstraction barrier at a κ^2 Me_6Tren bound ligand to Na is 5.2 kcal mol^{-1} higher than when the Me_6Tren ligand is triscoordinated.[28]

Further decomposition of the remaining trimeric $Na_3(OR)_3$ cluster through further ligand coordination is a likely progression of the tetramer disaggregation. It is important to note that full disaggregation is calculated to be highly endergonic (4 × **C**^{Na} (κ^3); ΔG = +40.0 kcal mol^{-1}, relative to **3**Na and 4 Me_6TreN ligands), suggesting that a level of aggregation may remain during the silyl abstraction.

Cartesian Coordinates and Raw Electronic Energies (in Hartrees)

1K + benzophenone

benzophenone
SCF (BP86) Energy = -576.623309717
Enthalpy 0K = -576.437367
Enthalpy 298K = -576.425338
Free Energy 298K = -576.475236
Lowest Frequency = 42.1627 cm^{-1}
Second Frequency = 64.2830 cm^{-1}
SCF (BP86-D3BJ) Energy = -576.670270946
SCF (C6H6) Energy = -576.626243463
SCF (BS2) Energy = -576.769378556

```
C   2.44536  0.99559 -0.49330
C   3.69978  0.37702 -0.45868
C   1.30634  0.33677  0.02146
H   4.57377  0.88913 -0.87469
C   3.83962 -0.89856  0.11829
C   1.45683 -0.93991  0.60707
H   4.82268 -1.38011  0.15230
H   0.59035 -1.44159  1.04850
C   2.71979 -1.54968  0.65995
H   2.82992 -2.53307  1.12889
H   2.31488  1.99757 -0.91328
C  -0.00003  1.08377  0.00031
O  -0.00008  2.32310  0.00064
C  -1.30632  0.33663 -0.02137
C  -1.45657 -0.94035 -0.60636
C  -2.44559  0.99572  0.49254
C  -2.71952 -1.55013 -0.65946
C  -3.70002  0.37720  0.45763
C  -3.83960 -0.89870 -0.11871
H  -0.58995 -1.44234 -1.04714
H  -2.31521  1.99790  0.91208
H  -2.82942 -2.53380 -1.12788
H  -4.57419  0.88955  0.87294
H  -4.82265 -1.38027 -0.15294
```

1K (κ^3)
SCF (BP86) Energy = -885.597174036
Enthalpy 0K = -885.050647
Enthalpy 298K = -885.015298
Free Energy 298K = -885.121162
Lowest Frequency = 15.1165 cm^{-1}
Second Frequency = 19.3440 cm^{-1}
SCF (BP86-D3BJ) Energy = -885.715426746
SCF (C6H6) Energy = -885.607314714
SCF (BS2) Energy = -1743.08643524

```
Si -3.96172  0.08400 -0.60476
N   1.85906  0.14120  0.14000
N   0.38829 -1.26436  2.54248
N   1.94401 -2.36396 -1.71572
N   1.48198  3.89248 -0.57523
C   2.35511 -0.01646  1.52229
H   3.06256  0.79618  1.80701
H   2.94715 -0.94796  1.55988
C   2.90989 -0.17544 -0.84874
H   2.64572  0.32652 -1.79557
C   1.21154  1.44830 -0.12230
H   0.31735  1.52421  0.52092
H   0.83552  1.43403 -1.16170
C   2.09484  2.70890  0.03915
H   2.33391  2.88375  1.11950
H   3.06041  2.53578 -0.47229
C   0.26784  4.31960  0.12846
H  -0.50729  3.53722  0.09224
H  -0.14448  5.21283 -0.36972
H   0.45460  4.58009  1.19998
C   2.44364  4.99410 -0.64592
H   2.79263  5.34575  0.35617
H   1.98281  5.85303 -1.16286
H   3.32991  4.68213 -1.22525
C  -2.88419 -1.00524 -1.62733
H  -2.58498 -0.48358 -2.56234
H  -3.44119 -1.91520 -1.93376
C  -4.63662 -0.85026  0.94172
H  -5.22744 -1.73346  0.63887
H  -5.28973 -0.21147  1.56287
H  -3.81163 -1.21248  1.58301
C  -5.52368  0.90095 -1.40821
H  -6.08459  1.54069 -0.70105
H  -6.21658  0.12643 -1.78295
H  -5.23837  1.52656 -2.27311
C  -2.95443  1.58866  0.08759
H  -2.17429  1.24456  0.79510
H  -3.58947  2.31801  0.62198
H  -2.44747  2.12497 -0.73612
K  -0.50359 -1.58152 -0.27334
C   1.25934 -0.07268  2.60097
H   1.76006  0.00703  3.59666
H   0.61254  0.81799  2.51368
C  -0.81507 -1.04993  3.36563
H  -1.46852 -1.93647  3.30665
H  -1.38253 -0.18493  2.98420
H  -0.57637 -0.86918  4.43962
C   1.09570 -2.46610  3.00920
H   1.97401 -2.67293  2.37567
H   0.42325 -3.33848  2.94743
H   1.44629 -2.37486  4.06454
C   3.10130 -1.68244 -1.10544
H   4.01848 -1.80484 -1.73349
H   3.31641 -2.19293 -0.14798
C   2.14672 -3.82070 -1.68348
H   3.04105 -4.14861 -2.26381
H   1.26328 -4.32778 -2.10742
H   2.27388 -4.16021 -0.64085
C   1.72152 -1.93046 -3.10531
H   0.85122 -2.46479 -3.52060
H   2.60175 -2.12920 -3.76092
H   1.49421 -0.85346 -3.14750
H   3.90420  0.23550 -0.55679
```

1K (κ^4)
SCF (BP86) Energy = -885.605851158
Enthalpy 0K = -885.059464
Enthalpy 298K = -885.024439

Free Energy 298K = -885.128914
Lowest Frequency = 8.8068 cm^{-1}
Second Frequency = 16.3189 cm^{-1}
SCF (BP86-D3BJ) Energy =
-885.731469725
SCF (C6H6) Energy = -885.613869978
SCF (BS2) Energy = -1743.09387950

```
Si  4.04288 -0.15862  0.00305
K   0.29753 -0.12391  0.05239
N  -2.71266  0.16717 -0.23575
N  -0.70925  2.60271 -0.45602
N  -1.34652 -0.81866  2.43758
N  -0.99998 -1.79750 -1.85198
C  -3.03838  1.61016 -0.26794
H  -3.07906  1.97864  0.77094
H  -4.05967  1.78093 -0.68654
C  -2.04619  2.47638 -1.06528
H  -1.90977  2.04547 -2.07366
H  -2.51597  3.47904 -1.22057
C  -3.36347 -0.49024  0.91670
H  -3.38381 -1.57572  0.72491
H  -4.43365 -0.18168  1.00584
C  -2.69212 -0.23499  2.27828
H  -2.59880  0.85367  2.44596
H  -3.38759 -0.60893  3.07029
C  -3.11933 -0.47851 -1.50523
H  -2.86443  0.20969 -2.32864
H  -4.22788 -0.60946 -1.55328
C  -2.46365 -1.84465 -1.78450
H  -2.73617 -2.55981 -0.98589
H  -2.92229 -2.24866 -2.72159
C   0.25324  3.13290 -1.43844
H   1.25561  3.18411 -0.98255
H  -0.02020  4.15056 -1.80327
H   0.31393  2.45540 -2.30642
C  -0.73279  3.47613  0.72609
H  -1.40260  3.06881  1.50159
H  -1.07292  4.51200  0.48757
H   0.28070  3.53882  1.15584
C  -0.70680 -0.28082  3.64943
H  -0.62692  0.81720  3.57619
H   0.31142 -0.69351  3.74650
H  -1.26788 -0.52558  4.58179
C  -1.39694 -2.28637  2.52065
H  -1.80523 -2.71536  1.59144
H  -2.01971 -2.64665  3.37360
H  -0.37594 -2.68222  2.64942
C  -0.39433 -3.13070 -1.72875
H   0.70172 -3.01160 -1.67617
H  -0.64309 -3.80327 -2.58358
H  -0.73881 -3.61648 -0.79866
C  -0.49371 -1.14514 -3.06910
H  -0.93378 -0.13950 -3.18235
H  -0.72516 -1.72657 -3.99313
H   0.60062 -1.02542 -2.96597
C   2.78335 -0.70894 -1.22402
H   2.96560 -1.77588 -1.48067
H   2.91549 -0.13620 -2.16908
C   5.93191 -0.28840 -0.41013
H   6.21400 -1.33638 -0.61728
H   6.57789  0.07782  0.41010
H   6.17373  0.29688 -1.31554
C   3.78855  1.70654  0.45884
H   3.82664  2.34103 -0.44598
H   4.56201  2.07840  1.15477
H   2.80527  1.87179  0.94137
C   3.85045 -1.12092  1.66990
H   4.63408 -0.85420  2.40165
H   2.87137 -0.91062  2.14266
H   3.90781 -2.21227  1.50440
```

A-K
SCF (BP86) Energy = -1462.23698337
Enthalpy 0K = -1461.504466
Enthalpy 298K = -1461.455681
Free Energy 298K = -1461.596050
Lowest Frequency = 6.6289 cm^{-1}
Second Frequency = 8.6284 cm^{-1}
SCF (BP86-D3BJ) Energy =
-1462.41569429
SCF (C6H6) Energy = -1462.24440537
SCF (BS2) Energy = -2319.86891682

```
Si -1.68541  3.06106  2.07921
N  -2.79612 -1.61562 -0.55107
N  -0.41896 -0.98548 -2.42754
N  -1.06063 -2.64343  1.85234
N  -6.13893  0.24891 -0.77045
C  -2.53643 -2.23563 -1.86690
H  -3.47487 -2.57829 -2.36121
H  -1.94349 -3.15068 -1.69315
C  -3.18988 -2.62493  0.45254
H  -3.72222 -2.10204  1.26594
C  -3.74707 -0.48166 -0.60826
H  -3.28978  0.31527 -1.22085
H  -3.84234 -0.06858  0.41226
C  -5.17306 -0.79847 -1.12360
H  -5.15647 -0.98437 -2.22821
H  -5.52183 -1.73669 -0.65267
C  -5.85445  1.51861 -1.44508
H  -4.87578  1.91710 -1.13350
H  -6.61707  2.26146 -1.15742
H  -5.86210  1.43291 -2.56047
C  -7.50436 -0.18998 -1.06256
H  -7.68641 -0.38794 -2.14776
H  -8.21903  0.58475 -0.73610
H  -7.72712 -1.11663 -0.50573
C  -0.04386  2.24120  1.90946
H   0.35458  1.83377  2.85911
H   0.72842  2.89048  1.45637
C  -2.41454  3.51171  0.35323
H  -1.73363  4.18115 -0.20281
H  -3.38925  4.02477  0.43665
H  -2.55884  2.60455 -0.26248
C  -1.76166  4.70477  3.09420
H  -2.78422  5.12442  3.13335
H  -1.09918  5.47016  2.65276
H  -1.42378  4.53810  4.13215
C  -2.94999  1.88825  2.93669
H  -3.09528  0.96032  2.35285
H  -3.93956  2.36261  3.05886
H  -2.59452  1.59583  3.94115
K  -0.22314 -0.23095  0.38320
C  -1.78157 -1.33481 -2.86037
H  -1.78022 -1.84785 -3.85335
H  -2.34308 -0.39460 -3.00499
C   0.10060  0.18148 -3.15755
```

```
H   1.08322  0.45457 -2.73821
H  -0.58248  1.03849 -3.02877
H   0.22045 -0.00549 -4.25070
C   0.51013 -2.11392 -2.58416
H   0.15537 -2.98992 -2.01546
H   1.49400 -1.82431 -2.18180
H   0.62911 -2.42317 -3.65019
C  -2.01435 -3.42615  1.04346
H  -2.44737 -4.26774  1.63965
H  -1.44151 -3.89690  0.22300
C   0.12904 -3.45897  2.14376
H  -0.10802 -4.37727  2.73136
H   0.85633 -2.86393  2.72112
H   0.61131 -3.77015  1.20146
C  -1.66361 -2.18599  3.11525
H  -0.91322 -1.62635  3.69721
H  -2.03272 -3.03181  3.74231
H  -2.50478 -1.50131  2.92411
H  -3.91505 -3.36793  0.04485
O   2.37984 -0.37734 -0.42692
C   3.58114  0.00094 -0.36318
C   4.60831 -0.90280  0.22978
C   5.80015 -0.42728  0.83126
C   4.34439 -2.29492  0.26094
C   6.70287 -1.31925  1.42587
H   5.99910  0.64802  0.85996
C   5.25416 -3.18371  0.84178
H   3.41357 -2.65284 -0.18921
C   6.43969 -2.70009  1.42629
H   7.61242 -0.93447  1.89918
H   5.04334 -4.25878  0.84209
H   7.15016 -3.39529  1.88568
C   3.95245  1.35468 -0.86826
C   5.20770  1.63511 -1.46177
C   2.97630  2.38005 -0.81600
C   5.47724  2.90733 -1.98490
H   5.95841  0.84269 -1.54147
C   3.26183  3.65647 -1.31333
H   2.01068  2.16685 -0.34358
C   4.50974  3.92387 -1.90587
H   6.44423  3.10539 -2.45959
H   2.50883  4.44822 -1.23899
H   4.72533  4.91925 -2.30850
```

TS(A-B)-K

SCF (BP86) Energy = -1462.23166940
Enthalpy 0K = -1461.498313
Enthalpy 298K = -1461.450721
Free Energy 298K = -1461.586496
Lowest Frequency = -117.6720 cm^{-1}
Second Frequency = 6.0995 cm^{-1}
SCF (BP86-D3BJ) Energy = -1462.41539442
SCF (C6H6) Energy = -1462.24079099
SCF (BS2) Energy = -2319.86448991

```
Si -2.13073 -2.14281  2.50504
N   3.64701  0.65618 -0.31330
N   1.78232  0.23006 -2.69565
N   1.97045  2.82914  1.15993
N   5.73989 -2.33885  0.86314
C   3.99919  0.93934 -1.72089
H   5.09073  0.81978 -1.91279
H   3.78074  2.00560 -1.90736
C   4.17535  1.69169  0.59775
H   4.21923  1.25811  1.61243
C   3.98364 -0.71526  0.12809
H   3.39385 -1.42111 -0.48381
H   3.62911 -0.83261  1.16935
C   5.48010 -1.11099  0.10126
H   5.84272 -1.19522 -0.95472
H   6.06808 -0.30158  0.57251
C   5.15415 -3.52263  0.22744
H   4.05769 -3.43186  0.16701
H   5.37634 -4.41267  0.83932
H   5.54731 -3.70668 -0.80311
C   7.17778 -2.53025  1.06574
H   7.74737 -2.66561  0.11395
H   7.34631 -3.42403  1.69031
H   7.60025 -1.65920  1.59546
C  -1.54567 -0.96662  1.21489
H  -1.53201  0.11704  1.37332
H  -1.32151 -1.31757  0.20577
C  -1.83693 -3.93887  1.90618
H  -2.35167 -4.11622  0.94590
H  -2.21762 -4.67779  2.63279
H  -0.76153 -4.13752  1.75448
C  -4.00295 -1.98923  2.92671
H  -4.30923 -2.71863  3.69865
H  -4.61921 -2.16363  2.02805
H  -4.24284 -0.98113  3.30923
C  -1.21377 -1.90481  4.17940
H  -0.13577 -2.11599  4.06790
H  -1.61342 -2.56714  4.96868
H  -1.31499 -0.86382  4.53614
K   0.70489  0.50069 -0.06477
C   3.24466  0.08584 -2.75661
H   3.64850  0.34547 -3.76576
H   3.48456 -0.98125 -2.60003
C   1.09143 -0.87375 -3.38321
H   0.00632 -0.76109 -3.22158
H   1.41233 -1.83902 -2.95470
H   1.29486 -0.89655 -4.47922
C   1.32567  1.51223 -3.25438
H   1.79447  2.35807 -2.72244
H   0.23306  1.58580 -3.12610
H   1.56742  1.61564 -4.33865
C   3.33895  2.98547  0.62968
H   3.91287  3.74715  1.21271
H   3.25081  3.38910 -0.39567
C   1.17587  4.03512  0.86869
H   1.58101  4.94864  1.36321
H   0.13656  3.89080  1.20818
H   1.15624  4.21545 -0.21962
C   1.97454  2.58474  2.61233
H   0.93732  2.47692  2.97013
H   2.45474  3.41405  3.18275
H   2.50723  1.64997  2.85097
H   5.22175  1.98376  0.34725
O  -1.50480  0.42611 -1.56283
C  -2.65020  0.36486 -1.01010
C  -3.10952  1.54537 -0.19824
C  -4.04409  1.47051  0.86322
C  -2.55185  2.80912 -0.50729
C  -4.41502  2.61800  1.57383
H  -4.43053  0.49149  1.16087
C  -2.92558  3.96042  0.20530
H  -1.83536  2.86681 -1.33323
```

```
C  -3.86076  3.87216  1.24802
H  -5.12933  2.53410  2.40017
H  -2.49518  4.93189 -0.06500
H  -4.15359  4.76728  1.80716
C  -3.60834 -0.70820 -1.42204
C  -5.01602 -0.57029 -1.33824
C  -3.09348 -1.86668 -2.05681
C  -5.86714 -1.57604 -1.82017
H  -5.44864  0.34082 -0.91609
C  -3.94212 -2.87066 -2.52934
H  -2.00787 -1.96164 -2.15287
C  -5.33896 -2.73571 -2.40872
H  -6.95235 -1.44510 -1.74420
H  -3.51747 -3.76532 -2.99866
H  -6.00525 -3.52140 -2.78023
```

B-K

SCF (BP86) Energy = -1462.27808008
Enthalpy 0K = -1461.540545
Enthalpy 298K = -1461.494178
Free Energy 298K = -1461.623296
Lowest Frequency = 8.9296 cm^{-1}
Second Frequency = 15.1959 cm^{-1}
SCF (BP86-D3BJ) Energy = -1462.47285544
SCF (C6H6) Energy = -1462.28441099
SCF (BS2) Energy = -2319.90949737

```
Si -3.92801  1.39889 -1.42411
N   3.08095  0.90824  0.46083
N   2.19611 -1.55123  2.34502
N   4.29975 -1.09694 -1.56524
N   1.45370  4.36325  0.02786
C   3.28425  0.69968  1.91280
H   3.40820  1.66538  2.45066
H   4.24226  0.16548  2.04381
C   4.35216  1.08919 -0.26051
H   4.14523  1.65677 -1.18492
C   2.06702  1.94386  0.13870
H   1.08940  1.59026  0.52074
H   1.96153  1.97034 -0.96300
C   2.39273  3.38537  0.59486
H   2.42193  3.45260  1.71152
H   3.40527  3.65513  0.23800
C   0.11960  4.24960  0.62718
H  -0.29516  3.24178  0.47012
H  -0.55982  4.97330  0.14557
H   0.12076  4.45751  1.72602
C   1.96278  5.72619  0.18444
H   2.08688  6.03558  1.25191
H   1.26605  6.43756 -0.29182
H   2.94310  5.82219 -0.31409
C  -2.23103  1.07912 -0.56944
H  -1.43843  1.43742 -1.25603
H  -2.20445  1.79286  0.27749
C  -5.30631  1.70293 -0.14051
H  -5.47709  0.81365  0.48727
H  -6.25656  1.96289 -0.63941
H  -5.03916  2.53892  0.52899
C  -4.49469  0.03999 -2.64189
H  -5.42234  0.35826 -3.15000
H  -4.70413 -0.91303 -2.12955
H  -3.73634 -0.16051 -3.41678
C  -3.70704  3.01147 -2.43537
H  -3.37912  3.84503 -1.79032
H  -4.65305  3.31420 -2.91774
H  -2.95044  2.88950 -3.23028
K   1.55234 -1.31222 -0.43901
C   2.15833 -0.09252  2.60328
H   2.21255  0.11006  3.70110
H   1.17451  0.26924  2.25004
C   0.90675 -2.14473  2.75689
H   0.91300 -3.22802  2.53942
H   0.09907 -1.64998  2.18372
H   0.71302 -2.01931  3.84780
C   3.29736 -2.20357  3.06356
H   4.27352 -1.79986  2.74564
H   3.29390 -3.28492  2.84344
H   3.21873 -2.07807  4.17016
C   5.04430 -0.24087 -0.61525
H   6.06663 -0.01205 -1.00552
H   5.18744 -0.82825  0.31052
C   4.92683 -2.42728 -1.62707
H   5.98336 -2.39343 -1.98296
H   4.35988 -3.07622 -2.31674
H   4.91906 -2.89292 -0.62687
C   4.27195 -0.50826 -2.91411
H   3.72632 -1.17994 -3.59809
H   5.29336 -0.34610 -3.33228
H   3.74719  0.46029 -2.90595
H   5.08962  1.70240  0.30761
O  -0.46415 -0.09570  0.61614
C  -1.71946 -0.27534  0.05799
C  -1.48800 -1.38536 -1.00738
C  -1.11967 -1.08567 -2.34134
C  -1.35682 -2.73714 -0.59510
C  -0.61857 -2.07334 -3.20968
H  -1.21755 -0.05947 -2.70920
C  -0.86271 -3.72818 -1.45736
H  -1.64848 -3.00452  0.42604
C  -0.47661 -3.40237 -2.77290
H  -0.35627 -1.80470 -4.24015
H  -0.79052 -4.76496 -1.10705
H  -0.10364 -4.17613 -3.45261
C  -2.76909 -0.70844  1.12146
C  -3.90198 -1.50101  0.84365
C  -2.58840 -0.24965  2.44087
C  -4.82434 -1.82647  1.85433
H  -4.05908 -1.88386 -0.17107
C  -3.51340 -0.55576  3.45011
H  -1.68957  0.34229  2.64308
C  -4.63639 -1.35075  3.16207
H  -5.68987 -2.45640  1.61808
H  -3.35580 -0.18031  4.46820
H  -5.35497 -1.60212  3.95005
```

TS(B-C)-K

SCF (BP86) Energy = -1462.27041366
Enthalpy 0K = -1461.533159
Enthalpy 298K = -1461.487590
Free Energy 298K = -1461.613624
Lowest Frequency = -64.2175 cm^{-1}
Second Frequency = 11.6556 cm^{-1}
SCF (BP86-D3BJ) Energy = -1462.46665463
SCF (C6H6) Energy = -1462.27674295
SCF (BS2) Energy = -2319.90186533

```
Si -3.95614  1.32450 -1.28322
N   2.95817  1.25866  0.41614
N   2.21468 -1.11287  2.47382
N   4.56129 -0.69314 -1.35667
N   0.58688  4.23338 -0.15039
C   3.08871  1.19700  1.88805
H   3.07888  2.21167  2.34479
H   4.08603  0.78098  2.11778
C   4.23547  1.57315 -0.24431
H   4.01315  2.03946 -1.22023
C   1.81674  2.07726 -0.06310
H   0.88018  1.57935  0.25608
H   1.80911  2.01288 -1.16767
C   1.81739  3.57635  0.31265
H   1.95809  3.71455  1.41399
H   2.67492  4.07774 -0.17519
C  -0.56584  3.87254  0.68384
H  -0.72027  2.78108  0.69299
H  -1.47623  4.33852  0.26990
H  -0.45436  4.21945  1.74163
C   0.74640  5.68714 -0.18995
H   0.94832  6.14068  0.81231
H  -0.17423  6.14896 -0.58657
H   1.58042  5.95724 -0.86083
C  -2.20033  0.53962 -1.26610
H  -2.06328  0.24482 -2.32434
H  -1.51465  1.39488 -1.12247
C  -4.55647  2.00502  0.39288
H  -4.77119  1.19610  1.10867
H  -5.47759  2.59801  0.25133
H  -3.79907  2.66492  0.84946
C  -5.29989  0.18124 -2.01951
H  -6.20998  0.76517 -2.24451
H  -5.57920 -0.62386 -1.32217
H  -4.96065 -0.28336 -2.96180
C  -3.78330  2.80920 -2.48477
H  -3.02913  3.53115 -2.12613
H  -4.74107  3.34838 -2.59126
H  -3.47320  2.47936 -3.49189
K   1.78416 -1.21961 -0.34537
C   2.00857  0.34977  2.58648
H   1.96471  0.65570  3.66025
H   1.01933  0.56961  2.14463
C   0.96411 -1.80513  2.84789
H   1.10074 -2.89627  2.74375
H   0.16024 -1.46385  2.16789
H   0.66513 -1.59920  3.90216
C   3.31697 -1.57351  3.32693
H   4.26703 -1.09153  3.04090
H   3.44495 -2.66332  3.21065
H   3.13687 -1.36168  4.40807
C   5.12729  0.33399 -0.45429
H   6.12702  0.67636 -0.81926
H   5.30116 -0.15170  0.52376
C   5.37281 -1.91873 -1.28082
H   6.43065 -1.76018 -1.59697
H   4.93903 -2.69557 -1.93396
H   5.38035 -2.30043 -0.24563
C   4.51849 -0.22185 -2.75041
H   4.11189 -1.01813 -3.39647
H   5.52578  0.05701 -3.14044
H   3.85933  0.65577 -2.84472
H   4.83921  2.32478  0.31526
O  -0.47742 -0.14463  0.35451
C  -1.62971 -0.57934 -0.27984
C  -1.15383 -1.85819 -1.05477
C  -0.61040 -1.80968 -2.36195
C  -0.98038 -3.07385 -0.34230
C   0.08607 -2.89934 -2.91880
H  -0.71973 -0.90064 -2.96297
C  -0.29068 -4.16537 -0.89107
H  -1.40021 -3.15443  0.66553
C   0.26052 -4.08542 -2.18516
H   0.47612 -2.82452 -3.94130
H  -0.19371 -5.09132 -0.31157
H   0.78534 -4.94138 -2.62301
C  -2.73527 -0.97273  0.74160
C  -3.82053 -1.81430  0.41849
C  -2.65343 -0.46577  2.05089
C  -4.79946 -2.13174  1.37518
H  -3.88949 -2.24144 -0.58889
C  -3.63447 -0.76890  3.00923
H  -1.79142  0.16821  2.28221
C  -4.71326 -1.60524  2.67564
H  -5.62801 -2.79762  1.10702
H  -3.55606 -0.35541  4.02180
H  -5.47592 -1.85188  3.42263
```

C-K

SCF (BP86) Energy = -1462.27900547
Enthalpy 0K = -1461.541955
Enthalpy 298K = -1461.495325
Free Energy 298K = -1461.626023
Lowest Frequency = 7.9617 cm^{-1}
Second Frequency = 16.4544 cm^{-1}
SCF (BP86-D3BJ) Energy = -1462.47510049
SCF (C6H6) Energy = -1462.28440582
SCF (BS2) Energy = -2319.91082960

```
Si  3.75416  0.53858 -1.81719
C   3.03424 -1.07514 -1.06844
H   2.58154 -1.59773 -1.93414
H   3.88775 -1.70652 -0.74756
C   4.76703  1.57621 -0.57851
H   4.13392  1.97611  0.22951
H   5.24907  2.42474 -1.09621
H   5.56010  0.97235 -0.10616
C   2.37187  1.61601 -2.56467
H   2.74271  2.60101 -2.89728
H   1.59672  1.74210 -1.79209
H   1.90314  1.11429 -3.42890
C   4.94412 -0.00938 -3.21604
H   5.38534  0.86497 -3.72625
H   4.42127 -0.61218 -3.97922
H   5.77553 -0.61919 -2.82121
K  -1.47537 -1.31179  0.08179
O   0.82354 -0.27981 -0.37276
C   1.94186 -0.96455  0.05587
C   2.61061 -0.32478  1.31095
C   3.75694 -0.86727  1.93014
C   2.03690  0.82990  1.87010
C   4.32179 -0.25860  3.06174
H   4.20742 -1.78399  1.53096
C   2.59680  1.44625  3.00292
H   1.13940  1.21631  1.37557
C   3.74543  0.90492  3.60213
H   5.21376 -0.69433  3.52689
```

```
H   2.13515  2.34972  3.41902
H   4.18758  1.38075  4.48457
C   1.40915 -2.38638  0.46890
C   0.88223 -2.60589  1.76783
C   1.20214 -3.41296 -0.48378
C   0.15919 -3.76833  2.08722
H   1.05408 -1.84889  2.54116
C   0.48219 -4.57860 -0.17127
H   1.61466 -3.30080 -1.49164
C  -0.05703 -4.76267  1.11508
H  -0.21569 -3.90962  3.10855
H   0.35572 -5.35731 -0.93316
H  -0.60405 -5.67835  1.36462
N  -2.78060  1.21978 -0.26198
N  -1.96742 -0.72760 -2.68247
N  -4.19250 -1.10841  1.24256
N  -0.98205  4.38284  0.97327
C  -3.00397  1.37325 -1.71775
H  -3.11516  2.44236 -2.00546
H  -3.97165  0.89897 -1.96036
C  -4.03711  1.32385  0.50241
H  -3.78673  1.62997  1.53334
C  -1.70454  2.09119  0.27254
H  -0.75475  1.79050 -0.20493
H  -1.58616  1.85274  1.34612
C  -1.94204  3.61696  0.16673
H  -1.92990  3.94726 -0.90224
H  -2.95288  3.84563  0.55462
C   0.36806  4.34936  0.39957
H   0.74735  3.31711  0.34599
H   1.05391  4.92119  1.04694
H   0.41422  4.79054 -0.62628
C  -1.42746  5.76837  1.13204
H  -1.49917  6.32694  0.16623
H  -0.71947  6.31105  1.78178
H  -2.42036  5.79187  1.61398
C  -1.90197  0.74740 -2.59199
H  -1.95551  1.20724 -3.60857
H  -0.91027  1.00582 -2.18191
C  -0.66540 -1.24697 -3.15064
H  -0.71644 -2.34790 -3.22280
H   0.11077 -0.96604 -2.40945
H  -0.38907 -0.85246 -4.15634
C  -3.04204 -1.16262 -3.58328
H  -4.02282 -0.80077 -3.23039
H  -3.08028 -2.26492 -3.61031
H  -2.89803 -0.79675 -4.62796
C  -4.84708  0.01401  0.53619
H  -5.84708  0.23584  0.98402
H  -5.03772 -0.31925 -0.50072
C  -4.94330 -2.35077  0.99715
H  -5.99353 -2.30382  1.36948
H  -4.44360 -3.19526  1.50185
H  -4.97463 -2.56317 -0.08514
C  -4.11434 -0.86374  2.69224
H  -3.64071 -1.72860  3.18629
H  -5.11708 -0.70837  3.15545
H  -3.49646  0.02289  2.90599
H  -4.71416  2.11790  0.11052
```

TS(C-D)-K

SCF (BP86) Energy = -1462.24640532
Enthalpy 0K = -1461.511935
Enthalpy 298K = -1461.465291
Free Energy 298K = -1461.595599
Lowest Frequency = -364.1479 cm^{-1}
Second Frequency = 11.2397 cm^{-1}
SCF (BP86-D3BJ) Energy = -1462.44587920
SCF (C6H6) Energy = -1462.25475624
SCF (BS2) Energy = -2319.87922951

```
Si -1.37269 -2.78538 -1.21939
C  -3.82003 -1.64785 -1.22881
H  -4.11656 -1.35415 -2.24001
H  -4.50877 -2.32019 -0.70973
C  -2.19273 -4.27906 -0.35069
H  -2.43326 -4.02621  0.69591
H  -1.51743 -5.15491 -0.34170
H  -3.13470 -4.55828 -0.84743
C   0.53275 -3.10813 -0.97843
H   0.82323 -4.09159 -1.39283
H   0.81802 -3.09735  0.08919
H   1.13956 -2.34378 -1.50309
C  -1.53125 -2.90836 -3.12871
H  -0.88845 -3.72437 -3.51014
H  -1.19057 -1.97667 -3.61625
H  -2.56918 -3.09397 -3.44192
K   0.15391  0.76567 -0.17065
O  -1.40671 -1.25253 -0.43534
C  -3.02607 -0.73898 -0.43771
C  -3.40826 -0.71907  1.04596
C  -4.73517 -0.37383  1.39247
C  -2.51568 -1.03364  2.09117
C  -5.15043 -0.33115  2.73168
H  -5.44116 -0.15402  0.58458
C  -2.93461 -1.01247  3.43506
H  -1.50143 -1.33746  1.81836
C  -4.25066 -0.65344  3.76301
H  -6.18507 -0.06021  2.97084
H  -2.23076 -1.29530  4.22712
H  -4.57800 -0.63860  4.80846
C  -2.86231  0.64956 -1.04053
C  -3.00138  1.84538 -0.28974
C  -2.52262  0.79009 -2.41494
C  -2.83724  3.11126 -0.88386
H  -3.27310  1.78172  0.76894
C  -2.36687  2.04910 -3.00730
H  -2.39625 -0.11352 -3.01783
C  -2.51631  3.22788 -2.24561
H  -2.97627  4.01231 -0.27438
H  -2.13567  2.11627 -4.07737
H  -2.40568  4.21249 -2.71239
N   3.00427  0.89926  0.54330
N   1.91236  1.70685 -2.25736
N   0.78631  2.21960  2.27508
N   4.98407 -2.34630  1.00522
C   3.70022  1.73343 -0.45883
H   4.80835  1.64903 -0.37410
H   3.46634  2.78873 -0.23156
C   3.17946  1.44578  1.90572
H   3.03590  0.62005  2.62385
C   3.34075 -0.54172  0.46510
H   2.98359 -0.92659 -0.50621
H   2.74874 -1.06572  1.23774
C   4.82501 -0.92489  0.67771
H   5.43303 -0.63764 -0.21856
H   5.22641 -0.34384  1.52939
```

```
C   4.59320 -3.22069 -0.10608
H   3.52313 -3.10403 -0.34317
H   4.75076 -4.27170  0.18796
H   5.18406 -3.03306 -1.03694
C   6.35989 -2.62851  1.41910
H   7.11399 -2.43853  0.61632
H   6.44503 -3.68687  1.71874
H   6.62492 -2.00431  2.29005
C   3.32374  1.43732 -1.92053
H   4.01457  2.02385 -2.57357
H   3.52416  0.37430 -2.14285
C   1.55046  1.05327 -3.52756
H   0.48206  1.22812 -3.73623
H   1.71039 -0.03498 -3.44852
H   2.14337  1.43289 -4.39195
C   1.64593  3.15135 -2.35641
H   1.88491  3.65672 -1.40578
H   0.57495  3.31367 -2.56528
H   2.24102  3.64025 -3.16339
C   2.21343  2.59555  2.24801
H   2.53487  3.03014  3.22657
H   2.32680  3.40423  1.50187
C  -0.04823  3.42991  2.36244
H   0.13542  4.01404  3.29428
H  -1.11362  3.14743  2.34007
H   0.15203  4.08854  1.50007
C   0.47435  1.33182  3.40925
H  -0.59364  1.05891  3.38159
H   0.69210  1.80769  4.39400
H   1.05548  0.39793  3.34430
H   4.21426  1.82074  2.08044
```

D-K

SCF (BP86) Energy = -1462.30929346
Enthalpy 0K = -1461.572523
Enthalpy 298K = -1461.525115
Free Energy 298K = -1461.657422
Lowest Frequency = 13.2258 cm^{-1}
Second Frequency = 17.5068 cm^{-1}
SCF (BP86-D3BJ) Energy = -1462.50593145
SCF (C6H6) Energy = -1462.31603355
SCF (BS2) Energy = -2319.94492543

```
Si -0.70714 -1.03706  3.10142
N   2.53474 -0.55171 -0.38587
N   2.10439  1.27419 -2.76352
N   2.98525  1.99246  1.45219
N   1.11350 -4.08999 -0.75573
C   3.46581 -0.48415 -1.51683
H   3.96881 -1.45725 -1.73029
H   4.27809  0.21684 -1.25976
C   3.16722 -0.50883  0.94886
H   2.34941 -0.69418  1.67034
C   1.54948 -1.65572 -0.48102
H   1.06425 -1.59610 -1.47246
H   0.77183 -1.44833  0.28479
C   2.08446 -3.09313 -0.26626
H   3.08665 -3.23932 -0.74064
H   2.22219 -3.25931  0.81589
C   1.24998 -4.31388 -2.19516
H   1.13923 -3.36363 -2.74425
H   0.45661 -4.99633 -2.54509
H   2.23701 -4.75865 -2.47578
C   1.21641 -5.35770 -0.02781
H   2.20749 -5.85908 -0.14889
H   0.44080 -6.05454 -0.39028
H   1.04258 -5.18137  1.04642
C  -5.10480  1.19321  0.51797
H  -5.81977  0.37405  0.63660
H  -5.36374  2.15627  0.96949
C  -0.08031 -0.32476  4.77788
H   1.02213 -0.35276  4.84696
H  -0.47994 -0.89650  5.63535
H  -0.39543  0.72686  4.90557
C  -0.17744 -2.88936  3.09208
H  -0.61925 -3.45392  3.93329
H   0.92152 -2.98493  3.16904
H  -0.49286 -3.37843  2.15305
C  -2.62439 -1.02996  3.23311
H  -3.01066  0.00263  3.30496
H  -2.97121 -1.57934  4.12747
H  -3.09248 -1.49604  2.34885
K   0.51294  1.36572 -0.05541
C   2.80370 -0.02584 -2.83071
H   3.59463 -0.01302 -3.62155
H   2.06032 -0.77757 -3.15103
C   1.37872  1.50362 -4.02086
H   0.83090  2.46002 -3.96625
H   0.64595  0.69537 -4.18329
H   2.05043  1.54573 -4.91118
C   3.04703  2.37596 -2.52444
H   3.51119  2.27125 -1.53106
H   2.50275  3.33577 -2.54143
H   3.85637  2.42782 -3.29179
C   3.86921  0.81742  1.29713
H   4.45384  0.64371  2.23424
H   4.61674  1.06569  0.52031
C   3.80089  3.21528  1.48745
H   4.52506  3.22966  2.33648
H   3.14558  4.09713  1.59031
H   4.37420  3.31972  0.54947
C   2.18926  1.88902  2.69486
H   1.62382  2.82704  2.83726
H   2.83509  1.74428  3.59261
H   1.45664  1.05997  2.62241
H   3.92616 -1.31517  1.08607
O  -0.12864 -0.21533  1.79896
C  -3.94787  1.01518 -0.16404
C  -2.96787  2.13225 -0.29711
C  -2.66955  2.98608  0.79097
C  -2.34722  2.39360 -1.54306
C  -1.80451  4.08046  0.63300
H  -3.10934  2.76934  1.76917
C  -1.49375  3.49743 -1.70529
H  -2.56832  1.73926 -2.39295
C  -1.21721  4.34695 -0.61736
H  -1.58980  4.72876  1.48935
H  -1.05878  3.70658 -2.68898
H  -0.55949  5.21343 -0.74543
C  -3.64559 -0.28198 -0.84482
C  -4.56926 -0.83251 -1.76062
C  -2.45260 -0.98984 -0.55912
C  -4.31147 -2.06417 -2.38266
H  -5.48748 -0.27930 -1.98712
C  -2.20679 -2.22950 -1.17356
H  -1.74457 -0.61674  0.20147
C  -3.13022 -2.76402 -2.08996
```

```
H  -5.03519 -2.47562 -3.09496
H  -1.29696 -2.78833 -0.91845
H  -2.93279 -3.72975 -2.56823
```

$K(CH_2SiMe_3)$ + benzophenone

$K(CH_2SiMe_3)$

SCF (BP86) Energy = -191.289700953
Enthalpy 0K = -191.158534
Enthalpy 298K = -191.146127
Free Energy 298K = -191.196659
Lowest Frequency = 19.4976 cm^{-1}
Second Frequency = 49.6112 cm^{-1}
SCF (BP86-D3BJ) Energy = -191.316401573
SCF (C6H6) Energy = -191.302662111
SCF (BS2) Energy = -1048.60894096

Si	0.98844	0.00029	0.08044
C	-0.39799	0.00352	1.30938
H	-0.37827	-0.89010	1.96346
H	-0.37751	0.89990	1.95962
C	2.77892	-0.00376	0.79777
H	2.94921	0.88531	1.42995
H	3.54759	-0.00620	0.00337
H	2.94475	-0.89289	1.43107
C	0.89119	1.54590	-1.06385
H	1.75236	1.60041	-1.75294
H	0.88184	2.47814	-0.47111
H	-0.02491	1.53902	-1.68470
C	0.88454	-1.54553	-1.06318
H	0.86901	-2.47739	-0.46992
H	1.74649	-1.60529	-1.75086
H	-0.03047	-1.53386	-1.68562
K	-2.77149	-0.00010	0.00110

^{K}**A**

SCF (BP86) Energy = -767.934613840
Enthalpy 0K = -767.617653
Enthalpy 298K = -767.591768
Free Energy 298K = -767.680383
Lowest Frequency = 6.3797 cm^{-1}
Second Frequency = 8.9495 cm^{-1}
SCF (BP86-D3BJ) Energy = -768.014672082
SCF (C6H6) Energy = -767.943922904
SCF (BS2) Energy = -1625.39788758

Si	5.08747	-0.50408	-0.08256
C	3.88373	-0.20758	-1.45449
H	3.87047	0.83218	-1.83194
H	3.99008	-0.90137	-2.30916
C	6.94757	-0.19915	-0.49186
H	7.27809	-0.85148	-1.31878
H	7.59957	-0.39715	0.37862
H	7.11352	0.84508	-0.80931
C	4.97586	-2.32128	0.53734
H	5.73599	-2.53541	1.30905
H	5.13652	-3.03280	-0.29212
H	3.98742	-2.54624	0.97973
C	4.69377	0.63177	1.42164
H	4.65757	1.69349	1.11830
H	5.45791	0.53975	2.21344
H	3.71824	0.38274	1.88055
K	1.31680	-0.60033	-0.43606
O	-1.08178	-0.97745	0.59497
C	-2.07898	-0.24647	0.33482
C	-3.42296	-0.85412	0.16051
C	-4.62730	-0.12292	0.31788
C	-3.50158	-2.24487	-0.10347
C	-5.86765	-0.76148	0.18878
H	-4.58743	0.94003	0.57358
C	-4.74116	-2.87495	-0.24412
H	-2.56716	-2.80681	-0.19238
C	-5.93042	-2.13531	-0.10192
H	-6.79000	-0.18695	0.32380
H	-4.78629	-3.94744	-0.46106
H	-6.90133	-2.63043	-0.20698
C	-1.86415	1.22450	0.18781
C	-0.82558	1.83150	0.94027
C	-2.56395	2.01443	-0.75771
C	-0.51605	3.18722	0.77281
H	-0.30101	1.22664	1.68813
C	-2.23798	3.36627	-0.93617
H	-3.34197	1.55490	-1.37502
C	-1.21993	3.95943	-0.16869
H	0.27137	3.64578	1.38020
H	-2.77668	3.95914	-1.68271
H	-0.97388	5.01725	-0.30550

^{K}**TS(A-B)**

SCF (BP86) Energy = -767.916683789
Enthalpy 0K = -767.599003
Enthalpy 298K = -767.574398
Free Energy 298K = -767.654668
Lowest Frequency = -193.0267 cm^{-1}
Second Frequency = 15.0936 cm^{-1}
SCF (BP86-D3BJ) Energy = -768.006094553
SCF (C6H6) Energy = -767.931708418
SCF (BS2) Energy = -1625.38252874

Si	-2.01972	-2.04376	-0.51610
C	-0.54598	-1.45793	0.43441
H	0.47268	-1.65328	0.08125
H	-0.67640	-0.97181	1.40326
C	-2.00912	-1.39029	-2.32125
H	-2.04215	-0.28744	-2.33503
H	-2.87802	-1.76457	-2.89156
H	-1.09874	-1.71001	-2.85896
C	-3.60726	-1.41281	0.34228
H	-4.51173	-1.70080	-0.22113
H	-3.59253	-0.31273	0.42423
H	-3.70249	-1.82610	1.36164
C	-2.13512	-3.95914	-0.65448
H	-1.22663	-4.37550	-1.12523
H	-3.00213	-4.27352	-1.26378
H	-2.23466	-4.41972	0.34385
K	1.64186	-1.73354	2.37525
O	0.97815	0.71252	2.02581
C	0.74071	0.93656	0.79347
C	-0.40233	1.82462	0.41654
C	-0.46154	2.55302	-0.79590
C	-1.41604	2.04890	1.38072
C	-1.52764	3.42756	-1.05481
H	0.34181	2.45530	-1.53121
C	-2.47962	2.91659	1.11839
H	-1.34260	1.51913	2.33483
C	-2.54713	3.60752	-0.10689
H	-1.55324	3.98129	-1.99981
H	-3.26010	3.06340	1.87327
H	-3.37949	4.28878	-0.31243

C   1.82397 0.58548 -0.19628
C   3.15708 0.56571 0.28438
C   1.60058 0.24624 -1.55302
C   4.22966 0.23044 -0.56058
H   3.33356 0.86611 1.32369
C   2.66955 -0.08513 -2.39374
H   0.57208 0.18935 -1.92110
C   3.99141 -0.09476 -1.90383
H   5.25410 0.24046 -0.17068
H   2.47207 -0.35579 -3.43662
H   4.82303 -0.35818 -2.56576

[K]**B**

SCF (BP86) Energy = -767.955412262
Enthalpy 0K = -767.634418
Enthalpy 298K = -767.610573
Free Energy 298K = -767.687378
Lowest Frequency = 24.1415 cm^{-1}
Second Frequency = 37.4229 cm^{-1}
SCF (BP86-D3BJ) Energy =
-768.049664833
SCF (C6H6) Energy = -767.970369053
SCF (BS2) Energy = -1625.42679906

Si 2.11802 -1.90817 -0.15230
C   0.83020 -0.92493 -1.18998
H   1.40150 -0.47079 -2.02322
H   0.18160 -1.67187 -1.68995
C   1.49002 -2.48055 1.56049
H   1.28123 -1.63512 2.23654
H   2.25093 -3.11708 2.04598
H   0.56172 -3.07036 1.47645
C   3.75494 -0.95159 0.06002
H   4.50306 -1.56923 0.58760
H   3.61284 -0.01826 0.62762
H   4.17621 -0.68169 -0.92395
C   2.50407 -3.48780 -1.16655
H   1.60718 -4.12063 -1.28670
H   3.28121 -4.09949 -0.67548
H   2.86847 -3.23268 -2.17686
K -3.33139 0.51715 -1.55089
O -0.92220 0.64645 -1.74961
C -0.12473 0.23317 -0.70438
C   0.75476 1.39101 -0.15344
C   1.31326 1.41192 1.14095
C   1.00531 2.48234 -1.00581
C   2.10631 2.49105 1.56899
H   1.11131 0.58427 1.83105
C   1.81087 3.55446 -0.59139
H   0.53264 2.45258 -1.99341
C   2.36339 3.56522 0.70077
H   2.52015 2.49415 2.58414
H   2.00262 4.39053 -1.27461
H   2.98308 4.40596 1.03209
C -1.17809 -0.22871 0.34418
C -1.84126 0.73477 1.15245
C -1.75995 -1.52214 0.28661
C -3.04608 0.44228 1.81271
H -1.40041 1.73329 1.24440
C -2.96722 -1.81973 0.94348
H -1.26920 -2.30365 -0.30231
C -3.63422 -0.83435 1.69786
H -3.52428 1.21022 2.43311
H -3.38313 -2.83264 0.87941
H -4.56655 -1.06914 2.22231

[K]**TS(B-C)**

SCF (BP86) Energy = -767.947066721
Enthalpy 0K = -767.626603
Enthalpy 298K = -767.603433
Free Energy 298K = -767.679548
Lowest Frequency = -43.1632 cm^{-1}
Second Frequency = 9.6085 cm^{-1}
SCF (BP86-D3BJ) Energy =
-768.041808281
SCF (C6H6) Energy = -767.962463936
SCF (BS2) Energy = -1625.41624279

Si -2.72162 -1.23233 -0.16409
C -0.92219 -1.34445 -0.81982
H -0.58408 -2.31689 -0.40965
H -0.98170 -1.50194 -1.91307
C -2.80055 -0.89361 1.71405
H -2.22176 -1.64738 2.27620
H -3.84298 -0.93915 2.07538
H -2.39482 0.09976 1.96484
C -3.46162 -2.97133 -0.47887
H -4.50915 -3.02845 -0.13411
H -2.89074 -3.75381 0.05040
H -3.44918 -3.22154 -1.55400
C -3.85931 0.00683 -1.07867
H -3.70906 1.04564 -0.74455
H -4.91681 -0.25246 -0.89360
H -3.69270 -0.02987 -2.16914
K   1.81748 2.36179 -1.44507
O   0.52885 0.32984 -1.88175
C   0.25832 -0.26978 -0.67845
C   1.49593 -1.05442 -0.12141
C   1.49969 -1.75658 1.10446
C   2.66544 -1.08435 -0.90182
C   2.64739 -2.43365 1.54696
H   0.59234 -1.77496 1.72097
C   3.81506 -1.77047 -0.47254
H   2.60077 -0.59094 -1.88017
C   3.81456 -2.43907 0.76170
H   2.63094 -2.96499 2.50571
H   4.70844 -1.79707 -1.10869
H   4.70710 -2.97429 1.10405
C -0.09303 0.91712 0.29828
C   0.62112 1.27773 1.46408
C -1.04252 1.85449 -0.18966
C   0.40421 2.51723 2.10687
H   1.36247 0.58732 1.87897
C -1.28400 3.07171 0.46014
H -1.55006 1.61402 -1.12846
C -0.54748 3.42031 1.61384
H   0.98241 2.76622 3.00492
H -2.04266 3.75902 0.06670
H -0.72522 4.37533 2.12003

[K]**C**

SCF (BP86) Energy = -767.960907417
Enthalpy 0K = -767.640031
Enthalpy 298K = -767.616205
Free Energy 298K = -767.692949
Lowest Frequency = 25.2193 cm^{-1}
Second Frequency = 38.9930 cm^{-1}
SCF (BP86-D3BJ) Energy =

-768.056224701
SCF (C6H6) Energy = -767.973954023
SCF (BS2) Energy = -1625.42883637

```
Si -1.94752 -1.99118  0.25973
C  -0.37160 -1.27489  1.08636
H  -0.56834 -0.88698  2.10365
H   0.28790 -2.14981  1.24206
C  -1.52869 -3.22894 -1.12553
H  -0.96491 -2.70651 -1.91304
H  -2.43944 -3.68015 -1.55794
H  -0.90142 -4.05028 -0.73744
C  -3.27199 -0.74131 -0.37780
H  -4.28981 -1.14394 -0.22958
H  -3.13495 -0.57330 -1.46050
H  -3.22089  0.22519  0.15425
C  -2.83992 -2.98276  1.64620
H  -2.16697 -3.73669  2.09124
H  -3.72405 -3.51688  1.25477
H  -3.18416 -2.32598  2.46521
K  -1.44136  1.72920 -1.86202
O  -0.26837 -0.28566 -1.12453
C   0.33087 -0.24663  0.12681
C   1.84809 -0.57564  0.07537
C   2.67490 -0.49944  1.21600
C   2.41636 -0.98448 -1.14263
C   4.03701 -0.82709  1.13804
H   2.24694 -0.17636  2.17321
C   3.77881 -1.31947 -1.22497
H   1.74423 -1.03368 -2.00610
C   4.59500 -1.24062 -0.08521
H   4.66678 -0.76048  2.03287
H   4.20576 -1.64321 -2.18183
H   5.65815 -1.49879 -0.14615
C   0.14677  1.23410  0.59082
C   0.98199  2.23659  0.02885
C  -0.99113  1.67699  1.30820
C   0.66620  3.60065  0.12774
H   1.88814  1.92223 -0.50099
C  -1.31551  3.04353  1.40863
H  -1.64131  0.94158  1.79292
C  -0.50028  4.01663  0.80335
H   1.34075  4.34744 -0.30841
H  -2.20229  3.34929  1.97677
H  -0.74337  5.08083  0.89207
```

[K]**TS(C-D)**
SCF (BP86) Energy = -767.931488117
Enthalpy 0K = -767.612714
Enthalpy 298K = -767.589068
Free Energy 298K = -767.665138
Lowest Frequency = -384.0267 cm^{-1}
Second Frequency = 26.7801 cm^{-1}
SCF (BP86-D3BJ) Energy =
-768.029923625
SCF (C6H6) Energy = -767.945655231
SCF (BS2) Energy = -1625.39895682

```
Si  1.08017 -2.24151 -0.16979
C  -0.18848 -0.81259 -1.93006
H   0.50065 -0.44814 -2.69844
H  -1.03371 -1.39350 -2.30956
C  -0.14413 -3.66465 -0.52898
H  -1.14159 -3.40955 -0.13293
H   0.18089 -4.60709 -0.05133
H  -0.25200 -3.83115 -1.61225
C   1.97927 -2.84100  1.46316
H   2.43608 -3.83688  1.31208
H   1.28326 -2.92393  2.31720
H   2.81101 -2.17096  1.76947
C   2.57207 -2.22832 -1.38554
H   2.24481 -2.35115 -2.42903
H   3.28255 -3.03809 -1.13365
H   3.13398 -1.27747 -1.32565
K   1.88994  0.80964  1.82992
O   0.48295 -0.77876  0.54007
C  -0.40104 -0.02705 -0.74204
C  -1.84401 -0.00994 -0.23790
C  -2.84702  0.46960 -1.11083
C  -2.23004 -0.43185  1.05030
C  -4.18957  0.52745 -0.70910
H  -2.55518  0.78527 -2.11814
C  -3.57754 -0.38816  1.45021
H  -1.45344 -0.82290  1.71264
C  -4.56232  0.09502  0.57542
H  -4.94921  0.90107 -1.40491
H  -3.85803 -0.74153  2.44932
H  -5.61218  0.12781  0.88671
C   0.28414  1.32919 -0.66311
C  -0.26001  2.40552  0.08428
C   1.56849  1.52889 -1.23823
C   0.45288  3.60486  0.27369
H  -1.25558  2.29356  0.52506
C   2.28087  2.72157 -1.04900
H   2.01345  0.72218 -1.82794
C   1.73619  3.76940 -0.27584
H  -0.00493  4.41798  0.84947
H   3.26689  2.84071 -1.51338
H   2.28907  4.70413 -0.13538
```

[K]**D**
SCF (BP86) Energy = -767.996638256
Enthalpy 0K = -767.676739
Enthalpy 298K = -767.651592
Free Energy 298K = -767.736409
Lowest Frequency = 8.0374 cm^{-1}
Second Frequency = 10.0700 cm^{-1}
SCF (BP86-D3BJ) Energy =
-768.081410881
SCF (C6H6) Energy = -768.010133917
SCF (BS2) Energy = -1625.46907715

```
Si -3.36310 -0.45579  0.20180
C   2.86049 -0.26182  2.33502
H   3.15304  0.64091  2.88055
H   3.02939 -1.22077  2.83267
C  -4.21348  1.23290  0.65307
H  -3.61440  1.80717  1.38814
H  -5.21122  1.08817  1.10605
H  -4.36280  1.86797 -0.24300
C  -3.32034 -1.44497  1.84533
H  -4.33181 -1.58307  2.26878
H  -2.70564 -0.92845  2.60436
H  -2.88058 -2.44566  1.69093
C  -4.60210 -1.34874 -0.95914
H  -4.75035 -0.77496 -1.89161
H  -5.59170 -1.48730 -0.48703
H  -4.22161 -2.34590 -1.24190
```

```
K  -1.00695  2.00572 -0.70538
O  -1.89450 -0.20428 -0.47119
C   2.33125 -0.21301  1.08607
C   2.06822 -1.47068  0.32030
C   3.08587 -2.44922  0.22925
C   0.81748 -1.71611 -0.29550
C   2.86511 -3.65114 -0.45876
H   4.06000 -2.24837  0.68907
C   0.60640 -2.92568 -0.97696
H  -0.03880 -1.01914 -0.22142
C   1.62159 -3.89222 -1.06594
H   3.66662 -4.39492 -0.52564
H  -0.37467 -3.10351 -1.42957
H   1.44635 -4.82992 -1.60476
C   2.08884  1.10264  0.42455
C   2.24836  1.24518 -0.97705
C   1.75011  2.25991  1.17155
C   2.12205  2.49931 -1.59821
H   2.49570  0.36064 -1.57168
C   1.62381  3.51452  0.55237
H   1.58350  2.16640  2.24965
C   1.81535  3.64418 -0.83741
H   2.28386  2.58572 -2.67842
H   1.38275  4.39549  1.15785
H   1.73982  4.62573 -1.31770
```

3M Clusters and Conformers

1Li (κ^4)
SCF (BP86) Energy = -864.816498657
Enthalpy 0K = -864.266747
Enthalpy 298K = -864.233465
Free Energy 298K = -864.328771
Lowest Frequency = 16.2568 cm^{-1}
Second Frequency = 33.1197 cm^{-1}
SCF (BP86-D3BJ) Energy = -864.945854349
SCF (Bnz) Energy = -864.821503005
SCF (BS2) Energy = -1150.65380933

```
Si  3.54847 -0.28664 -0.07411
Li -0.16949 -0.09885 -0.02685
N  -2.67624  0.37881  0.11730
N  -0.58606  1.40993 -1.74315
N  -0.47645  0.81905  2.03829
N  -1.42029 -2.28389 -0.33322
C  -2.87943  1.56359 -0.73667
H  -2.60584  2.45899 -0.15387
H  -3.95022  1.69912 -1.01812
C  -2.03031  1.51671 -2.01797
H  -2.31894  0.63465 -2.61833
H  -2.27302  2.40964 -2.64287
C  -2.93312  0.65695  1.54176
H  -3.05341 -0.31013  2.05848
H  -3.89131  1.20733  1.69826
C  -1.79666  1.44825  2.21143
H  -1.73775  2.45962  1.76900
H  -2.05626  1.59812  3.28702
C  -3.43738 -0.78719 -0.36665
H  -3.46194 -0.74303 -1.46869
H  -4.50340 -0.75700 -0.03797
C  -2.82620 -2.12870  0.07036
H  -2.86322 -2.21113  1.17226
H  -3.47218 -2.95297 -0.32050
C   0.14949  0.97278 -2.94323
H   1.20799  0.82912 -2.67784
H   0.07660  1.70719 -3.77817
H  -0.24717  0.00536 -3.29208
C  -0.02622  2.69101 -1.28011
H  -0.54607  3.03420 -0.37112
H  -0.11294  3.49112 -2.05183
H   1.03731  2.54823 -1.03187
C   0.60620  1.76585  2.35596
H   0.50206  2.67325  1.73877
H   1.56987  1.29370  2.10967
H   0.60628  2.06831  3.42850
C  -0.31988 -0.38585  2.87132
H  -1.11546 -1.11471  2.64730
H  -0.35581 -0.15283  3.96093
H   0.64711 -0.85494  2.62864
C  -0.78921 -3.39605  0.39438
H   0.27732 -3.44164  0.12363
H  -1.26353 -4.37978  0.16858
H  -0.85734 -3.21933  1.48069
C  -1.28562 -2.51845 -1.77825
H  -1.73212 -1.68848 -2.34964
H  -1.77739 -3.46573 -2.10398
H  -0.21469 -2.56595 -2.03280
C   1.80220 -0.89457 -0.27036
H   1.76477 -1.85251  0.30183
H   1.68303 -1.19986 -1.33949
C   4.90965 -1.25690 -1.04570
H   4.92878 -2.31781 -0.73788
H   5.92297 -0.84269 -0.88793
H   4.70829 -1.23811 -2.13240
C   3.77986  1.54556 -0.63392
H   3.46895  1.68898 -1.68553
H   4.83319  1.87159 -0.56088
H   3.17636  2.23030 -0.01058
C   4.11481 -0.36091  1.76677
H   3.49994  0.27588  2.42756
H   5.16345 -0.03229  1.87896
H   4.04993 -1.39407  2.15313
```

3Li
SCF (BP86) Energy = -2988.86476079
Enthalpy 0K = -2987.564349
Enthalpy 298K = -2987.471700
Free Energy 298K = -2987.697140
Lowest Frequency = 5.4244 cm^{-1}
Second Frequency = 12.0624 cm^{-1}
SCF (BP86-D3BJ) Energy = -2989.38389496
SCF (C6H6) Energy = -2988.87365280
SCF (BS2) Energy = -4132.08386326

```
Si -5.61307 -0.44233 -1.75153
Si -2.44862  2.90236  3.62757
Si  2.39366 -3.38863  3.35645
Si  5.69495  0.53199 -1.37201
O  -1.46127  0.00756 -0.77795
O  -0.10619  1.50147  1.31678
O   0.01301 -1.47860  1.32623
O   1.49227  0.03136 -0.68463
C  -2.54131  0.07741 -1.70287
C  -2.44196 -1.09843 -2.71420
C  -2.43982  1.45200 -2.41173
C   0.25061  2.52687  2.21713
C   0.01310  3.93963  1.62782
C   1.76930  2.32973  2.54961
C  -0.34024 -2.56654  2.15490
C  -0.25852 -3.91891  1.39794
C  -1.81264 -2.33280  2.63312
C   2.63317  0.02405 -1.53599
C   2.58563 -1.30415 -2.33365
C   2.58885  1.26681 -2.46754
Li  0.01835 -1.24466 -0.65015
Li  1.25086  0.00100  1.22704
Li -1.33204  0.01913  1.15306
Li  0.01224  1.29356 -0.64467
C  -2.78811  4.77042  3.47648
H  -2.19010  5.34523  4.20367
H  -2.55828  5.15846  2.47173
H  -3.85391  4.96983  3.68770
C  -3.01987  2.35824  5.36894
H  -2.47160  2.90229  6.15762
H  -4.09576  2.55944  5.51216
H  -2.85590  1.27908  5.52819
C  -3.46133  1.96320  2.31636
H  -3.06509  2.16594  1.30742
H  -3.44223  0.87387  2.50223
H  -4.52071  2.27352  2.33572
C  -0.58307  2.43326  3.54654
H  -0.09195  3.04386  4.33091
```

```
H  -0.52834  1.38663  3.90559
C   0.60720 -2.67238  3.40241
H   0.10967 -3.28795  4.17962
H   0.69553 -1.65735  3.83877
C   2.43340 -5.27978  3.13320
H   1.77279 -5.78141  3.86057
H   2.11734 -5.58738  2.12398
H   3.45949 -5.65264  3.30074
C   3.09619 -3.00555  5.09509
H   2.50535 -3.50264  5.88409
H   4.13743 -3.36033  5.18694
H   3.09163 -1.92287  5.31114
C   3.51629 -2.56367  2.06072
H   3.13061 -2.71750  1.04002
H   3.59953 -1.47805  2.24245
H   4.53533 -2.98649  2.10874
C   6.90279  0.04544  0.02771
H   6.65193  0.57086  0.96518
H   6.86693 -1.03838  0.23374
H   7.94346  0.30249 -0.23585
C   5.89861  2.39635 -1.71658
H   6.94521  2.62538 -1.98403
H   5.25320  2.73136 -2.54479
H   5.64022  2.99498 -0.82646
C   3.92961  0.15785 -0.67198
H   4.00437 -0.72845 -0.01476
H   3.74166  1.00936  0.01131
C   6.19643 -0.42350 -2.94637
H   7.21827 -0.12386 -3.23998
H   6.19555 -1.51526 -2.79789
H   5.52638 -0.19852 -3.79165
C  -5.78480 -2.28841 -2.19767
H  -5.08315 -2.58153 -2.99550
H  -5.58650 -2.93277 -1.32377
H  -6.80966 -2.50530 -2.54687
C  -6.89464 -0.03076 -0.39347
H  -6.68962 -0.59492  0.53270
H  -6.88044  1.04297 -0.13754
H  -7.91794 -0.28297 -0.72174
C  -6.04208  0.58945 -3.29890
H  -7.05951  0.32420 -3.63742
H  -6.02458  1.67372 -3.10368
H  -5.34983  0.38571 -4.13150
C  -3.89245 -0.10017 -0.93630
H  -4.01122  0.74713 -0.23494
H  -3.74081 -0.98381 -0.28703
C   4.52574  1.82689  3.10339
H   5.58141  1.64817  3.33160
C   3.52566  1.04684  3.70602
H   3.79803  0.25351  4.41047
C   2.16971  1.29565  3.43055
H   1.40853  0.69437  3.93995
C   4.15009  2.84355  2.21218
H   4.91591  3.46310  1.73282
C   2.79357  3.08422  1.93440
H   2.52695  3.89387  1.24933
C   0.47749  5.09609  2.29586
H   1.04792  4.98950  3.22539
C   0.23544  6.37820  1.78254
H   0.61355  7.25680  2.31686
C  -0.48926  6.53705  0.58828
H  -0.67867  7.53776  0.18559
C  -0.97261  5.40053 -0.07671
H  -1.54987  5.49938 -1.00166
C  -0.72171  4.11631  0.44147
H  -1.13997  3.24098 -0.06649
C  -3.18185  3.75597 -2.82458
H  -3.92201  4.54634 -2.65665
C  -2.07408  3.99205 -3.65169
H  -1.94107  4.96453 -4.13746
C  -1.13711  2.96368 -3.85189
H  -0.25883  3.12854 -4.48353
C  -3.35877  2.50190 -2.21161
H  -4.23264  2.34852 -1.57384
C  -1.31976  1.71698 -3.23544
H  -0.58613  0.92275 -3.41038
C   2.26232  2.51679 -1.89391
H   2.03343  2.56724 -0.82226
C   2.27315  3.69685 -2.65232
H   2.01835  4.64932 -2.17563
C   2.61203  3.65432 -4.01590
H   2.62559  4.57270 -4.61247
C   2.93543  2.42108 -4.60334
H   3.20010  2.37154 -5.66549
C   2.92610  1.24140 -3.83642
H   3.18288  0.29032 -4.31258
C   1.53105 -1.51692 -3.25323
H   0.81112 -0.71246 -3.43635
C   1.39361 -2.72639 -3.95016
H   0.56617 -2.85012 -4.65561
C   2.30874 -3.77141 -3.73491
H   2.21141 -4.71474 -4.28287
C   3.34926 -3.58903 -2.81229
H   4.07042 -4.39348 -2.62959
C   3.48306 -2.37054 -2.12160
H   4.30598 -2.25803 -1.41169
C  -2.12575 -2.38379 -2.22158
H  -1.92774 -2.51057 -1.15064
C  -2.09477 -3.50664 -3.06179
H  -1.84730 -4.48775 -2.64309
C  -2.37977 -3.36772 -4.43098
H  -2.36034 -4.24030 -5.09259
C  -2.69030 -2.09692 -4.94060
H  -2.91059 -1.97182 -6.00664
C  -2.72313 -0.97627 -4.09085
H  -2.96755  0.00548 -4.50760
C   0.51265 -4.04125  0.22637
H   1.07250 -3.17503 -0.14360
C   0.62697 -5.27068 -0.44819
H   1.23644 -5.32752 -1.35596
C  -0.03103 -6.40813  0.04430
H   0.05110 -7.36639 -0.47957
C  -0.79128 -6.30625  1.22238
H  -1.30537 -7.18747  1.62182
C  -0.89940 -5.07763  1.89038
H  -1.50266 -5.01265  2.80252
C  -4.22876 -2.44195  2.18034
H  -5.04524 -2.73978  1.51354
C  -2.89877 -2.67095  1.79225
H  -2.69972 -3.16025  0.83374
C  -4.51310 -1.85587  3.42438
H  -5.54916 -1.68897  3.73630
C  -3.44968 -1.49693  4.26661
H  -3.65272 -1.04697  5.24441
C  -2.11977 -1.72919  3.87334
H  -1.31326 -1.46056  4.56164
```

3Li’

tetramer_Li_alltrans
SCF (BP86) Energy = -2988.87157590
Enthalpy 0K = -2987.571828
Enthalpy 298K = -2987.478557
Free Energy 298K = -2987.710090
Lowest Frequency = 11.6519 cm^{-1}
Second Frequency = 12.4202 cm^{-1}
SCF (BP86-D3BJ) Energy = -2989.37147158
SCF (C6H6) Energy = -2988.88139021
SCF (BS2) Energy = -4132.09198683

Si	-3.31438	4.66793	1.58730
Si	-4.39530	-1.12369	-3.84605
Si	4.39582	1.12645	-3.84464
Si	3.31374	-4.66988	1.58431
O	-0.81294	1.23453	1.03158
O	-1.25366	-0.79124	-0.96415
O	1.25331	0.79230	-0.96389
O	0.81358	-1.23540	1.02991
C	-1.63658	2.05109	1.84710
C	-0.75920	2.82567	2.86694
C	-2.63700	1.09791	2.55172
C	-1.97661	-1.53948	-1.92613
C	-2.83061	-2.62773	-1.22166
C	-0.90394	-2.17363	-2.85183
C	1.97650	1.54122	-1.92515
C	2.83047	2.62881	-1.21962
C	0.90411	2.17616	-2.85060
C	1.63690	-2.05253	1.84519
C	2.63732	-1.09992	2.55056
C	0.75913	-2.82745	2.86445
Li	1.05896	0.72242	0.96434
Li	0.70866	-1.05573	-0.88903
Li	-0.70877	1.05657	-0.88779
Li	-1.05854	-0.72325	0.96411
C	-4.93093	0.47064	-4.75533
H	-5.23522	1.25373	-4.03925
H	-4.11057	0.88097	-5.36912
H	-5.78795	0.27853	-5.42402
C	-5.87990	-1.75660	-2.83244
H	-6.19466	-1.00962	-2.08362
H	-6.74187	-1.95088	-3.49471
H	-5.64199	-2.68988	-2.29717
C	-3.93859	-2.43280	-5.15776
H	-3.14867	-2.08309	-5.84252
H	-3.59264	-3.37253	-4.69775
H	-4.83011	-2.66633	-5.76637
C	-2.95322	-0.57694	-2.68090
H	-2.34941	0.18352	-3.21523
H	-3.47066	-0.01936	-1.87454
C	2.95321	0.57917	-2.68042
H	2.34945	-0.18076	-3.21558
H	3.47030	0.02083	-1.87436
C	3.93963	2.43624	-5.15587
H	3.15170	2.08586	-5.84259
H	3.59114	3.37491	-4.69562
H	4.83196	2.67190	-5.76246
C	4.93179	-0.46740	-4.75456
H	4.11164	-0.87736	-5.36888
H	5.78904	-0.27490	-5.42284
H	5.23584	-1.25089	-4.03883
C	5.88004	1.75878	-2.83010
H	5.64218	2.69208	-2.29483
H	6.19415	1.01162	-2.08119
H	6.74243	1.95284	-3.49190
C	4.36204	-5.25498	0.09544
H	3.72870	-5.46183	-0.78494
H	5.10703	-4.49586	-0.20020
H	4.90857	-6.18339	0.33588
C	2.08891	-6.05681	2.03915
H	2.63398	-6.98100	2.29981
H	1.45960	-5.77656	2.89937
H	1.41876	-6.29044	1.19404
C	2.35335	-3.11507	0.94437
H	3.00069	-2.57834	0.22238
H	1.54239	-3.55904	0.33372
C	4.48850	-4.38368	3.06001
H	4.98126	-5.33767	3.31888
H	5.27771	-3.64747	2.83788
H	3.94442	-4.03586	3.95268
C	-2.09040	6.05521	2.04328
H	-1.46078	5.77451	2.90313
H	-1.42054	6.29009	1.19829
H	-2.63601	6.97881	2.30494
C	-4.36271	5.25328	0.09855
H	-3.72935	5.46088	-0.78164
H	-5.10728	4.49393	-0.19755
H	-4.90974	6.18131	0.33936
C	-4.48933	4.38002	3.06250
H	-4.98289	5.33347	3.32185
H	-5.27792	3.64334	2.83969
H	-3.94524	4.03202	3.95511
C	-2.35303	3.11399	0.94667
H	-2.99990	2.57749	0.22408
H	-1.54197	3.55866	0.33667
C	1.28614	-3.21817	-4.35157
H	2.12244	-3.62193	-4.93118
C	0.42477	-2.26451	-4.91323
H	0.58651	-1.91893	-5.94039
C	-0.65310	-1.74749	-4.17087
H	-1.29928	-0.99666	-4.63152
C	1.05533	-3.65557	-3.03527
H	1.70955	-4.40871	-2.58273
C	-0.02461	-3.14039	-2.30114
H	-0.21237	-3.50748	-1.28507
C	-3.01757	-3.92587	-1.74126
H	-2.51405	-4.21476	-2.66923
C	-3.83184	-4.86290	-1.08117
H	-3.95148	-5.86704	-1.50283
C	-4.48391	-4.51814	0.11295
H	-5.11799	-5.24766	0.62801
C	-4.31780	-3.22467	0.63658
H	-4.82519	-2.93011	1.56113
C	-3.50065	-2.29451	-0.02480
H	-3.40367	-1.28061	0.38167
C	-4.85484	0.08510	2.84482
H	-5.92103	0.07435	2.59289
C	-4.33849	-0.84333	3.76058
H	-4.99583	-1.58043	4.23382
C	-2.96687	-0.81326	4.06840
H	-2.54112	-1.53589	4.77175
C	-4.01213	1.04026	2.24638
H	-4.44359	1.74899	1.53507
C	-2.13390	0.14346	3.46929
H	-1.07005	0.16643	3.72756
C	-0.45909	-3.38137	2.41411
H	-0.76665	-3.22161	1.37399

```
C  -1.27378 -4.14297  3.26522
H  -2.21017 -4.56134  2.88126
C  -0.88808 -4.36385  4.59880
H  -1.52102 -4.95662  5.26789
C   0.31866 -3.81745  5.06363
H   0.63036 -3.97759  6.10180
C   1.13468 -3.06077  4.20345
H   2.07020 -2.63937  4.58438
C   2.13399 -0.14538  3.46791
H   1.06992 -0.16786  3.72532
C   2.96697  0.81084  4.06780
H   2.54106  1.53356  4.77096
C   4.33884  0.84027  3.76099
H   4.99619  1.57698  4.23484
C   4.85541 -0.08824  2.84546
H   5.92179 -0.07797  2.59430
C   4.01268 -1.04291  2.24624
H   4.44430 -1.75174  1.53513
C   0.45912  3.37985  2.41723
H   0.76708  3.22049  1.37717
C   1.27347  4.14119  3.26891
H   2.20996  4.55978  2.88542
C   0.88731  4.36153  4.60245
H   1.51999  4.95409  5.27198
C  -0.31955  3.81486  5.06667
H  -0.63160  3.97458  6.10479
C  -1.13521  3.05847  4.20591
H  -2.07083  2.63684  4.58638
C   3.50059  2.29435 -0.02315
H   3.40375  1.27998  0.38221
C   4.31767  3.22385  0.63922
H   4.82512  2.92833  1.56343
C   4.48363  4.51792  0.11701
H   5.11766  5.24693  0.63284
C   3.83148  4.86393 -1.07671
H   3.95101  5.86854 -1.49728
C   3.01727  3.92754 -1.73780
H   2.51370  4.21741 -2.66542
C  -1.05562  3.65762 -3.03306
H  -1.71038  4.40988 -2.57983
C   0.02411  3.14183 -2.29906
H   0.21116  3.50756 -1.28237
C  -1.28554  3.22195 -4.35010
H  -2.12170  3.62618 -4.92959
C  -0.42348  2.26944 -4.91261
H  -0.58451  1.92524 -5.94034
C   0.65419  1.75181 -4.17038
H   1.30097  1.00194 -4.63174
```

3Li·κ[1]L
SCF (BP86) Energy = -3683.12642719
Enthalpy 0K = -3681.409364
Enthalpy 298K = -3681.294241
Free Energy 298K = -3681.568231
Lowest Frequency = 7.7264 cm^{-1}
Second Frequency = 10.3951 cm^{-1}
SCF (BP86-D3BJ) Energy = -3683.75477415
SCF (C6H6) Energy = -3683.13532947
SCF (BS2) Energy = -4826.51313521

```
Si  0.89129 -0.35300  5.43352
Si -0.40464 -5.73044 -0.79344
Si -5.22143 -2.58572 -1.50401
Si -0.58777  4.99485 -3.14112
O  -0.28502  0.60703  1.40722
O  -0.23615 -1.41604 -0.77146
O  -2.86834 -0.74995  0.49643
O  -1.47467  1.25099 -1.23036
C   0.23273  0.99152  2.67774
C  -0.81500  1.84594  3.45612
C   1.52530  1.82119  2.45983
C   0.20729 -2.63673 -1.33782
C   1.70663 -2.87578 -0.98334
C  -0.02053 -2.63919 -2.87241
C  -3.98734 -1.38114  1.08673
C  -5.20351 -0.42748  1.21800
C  -3.56156 -1.92145  2.49801
C  -1.86399  2.41971 -1.94923
C  -2.72912  1.98947 -3.16271
C  -2.64723  3.38989 -1.01330
C   1.37226 -6.35247 -1.09962
H   1.76013 -6.11305 -2.10270
H   2.07222 -5.91895 -0.36638
H   1.39312 -7.45088 -0.98513
C  -1.60926 -6.48868 -2.06659
H  -1.43222 -6.13165 -3.09334
H  -1.52466 -7.58950 -2.06720
H  -2.65158 -6.23727 -1.80300
C  -0.93011 -6.37580  0.92850
H  -0.22576 -6.03661  1.70846
H  -1.93881 -6.02344  1.20607
H  -0.94532 -7.47929  0.95155
C  -0.61684 -3.80632 -0.66781
H  -1.68617 -3.60555 -0.87757
H  -0.48978 -3.63798  0.42039
C  -4.46148 -2.63553  0.26253
H  -5.19934 -3.19439  0.87163
H  -3.59905 -3.32350  0.17251
C  -6.73958 -1.45371 -1.69799
H  -7.53642 -1.73054 -0.98819
H  -6.49120 -0.39620 -1.51460
H  -7.14809 -1.54532 -2.72004
C  -5.78197 -4.38539 -1.82488
H  -6.55551 -4.70212 -1.10399
H  -6.20657 -4.48897 -2.83861
H  -4.93942 -5.09403 -1.74439
C  -3.91283 -2.15130 -2.81792
H  -3.68181 -1.07202 -2.84249
H  -2.98022 -2.72438 -2.67658
H  -4.28536 -2.39543 -3.82818
C   1.07306  5.19526 -4.07022
H   1.93939  5.08718 -3.39568
H   1.18252  4.45218 -4.87930
H   1.13603  6.19725 -4.52984
C  -0.63787  6.28591 -1.73898
H  -0.53104  7.30502 -2.15024
H  -1.58600  6.24219 -1.17867
H   0.18583  6.12682 -1.02190
C  -0.58404  3.20378 -2.39371
H   0.00406  2.54638 -3.06242
H   0.02488  3.30066 -1.47417
C  -1.97703  5.39384 -4.38627
H  -1.82500  6.41773 -4.77210
H  -1.98338  4.70481 -5.24564
H  -2.97383  5.35757 -3.91944
C  -0.65987  0.01352  6.47370
H  -0.97068  1.06712  6.38763
```

H	-1.50557	-0.61611	6.15209
H	-0.45942	-0.19916	7.53884
C	1.41853	-2.16323	5.75248
H	0.62558	-2.86360	5.43857
H	2.33771	-2.42430	5.19954
H	1.61396	-2.33641	6.82504
C	2.29694	0.78388	6.04972
H	2.36488	0.69376	7.14873
H	3.28019	0.51284	5.63219
H	2.11070	1.84333	5.81142
C	0.48812	-0.29663	3.53574
H	1.23910	-0.91838	3.00795
H	-0.46079	-0.87109	3.49333
C	-0.48862	-2.61936	-5.68407
H	-0.67545	-2.61306	-6.76316
C	-0.09072	-3.80123	-5.03820
H	0.04302	-4.72521	-5.61172
C	0.13805	-3.80570	-3.65363
H	0.46427	-4.73397	-3.17758
C	-0.64006	-1.44851	-4.92824
H	-0.95596	-0.51692	-5.40834
C	-0.40339	-1.45969	-3.54180
H	-0.50588	-0.52964	-2.97105
C	2.71008	-3.15604	-1.93346
H	2.45481	-3.18907	-2.99650
C	4.03794	-3.40597	-1.54196
H	4.79422	-3.61753	-2.30592
C	4.39968	-3.39015	-0.18703
H	5.44322	-3.55405	0.10499
C	3.41228	-3.10056	0.77324
H	3.67387	-3.06973	1.83692
C	2.09227	-2.83910	0.37632
H	1.34664	-2.59136	1.13995
C	3.94647	2.17936	2.66396
H	4.92480	1.80408	2.98364
C	3.83098	3.46611	2.11914
H	4.71266	4.10715	2.01484
C	2.56471	3.92215	1.71162
H	2.45205	4.92417	1.28303
C	2.80781	1.36799	2.82356
H	2.93020	0.37404	3.25945
C	1.43582	3.10654	1.87517
H	0.45570	3.49397	1.58279
C	-2.16135	3.58286	0.29900
H	-1.26256	3.03841	0.61504
C	-2.76783	4.48122	1.19159
H	-2.36637	4.59175	2.20415
C	-3.88400	5.22787	0.78133
H	-4.36399	5.93102	1.46986
C	-4.37530	5.06262	-0.52360
H	-5.24182	5.64240	-0.86115
C	-3.76556	4.15535	-1.40885
H	-4.17111	4.04187	-2.41792
C	-4.05320	1.53502	-2.96012
H	-4.46593	1.51655	-1.94619
C	-4.86435	1.13222	-4.03268
H	-5.89057	0.80338	-3.83765
C	-4.36338	1.14496	-5.34434
H	-4.99482	0.83478	-6.18342
C	-3.04238	1.56117	-5.56327
H	-2.63187	1.57649	-6.57919
C	-2.24058	1.97864	-4.48592
H	-1.21997	2.31123	-4.69382
C	-2.17498	1.47416	3.40012
H	-2.47034	0.59630	2.81364
C	-3.15874	2.17627	4.11430
H	-4.20498	1.86203	4.04092
C	-2.80309	3.27445	4.91354
H	-3.56769	3.82737	5.46940
C	-1.45303	3.65172	4.99328
H	-1.15555	4.50285	5.61603
C	-0.47224	2.94358	4.27595
H	0.57282	3.25680	4.35419
C	-5.14063	0.90316	0.76846
H	-4.21910	1.26869	0.30815
C	-6.24719	1.76742	0.86292
H	-6.15468	2.79621	0.49972
C	-7.45160	1.31099	1.41670
H	-8.31577	1.97917	1.49465
C	-7.53726	-0.01719	1.86809
H	-8.47123	-0.39114	2.30219
C	-6.42988	-0.87223	1.76628
H	-6.51900	-1.90110	2.13167
C	-3.68293	-2.01844	4.94940
H	-4.13142	-1.65170	5.87918
C	-4.10075	-1.47384	3.72146
H	-4.87189	-0.69906	3.72168
C	-2.70739	-3.02486	4.99025
H	-2.39418	-3.45816	5.94555
C	-2.13932	-3.46995	3.78443
H	-1.38224	-4.26182	3.78896
C	-2.56146	-2.92145	2.56377
H	-2.13319	-3.31605	1.63547
Li	-0.99199	-1.16601	0.98956
Li	0.40946	0.50077	-0.52324
Li	-2.00519	1.05047	0.64282
Li	-2.01217	-0.61722	-1.25821
N	6.35837	0.49930	-0.70389
N	7.91237	-2.32649	-0.03655
N	9.13280	1.78643	-0.59305
N	2.67197	1.08803	-1.59229
C	6.43017	-0.35107	0.50423
H	5.65057	-1.12844	0.43889
H	6.19658	0.24910	1.41294
C	6.59113	1.92308	-0.43918
H	6.45306	2.46329	-1.39384
C	5.14423	0.27452	-1.51356
H	5.20671	-0.74422	-1.93646
H	5.20673	0.97041	-2.37089
C	3.75523	0.43459	-0.79898
H	3.38497	-0.55923	-0.49774
H	3.87041	1.03049	0.12283
C	2.95100	2.52418	-1.76310
H	3.01563	3.00765	-0.77543
H	2.13169	2.98920	-2.33109
H	3.89615	2.72303	-2.31774
C	2.49986	0.45301	-2.91344
H	3.36597	0.60912	-3.59404
H	1.60917	0.87573	-3.40765
H	2.34514	-0.62874	-2.78587
C	7.77477	-1.07461	0.73817
H	8.62000	-0.37732	0.53914
H	7.80947	-1.35698	1.80759
C	8.93487	-3.19426	0.55215
H	8.99419	-4.13550	-0.02215
H	8.66609	-3.44506	1.59308
H	9.95558	-2.73852	0.55890
C	8.23757	-2.06132	-1.44439

```
H   7.50604 -1.35835 -1.86851
H   8.21311 -3.00754 -2.01376
H   9.24800 -1.60420 -1.57111
C   7.96774  2.28256  0.14625
H   7.98119  3.40060  0.23545
H   8.04642  1.89443  1.18034
C  10.36576  2.24864  0.04110
H  10.48159  3.36106  0.03221
H  11.23792  1.81220 -0.47531
H  10.39051  1.91653  1.09399
C   9.10377  2.12941 -2.01336
H  10.02202  1.75165 -2.49440
H   9.04667  3.22939 -2.20700
H   8.24459  1.63877 -2.49733
H   5.83991  2.35445  0.26861
```

E^{Li}

```
SCF (BP86) Energy = -3683.11767513
Enthalpy 0K = -3681.403045
Enthalpy 298K = -3681.285445
Free Energy 298K = -3681.585862
```
Lowest Frequency = 2.3044 cm^{-1}
Second Frequency = 2.8833 cm^{-1}
```
SCF (BP86-D3BJ) Energy =
                    -3683.64820433
SCF (C6H6) Energy = -3683.13441739
SCF (BS2) Energy = -4826.51333428

Si  0.27239  2.70258  1.78967
Si  8.62759  3.24888  0.22742
Si -4.61315 -4.86778 -0.56554
Si -10.33456 -0.26268 -0.92867
O  -3.76398  1.42146  1.03983
O   8.26477 -0.08913 -0.07124
O  -4.50335 -1.83816  1.09825
O  -6.06779  0.23207 -0.99193
C  -2.78144  2.44271  1.07006
C  -3.16004  3.52116  2.11880
C  -2.77860  3.03200 -0.36622
C   9.54927  0.39984 -0.18227
C  10.35416 -0.55743 -1.12419
C  10.29261  0.41989  1.18976
C  -3.98509 -2.98347  1.72527
C  -5.04955 -3.70919  2.59159
C  -2.85997 -2.48311  2.69074
C  -7.33263  0.57046 -1.52228
C  -7.59306  2.05875 -1.13659
C  -7.34584  0.35851 -3.05733
C   8.46995  4.71585 -0.99386
H   7.98949  5.58477 -0.51099
H   9.45932  5.04293 -1.35859
H   7.86247  4.44522 -1.87517
C   9.62963  3.85557  1.73286
H  10.64415  4.16889  1.43218
H   9.13471  4.72476  2.20122
H   9.73676  3.06662  2.49438
C   6.87554  2.75007  0.79098
H   6.27126  2.41981 -0.07083
H   6.93587  1.90872  1.49901
H   6.35688  3.59479  1.27783
C   9.53215  1.86281 -0.74484
H   9.04411  1.82178 -1.73875
H  10.56129  2.23746 -0.92022
C  -3.42576 -3.97509  0.65721
H  -2.85164 -4.79068  1.14183
H  -2.70112 -3.41286  0.03666
C  -5.46793 -6.37375  0.22513
H  -4.72840 -7.07108  0.65455
H  -6.15436 -6.07607  1.03392
H  -6.04766 -6.92642 -0.53490
C  -3.51954 -5.46840 -2.01126
H  -2.71650 -6.13688 -1.65533
H  -4.11473 -6.02855 -2.75289
H  -3.04278 -4.62130 -2.53432
C  -5.97016 -3.73128 -1.29174
H  -6.64424 -3.36582 -0.49583
H  -5.55528 -2.88383 -1.86877
H  -6.60026 -4.29859 -1.99895
C  -10.93352 -1.00382  0.72912
H  -10.55043 -2.02950  0.87090
H  -10.59567 -0.39616  1.58646
H  -12.03564 -1.05252  0.76721
C  -11.04154 -1.35840 -2.32231
H  -12.14526 -1.33260 -2.31131
H  -10.70047 -1.04830 -3.32244
H  -10.73212 -2.40826 -2.17735
C  -8.40279 -0.36931 -0.84663
H  -8.18320 -0.32434  0.23928
H  -8.15348 -1.40355 -1.16283
C  -11.02588  1.51101 -1.04696
H  -12.12802  1.48268 -0.98399
H  -10.65295  2.13273 -0.21621
H  -10.75568  2.02434 -1.98399
C   0.32043  3.45128  3.54211
H  -0.43448  4.24348  3.66916
H   0.12609  2.67643  4.30377
H   1.31431  3.88459  3.75060
C   1.58518  1.31702  1.69518
H   1.31868  0.48149  2.36636
H   1.68778  0.91613  0.66977
H   2.57213  1.70110  2.00881
C   0.74607  4.03942  0.51302
H   1.77088  4.39278  0.72453
H   0.73130  3.64785 -0.51685
H   0.07926  4.91591  0.54707
C  -1.41348  1.80177  1.48190
H  -1.20357  0.98203  0.76533
H  -1.60873  1.29787  2.45164
C  11.58512  0.48623  3.71426
H  12.08563  0.51216  4.68838
C  12.31191  0.73709  2.53687
H  13.38437  0.95629  2.59216
C  11.67130  0.70219  1.28922
H  12.25216  0.88086  0.37667
C  10.21388  0.19851  3.62754
H   9.63787  0.00241  4.54000
C   9.57883  0.16448  2.37345
H   8.50864 -0.04712  2.27216
C  10.91828 -0.17811 -2.35662
H  10.86521  0.86553 -2.68098
C  11.55093 -1.12075 -3.19121
H  11.97825 -0.79579 -4.14701
C  11.64253 -2.46393 -2.80264
H  12.14089 -3.19627 -3.44712
C  11.09872 -2.85679 -1.56415
H  11.18308 -3.89960 -1.23552
C  10.46514 -1.91489 -0.74388
H  10.04989 -2.21954  0.22336
```

C -2.02194 3.01707 -2.70049
H -1.30457 2.67081 -3.45289
C -3.07158 3.87097 -3.07381
H -3.18059 4.19398 -4.11418
C -3.97912 4.30693 -2.09507
H -4.80620 4.97180 -2.36365
C -1.88212 2.59909 -1.36644
H -1.06174 1.92419 -1.10668
C -3.83225 3.89393 -0.75948
H -4.53575 4.26199 -0.00321
C -6.18119 -0.09375 -3.70667
H -5.28697 -0.24869 -3.09614
C -6.16407 -0.32587 -5.09297
H -5.24380 -0.67908 -5.57190
C -7.31467 -0.10015 -5.86318
H -7.30433 -0.27924 -6.94363
C -8.47985 0.36770 -5.23319
H -9.38354 0.56135 -5.82181
C -8.49190 0.59347 -3.84754
H -9.40500 0.97530 -3.38040
C -7.71643 3.11040 -2.06799
H -7.69588 2.88808 -3.13836
C -7.86555 4.44372 -1.64398
H -7.96646 5.23788 -2.39235
C -7.88501 4.76037 -0.27782
H -8.00157 5.79829 0.05091
C -7.74911 3.72732 0.66611
H -7.75326 3.95556 1.73715
C -7.60292 2.39805 0.23924
H -7.48918 1.60623 0.98819
C -4.01419 3.17786 3.18427
H -4.43374 2.16732 3.20081
C -4.34039 4.11155 4.18177
H -5.00499 3.81985 5.00303
C -3.82778 5.41735 4.12325
H -4.08636 6.14970 4.89555
C -2.98874 5.77903 3.05630
H -2.59391 6.79886 2.98863
C -2.65867 4.83861 2.06654
H -2.01776 5.13912 1.23106
C -6.37641 -3.24634 2.63559
H -6.63029 -2.34496 2.06836
C -7.34990 -3.91163 3.40263
H -8.37822 -3.53393 3.42213
C -7.00413 -5.04800 4.14901
H -7.75917 -5.56721 4.74864
C -5.67507 -5.50731 4.13091
H -5.39040 -6.38564 4.72064
C -4.70908 -4.84190 3.36282
H -3.67338 -5.20117 3.36996
C -2.28263 -1.08849 4.63365
H -2.60620 -0.46223 5.47187
C -3.23997 -1.65741 3.77893
H -4.30727 -1.48890 3.96724
C -0.91444 -1.33830 4.42500
H -0.16500 -0.90483 5.09490
C -0.52274 -2.15699 3.35567
H 0.53945 -2.36526 3.18669
C -1.48441 -2.71967 2.49599
H -1.15368 -3.35725 1.67091
Li -3.60795 -0.29085 1.59797
Li 6.90156 -1.11050 0.02160
Li -5.01231 1.59050 -0.31888
Li -5.59932 -1.35311 -0.24514
N 5.75929 -2.36173 -1.34663
N 2.65288 -0.08426 -1.71482
N 6.64492 -0.90427 -4.14467
N 6.20230 -2.37435 1.60755
C 4.55041 -1.77389 -1.99615
H 3.97136 -2.58529 -2.49497
H 4.92999 -1.10910 -2.79219
C 6.74500 -2.76940 -2.39885
H 7.54576 -3.33076 -1.88621
C 5.42209 -3.52900 -0.48584
H 6.28082 -4.22290 -0.50206
H 4.55900 -4.09878 -0.89609
C 5.11368 -3.15384 0.97532
H 4.20269 -2.53585 1.02130
H 4.89595 -4.08762 1.54498
C 5.71742 -1.69651 2.82525
H 4.87898 -1.02706 2.57179
H 6.53064 -1.08800 3.25235
H 5.37339 -2.41498 3.60229
C 7.36527 -3.22256 1.94142
H 7.10543 -4.01387 2.67987
H 8.16649 -2.59084 2.35680
H 7.76014 -3.70811 1.03462
C 3.62207 -0.96784 -1.05373
H 3.03769 -1.64497 -0.40241
H 4.24522 -0.33499 -0.39075
C 1.66644 -0.79514 -2.52856
H 0.86669 -0.09411 -2.82607
H 1.20531 -1.60154 -1.93170
H 2.07504 -1.24745 -3.46216
C 3.26530 1.03232 -2.43744
H 3.95083 1.57448 -1.76413
H 2.47372 1.73438 -2.75244
H 3.83739 0.73785 -3.34681
C 7.44641 -1.60559 -3.12125
H 7.76020 -0.87223 -2.35331
H 8.38771 -2.01184 -3.56377
C 6.45983 -1.71833 -5.34890
H 7.42528 -1.98657 -5.84239
H 5.84321 -1.16606 -6.07841
H 5.93147 -2.65535 -5.10618
C 7.27677 0.37430 -4.49067
H 6.65205 0.90868 -5.22707
H 8.29641 0.25172 -4.92649
H 7.36588 1.00017 -3.58771
H 6.27298 -3.47347 -3.12097

TS(E-F)^{Li}
SCF (BP86) Energy = -3683.07578840
Enthalpy 0K = -3681.363067
Enthalpy 298K = -3681.246229
Free Energy 298K = -3681.540368
Lowest Frequency = -398.3731 cm^{-1}
Second Frequency = 2.0130 cm^{-1}
SCF (BP86-D3BJ) Energy = -3683.61764156
SCF (C6H6) Energy = -3683.09100420
SCF (BS2) Energy = -4826.47331963

Si -1.60871 5.49164 0.70532
Si 6.19264 -1.16453 2.01032
Si -1.62934 -3.94288 2.48603
Si -7.90582 -3.74212 -1.27344
O -3.82193 1.83657 0.25419

O	6.50327	-1.27889	0.30229
O	-3.23804	-0.89752	2.07534
O	-4.48494	-1.15875	-0.99200
C	-3.50802	3.14994	-0.17502
C	-4.74616	4.07279	-0.02158
C	-3.10062	2.99423	-1.66490
C	7.74171	-2.64271	0.35436
C	8.89678	-1.90566	-0.29733
C	7.10887	-3.71936	-0.50683
C	-2.59493	-1.18224	3.29320
C	-3.55504	-1.84271	4.31865
C	-2.15084	0.19602	3.88616
C	-5.45352	-1.80024	-1.79543
C	-6.37137	-0.67674	-2.36833
C	-4.76504	-2.61576	-2.91873
C	7.45608	-0.28166	3.16247
H	7.04611	0.64881	3.59645
H	7.75949	-0.94532	3.98936
H	8.37098	-0.02415	2.60093
C	5.03200	-2.36422	2.97333
H	5.59613	-2.91684	3.74230
H	4.20416	-1.81992	3.46406
H	4.58565	-3.11797	2.30186
C	4.90348	0.32563	1.74467
H	5.36538	1.22562	1.29080
H	4.01606	0.03939	1.14062
H	4.50473	0.64876	2.72439
C	7.76921	-2.95210	1.75618
H	8.64164	-2.66540	2.35114
H	7.32324	-3.90468	2.05776
C	-1.37227	-2.12250	3.05198
H	-0.74867	-2.19554	3.96577
H	-0.73585	-1.64251	2.28335
C	-2.10582	-5.09302	3.92548
H	-1.36997	-5.02781	4.74502
H	-3.09345	-4.83913	4.34251
H	-2.13337	-6.14227	3.58274
C	0.04526	-4.51024	1.76725
H	0.85433	-4.41484	2.51160
H	0.00504	-5.56694	1.45138
H	0.32838	-3.90697	0.88721
C	-2.94823	-4.13819	1.11202
H	-3.94605	-3.83263	1.47614
H	-2.68607	-3.59042	0.18723
H	-3.03400	-5.19919	0.81883
C	-8.80808	-3.90488	0.40440
H	-8.16889	-4.39849	1.15711
H	-9.09672	-2.91695	0.80332
H	-9.72800	-4.50661	0.30357
C	-7.53336	-5.51121	-1.88523
H	-8.47233	-6.06748	-2.05219
H	-6.95420	-5.52563	-2.82174
H	-6.95066	-6.06267	-1.12678
C	-6.28217	-2.76847	-0.86772
H	-6.55602	-2.15811	0.01654
H	-5.56727	-3.53186	-0.49715
C	-9.08568	-2.84502	-2.47397
H	-10.02315	-3.42056	-2.57066
H	-9.33970	-1.84150	-2.09413
H	-8.66573	-2.71346	-3.48443
C	-2.71670	6.69102	1.68935
H	-3.70990	6.80260	1.22581
H	-2.86519	6.32882	2.72131
H	-2.24960	7.68972	1.74930
C	0.06374	5.33636	1.61553
H	-0.06157	4.81939	2.58284
H	0.80863	4.77327	1.02366
H	0.48364	6.33669	1.82383
C	-1.26417	6.22579	-1.02338
H	-0.72322	7.18208	-0.90942
H	-0.64142	5.55682	-1.63911
H	-2.18897	6.43337	-1.58501
C	-2.35777	3.70771	0.72787
H	-1.50574	3.00210	0.65754
H	-2.73070	3.60607	1.76808
C	6.00140	-5.92035	-1.93332
H	5.57552	-6.76726	-2.48178
C	7.37836	-5.65011	-1.99798
H	8.03601	-6.28911	-2.59785
C	7.92463	-4.56856	-1.29063
H	9.00248	-4.38288	-1.33634
C	5.18011	-5.09323	-1.15023
H	4.10511	-5.29641	-1.08079
C	5.72651	-4.00375	-0.45137
H	5.08686	-3.35108	0.14706
C	10.06100	-1.54573	0.41282
H	10.15057	-1.81079	1.46979
C	11.12283	-0.87357	-0.21537
H	12.01339	-0.61118	0.36639
C	11.05842	-0.55961	-1.58134
H	11.89407	-0.05287	-2.07569
C	9.91203	-0.92276	-2.31096
H	9.85207	-0.70327	-3.38340
C	8.84871	-1.57780	-1.67435
H	7.96527	-1.86714	-2.25266
C	-1.43310	2.55283	-3.40986
H	-0.38037	2.47390	-3.70392
C	-2.44392	2.33511	-4.35903
H	-2.18945	2.08430	-5.39399
C	-3.78674	2.44590	-3.96400
H	-4.59262	2.28043	-4.68606
C	-1.75783	2.87200	-2.08071
H	-0.94916	3.02437	-1.36044
C	-4.10894	2.77377	-2.63581
H	-5.16325	2.88130	-2.35227
C	-3.36027	-2.61852	-3.01528
H	-2.80091	-2.00775	-2.30066
C	-2.70034	-3.37150	-4.00195
H	-1.60561	-3.35779	-4.05241
C	-3.43576	-4.13270	-4.92272
H	-2.92432	-4.72003	-5.69282
C	-4.83866	-4.12712	-4.85037
H	-5.42887	-4.70672	-5.56892
C	-5.49191	-3.37712	-3.85948
H	-6.58599	-3.37385	-3.83124
C	-6.48725	-0.36537	-3.73894
H	-5.95986	-0.97556	-4.47727
C	-7.27157	0.71852	-4.17483
H	-7.34838	0.92977	-5.24745
C	-7.95082	1.52561	-3.25040
H	-8.56020	2.36920	-3.59078
C	-7.83595	1.24029	-1.87846
H	-8.34868	1.86605	-1.14058
C	-7.05547	0.15582	-1.44797
H	-6.96914	-0.04687	-0.37456
C	-5.72103	3.75845	0.94516
H	-5.60057	2.83165	1.51452
C	-6.82921	4.59490	1.15669

H	-7.57162	4.33143	1.91867
C	-6.99268	5.75990	0.38972
H	-7.85971	6.41034	0.54736
C	-6.03776	6.07678	-0.59025
H	-6.15984	6.97422	-1.20694
C	-4.92597	5.24155	-0.79051
H	-4.19785	5.49238	-1.56886
C	-4.88030	-2.15163	3.96489
H	-5.22280	-1.88616	2.95944
C	-5.74942	-2.76839	4.88303
H	-6.77825	-3.00088	4.58619
C	-5.30508	-3.07519	6.17779
H	-5.97955	-3.55376	6.89568
C	-3.98827	-2.75106	6.55032
H	-3.63410	-2.97413	7.56277
C	-3.12514	-2.13858	5.63066
H	-2.10593	-1.87804	5.93891
C	-2.84856	2.39480	4.74177
H	-3.65092	3.06965	5.05893
C	-3.16323	1.10340	4.28906
H	-4.20831	0.77056	4.27534
C	-1.50679	2.81052	4.80879
H	-1.25582	3.81165	5.17384
C	-0.49340	1.92425	4.41458
H	0.55611	2.23319	4.46726
C	-0.81237	0.63310	3.95490
H	-0.00277	-0.04060	3.65989
Li	-3.24547	0.92102	1.70462
Li	5.54297	-0.06473	-0.68995
Li	-4.37503	0.68178	-1.08351
Li	-3.81863	-1.73916	0.59400
N	5.79077	1.88075	-1.75253
N	3.33691	4.30096	0.03081
N	8.10430	3.31420	0.09145
N	4.26150	-0.64319	-2.30158
C	5.26964	3.15970	-1.17906
H	5.43669	3.97674	-1.91942
H	5.90450	3.38940	-0.30667
C	7.27196	1.99761	-1.94232
H	7.59968	1.09075	-2.48076
C	5.14454	1.57720	-3.06349
H	5.89584	1.09952	-3.71654
H	4.83784	2.51052	-3.58368
C	3.92519	0.64765	-2.94423
H	3.14664	1.12584	-2.32845
H	3.48683	0.49498	-3.95813
C	3.03651	-1.32976	-1.84362
H	2.50157	-0.69585	-1.11674
H	3.31645	-2.27321	-1.34951
H	2.34470	-1.56316	-2.68295
C	5.00376	-1.53251	-3.22051
H	4.40493	-1.78287	-4.12455
H	5.26571	-2.46326	-2.69328
H	5.93933	-1.05190	-3.54904
C	3.78042	3.14527	-0.75952
H	3.12654	3.10234	-1.65156
H	3.58566	2.23640	-0.15827
C	3.39902	5.57390	-0.68710
H	2.85148	6.34027	-0.11098
H	2.90379	5.47189	-1.66862
H	4.43145	5.95878	-0.85921
C	3.95121	4.38762	1.35705
H	3.86993	3.41196	1.86506
H	3.40253	5.13440	1.95732
H	5.02639	4.68145	1.34900
C	8.11330	2.04080	-0.65431
H	7.75664	1.24015	0.01963
H	9.15411	1.75452	-0.94267
C	8.80349	4.37936	-0.63227
H	9.87493	4.13565	-0.83443
H	8.77017	5.30886	-0.03925
H	8.31272	4.58285	-1.59823
C	8.70863	3.12740	1.41553
H	8.65924	4.07423	1.97994
H	9.77839	2.81246	1.36494
H	8.15356	2.35892	1.97694
H	7.50877	2.86284	-2.60202

F^{Li}

SCF (BP86) Energy = -3683.15149779
Enthalpy 0K = -3681.437973
Enthalpy 298K = -3681.319205
Free Energy 298K = -3681.622134
Lowest Frequency = 1.9842 cm^{-1}
Second Frequency = 4.4678 cm^{-1}
SCF (BP86-D3BJ) Energy = -3683.67379016
SCF (C6H6) Energy = -3683.16759148
SCF (BS2) Energy = -4826.55234831

Si	-2.11131	5.53789	0.31134
Si	4.73452	-0.32150	2.69687
Si	-1.13730	-3.86209	2.12498
Si	-7.83322	-4.17971	-0.82809
O	-4.00195	1.68445	0.13652
O	5.31932	-0.12629	1.16826
O	-3.02067	-0.95486	1.91463
O	-4.57692	-1.38295	-0.98887
C	-3.85148	3.00872	-0.34436
C	-5.15147	3.81875	-0.09526
C	-3.56612	2.84970	-1.86206
C	9.43522	-2.08860	-0.13771
C	10.48581	-2.02711	-1.19884
C	8.38102	-3.14341	-0.22306
C	-2.20615	-1.17338	3.04032
C	-2.97386	-1.89059	4.18332
C	-1.79638	0.24046	3.57062
C	-5.58621	-2.10360	-1.66474
C	-6.63180	-1.05346	-2.15128
C	-4.97745	-2.90308	-2.84422
C	6.13813	-0.57022	3.97615
H	5.74523	-0.68074	5.00277
H	6.72292	-1.47776	3.74395
H	6.83589	0.28557	3.97550
C	3.56577	-1.83711	2.84837
H	3.15524	-1.93574	3.86971
H	2.71508	-1.74769	2.14887
H	4.09960	-2.77464	2.61255
C	3.70903	1.20125	3.26913
H	4.32868	2.11564	3.28668
H	2.86728	1.37976	2.57463
H	3.28950	1.06106	4.28193
C	9.44806	-1.19806	0.88807
H	10.18965	-0.39465	0.93216
H	8.71546	-1.24587	1.69808
C	-0.95617	-2.01953	2.64413
H	-0.21341	-2.03407	3.46670
H	-0.46497	-1.49911	1.79910

C	-1.28314	-5.02346	3.62589
H	-0.43604	-4.88225	4.31867
H	-2.21128	-4.84477	4.19197
H	-1.27508	-6.07742	3.29679
C	0.45019	-4.29265	1.15678
H	1.35207	-4.06047	1.74902
H	0.48236	-5.36448	0.89546
H	0.51012	-3.71575	0.21729
C	-2.62984	-4.19884	0.97284
H	-3.58247	-3.95027	1.47506
H	-2.55044	-3.67081	0.00370
H	-2.67815	-5.27365	0.72450
C	-8.52540	-4.35416	0.94568
H	-7.77380	-4.78756	1.62836
H	-8.82921	-3.37586	1.35683
H	-9.41025	-5.01390	0.96337
C	-7.41997	-5.93823	-1.44460
H	-8.33321	-6.55749	-1.48375
H	-6.95871	-5.94279	-2.44459
H	-6.71447	-6.42971	-0.75206
C	-6.24137	-3.09639	-0.63076
H	-6.45355	-2.48074	0.26665
H	-5.44121	-3.80308	-0.32836
C	-9.19901	-3.39178	-1.90168
H	-10.10256	-4.02608	-1.87474
H	-9.47127	-2.39460	-1.51785
H	-8.90791	-3.26574	-2.95719
C	-3.24069	6.66469	1.35512
H	-4.27381	6.67636	0.97280
H	-3.27326	6.32190	2.40375
H	-2.86041	7.70134	1.35383
C	-0.36443	5.56720	1.08338
H	-0.36879	5.09460	2.08100
H	0.37675	5.03333	0.46108
H	-0.01572	6.60802	1.20788
C	-1.97772	6.24156	-1.45884
H	-1.53110	7.25091	-1.41517
H	-1.33784	5.61718	-2.10324
H	-2.95879	6.33442	-1.95175
C	-2.68345	3.69441	0.43938
H	-1.77803	3.06883	0.30602
H	-2.95165	3.58735	1.51073
C	6.41874	-5.19127	-0.30140
H	5.66063	-5.98146	-0.32914
C	7.72319	-5.44566	-0.75483
H	7.99116	-6.43835	-1.13280
C	8.69199	-4.43216	-0.71922
H	9.70952	-4.64185	-1.06476
C	6.09553	-3.91352	0.18698
H	5.08249	-3.69782	0.54393
C	7.06048	-2.89414	0.22591
H	6.76389	-1.90094	0.59473
C	11.82413	-1.70173	-0.87686
H	12.09683	-1.56096	0.17428
C	12.80080	-1.58698	-1.87617
H	13.83211	-1.34132	-1.60065
C	12.46433	-1.80291	-3.22270
H	13.22817	-1.71951	-4.00292
C	11.14297	-2.14118	-3.55836
H	10.87050	-2.31636	-4.60489
C	10.16702	-2.25769	-2.55815
H	9.13985	-2.52606	-2.82596
C	-2.02651	2.50770	-3.74191
H	-1.00101	2.51341	-4.12839
C	-3.09374	2.17528	-4.59092
H	-2.91024	1.91864	-5.63938
C	-4.40086	2.17934	-4.07826
H	-5.24947	1.92420	-4.72097
C	-2.25992	2.83429	-2.39554
H	-1.40747	3.07693	-1.75489
C	-4.63250	2.51509	-2.73322
H	-5.66332	2.53862	-2.35817
C	-3.59604	-2.81708	-3.10294
H	-3.00133	-2.15274	-2.46928
C	-3.00417	-3.55035	-4.14601
H	-1.92593	-3.46637	-4.32358
C	-3.78726	-4.38084	-4.96158
H	-3.32905	-4.95293	-5.77545
C	-5.16982	-4.46449	-4.72714
H	-5.79804	-5.09903	-5.36223
C	-5.75442	-3.73343	-3.68078
H	-6.83598	-3.79954	-3.52674
C	-6.91492	-0.78203	-3.50601
H	-6.43397	-1.37697	-4.28722
C	-7.80722	0.24276	-3.87148
H	-8.01262	0.42404	-4.93260
C	-8.43029	1.02971	-2.89174
H	-9.12313	1.82801	-3.17730
C	-8.15018	0.78384	-1.53605
H	-8.61557	1.39665	-0.75717
C	-7.26254	-0.24217	-1.17553
H	-7.04799	-0.41411	-0.11480
C	-5.99911	3.44587	0.96604
H	-5.73883	2.55230	1.54178
C	-7.15728	4.18280	1.26276
H	-7.79779	3.87617	2.09761
C	-7.50007	5.30364	0.48905
H	-8.40661	5.87595	0.71343
C	-6.67299	5.67745	-0.58280
H	-6.93510	6.54100	-1.20427
C	-5.51002	4.94273	-0.86838
H	-4.88235	5.23696	-1.71601
C	-4.30896	-2.29523	4.00746
H	-4.79808	-2.06497	3.05529
C	-5.00455	-2.96052	5.03325
H	-6.04451	-3.26738	4.87486
C	-4.37390	-3.22087	6.25899
H	-4.91310	-3.73703	7.06039
C	-3.04588	-2.80133	6.45453
H	-2.54728	-2.98739	7.41216
C	-2.35605	-2.14051	5.42816
H	-1.32646	-1.80536	5.59902
C	-2.55035	2.40119	4.47415
H	-3.36130	3.02815	4.86057
C	-2.82304	1.08735	4.06024
H	-3.84158	0.68898	4.14700
C	-1.23659	2.89991	4.41544
H	-1.01690	3.91862	4.75122
C	-0.20925	2.07358	3.93639
H	0.82178	2.44074	3.89254
C	-0.48729	0.75999	3.51505
H	0.33581	0.13636	3.15461
Li	-3.19377	0.85144	1.52666
Li	5.01834	0.39986	-0.46362
Li	-4.60365	0.45889	-1.11544
Li	-3.70517	-1.87254	0.52722
N	5.67795	2.10294	-1.61757
N	2.99235	4.61157	-0.40201

N	7.56752	3.44409	0.71127
N	4.25690	-0.42108	-2.27466
C	5.09747	3.42129	-1.22842
H	5.36620	4.18359	-1.99570
H	5.59991	3.71348	-0.29020
C	7.16337	2.12238	-1.43191
H	7.55354	1.18936	-1.87638
C	5.32744	1.72893	-3.01698
H	6.20009	1.23051	-3.47408
H	5.13156	2.62944	-3.63771
C	4.10763	0.79258	-3.11009
H	3.20524	1.31922	-2.75697
H	3.93197	0.53398	-4.18066
C	2.95734	-1.09906	-2.09957
H	2.23503	-0.40782	-1.63457
H	3.08716	-1.96534	-1.43176
H	2.53245	-1.45634	-3.06366
C	5.23568	-1.36762	-2.84779
H	4.91925	-1.73678	-3.84896
H	5.35497	-2.22731	-2.16968
H	6.22159	-0.88720	-2.95166
C	3.56462	3.40695	-1.01398
H	3.03796	3.25777	-1.97632
H	3.30757	2.54817	-0.36149
C	3.09144	5.80797	-1.23759
H	2.46078	6.60395	-0.80436
H	2.70916	5.58744	-2.24977
H	4.12362	6.21785	-1.34017
C	3.44699	4.85720	0.96812
H	3.33526	3.93663	1.56550
H	2.81000	5.63656	1.42141
H	4.50672	5.19211	1.04905
C	7.63307	2.13437	0.03570
H	7.01015	1.41741	0.60834
H	8.67690	1.73338	0.04969
C	8.60394	4.36335	0.23642
H	9.63797	3.98213	0.42118
H	8.50028	5.33485	0.74892
H	8.49815	4.54380	-0.84624
C	7.66717	3.27209	2.16466
H	7.55993	4.25151	2.66175
H	8.64169	2.83096	2.48502
H	6.85941	2.60960	2.51476
H	7.61865	2.95922	-2.00792

1Na (κ^4)

SCF (BP86) Energy = -857.495290800
Enthalpy 0K = -856.947715
Enthalpy 298K = -856.913319
Free Energy 298K = -857.012691
Lowest Frequency = 23.6776 cm^{-1}
Second Frequency = 30.3470 cm^{-1}
SCF (BP86-D3BJ) Energy = -857.622227464
SCF (C6H6) Energy = -857.502120206
SCF (BS2) Energy = -1305.42135644

```
Si -3.70857 -0.17661  0.23453
Na -0.02776 -0.13320  0.19374
N   2.65579  0.21358 -0.18215
N   0.67647  2.33156  0.81736
N   0.58138 -0.42504 -2.36328
N   1.34381 -1.92662  1.49531
C   2.94735  1.65700 -0.04995
H   2.73242  2.14114 -1.01728
H   4.03101  1.83883  0.14491
C   2.13054  2.35660  1.05092
H   2.31796  1.85953  2.02029
H   2.51594  3.40003  1.16016
C   2.97267 -0.28050 -1.53739
H   3.04780 -1.37969 -1.49092
H   3.97289  0.07663 -1.88196
C   1.93535  0.10663 -2.60596
H   1.85043  1.20748 -2.65677
H   2.33002 -0.21712 -3.60017
C   3.36221 -0.56652  0.85634
H   3.33155  0.01625  1.79254
H   4.44454 -0.69055  0.61125
C   2.76260 -1.96118  1.11734
H   2.84640 -2.57915  0.20430
H   3.39716 -2.46681  1.88641
C  -0.06097  2.64871  2.05281
H  -1.14157  2.54383  1.86819
H   0.14495  3.68080  2.42159
H   0.21192  1.93192  2.84468
C   0.27481  3.26618 -0.24479
H   0.78297  3.02196 -1.19253
H   0.51152  4.32679  0.00879
H  -0.81138  3.18108 -0.41270
C  -0.39709  0.25724 -3.22828
H  -0.37913  1.34196 -3.03001
H  -1.41008 -0.11327 -3.00255
H  -0.19663  0.09621 -4.31326
C   0.51484 -1.87627 -2.60158
H   1.20620 -2.41196 -1.93093
H   0.77050 -2.14694 -3.65317
H  -0.50529 -2.23415 -2.38658
C   0.70519 -3.24292  1.34795
H  -0.37579 -3.13349  1.53412
H   1.11862 -4.00563  2.04905
H   0.84058 -3.61267  0.31706
C   1.13119 -1.43265  2.86433
H   1.59929 -0.44276  2.99646
H   1.55480 -2.11926  3.63523
H   0.04712 -1.31614  3.03075
C  -2.22782 -0.54728  1.27620
H  -2.32441 -1.58801  1.66075
H  -2.24551  0.10659  2.17815
C  -5.45568 -0.17943  1.06940
H  -5.67292 -1.16700  1.51471
H  -6.27070  0.05607  0.35930
H  -5.49956  0.56224  1.88755
C  -3.57245  1.57450 -0.57308
H  -3.44127  2.35361  0.20129
H  -4.47196  1.84156 -1.15683
H  -2.70290  1.62990 -1.25423
C  -3.86526 -1.43142 -1.22187
H  -2.95301 -1.43619 -1.84697
H  -4.72288 -1.20287 -1.87957
H  -4.00345 -2.45941 -0.84066
```

3Na

SCF (BP86) Energy = -2959.55971421
Enthalpy 0K = -2958.266606
Enthalpy 298K = -2958.170749
Free Energy 298K = -2958.404049
Lowest Frequency = 11.6901 cm^{-1}
Second Frequency = 15.4993 cm^{-1}
SCF (BP86-D3BJ) Energy = -2960.06998388
SCF (C6H6) Energy = -2959.57014299
SCF (BS2) Energy = -4751.13639215

```
Si -5.72618 -1.38542 -1.87222
Si -3.14366  2.48765  3.76324
Si  3.14397 -2.48632  3.76392
Si  5.72585  1.38491 -1.87312
O  -1.65352 -0.44399 -0.91548
O  -0.51662  1.64958  1.45528
O   0.51690 -1.64909  1.45588
O   1.65325  0.44360 -0.91592
C  -2.72486 -0.54496 -1.81978
C  -2.48135 -1.71890 -2.81361
C  -2.79880  0.82656 -2.54760
C  -0.41419  2.70482  2.36325
C  -0.93783  4.05188  1.78918
C   1.10974  2.86150  2.69080
C   0.41446 -2.70412  2.36408
C   0.93813 -4.05128  1.79027
C  -1.10944 -2.86071  2.69178
C   2.72458  0.54436 -1.82024
C   2.79862 -0.82742 -2.54755
C   2.48091  1.71791 -2.81449
C  -3.83767  4.25880  3.67905
H  -3.35481  4.90819  4.42883
H  -3.68536  4.71779  2.68948
H  -4.92123  4.24712  3.89297
C  -3.59346  1.77099  5.47881
H  -3.15415  2.37541  6.29145
H  -4.68741  1.75707  5.62691
H  -3.22425  0.73689  5.59324
C  -4.00219  1.41008  2.43951
H  -3.66464  1.67127  1.42302
H  -3.82846  0.33310  2.61897
H  -5.09554  1.56091  2.47960
C  -1.22256  2.42199  3.68407
H  -0.89699  3.12138  4.48228
H  -0.93831  1.41033  4.03835
C   1.22285 -2.42095  3.68482
H   0.89744 -3.12024  4.48318
H   0.93844 -1.40927  4.03891
C   3.83812 -4.25745  3.68028
H   3.35524 -4.90665  4.43021
```

H 3.68587 -4.71672 2.69083
H 4.92166 -4.24568 3.89426
C 3.59375 -1.76904 5.47923
H 3.15440 -2.37314 6.29208
H 4.68769 -1.75508 5.62737
H 3.22456 -0.73489 5.59327
C 4.00243 -1.40918 2.43981
H 3.66513 -1.67100 1.42339
H 3.82837 -0.33215 2.61865
H 5.09581 -1.55969 2.48021
C 7.05892 1.13828 -0.52359
H 6.81440 1.71473 0.38559
H 7.14611 0.07667 -0.23340
H 8.05016 1.47200 -0.87656
C 5.70604 3.22874 -2.36003
H 6.69624 3.53880 -2.73758
H 4.96078 3.43246 -3.14611
H 5.46400 3.86691 -1.49253
C 4.05088 0.88224 -1.04845
H 4.25448 0.06006 -0.33424
H 3.79610 1.75368 -0.41291
C 6.23063 0.35939 -3.40094
H 7.21586 0.70199 -3.76387
H 6.30832 -0.71684 -3.17677
H 5.50981 0.47918 -4.22593
C -5.70641 -3.22944 -2.35839
H -4.96117 -3.43352 -3.14439
H -5.46437 -3.86726 -1.49063
H -6.69663 -3.53964 -2.73579
C -7.05917 -1.13827 -0.52271
H -6.81456 -1.71437 0.38666
H -7.14632 -0.07655 -0.23291
H -8.05044 -1.47212 -0.87545
C -6.23102 -0.36051 -3.40044
H -7.21622 -0.70332 -3.76326
H -6.30880 0.71579 -3.17667
H -5.51018 -0.48055 -4.22538
C -4.05118 -0.88241 -1.04783
H -4.25472 -0.05990 -0.33399
H -3.79640 -1.75356 -0.41189
C 3.94268 2.91895 3.05639
H 5.02742 2.94729 3.20152
C 3.11871 2.24690 3.97370
H 3.55960 1.74988 4.84500
C 1.72228 2.21990 3.79160
H 1.10321 1.70495 4.53295
C 3.35202 3.56596 1.95602
H 3.97682 4.10748 1.23732
C 1.96005 3.53212 1.77847
H 1.51337 4.05737 0.92752
C -0.63963 5.28704 2.40577
H 0.00604 5.30438 3.29142
C -1.14198 6.49288 1.89422
H -0.89247 7.43853 2.38836
C -1.96006 6.49144 0.75058
H -2.35056 7.43271 0.34909
C -2.27369 5.27085 0.13309
H -2.91300 5.24690 -0.75607
C -1.76504 4.06662 0.65153
H -2.04481 3.11184 0.19396
C -3.68719 3.10483 -2.80637
H -4.45983 3.84216 -2.55992
C -2.64139 3.44672 -3.67644
H -2.58328 4.45089 -4.10911
C -1.66844 2.47914 -3.98861
H -0.83876 2.72495 -4.65986
C -3.75971 1.81514 -2.24682
H -4.58414 1.58485 -1.56682
C -1.75292 1.19351 -3.43187
H -0.99663 0.44582 -3.69398
C 2.00100 2.93903 -2.28887
H 1.82841 3.00971 -1.20822
C 1.78812 4.05924 -3.10598
H 1.42583 4.99468 -2.66483
C 2.04584 3.98293 -4.48645
H 1.87967 4.85309 -5.13051
C 2.52980 2.78118 -5.02612
H 2.74277 2.70794 -6.09859
C 2.74992 1.66475 -4.19785
H 3.13249 0.73913 -4.63908
C 1.75258 -1.19497 -3.43138
H 0.99605 -0.44755 -3.69360
C 1.66826 -2.48084 -3.98758
H 0.83845 -2.72712 -4.65849
C 2.64154 -3.44806 -3.67533
H 2.58357 -4.45240 -4.10760
C 3.68752 -3.10556 -2.80572
H 4.46043 -3.84259 -2.55922
C 3.75988 -1.81564 -2.24667
H 4.58446 -1.58488 -1.56703
C -2.00140 -2.93984 -2.28761
H -1.82868 -3.01011 -1.20696
C -1.78866 -4.06036 -3.10433
H -1.42632 -4.99564 -2.66288
C -2.04658 -3.98457 -4.48479
H -1.88052 -4.85497 -5.12854
C -2.53061 -2.78301 -5.02484
H -2.74374 -2.71018 -6.09730
C -2.75058 -1.66627 -4.19696
H -3.13322 -0.74081 -4.63848
C 1.76521 -4.06619 0.65254
H 2.04491 -3.11150 0.19474
C 2.27388 -5.27051 0.13429
H 2.91310 -5.24670 -0.75494
C 1.96039 -6.49099 0.75207
H 2.35091 -7.43232 0.35073
C 1.14243 -6.49225 1.89579
H 0.89305 -7.43781 2.39015
C 0.64007 -5.28633 2.40716
H -0.00549 -5.30354 3.29288
C -3.35181 -3.56514 1.95724
H -3.97670 -4.10668 1.23862
C -1.95986 -3.53136 1.77956
H -1.51327 -4.05666 0.92859
C -3.94234 -2.91804 3.05763
H -5.02706 -2.94635 3.20287
C -3.11828 -2.24593 3.97479
H -3.55907 -1.74882 4.84609
C -1.72186 -2.21898 3.79256
H -1.10270 -1.70397 4.53379
Na -1.54520 -0.38973 1.36125
Na -0.38157 1.53313 -0.88994
Na 0.38134 -1.53354 -0.88923
Na 1.54536 0.39023 1.36076

3Na’
SCF (BP86) Energy = -2959.55971154
Enthalpy 0K = -2958.267382

Enthalpy 298K = -2958.170829
Free Energy 298K = -2958.411609
Lowest Frequency = 6.8709 cm^{-1}
Second Frequency = 9.0076 cm^{-1}
SCF (BP86-D3BJ) Energy =
-2960.05291742
SCF (C6H6) Energy = -2959.57236088
SCF (BS2) Energy = -4751.13860558

Si	4.31642	4.10530	-1.77830
Si	3.90009	-2.02861	4.39959
Si	-3.90290	1.90965	4.45015
Si	-4.31655	-4.05501	-1.88938
O	1.18542	1.24770	-1.13870
O	1.26110	-1.17453	1.12910
O	-1.26100	1.14373	1.16044
O	-1.18535	-1.21629	-1.17164
C	2.08777	1.92157	-1.97424
C	1.32740	2.90828	-2.90808
C	2.82491	0.81666	-2.78174
C	1.83013	-2.06077	2.05403
C	2.74233	-3.10150	1.34440
C	0.61916	-2.74064	2.75515
C	-1.83082	2.00506	2.10817
C	-2.74271	3.06406	1.42577
C	-0.62045	2.66613	2.82808
C	-2.08700	-1.86772	-2.02552
C	-2.82279	-0.74167	-2.80460
C	-1.32599	-2.82977	-2.98425
C	3.11047	-3.41961	5.43995
H	2.17943	-3.09107	5.93060
H	2.87168	-4.30446	4.82798
H	3.81491	-3.73767	6.22869
C	4.33419	-0.58501	5.57785
H	3.43594	-0.20000	6.09171
H	5.05120	-0.90615	6.35324
H	4.79173	0.25439	5.02555
C	5.52020	-2.66168	3.62045
H	5.33857	-3.49715	2.92526
H	6.02516	-1.85749	3.05758
H	6.21616	-3.01063	4.40334
C	2.74468	-1.26174	3.05381
H	2.11842	-0.49327	3.55099
H	3.43645	-0.69114	2.40113
C	-2.74586	1.17931	3.08547
H	-2.11987	0.39775	3.56212
H	-3.43698	0.62634	2.41710
C	-3.11575	3.27349	5.52775
H	-2.18479	2.93311	6.01041
H	-2.87757	4.17471	4.93993
H	-3.82128	3.56956	6.32407
C	-4.33687	0.43521	5.58957
H	-3.43878	0.03748	6.09392
H	-5.05484	0.73523	6.37249
H	-4.79327	-0.38965	5.01486
C	-5.52275	2.56143	3.68598
H	-5.34109	3.41484	3.01291
H	-6.02631	1.77167	3.10184
H	-6.21995	2.88935	4.47681
C	-5.54812	-4.40783	-0.46846
H	-5.02076	-4.74738	0.44020
H	-6.12626	-3.50543	-0.20267
H	-6.26803	-5.19537	-0.75119
C	-3.41068	-5.67788	-2.31324
H	-4.13240	-6.45349	-2.62411
H	-2.68695	-5.53534	-3.13215
H	-2.85871	-6.06489	-1.43921
C	-3.07354	-2.75414	-1.17959
H	-3.63325	-2.08485	-0.49487
H	-2.41484	-3.35693	-0.52190
C	-5.31456	-3.49150	-3.41439
H	-6.01012	-4.29516	-3.71444
H	-5.91094	-2.58649	-3.21443
H	-4.65850	-3.27826	-4.27375
C	3.41022	5.73827	-2.16039
H	2.68721	5.61683	-2.98332
H	2.85742	6.10213	-1.27699
H	4.13190	6.52196	-2.45036
C	5.54641	4.42110	-0.34734
H	5.01803	4.73630	0.56947
H	6.12474	3.51226	-0.10491
H	6.26619	5.21617	-0.60847
C	5.31624	3.58262	-3.31664
H	6.01176	4.39420	-3.59464
H	5.91276	2.67282	-3.14011
H	4.66100	3.39197	-4.18193
C	3.07306	2.78580	-1.10428
H	3.63211	2.09880	-0.43678
H	2.41340	3.37101	-0.43183
C	-1.87096	-3.71911	3.76447
H	-2.82309	-4.09466	4.15317
C	-1.14092	-2.75398	4.47576
H	-1.52320	-2.37113	5.42847
C	0.08487	-2.27477	3.97707
H	0.62670	-1.51908	4.55263
C	-1.35454	-4.20353	2.54823
H	-1.90155	-4.96723	1.98424
C	-0.13322	-3.71750	2.05360
H	0.25400	-4.10577	1.10552
C	2.87295	-4.44496	1.75538
H	2.28534	-4.80982	2.60394
C	3.73597	-5.33112	1.08644
H	3.80980	-6.37139	1.42286
C	4.49791	-4.88929	-0.00646
H	5.16950	-5.57909	-0.52878
C	4.39334	-3.54958	-0.41732
H	4.98432	-3.18236	-1.26336
C	3.52493	-2.67274	0.25106
H	3.46892	-1.62229	-0.05779
C	4.70052	-0.73196	-3.14176
H	5.73043	-1.02579	-2.90885
C	3.95334	-1.46791	-4.07298
H	4.38713	-2.34301	-4.56778
C	2.63777	-1.06281	-4.36679
H	2.03634	-1.62472	-5.08941
C	4.14059	0.39087	-2.50264
H	4.74861	0.93811	-1.77726
C	2.08991	0.06157	-3.73070
H	1.07016	0.37664	-3.97714
C	-0.27105	-3.60239	-2.44945
H	-0.03483	-3.49877	-1.38359
C	0.44750	-4.51246	-3.23943
H	1.25539	-5.10166	-2.79145
C	0.12823	-4.66809	-4.59991
H	0.68771	-5.37393	-5.22311
C	-0.92174	-3.91479	-5.14753
H	-1.18582	-4.02936	-6.20494
C	-1.64278	-3.01007	-4.34654

H -2.45738 -2.43081 -4.79286
C -2.08630 0.03790 -3.73231
H -1.06641 -0.27107 -3.98563
C -2.63275 1.17900 -4.33918
H -2.03002 1.75955 -5.04579
C -3.94849 1.57689 -4.03639
H -4.38120 2.46503 -4.50836
C -4.69713 0.81698 -3.12578
H -5.72722 1.10499 -2.88650
C -4.13855 -0.32265 -2.51586
H -4.74778 -0.88850 -1.80590
C 0.27176 3.66628 -2.35414
H 0.03471 3.53496 -1.29153
C -0.44670 4.59624 -3.12068
H -1.25533 5.17299 -2.65810
C -0.12648 4.78749 -4.47637
H -0.68591 5.50903 -5.08138
C 0.92429 4.04922 -5.04264
H 1.18909 4.19142 -6.09650
C 1.64516 3.12424 -4.26495
H 2.46035 2.55719 -4.72560
C -3.52414 2.66453 0.32055
H -3.46798 1.62263 -0.01597
C -4.39174 3.55886 -0.32536
H -4.98161 3.21428 -1.18163
C -4.49680 4.88716 0.12086
H -5.16773 5.59065 -0.38376
C -3.73624 5.29971 1.22610
H -3.81046 6.33066 1.58998
C -2.87393 4.39608 1.87218
H -2.28708 4.73827 2.73066
C 1.35355 4.13384 2.66169
H 1.90123 4.91197 2.11846
C 0.13270 3.66107 2.15322
H -0.25350 4.07416 1.21527
C 1.86864 3.61767 3.86538
H 2.82037 3.98280 4.26483
C 1.13775 2.63431 4.55035
H 1.51887 2.22669 5.49319
C -0.08752 2.16843 4.03793
H -0.63015 1.39819 4.59310
Na 1.10251 1.10779 1.13834
Na 1.12660 -1.09930 -1.18499
Na -1.12610 1.13068 -1.15508
Na -1.10259 -1.13795 1.10788

3Na·κ^1L
SCF (BP86) Energy = -3653.82990533
Enthalpy 0K = -3652.121366
Enthalpy 298K = -3652.002424
Free Energy 298K = -3652.289604
Lowest Frequency = 4.8539 cm^{-1}
Second Frequency = 10.2591 cm^{-1}
SCF (BP86-D3BJ) Energy =
-3654.45078349
SCF (C6H6) Energy = -3653.84127569
SCF (BS2) Energy = -5445.57431765

Si -0.38256 -5.87264 -1.10480
Si -0.03529 -2.13988 4.90269
Si 5.76399 1.28870 2.63829
Si 3.56889 5.02006 -2.25394
O 0.27033 -1.62482 -0.87964
O 0.16587 0.13937 2.03553
O 3.23927 -0.48382 0.60457
O 1.11066 1.74574 -0.92914
C -0.17098 -2.77058 -1.56463
C 0.80898 -3.13563 -2.72899
C -1.58425 -2.48968 -2.14069
C -0.12867 0.53805 3.34161
C -1.64923 0.48376 3.66475
C 0.36734 2.01365 3.50474
C 4.43290 -1.04345 1.07133
C 5.61891 -0.84706 0.08594
C 4.18210 -2.57667 1.27597
C 1.47643 2.71523 -1.88236
C 1.70686 1.95100 -3.21601
C 0.39351 3.82146 -2.03363
C -1.59402 -2.10108 5.99449
H -1.45701 -1.42445 6.85504
H -2.48509 -1.76667 5.44062
H -1.79265 -3.11244 6.39199
C 1.37572 -2.87323 5.96756
H 1.55921 -2.25218 6.86181
H 1.11698 -3.88855 6.31599
H 2.32584 -2.94093 5.40970
C -0.33394 -3.32435 3.43304
H -1.02886 -2.89551 2.69284
H 0.60818 -3.60915 2.93095
H -0.78318 -4.26370 3.80136
C 0.57769 -0.38010 4.41518
H 0.59527 0.14147 5.39520
H 1.64176 -0.48302 4.11725
C 4.88370 -0.41341 2.45007
H 5.57943 -1.11068 2.96220
H 3.98692 -0.36513 3.10081
C 7.56733 1.28220 2.02890
H 8.13554 0.44239 2.46305
H 7.63331 1.20144 0.93243
H 8.06376 2.21955 2.33759
C 5.79185 1.61215 4.52396
H 6.33584 0.81386 5.05832
H 6.29454 2.56838 4.75208
H 4.77177 1.65948 4.94357
C 4.85083 2.74519 1.81072
H 4.69522 2.57017 0.73405
H 3.88164 2.95732 2.29714
H 5.45540 3.66444 1.90866
C 5.36906 5.08428 -1.61261
H 5.39679 5.11437 -0.50967
H 5.94729 4.20109 -1.93582
H 5.89128 5.98192 -1.98710
C 2.68018 6.60835 -1.68800
H 3.18287 7.50078 -2.10032
H 1.62784 6.62521 -2.01487
H 2.69520 6.69301 -0.58741
C 2.77457 3.46923 -1.40716
H 3.57888 2.71931 -1.26838
H 2.53783 3.83159 -0.38635
C 3.62862 4.98468 -4.16098
H 4.17187 5.87526 -4.52381
H 4.14711 4.09136 -4.54559
H 2.61963 5.00474 -4.60278
C 1.22211 -6.54956 -1.87817
H 1.43288 -6.08492 -2.85498
H 2.08449 -6.35626 -1.21860
H 1.14342 -7.64123 -2.02541
C -0.68457 -6.80245 0.53944

H	0.13654	-6.61514	1.25294
H	-1.62598	-6.48133	1.01846
H	-0.74618	-7.89223	0.37404
C	-1.82456	-6.28332	-2.28679
H	-1.78210	-7.35813	-2.53855
H	-2.81155	-6.08355	-1.83927
H	-1.76119	-5.71494	-3.22858
C	-0.16635	-4.01425	-0.59617
H	-0.86218	-3.80336	0.24047
H	0.84823	-4.02525	-0.14542
C	1.28591	4.71716	3.57430
H	1.63164	5.75543	3.61052
C	2.02638	3.69777	4.19045
H	2.95946	3.93423	4.71372
C	1.57126	2.36441	4.15456
H	2.15958	1.59538	4.66464
C	0.08598	4.38673	2.91878
H	-0.51102	5.17273	2.44322
C	-0.35834	3.05685	2.88099
H	-1.30154	2.81684	2.38093
C	-2.20394	1.17294	4.76535
H	-1.56376	1.82457	5.37090
C	-3.56253	1.04225	5.09225
H	-3.97098	1.59053	5.94850
C	-4.39555	0.20424	4.33092
H	-5.45328	0.09108	4.59090
C	-3.85585	-0.48764	3.23403
H	-4.49369	-1.15018	2.63804
C	-2.49833	-0.33557	2.89908
H	-2.06802	-0.86552	2.04290
C	-4.00989	-2.85396	-2.28117
H	-4.89963	-3.38484	-1.92508
C	-4.11594	-1.92735	-3.32821
H	-5.08250	-1.73479	-3.80603
C	-2.96030	-1.24812	-3.75691
H	-3.02509	-0.51733	-4.57169
C	-2.75936	-3.12538	-1.69442
H	-2.70661	-3.86561	-0.89197
C	-1.72004	-1.51752	-3.16028
H	-0.82478	-0.99614	-3.51377
C	-0.21964	4.31269	-0.86191
H	0.03038	3.84007	0.09362
C	-1.12347	5.38554	-0.90661
H	-1.57361	5.75887	0.02071
C	-1.45734	5.98186	-2.13501
H	-2.16685	6.81524	-2.17277
C	-0.86728	5.49602	-3.31179
H	-1.11693	5.94726	-4.27862
C	0.05412	4.43426	-3.25858
H	0.51148	4.07841	-4.18686
C	0.59527	1.44578	-3.93366
H	-0.41380	1.70688	-3.59846
C	0.75929	0.63480	-5.06639
H	-0.12190	0.27762	-5.61079
C	2.04605	0.27180	-5.50308
H	2.17390	-0.37313	-6.37779
C	3.16097	0.74284	-4.79586
H	4.17228	0.47219	-5.11848
C	2.99064	1.57710	-3.67371
H	3.88199	1.94828	-3.15940
C	2.19817	-3.06613	-2.48349
H	2.54817	-2.78882	-1.48222
C	3.14001	-3.40667	-3.46726
H	4.21052	-3.34706	-3.23914

C	2.71242	-3.83389	-4.73559
H	3.44237	-4.09875	-5.50799
C	1.33639	-3.92975	-4.99277
H	0.98414	-4.27416	-5.97185
C	0.39885	-3.58821	-4.00142
H	-0.66814	-3.67233	-4.22733
C	5.51909	0.09405	-0.95354
H	4.58294	0.65469	-1.04550
C	6.59159	0.33116	-1.83135
H	6.49091	1.07661	-2.62873
C	7.79038	-0.38480	-1.68777
H	8.62766	-0.20730	-2.37110
C	7.90576	-1.33085	-0.65382
H	8.83647	-1.89552	-0.52944
C	6.83453	-1.55340	0.22447
H	6.93923	-2.29575	1.02382
C	4.08810	-4.86676	0.38035
H	4.28568	-5.55721	-0.44671
C	4.39819	-3.50496	0.23258
H	4.84806	-3.15813	-0.70288
C	3.53843	-5.34493	1.58094
H	3.30592	-6.40787	1.70216
C	3.30483	-4.43934	2.62929
H	2.89152	-4.79277	3.58051
C	3.62266	-3.07768	2.47538
H	3.46358	-2.40126	3.32246
Na	1.26056	-1.67819	1.20520
Na	-0.78408	0.44504	-0.20547
Na	2.08906	-0.33612	-1.50146
Na	2.00277	1.38315	1.16021
N	-6.90436	0.31630	-0.24479
N	-8.11829	2.93512	0.74323
N	-9.48201	-1.04142	-0.95552
N	-3.23665	1.51800	-0.54270
C	-7.14214	0.65616	1.16855
H	-6.23483	1.10917	1.61750
H	-7.34322	-0.26707	1.74818
C	-6.96887	-1.10729	-0.56846
H	-6.67213	-1.21461	-1.62800
C	-5.76025	1.02522	-0.84096
H	-5.88969	2.08971	-0.58000
H	-5.85299	0.95133	-1.94136
C	-4.34528	0.53181	-0.40596
H	-4.37225	0.23755	0.65824
H	-4.06906	-0.36557	-0.98726
C	-3.15517	2.06312	-1.91340
H	-3.07267	1.22788	-2.62903
H	-2.26092	2.70243	-2.00184
H	-4.03671	2.68040	-2.19419
C	-3.37605	2.61230	0.43623
H	-4.30097	3.21646	0.30297
H	-2.51802	3.29578	0.33238
H	-3.38323	2.19122	1.45567
C	-8.32354	1.62113	1.38926
H	-9.26053	1.13837	1.03588
H	-8.42245	1.77575	2.48079
C	-8.58481	4.04639	1.57259
H	-8.35416	5.00241	1.06993
H	-8.06229	4.04110	2.54450
H	-9.68537	4.02870	1.76973
C	-8.74615	2.98588	-0.58222
H	-8.42591	2.10849	-1.16363
H	-8.43685	3.90691	-1.10818
H	-9.86278	2.97681	-0.53095

C -8.35223 -1.75158 -0.35020
H -8.27370 -2.80826 -0.72018
H -8.55898 -1.82581 0.73455
C -10.73729 -1.72005 -0.63731
H -10.80330 -2.75568 -1.05480
H -11.58656 -1.13951 -1.03705
H -10.85659 -1.79070 0.45796
C -9.33239 -0.86874 -2.39911
H -10.22110 -0.35064 -2.79777
H -9.22347 -1.83442 -2.95351
H -8.45349 -0.24010 -2.61390
H -6.24278 -1.73298 0.00989

3Na·2κ^1L
SCF (BP86) Energy = -4348.10750947
Enthalpy 0K = -4345.983401
Enthalpy 298K = -4345.841421
Free Energy 298K = -4346.181937
Lowest Frequency = 5.5375 cm^{-1}
Second Frequency = 6.3550 cm^{-1}
SCF (BP86-D3BJ) Energy =
-4348.83413883
SCF (C6H6) Energy = -4348.11912288
SCF (BS2) Energy = -6140.02031326

Si -1.32773 -6.83096 -2.31712
Si -1.44568 -0.43650 -4.95819
Si -6.21137 1.25188 -1.02296
Si -0.25484 1.54682 5.70648
O -0.19289 -3.29607 -0.13746
O   0.02698 -0.16751 -1.54963
O -2.78052 -0.99915 0.27555
O   0.18705 -0.47150 1.92854
C -0.14296 -4.68598 -0.35291
C -0.94294 -5.45276 0.75272
C   1.34136 -5.14198 -0.34482
C   0.42297 0.55964 -2.66987
C   1.52207 -0.18135 -3.50036
C   1.05648 1.92474 -2.22105
C -4.16933 -0.84228 0.15487
C -4.86227 -0.76141 1.54738
C -4.70886 -2.05124 -0.66377
C   0.48753 -0.31039 3.29421
C   0.22574 -1.68498 3.97431
C   1.94927 0.17722 3.51368
C -0.33275 -0.48506 -6.50285
H -0.12177 0.53066 -6.87889
H   0.63220 -0.97519 -6.29736
H -0.83992 -1.04359 -7.30968
C -3.14699 0.26111 -5.48660
H -3.05049 1.28833 -5.87948
H -3.60197 -0.35863 -6.27896
H -3.85254 0.29538 -4.63780
C -1.71637 -2.24387 -4.39963
H -0.79676 -2.69015 -3.98652
H -2.55152 -2.36547 -3.68733
H -1.98801 -2.84543 -5.28542
C -0.78718 0.81760 -3.65170
H -0.55432 1.66571 -4.32762
H -1.64140 1.15943 -3.03367
C -4.47518 0.52283 -0.57582
H -3.88092 0.53212 -1.51187
H -4.00867 1.30182 0.06012
C -7.51633 0.01765 -1.66842
H -7.14921 -0.54269 -2.54338
H -7.82341 -0.71866 -0.90880
H -8.41807 0.57701 -1.97542
C -5.87797 2.48834 -2.44618
H -5.52158 1.97091 -3.35412
H -6.79263 3.04504 -2.71440
H -5.10654 3.22531 -2.16134
C -6.93775 2.22979 0.44449
H -7.12798 1.57787 1.31217
H -6.24394 3.02598 0.76676
H -7.88954 2.71059 0.15798
C -1.91349 2.43395 6.05969
H -2.13439 3.19759 5.29387
H -2.76116 1.72696 6.08099
H -1.88244 2.94345 7.03883
C   1.12233 2.86327 5.78057
H   1.15132 3.33430 6.77887
H   2.11526 2.43246 5.57484
H   0.93874 3.66111 5.03992
C -0.41296 0.82222 3.91844
H -1.47466 0.54856 3.76370
H -0.24924 1.69991 3.26388
C   0.01305 0.26420 7.09334
H -0.02127 0.77240 8.07332
H -0.76562 -0.51604 7.08708
H   0.99046 -0.23686 7.00971
C -2.89671 -7.44468 -1.42803
H -2.70546 -7.65012 -0.36221
H -3.70060 -6.69207 -1.48821
H -3.26170 -8.37510 -1.89804
C -1.73555 -6.62938 -4.17543
H -2.52284 -5.87056 -4.32585
H -0.84857 -6.31277 -4.75171
H -2.09531 -7.57936 -4.60786
C   0.01085 -8.18168 -2.15060
H -0.41506 -9.14621 -2.48048
H   0.90084 -7.97911 -2.76777
H   0.34543 -8.30162 -1.10763
C -0.85315 -5.05008 -1.71222
H -0.30780 -4.54360 -2.53355
H -1.84722 -4.55858 -1.66406
C   2.42014 4.28907 -1.37181
H   2.95139 5.19283 -1.05550
C   1.37786 4.36440 -2.30474
H   1.08063 5.33347 -2.72054
C   0.70950 3.19657 -2.72379
H -0.08806 3.29712 -3.46564
C   2.77189 3.03164 -0.84741
H   3.58816 2.94830 -0.12142
C   2.09131 1.87799 -1.25967
H   2.38627 0.90702 -0.85235
C   2.32114 0.48801 -4.45366
H   2.21661 1.57160 -4.57780
C   3.24994 -0.21011 -5.24102
H   3.85698 0.33279 -5.97436
C   3.39901 -1.60097 -5.09600
H   4.11876 -2.14845 -5.71404
C   2.61974 -2.27722 -4.14365
H   2.73416 -3.35807 -4.00410
C   1.70020 -1.56940 -3.34846
H   1.10632 -2.09703 -2.59321
C   3.36988 -6.02817 -1.41509
H   3.86890 -6.39239 -2.32024
C   4.05507 -6.01413 -0.19220

H	5.08860	-6.37055	-0.12909
C	3.39733	-5.52498	0.95090
H	3.92118	-5.49626	1.91339
C	2.03130	-5.59716	-1.48638
H	1.51736	-5.64375	-2.45010
C	2.06905	-5.08060	0.86764
H	1.56190	-4.71317	1.76556
C	2.46704	1.14194	2.62402
H	1.85879	1.43717	1.76276
C	3.72804	1.72280	2.83316
H	4.09324	2.49387	2.14524
C	4.52429	1.32516	3.92178
H	5.51329	1.76826	4.07815
C	4.03249	0.35117	4.80452
H	4.63854	0.02667	5.65780
C	2.75506	-0.20417	4.60845
H	2.38361	-0.94754	5.32029
C	1.16309	-2.73764	3.84139
H	2.12552	-2.53287	3.36141
C	0.89573	-4.02692	4.32718
H	1.65325	-4.81220	4.22765
C	-0.33785	-4.31841	4.93441
H	-0.55341	-5.32790	5.29695
C	-1.29229	-3.29798	5.05469
H	-2.26216	-3.50276	5.52114
C	-1.00886	-2.00015	4.58855
H	-1.77179	-1.22624	4.71330
C	-2.21517	-4.96149	1.12186
H	-2.62069	-4.08288	0.60584
C	-3.00673	-5.61086	2.08197
H	-3.99222	-5.20485	2.33740
C	-2.54684	-6.78496	2.70144
H	-3.16033	-7.29465	3.45213
C	-1.29744	-7.30356	2.32999
H	-0.92830	-8.22787	2.78908
C	-0.50983	-6.64789	1.36653
H	0.45872	-7.07606	1.09329
C	-4.10830	-0.33833	2.65779
H	-3.04535	-0.12582	2.50187
C	-4.69142	-0.18653	3.92675
H	-4.07979	0.15672	4.76928
C	-6.05300	-0.47025	4.11736
H	-6.51104	-0.35875	5.10594
C	-6.82080	-0.89884	3.02177
H	-7.88408	-1.12924	3.15285
C	-6.23199	-1.03549	1.75346
H	-6.84564	-1.37904	0.91486
C	-5.45107	-4.39497	-0.76953
H	-5.72276	-5.31629	-0.24310
C	-5.05750	-3.26656	-0.03270
H	-5.04359	-3.31996	1.06069
C	-5.49764	-4.34645	-2.17244
H	-5.81394	-5.22161	-2.74928
C	-5.13914	-3.15506	-2.82302
H	-5.17446	-3.09320	-3.91643
C	-4.75045	-2.02827	-2.07669
H	-4.50174	-1.10636	-2.61298
Na	-1.48907	-1.87848	-1.43100
Na	1.35180	-1.44532	0.06513
Na	-1.32590	-2.25617	1.63927
Na	-1.11691	0.75491	0.35261
N	7.11242	1.02182	-0.61926
N	9.07848	1.70999	1.84245
N	9.23075	2.26853	-2.41488
N	4.13829	-1.43328	0.01024
C	6.99866	2.02707	0.45501
H	6.50882	1.56506	1.33119
H	6.34377	2.86929	0.13099
C	6.83030	1.54291	-1.96089
H	6.83144	0.68444	-2.65737
C	6.42051	-0.24961	-0.33504
H	6.80292	-0.61989	0.63242
H	6.74240	-0.97811	-1.10251
C	4.86681	-0.17680	-0.30356
H	4.55570	0.57121	0.44795
H	4.50057	0.17041	-1.28601
C	4.41608	-2.48705	-0.98323
H	4.24275	-2.09201	-1.99700
H	3.73567	-3.33801	-0.82242
H	5.45484	-2.88253	-0.92691
C	4.43148	-1.91761	1.37199
H	5.48480	-2.25183	1.50620
H	3.78883	-2.78852	1.58670
H	4.21812	-1.12400	2.10604
C	8.33331	2.62254	0.95532
H	8.95089	2.95427	0.09013
H	8.07703	3.52048	1.54945
C	9.95296	2.44468	2.75632
H	10.45329	1.73314	3.43698
H	9.36020	3.14411	3.37150
H	10.75246	3.03153	2.23893
C	9.86051	0.72423	1.08577
H	9.21176	0.23144	0.34716
H	10.27308	-0.03072	1.77902
H	10.71072	1.18646	0.52816
C	7.80838	2.61723	-2.47058
H	7.48126	2.86541	-3.51433
H	7.67514	3.54483	-1.88107
C	10.04125	3.37067	-2.92957
H	9.85006	3.59949	-4.00764
H	11.11252	3.12974	-2.81865
H	9.83534	4.28741	-2.34948
C	9.54078	1.01869	-3.10571
H	10.62660	0.83218	-3.04716
H	9.25350	1.02322	-4.18678
H	9.02965	0.17813	-2.61016
H	5.81458	2.00367	-2.03988
N	-3.35935	6.13562	0.38605
N	-0.83609	8.36905	0.28039
N	-4.47386	7.58422	-2.23803
N	-1.73587	3.34585	0.78728
C	-2.94723	7.30306	1.16628
H	-2.23499	6.96966	1.94899
H	-3.84044	7.66650	1.70776
C	-4.72869	6.15603	-0.12432
H	-4.89358	5.21599	-0.68146
C	-2.33793	5.54390	-0.49121
H	-1.36376	5.98111	-0.21213
H	-2.48740	5.83812	-1.55274
C	-2.25877	3.99720	-0.44341
H	-3.25478	3.55827	-0.64106
H	-1.59806	3.68513	-1.27534
C	-0.42206	3.89083	1.17283
H	0.26731	3.85327	0.31382
H	0.00605	3.28099	1.98673
H	-0.46987	4.94161	1.53562
C	-2.67629	3.42573	1.92367
H	-2.84115	4.46309	2.27736

H -2.28653 2.81646 2.75707
H -3.65334 3.00884 1.62723
C -2.30329 8.48754 0.38992
H -2.71496 8.48612 -0.63462
H -2.58954 9.45993 0.86621
C -0.16775 8.75028 1.52639
H 0.91960 8.58771 1.43185
H -0.52727 8.12994 2.36388
H -0.33699 9.82170 1.79839
C -0.32301 9.17039 -0.83281
H -0.79034 8.83929 -1.77529
H 0.76862 9.03024 -0.91951
H -0.51515 10.26518 -0.71580
C -5.19950 7.36319 -0.97751
H -6.30009 7.24139 -1.15377
H -5.09057 8.28211 -0.37161
C -4.79660 8.90333 -2.79095
H -5.87908 9.02324 -3.03986
H -4.21697 9.07144 -3.71494
H -4.52410 9.69041 -2.06700
C -4.76473 6.54554 -3.23026
H -4.17952 6.73567 -4.14607
H -5.84489 6.50558 -3.51240
H -4.47769 5.55226 -2.84982
H -5.42973 6.11291 0.73565

3Na·κ^2L
SCF (BP86) Energy = -3653.79682518
Enthalpy 0K = -3652.088474
Enthalpy 298K = -3651.969363
Free Energy 298K = -3652.255760
Lowest Frequency = 8.3500 cm^{-1}
Second Frequency = 10.4035 cm^{-1}
SCF (BP86-D3BJ) Energy = -3654.42298697
SCF (C6H6) Energy = -3653.81351180
SCF (BS2) Energy = -5445.54194425

Si 1.51074 6.01480 -0.45863
Si 2.21049 0.19720 4.92790
Si -3.71508 -1.13078 4.23665
Si -5.54945 -3.97387 -1.64348
O 0.07040 1.99141 -0.95559
O 1.36159 -0.75990 1.42257
O -1.93579 0.69527 1.55175
O -2.15397 -1.41484 -1.05314
C 0.37371 3.25343 -1.47724
C -0.92337 4.03128 -1.87794
C 1.26786 3.06807 -2.73710
C 1.77991 -1.62763 2.44516
C 3.32796 -1.76519 2.54379
C 1.17237 -3.02983 2.11138
C -2.70541 1.28492 2.55815
C -4.20717 1.42177 2.18412
C -2.11590 2.70855 2.85380
C -3.09199 -2.03379 -1.88477
C -3.68332 -0.91898 -2.79506
C -2.44353 -3.16748 -2.73597
C 3.79547 -0.46422 5.75344
H 3.60768 -1.41952 6.27261
H 4.61358 -0.62363 5.03397
H 4.14148 0.26276 6.50978
C 0.99411 0.59339 6.34878
H 0.79933 -0.31006 6.95285
H 1.42196 1.35650 7.02233
H 0.02037 0.96859 5.99203
C 2.64921 1.81541 4.01381
H 3.40430 1.64641 3.23014
H 1.77182 2.30327 3.55830
H 3.07693 2.53346 4.73668
C 1.30888 -1.14631 3.87064
H 1.29222 -2.01232 4.56466
H 0.25519 -0.81686 3.77616
C -2.66357 0.44733 3.90339
H -2.92548 1.10702 4.75715
H -1.60845 0.15713 4.07535
C -5.52332 -0.74534 4.69267
H -5.58630 0.02197 5.48287
H -6.09610 -0.38244 3.82483
H -6.01162 -1.66048 5.07270
C -2.90088 -1.94965 5.76445
H -2.91442 -1.26960 6.63397
H -3.43720 -2.87075 6.05277
H -1.84696 -2.21886 5.57292
C -3.72964 -2.41093 2.82328
H -4.09057 -1.97027 1.88076
H -2.73702 -2.86094 2.64941
H -4.40981 -3.24130 3.08461
C -6.86252 -4.04705 -0.25255
H -6.40306 -4.32915 0.71087
H -7.35681 -3.07009 -0.11122
H -7.64681 -4.78975 -0.48091
C -4.81419 -5.72300 -1.84444
H -5.60508 -6.44839 -2.10471
H -4.04631 -5.75316 -2.63444
H -4.34594 -6.06046 -0.90324
C -4.20325 -2.73790 -1.01549
H -4.71197 -1.95741 -0.41707
H -3.63557 -3.33450 -0.27404
C -6.44320 -3.50178 -3.26360
H -7.21605 -4.25844 -3.48775
H -6.94110 -2.52069 -3.19789
H -5.74788 -3.46928 -4.11764
C -0.04728 7.08627 -0.22891
H -0.73767 6.98937 -1.08213
H -0.59169 6.78562 0.68135
H 0.23064 8.15025 -0.12686
C 2.66393 6.32081 1.03882
H 2.17570 6.00640 1.97765
H 3.60748 5.75518 0.94228
H 2.92164 7.39015 1.13375
C 2.42234 6.61285 -2.02654
H 2.56247 7.70692 -1.96401
H 3.41792 6.15285 -2.13627
H 1.85208 6.39857 -2.94473
C 1.08463 4.12801 -0.37132
H 2.00851 3.59449 -0.06374
H 0.40478 4.08261 0.50501
C -0.03339 -5.47742 1.25808
H -0.49452 -6.41542 0.93390
C -0.62004 -4.70985 2.27514
H -1.54266 -5.05062 2.75721
C -0.02205 -3.50559 2.69613
H -0.48380 -2.94656 3.51702
C 1.16536 -5.03109 0.67498
H 1.65390 -5.62826 -0.10273
C 1.75189 -3.82982 1.09795
H 2.71791 -3.53769 0.66638

C	3.93632	-2.84834	3.21732
H	3.30988	-3.65800	3.60742
C	5.32610	-2.89911	3.40084
H	5.77254	-3.74540	3.93436
C	6.14356	-1.86023	2.92060
H	7.22679	-1.89146	3.07760
C	5.55537	-0.78216	2.24199
H	6.17980	0.03807	1.87276
C	4.16188	-0.74458	2.04835
H	3.68729	0.11199	1.55375
C	3.41282	3.15779	-3.93617
H	4.47593	3.42535	-3.93033
C	2.81327	2.67012	-5.10644
H	3.39948	2.54609	-6.02357
C	1.44320	2.35098	-5.08543
H	0.95682	1.96250	-5.98771
C	2.64901	3.34609	-2.76725
H	3.14792	3.74089	-1.87812
C	0.69137	2.54076	-3.91780
H	-0.37741	2.30176	-3.92131
C	-1.50684	-4.01892	-2.11315
H	-1.21384	-3.80677	-1.07997
C	-0.95835	-5.12014	-2.78837
H	-0.24905	-5.77642	-2.27011
C	-1.31533	-5.38405	-4.12311
H	-0.88553	-6.24007	-4.65467
C	-2.23118	-4.53565	-4.76393
H	-2.51674	-4.72238	-5.80545
C	-2.79265	-3.44568	-4.07413
H	-3.51143	-2.79949	-4.58814
C	-2.83698	-0.28952	-3.74156
H	-1.83213	-0.69238	-3.89750
C	-3.26360	0.81643	-4.49168
H	-2.58621	1.27075	-5.22375
C	-4.55751	1.33956	-4.31132
H	-4.89094	2.20749	-4.88921
C	-5.41230	0.72925	-3.38333
H	-6.42588	1.11764	-3.23253
C	-4.97954	-0.38623	-2.64174
H	-5.67355	-0.84837	-1.93496
C	-2.07324	3.88019	-1.07611
H	-2.00821	3.25081	-0.18151
C	-3.26703	4.56119	-1.36487
H	-4.14249	4.42144	-0.72035
C	-3.33935	5.41928	-2.47446
H	-4.27019	5.94697	-2.70878
C	-2.19923	5.59804	-3.27374
H	-2.23470	6.27154	-4.13783
C	-1.00667	4.91568	-2.97534
H	-0.13143	5.06771	-3.61449
C	-4.72575	0.75075	1.06228
H	-4.04479	0.13828	0.45944
C	-6.09345	0.83009	0.74101
H	-6.47482	0.29771	-0.13657
C	-6.96752	1.59161	1.53117
H	-8.03074	1.65798	1.27628
C	-6.46245	2.26753	2.65575
H	-7.13193	2.86483	3.28486
C	-5.10093	2.17598	2.97905
H	-4.72186	2.70871	3.85842
C	-2.07358	5.14670	2.52985
H	-2.52679	6.03830	2.08323
C	-2.65031	3.88790	2.29134
H	-3.55315	3.82732	1.67753
C	-0.93175	5.26586	3.33676
H	-0.48955	6.24758	3.53554
C	-0.36991	4.10400	3.89239
H	0.51484	4.17363	4.53487
C	-0.95461	2.84963	3.64883
H	-0.52018	1.96452	4.12477
Na	0.28467	1.34291	1.22549
Na	2.58267	-1.16135	-0.50214
Na	-1.97571	0.89240	-0.96349
Na	-1.23937	-1.40308	0.97907
N	4.93430	-1.36319	-1.97312
N	6.41923	2.14557	-1.30890
N	6.34749	-4.64328	-0.59152
N	1.98480	-1.46094	-2.83495
C	5.97274	-0.36363	-1.60287
H	6.81627	-0.40720	-2.33167
H	6.38089	-0.64514	-0.61811
C	5.51331	-2.73545	-1.91972
H	4.76042	-3.41485	-2.35464
C	4.43695	-1.11543	-3.36636
H	4.51664	-2.05421	-3.94194
H	5.09820	-0.39523	-3.88844
C	2.99983	-0.58607	-3.45994
H	2.92360	0.40895	-2.98494
H	2.76912	-0.42174	-4.53896
C	0.67375	-0.77294	-2.85443
H	0.72921	0.19922	-2.32511
H	-0.09751	-1.38866	-2.35761
H	0.34627	-0.56690	-3.89815
C	1.86648	-2.74730	-3.54483
H	1.64558	-2.60217	-4.62730
H	1.04308	-3.33260	-3.10783
H	2.79548	-3.33563	-3.45955
C	5.43061	1.08407	-1.52485
H	4.89040	1.33661	-2.45416
H	4.68583	1.14684	-0.70565
C	7.36224	2.32296	-2.41129
H	7.90545	3.27483	-2.27559
H	6.81006	2.38129	-3.36499
H	8.12668	1.51533	-2.50667
C	7.08279	2.11302	-0.01006
H	6.32490	2.08055	0.79225
H	7.66840	3.03962	0.12288
H	7.78509	1.25634	0.13706
C	5.87985	-3.25095	-0.51797
H	6.70074	-2.64804	-0.09549
H	5.01599	-3.12797	0.18145
C	5.24438	-5.59869	-0.71539
H	4.56752	-5.59942	0.17456
H	5.65020	-6.61732	-0.83581
H	4.63587	-5.38048	-1.60821
C	7.19477	-4.99534	0.55033
H	7.57980	-6.02113	0.41834
H	6.65987	-4.95101	1.52885
H	8.05897	-4.31168	0.59995
H	6.41993	-2.81104	-2.56262

E^{Na}

SCF (BP86) Energy = -3653.78945458
Enthalpy 0K = -3652.082304
Enthalpy 298K = -3651.962256
Free Energy 298K = -3652.259101
Lowest Frequency = 5.4033 cm^{-1}
Second Frequency = 6.0915 cm^{-1}

```
SCF (BP86-D3BJ) Energy =
                    -3654.36262300
SCF (C6H6) Energy = -3653.81022815
SCF (BS2) Energy = -5445.54226405

Si  1.15376  6.59448 -0.29600
Si -3.17986 -0.60260 -3.07233
Si  2.33992 -3.72529 -3.76263
Si  5.47084 -4.44576  3.04450
O   2.31517  2.48160 -0.39301
O  -4.39416 -0.96923 -0.34686
O   2.28173 -0.49186 -2.00982
O   3.56780 -1.20292  0.98798
C   1.96133  3.62743  0.31408
C   3.19719  4.22655  1.04697
C   0.84760  3.20322  1.31021
C  -3.87136 -2.19487 -0.71599
C  -4.91195 -3.27488 -0.23947
C  -2.49783 -2.48421 -0.03392
C   2.65525 -0.67179 -3.34851
C   4.17324 -0.94637 -3.52841
C   2.30656  0.66336 -4.09356
C   4.48288 -1.64731  1.93927
C   5.82413 -0.91621  1.65498
C   4.01311 -1.32530  3.39096
C  -4.68036  0.56534 -3.20488
H  -4.42262  1.50722 -3.72073
H  -5.50112  0.08642 -3.76756
H  -5.03815  0.78231 -2.18670
C  -2.60943 -1.00853 -4.85626
H  -3.39743 -1.54305 -5.41555
H  -2.36970 -0.08880 -5.41896
H  -1.71125 -1.65098 -4.85701
C  -1.72869  0.31730 -2.22767
H  -2.00984  0.63486 -1.21164
H  -0.85017 -0.35103 -2.17116
H  -1.47906  1.20108 -2.84530
C  -3.65314 -2.27489 -2.27045
H  -4.59754 -2.55710 -2.77492
H  -2.92532 -3.05624 -2.56067
C   1.88202 -1.87289 -4.01397
H   1.91294 -1.76982 -5.11916
H   0.81403 -1.77523 -3.73471
C   3.91327 -4.24182 -4.70016
H   3.86235 -3.94399 -5.76105
H   4.81695 -3.78466 -4.26677
H   4.02698 -5.33987 -4.66375
C   0.86196 -4.69559 -4.49083
H   0.70076 -4.44078 -5.55265
H   1.03527 -5.78436 -4.43164
H  -0.07251 -4.47224 -3.94713
C   2.53011 -4.29351 -1.94424
H   3.36233 -3.78239 -1.42931
H   1.58857 -4.18238 -1.37465
H   2.76852 -5.37179 -1.92996
C   5.57467 -6.09755  2.07989
H   4.57459 -6.42521  1.74529
H   6.21268 -6.00259  1.18376
H   5.99676 -6.90139  2.70804
C   4.38729 -4.74449  4.58569
H   4.82953 -5.52851  5.22533
H   4.27589 -3.82903  5.18850
H   3.37723 -5.08140  4.29339
C   4.60892 -3.21707  1.82945
H   4.97030 -3.44426  0.80654
H   3.55596 -3.56826  1.84367
C   7.24227 -3.99979  3.60106
H   7.63306 -4.80295  4.25094
H   7.93147 -3.89436  2.74718
H   7.27366 -3.05971  4.17448
C   2.78992  7.57301 -0.25535
H   3.44144  7.23146  0.56541
H   3.34646  7.45088 -1.20066
H   2.59682  8.65129 -0.11592
C   0.10423  7.26173 -1.75438
H   0.61742  7.10027 -2.71859
H  -0.87649  6.75661 -1.80878
H  -0.08240  8.34506 -1.65183
C   0.18901  6.93306  1.31581
H  -0.04165  8.01057  1.39209
H  -0.76052  6.37505  1.35681
H   0.77862  6.65184  2.20311
C   1.47535  4.73571 -0.70091
H   0.57309  4.35421 -1.22352
H   2.26334  4.75339 -1.47881
C  -0.08106 -2.97600  1.37518
H   0.81707 -3.18785  1.96904
C  -0.60593 -3.95041  0.50024
H  -0.10464 -4.91989  0.39544
C  -1.79398 -3.69677 -0.20633
H  -2.20183 -4.47268 -0.86530
C  -0.75056 -1.74499  1.51837
H  -0.35168 -0.97092  2.18492
C  -1.95303 -1.51454  0.82628
H  -2.51743 -0.58289  0.93825
C  -6.17420 -3.34762 -0.88543
H  -6.34135 -2.76441 -1.79800
C  -7.21623 -4.14699 -0.38995
H  -8.16881 -4.19139 -0.93067
C  -7.03540 -4.90545  0.78277
H  -7.84056 -5.54339  1.16250
C  -5.79670 -4.84778  1.44009
H  -5.62797 -5.44463  2.34442
C  -4.75520 -4.04322  0.93822
H  -3.79690 -4.02368  1.46604
C  -1.51911  2.92095  1.93428
H  -2.57366  3.15009  1.72981
C  -1.15563  2.11735  3.02526
H  -1.92164  1.70982  3.69516
C   0.20655  1.84955  3.25920
H   0.51417  1.22960  4.10862
C  -0.52340  3.45818  1.09588
H  -0.83497  4.09521  0.26210
C   1.18671  2.37568  2.40686
H   2.24024  2.14756  2.59923
C   2.63474 -1.24251  3.65969
H   1.94980 -1.31930  2.81005
C   2.15642 -1.03821  4.96424
H   1.07633 -0.99774  5.14944
C   3.05727 -0.87886  6.03049
H   2.68868 -0.70909  7.04798
C   4.43754 -0.92617  5.77379
H   5.15335 -0.78514  6.59162
C   4.90717 -1.15168  4.46866
H   5.98594 -1.17733  4.28161
C   5.87854  0.48450  1.86078
H   5.02495  0.98631  2.33268
C   7.00936  1.23673  1.51072
```

H	7.01028	2.31808	1.68532
C	8.13159	0.60242	0.94647
H	9.01999	1.18339	0.67622
C	8.10180	-0.78480	0.74650
H	8.97290	-1.29618	0.32038
C	6.95996	-1.53181	1.09382
H	6.96426	-2.61239	0.92426
C	4.43875	4.18913	0.37853
H	4.47666	3.70052	-0.60147
C	5.58758	4.76817	0.93909
H	6.53596	4.74023	0.38960
C	5.52358	5.38476	2.20154
H	6.41921	5.83181	2.64650
C	4.29819	5.41929	2.88564
H	4.23441	5.88983	3.87342
C	3.14699	4.85183	2.31013
H	2.19952	4.88422	2.85774
C	4.97881	-1.30676	-2.43149
H	4.53723	-1.37478	-1.42826
C	6.34647	-1.59183	-2.60653
H	6.95246	-1.85501	-1.73375
C	6.93158	-1.51645	-3.87948
H	7.99720	-1.73230	-4.01296
C	6.13774	-1.15310	-4.98165
H	6.58208	-1.08535	-5.98110
C	4.77547	-0.87264	-4.80466
H	4.16853	-0.57954	-5.66922
C	2.80955	3.04332	-4.46629
H	3.49910	3.88628	-4.34912
C	3.16299	1.78307	-3.95998
H	4.12898	1.65622	-3.46032
C	1.57699	3.22650	-5.11772
H	1.29927	4.20866	-5.51421
C	0.71168	2.13007	-5.26067
H	-0.24748	2.25040	-5.77724
C	1.07438	0.86594	-4.75681
H	0.38517	0.02720	-4.89307
Na	1.03727	1.45411	-1.82975
Na	-6.15785	-1.00647	0.91659
Na	3.62855	0.70686	-0.19661
Na	1.98861	-1.93984	-0.32949
N	-7.69513	0.98607	1.43740
N	-4.94407	3.66632	0.89757
N	-9.97024	0.29452	-1.53356
N	-6.63584	-1.19671	3.34389
C	-7.07575	2.26397	0.98303
H	-7.65689	3.12321	1.38912
H	-7.17132	2.30782	-0.11500
C	-8.98228	0.78229	0.71219
H	-9.59825	0.08757	1.30802
C	-7.89193	0.97115	2.91534
H	-8.87260	0.51397	3.13215
H	-7.94976	2.00641	3.31515
C	-6.80957	0.22287	3.71821
H	-5.83123	0.71401	3.57890
H	-7.06403	0.32624	4.80110
C	-5.43347	-1.74508	4.00423
H	-4.54404	-1.16420	3.70865
H	-5.28383	-2.78724	3.68012
H	-5.51388	-1.72488	5.11465
C	-7.80763	-2.01446	3.70718
H	-8.00642	-2.00418	4.80347
H	-7.63291	-3.05522	3.38991
H	-8.71128	-1.65348	3.18992
C	-5.57752	2.42116	1.34288
H	-5.44794	2.37601	2.43993
H	-5.00600	1.57795	0.90619
C	-5.43140	4.87069	1.56848
H	-4.75690	5.71253	1.33481
H	-5.41445	4.71960	2.66168
H	-6.46250	5.18085	1.27675
C	-4.87298	3.82414	-0.55482
H	-4.46824	2.90435	-1.00843
H	-4.18718	4.65520	-0.79474
H	-5.85002	4.05204	-1.04305
C	-8.77269	0.19358	-0.69723
H	-7.96355	0.74488	-1.20952
H	-8.42197	-0.86987	-0.60437
C	-11.06003	-0.55768	-1.05144
H	-10.78865	-1.64238	-1.02664
H	-11.93453	-0.44156	-1.71278
H	-11.37104	-0.25743	-0.03756
C	-9.65926	-0.01295	-2.93224
H	-10.56011	0.13682	-3.55082
H	-9.30807	-1.06220	-3.08628
H	-8.87374	0.66813	-3.29984
H	-9.55557	1.73097	0.61836

TS(E-F)^{Na}

SCF (BP86) Energy = -3653.75613754
Enthalpy 0K = -3652.050914
Enthalpy 298K = -3651.931090
Free Energy 298K = -3652.227286
Lowest Frequency = -394.9436 cm^{-1}
Second Frequency = 7.3781 cm^{-1}
SCF (BP86-D3BJ) Energy = -3654.33355657
SCF (C6H6) Energy = -3653.77727228
SCF (BS2) Energy = -5445.50963006

Si	0.64053	6.41628	-1.36146
Si	-3.46002	-0.14103	-2.36837
Si	3.37213	-3.88335	-3.43046
Si	4.78808	-4.00895	3.87270
O	1.64598	2.35068	-0.50702
O	-3.73884	-1.22515	-1.06177
O	2.53377	-0.57499	-1.97924
O	3.14262	-1.00219	1.28442
C	1.30278	3.62760	-0.07605
C	2.55073	4.34288	0.52900
C	0.17707	3.48240	0.98746
C	-3.17986	-2.77651	-1.75359
C	-4.47373	-3.54848	-1.54658
C	-2.05545	-3.20719	-0.81855
C	3.16945	-0.79048	-3.20970
C	4.71713	-0.84201	-3.09514
C	2.78500	0.42093	-4.12783
C	3.87274	-1.32112	2.42548
C	5.21164	-0.53490	2.35196
C	3.12029	-0.91453	3.72936
C	-4.15713	1.44341	-1.45491
H	-4.06666	2.32544	-2.11745
H	-5.23585	1.35162	-1.21503
H	-3.60292	1.68452	-0.52819
C	-4.53801	-0.11715	-3.95600
H	-5.42938	-0.75425	-3.81967
H	-4.89255	0.90711	-4.17513
H	-3.98582	-0.49999	-4.82810

C	-1.63781	0.40877	-2.66542
H	-1.04223	-0.07819	-1.87170
H	-1.26440	0.07428	-3.64634
H	-1.55517	1.50955	-2.57553
C	-2.73736	-2.48083	-3.08331
H	-3.34479	-2.78551	-3.93990
H	-1.65870	-2.47544	-3.26799
C	2.70031	-2.13669	-3.88377
H	2.90284	-2.09472	-4.97493
H	1.59757	-2.18843	-3.78839
C	5.13321	-4.19392	-4.08101
H	5.21377	-3.94627	-5.15289
H	5.88189	-3.59274	-3.54113
H	5.39200	-5.26109	-3.96181
C	2.18222	-5.09990	-4.30029
H	2.15564	-4.92274	-5.38945
H	2.49784	-6.14561	-4.13986
H	1.15202	-4.99382	-3.91838
C	3.35952	-4.33502	-1.56896
H	3.98849	-3.65793	-0.96459
H	2.33621	-4.39632	-1.15322
H	3.79055	-5.34509	-1.45139
C	5.11659	-5.69342	3.02129
H	4.20169	-6.08325	2.54132
H	5.89246	-5.60545	2.24066
H	5.45890	-6.44858	3.75027
C	3.48473	-4.30112	5.23481
H	3.85238	-5.03706	5.97137
H	3.23697	-3.37032	5.76916
H	2.55055	-4.69963	4.80146
C	4.08384	-2.88620	2.46647
H	4.62382	-3.15851	1.53713
H	3.05942	-3.29219	2.33055
C	6.43104	-3.46202	4.67841
H	6.74223	-4.22261	5.41658
H	7.24112	-3.36385	3.93730
H	6.34524	-2.49928	5.20698
C	2.33989	7.26391	-1.53834
H	2.96780	7.09533	-0.64834
H	2.88772	6.87723	-2.41505
H	2.21802	8.35326	-1.67215
C	-0.37163	6.78237	-2.94624
H	0.12222	6.36024	-3.83899
H	-1.38422	6.34572	-2.88636
H	-0.48429	7.86867	-3.10786
C	-0.27810	7.20720	0.11282
H	-0.35475	8.29760	-0.04649
H	-1.29871	6.81009	0.23552
H	0.26124	7.04291	1.05947
C	0.84370	4.49601	-1.31336
H	-0.08719	4.04792	-1.71891
H	1.61838	4.30207	-2.08229
C	0.07831	-4.15444	0.81089
H	0.87809	-4.52763	1.46064
C	-0.48741	-4.98629	-0.17698
H	-0.11363	-6.00678	-0.31499
C	-1.53414	-4.51395	-0.98029
H	-1.96661	-5.15959	-1.75190
C	-0.41708	-2.84833	0.97211
H	0.00145	-2.20149	1.75245
C	-1.47380	-2.38009	0.16262
H	-1.87152	-1.36926	0.28275
C	-5.45897	-3.63332	-2.55717
H	-5.28366	-3.14780	-3.52086
C	-6.65524	-4.33500	-2.35300
H	-7.39110	-4.39148	-3.16293
C	-6.90850	-4.97821	-1.12796
H	-7.83453	-5.54318	-0.97749
C	-5.94519	-4.90229	-0.10981
H	-6.11434	-5.41154	0.84618
C	-4.74882	-4.19468	-0.31663
H	-3.99630	-4.16795	0.47896
C	-2.15274	3.66592	1.76090
H	-3.17478	4.02750	1.59058
C	-1.82803	3.01369	2.96004
H	-2.58906	2.86645	3.73584
C	-0.50478	2.58138	3.17082
H	-0.22629	2.07482	4.10123
C	-1.15702	3.89378	0.79089
H	-1.43670	4.41317	-0.12982
C	0.47376	2.80636	2.19403
H	1.49745	2.46261	2.37463
C	1.73003	-0.71323	3.68839
H	1.24750	-0.76423	2.70714
C	1.00297	-0.41663	4.85397
H	-0.08309	-0.27762	4.79779
C	1.66382	-0.28255	6.08563
H	1.10187	-0.04281	6.99496
C	3.05964	-0.44035	6.13491
H	3.59341	-0.31427	7.08374
C	3.77594	-0.75480	4.96868
H	4.86507	-0.86004	5.01677
C	5.16816	0.87406	2.48879
H	4.21976	1.35176	2.76364
C	6.31262	1.66627	2.31726
H	6.23762	2.75372	2.42495
C	7.54714	1.06395	2.01262
H	8.44678	1.67585	1.88650
C	7.61265	-0.33072	1.88347
H	8.56999	-0.81632	1.66039
C	6.45623	-1.11777	2.04405
H	6.53559	-2.20260	1.92903
C	3.79500	4.14973	-0.10898
H	3.82604	3.49479	-0.98773
C	4.95909	4.78716	0.34653
H	5.90949	4.62620	-0.17542
C	4.90652	5.63110	1.47047
H	5.81268	6.12825	1.83350
C	3.67830	5.82982	2.12090
H	3.62310	6.48084	3.00090
C	2.51365	5.19582	1.65157
H	1.56529	5.35606	2.17439
C	5.34253	-1.00349	-1.84453
H	4.72923	-1.07551	-0.93731
C	6.74427	-1.08415	-1.74460
H	7.20308	-1.19598	-0.75700
C	7.54385	-0.99826	-2.89449
H	8.63501	-1.05354	-2.81542
C	6.93105	-0.83284	-4.14911
H	7.54334	-0.76003	-5.05502
C	5.53416	-0.75701	-4.24476
H	5.06761	-0.61782	-5.22696
C	3.01316	2.82031	-4.62789
H	3.54794	3.76418	-4.47608
C	3.43987	1.66482	-3.95429
H	4.30806	1.71832	-3.28888
C	1.90699	2.76863	-5.49536
H	1.57248	3.66812	-6.02233

C 1.24354 1.54556 -5.68307
H 0.38601 1.48384 -6.36250
C 1.67912 0.38848 -5.00851
H 1.15002 -0.55400 -5.18045
Na 1.03432 1.19959 -2.24402
Na -5.04747 -0.75413 0.63506
Na 3.20279 0.84585 0.01535
Na 2.11005 -2.04768 -0.34277
N -7.04975 0.39951 1.82416
N -6.03830 4.13573 1.51853
N -9.56520 -0.82750 -0.76157
N -4.63293 -1.13409 3.06358
C -7.21388 1.86695 1.60466
H -8.06305 2.23888 2.22289
H -7.50159 2.01334 0.55013
C -8.26307 -0.29968 1.30351
H -8.30101 -1.29697 1.77402
C -6.84712 0.09627 3.27117
H -7.37273 -0.84574 3.50386
H -7.32789 0.87204 3.90631
C -5.37854 -0.03449 3.71648
H -4.83235 0.89792 3.49938
H -5.37253 -0.15491 4.82673
C -3.18941 -0.99448 3.35758
H -2.82276 -0.01504 3.00612
H -2.62807 -1.78521 2.83419
H -2.97499 -1.07566 4.44686
C -5.09523 -2.45543 3.53097
H -4.97629 -2.57551 4.63191
H -4.51038 -3.24477 3.03215
H -6.15647 -2.61266 3.27882
C -5.95065 2.71882 1.88020
H -5.69775 2.68327 2.95546
H -5.09569 2.28447 1.32648
C -6.94212 4.92037 2.35745
H -6.77984 5.99413 2.15862
H -6.71607 4.73674 3.42219
H -8.02617 4.71315 2.19256
C -6.23466 4.39222 0.09194
H -5.52024 3.79044 -0.49424
H -6.03625 5.45855 -0.11356
H -7.26389 4.17171 -0.28004
C -8.24930 -0.46408 -0.22939
H -7.95723 0.49134 -0.70133
H -7.46854 -1.21615 -0.51999
C -9.97814 -2.17173 -0.34157
H -9.25882 -2.96574 -0.65570
H -10.96224 -2.40273 -0.78208
H -10.09002 -2.21900 0.75426
C -9.57612 -0.72484 -2.22470
H -10.58959 -0.94559 -2.59994
H -8.86328 -1.42570 -2.72078
H -9.31568 0.30296 -2.52931
H -9.19514 0.23535 1.59153

F^{Na}
SCF (BP86) Energy = -3653.82209798
Enthalpy 0K = -3652.115756
Enthalpy 298K = -3651.994730
Free Energy 298K = -3652.294714
Lowest Frequency = 5.9624 cm^{-1}
Second Frequency = 8.0628 cm^{-1}
SCF (BP86-D3BJ) Energy =
-3654.39220298
SCF (C6H6) Energy = -3653.84328593
SCF (BS2) Energy = -5445.57943322

Si -0.85538 6.58242 0.88449
Si 3.66846 0.43206 2.19963
Si -3.02607 -3.74889 3.68647
Si -5.11590 -4.22235 -3.44778
O -1.96905 2.47801 0.44067
O 4.13622 -0.41786 0.88586
O -2.38049 -0.49939 2.03096
O -3.32844 -1.09247 -1.11163
C -1.61323 3.70661 -0.10795
C -2.84459 4.37678 -0.78819
C -0.47325 3.42979 -1.12737
C 3.02745 -4.35168 1.66954
C 4.46011 -4.11084 1.33170
C 1.98995 -3.94829 0.67048
C -2.89044 -0.66655 3.32441
C -4.44162 -0.74363 3.35954
C -2.43439 0.58304 4.15423
C -4.12884 -1.47194 -2.18508
C -5.47210 -0.70235 -2.04608
C -3.47169 -1.11281 -3.55266
C 4.55771 2.12536 2.38751
H 4.22233 2.67299 3.28722
H 5.65184 1.99056 2.46975
H 4.35453 2.76413 1.50959
C 3.92282 -0.50163 3.85817
H 4.99173 -0.73437 4.01177
H 3.58161 0.09433 4.72442
H 3.36680 -1.45609 3.86184
C 1.78407 0.86398 2.13089
H 1.59204 1.48412 1.23578
H 1.20931 -0.07863 2.05976
H 1.48397 1.41273 3.04414
C 2.67704 -4.95990 2.82928
H 3.42799 -5.31490 3.54159
H 1.62654 -5.11899 3.09066
C -2.34156 -1.97973 4.00649
H -2.43990 -1.89276 5.10926
H -1.25199 -2.02317 3.80876
C -4.74173 -4.04872 4.45118
H -4.76204 -3.75373 5.51374
H -5.52783 -3.47922 3.93059
H -4.99355 -5.12269 4.39448
C -1.77623 -4.91132 4.54986
H -1.70761 -4.69243 5.62948
H -2.07486 -5.96852 4.44103
H -0.76437 -4.79840 4.12249
C -3.10477 -4.29730 1.85216
H -3.79887 -3.67920 1.25577
H -2.10678 -4.33524 1.37571
H -3.49629 -5.32915 1.80994
C -5.36444 -5.87390 -2.50872
H -4.41205 -6.24079 -2.08705
H -6.07756 -5.75849 -1.67370
H -5.75722 -6.65864 -3.17859
C -3.91814 -4.56174 -4.89340
H -4.33556 -5.32733 -5.57085
H -3.71821 -3.65164 -5.48084
H -2.95092 -4.93948 -4.51768
C -4.31743 -3.03987 -2.14518
H -4.78148 -3.27936 -1.16717
H -3.28103 -3.43083 -2.07050

C	-6.81992	-3.71442	-4.14387
H	-7.17877	-4.49952	-4.83310
H	-7.57226	-3.59899	-3.34655
H	-6.78329	-2.76753	-4.70559
C	-2.50448	7.54037	0.91969
H	-3.12954	7.29578	0.04542
H	-3.08409	7.29723	1.82705
H	-2.32278	8.62960	0.91917
C	0.14740	7.07363	2.44119
H	-0.38661	6.78712	3.36411
H	1.13305	6.57579	2.45643
H	0.32137	8.16324	2.47938
C	0.14328	7.13114	-0.64668
H	0.35073	8.21434	-0.58516
H	1.10690	6.60204	-0.72507
H	-0.41469	6.94841	-1.57914
C	-1.16250	4.68448	1.04690
H	-0.26631	4.24939	1.53567
H	-1.96727	4.60083	1.80353
C	0.03050	-3.21156	-1.23571
H	-0.69927	-2.93500	-2.00613
C	0.03324	-4.51055	-0.69264
H	-0.70235	-5.24883	-1.03018
C	1.00316	-4.87223	0.25762
H	1.02255	-5.88938	0.66408
C	0.99448	-2.27676	-0.80399
H	0.98653	-1.25703	-1.20717
C	1.98123	-2.63781	0.13104
H	2.73167	-1.88909	0.45852
C	5.39542	-3.73032	2.32048
H	5.04192	-3.52595	3.33523
C	6.75198	-3.56980	2.00615
H	7.45660	-3.26451	2.78652
C	7.20696	-3.78549	0.69349
H	8.26956	-3.67561	0.45286
C	6.28641	-4.15636	-0.30459
H	6.63348	-4.36082	-1.32361
C	4.92537	-4.30744	0.00992
H	4.21433	-4.61418	-0.76456
C	1.90662	3.28350	-1.74496
H	2.95528	3.49050	-1.49505
C	1.57442	2.64081	-2.94715
H	2.35707	2.36039	-3.66266
C	0.22275	2.37759	-3.23708
H	-0.05862	1.87650	-4.16978
C	0.88907	3.67564	-0.85443
H	1.17714	4.18759	0.06853
C	-0.77878	2.75572	-2.33318
H	-1.82454	2.53449	-2.57091
C	-2.08307	-0.90164	-3.61600
H	-1.52997	-0.90924	-2.67150
C	-1.44031	-0.64564	-4.83939
H	-0.35474	-0.49539	-4.86255
C	-2.18561	-0.56389	-6.02647
H	-1.68984	-0.35464	-6.98067
C	-3.57990	-0.73337	-5.97338
H	-4.17869	-0.64803	-6.88731
C	-4.21190	-1.00692	-4.74945
H	-5.30086	-1.12051	-4.71901
C	-5.46712	0.69777	-2.25918
H	-4.55518	1.17409	-2.63923
C	-6.60710	1.48163	-2.02809
H	-6.56139	2.56267	-2.19826
C	-7.79811	0.87931	-1.58319
H	-8.69428	1.48444	-1.40883
C	-7.82583	-0.50736	-1.37603
H	-8.75016	-0.99363	-1.04253
C	-6.67387	-1.28548	-1.59864
H	-6.72287	-2.36366	-1.42079
C	-4.10004	4.23365	-0.16073
H	-4.14837	3.63232	0.75432
C	-5.25058	4.85055	-0.67532
H	-6.21040	4.73201	-0.15903
C	-5.17297	5.61936	-1.85037
H	-6.06884	6.09872	-2.25999
C	-3.93272	5.76640	-2.49182
H	-3.85750	6.35879	-3.41092
C	-2.78139	5.15654	-1.96185
H	-1.82287	5.27734	-2.47667
C	-5.17674	-0.98328	2.18287
H	-4.64911	-1.10208	1.22778
C	-6.58014	-1.08726	2.21670
H	-7.12523	-1.25893	1.28277
C	-7.27258	-0.94920	3.42940
H	-8.36526	-1.02227	3.45471
C	-6.55003	-0.70931	4.61146
H	-7.07792	-0.59622	5.56511
C	-5.15170	-0.60953	4.57371
H	-4.60000	-0.41087	5.49986
C	-2.66357	2.98833	4.62132
H	-3.23538	3.91581	4.50885
C	-3.13621	1.80643	4.02879
H	-4.07489	1.82550	3.46567
C	-1.46367	2.98470	5.35407
H	-1.09306	3.90513	5.81688
C	-0.74951	1.78303	5.48805
H	0.18410	1.75890	6.06121
C	-1.23045	0.59992	4.89576
H	-0.65624	-0.32317	5.02086
Na	-0.95923	1.36172	2.01926
Na	5.40116	-1.09413	-0.67143
Na	-3.37926	0.81758	0.07146
Na	-2.08341	-2.01378	0.42428
N	7.39530	0.12095	-1.84547
N	5.61753	3.54916	-1.44683
N	9.97341	0.13587	0.93634
N	4.99289	-1.38921	-3.18627
C	7.20940	1.60645	-1.93632
H	7.38527	1.93552	-2.98102
H	7.99252	2.09567	-1.33110
C	8.68489	-0.20266	-1.17905
H	8.89908	-1.26926	-1.37075
C	7.35453	-0.49994	-3.19269
H	7.69882	-1.54576	-3.09478
H	8.06915	0.00170	-3.88639
C	5.96334	-0.48250	-3.84520
H	5.55497	0.54148	-3.81012
H	6.07359	-0.73592	-4.92513
C	3.60641	-0.97858	-3.49331
H	3.41493	0.03801	-3.11195
H	2.90251	-1.66456	-2.99469
H	3.39326	-0.99363	-4.58587
C	5.19423	-2.77653	-3.64526
H	5.00894	-2.88665	-4.73833
H	4.50519	-3.44899	-3.10904
H	6.22546	-3.10736	-3.43986
C	5.82416	2.09795	-1.44238
H	5.02431	1.65074	-2.06403

H	5.64985	1.72900	-0.41390
C	5.65461	4.16365	-2.77293
H	5.28186	5.20028	-2.70368
H	4.98470	3.61325	-3.45636
H	6.66815	4.20632	-3.23834
C	6.42871	4.27842	-0.47211
H	6.31583	3.81383	0.52164
H	6.06310	5.31781	-0.40339
H	7.51643	4.32782	-0.71847
C	8.63588	0.05460	0.33938
H	8.13194	1.01921	0.52846
H	8.00377	-0.73064	0.82791
C	10.68376	-1.14316	0.88892
H	10.15587	-1.95932	1.44337
H	11.68422	-1.02413	1.33746
H	10.82613	-1.47148	-0.15380
C	9.89963	0.62888	2.31417
H	10.91934	0.74491	2.71886
H	9.33368	-0.04999	2.99726
H	9.40933	1.61657	2.33275
H	9.52727	0.38347	-1.61120

3K

SCF (BP86) Energy = -3072.02869330

Enthalpy 0K = -3070.739148

Enthalpy 298K = -3070.641470

Free Energy 298K = -3070.883762

Lowest Frequency = 9.1472 cm^{-1}

Second Frequency = 9.8746 cm^{-1}

SCF (BP86-D3BJ) Energy = -3072.52468954

SCF (C6H6) Energy = -3072.03711786

SCF (BS2) Energy = -6501.86404660

Si	-5.92076	-1.49600	-1.92671
Si	-3.39254	2.35524	4.09417
Si	3.39267	-2.35645	4.09324
Si	5.92068	1.49670	-1.92626
O	-1.79047	-0.66250	-1.10459
O	-0.78348	1.78210	1.60371
O	0.78351	-1.78270	1.60302
O	1.79039	0.66291	-1.10443
C	-2.85805	-0.84927	-1.98158
C	-2.67581	-2.15610	-2.81297
C	-2.87400	0.41280	-2.89017
C	-0.81245	2.87131	2.46071
C	-1.58069	4.08900	1.86509
C	0.67938	3.28976	2.69948
C	0.81251	-2.87211	2.45976
C	1.58070	-4.08967	1.86382
C	-0.67931	-3.29062	2.69850
C	2.85798	0.84994	-1.98135
C	2.87398	-0.41187	-2.89028
C	2.67574	2.15700	-2.81238
C	-4.31328	4.02003	4.01584
H	-3.85503	4.75535	4.69879
H	-4.30575	4.45331	3.00328
H	-5.36412	3.88322	4.32687
C	-3.59693	1.66052	5.86508
H	-3.16250	2.34351	6.61551
H	-4.66312	1.52441	6.11703
H	-3.09806	0.68177	5.97712
C	-4.24703	1.11626	2.91147
H	-4.03567	1.33880	1.85250
H	-3.94997	0.07590	3.13700
H	-5.34132	1.16599	3.04999
C	-1.49472	2.53446	3.84518
H	-1.22037	3.30420	4.59753
H	-1.04420	1.58803	4.20749
C	1.49483	-2.53559	3.84428
H	1.22052	-3.30551	4.59646
H	1.04433	-1.58925	4.20684
C	4.31337	-4.02124	4.01447
H	3.85511	-4.75673	4.69722
H	4.30582	-4.45424	3.00179
H	5.36422	-3.88453	4.32552
C	3.59713	-1.66216	5.86431
H	3.16269	-2.34531	6.61459
H	4.66333	-1.52617	6.11627
H	3.09831	-0.68342	5.97659
C	4.24715	-1.11722	2.91080
H	4.03571	-1.33948	1.85179
H	3.95020	-0.07689	3.13660
H	5.34145	-1.16707	3.04924
C	7.21098	1.04134	-0.58913
H	6.99263	1.55209	0.36509
H	7.22216	-0.04492	-0.39224
H	8.22887	1.33524	-0.89941
C	6.02438	3.37562	-2.23500
H	7.03208	3.65637	-2.58822
H	5.29248	3.70363	-2.99081
H	5.82679	3.93926	-1.30675
C	4.20307	1.02505	-1.17617
H	4.34468	0.11943	-0.55197
H	3.99492	1.84273	-0.45621
C	6.38559	0.57628	-3.53187
H	7.40058	0.87488	-3.84858
H	6.38130	-0.51797	-3.39954
H	5.69397	0.81561	-4.35571
C	-6.02448	-3.37484	-2.23597
H	-5.29258	-3.70265	-2.99186
H	-5.82690	-3.93873	-1.30787
H	-7.03219	-3.65547	-2.58927
C	-7.21107	-1.04099	-0.58947
H	-6.99273	-1.55199	0.36462
H	-7.22224	0.04523	-0.39229
H	-8.22895	-1.33479	-0.89983
C	-6.38564	-0.57514	-3.53207
H	-7.40065	-0.87361	-3.84885
H	-6.38130	0.51907	-3.39946
H	-5.69404	-0.81429	-4.35599
C	-4.20315	-1.02457	-1.17647
H	-4.34476	-0.11912	-0.55203
H	-3.99503	-1.84246	-0.45673
C	3.49773	3.74400	2.86125
H	4.57539	3.92564	2.92762
C	2.81715	3.09937	3.90716
H	3.36367	2.77972	4.80192
C	1.42778	2.87936	3.82581
H	0.92280	2.38767	4.66319
C	2.76713	4.17864	1.73878
H	3.27549	4.70360	0.92253
C	1.38440	3.95184	1.66504
H	0.82558	4.31123	0.79354
C	-1.42658	5.40143	2.36077
H	-0.71549	5.59023	3.17344
C	-2.15837	6.47004	1.81924
H	-2.01686	7.48191	2.21540
C	-3.06910	6.24748	0.77153
H	-3.63964	7.08121	0.34861
C	-3.24109	4.94482	0.27561
H	-3.95562	4.75450	-0.53329
C	-2.49784	3.88192	0.81830
H	-2.63616	2.85565	0.46092
C	-3.54693	2.73684	-3.34413
H	-4.22522	3.57318	-3.14034
C	-2.52885	2.87700	-4.30024
H	-2.40034	3.81870	-4.84360
C	-1.68509	1.78033	-4.56147
H	-0.89149	1.86688	-5.31204
C	-3.71522	1.52220	-2.65198
H	-4.51962	1.44647	-1.91458
C	-1.85630	0.57513	-3.86334
H	-1.18574	-0.26567	-4.06870
C	2.16304	3.29072	-2.14523
H	1.92036	3.19109	-1.08094
C	1.99096	4.51812	-2.80370
H	1.60186	5.38431	-2.25557
C	2.32282	4.63847	-4.16507
H	2.18400	5.59097	-4.68736

C 2.84164 3.52474 -4.84345
H 3.11003 3.60451 -5.90300
C 3.02235 2.30127 -4.17187
H 3.43132 1.44593 -4.71938
C 1.85632 -0.57395 -3.86354
H 1.18576 0.26691 -4.06871
C 1.68514 -1.77896 -4.56200
H 0.89157 -1.86531 -5.31263
C 2.52888 -2.87570 -4.30103
H 2.40039 -3.81726 -4.84464
C 3.54693 -2.73579 -3.34484
H 4.22522 -3.57218 -3.14125
C 3.71519 -1.52134 -2.65237
H 4.51957 -1.44580 -1.91492
C -2.16312 -3.29000 -2.14613
H -1.92045 -3.19067 -1.08181
C -1.99104 -4.51722 -2.80494
H -1.60195 -5.38357 -2.25705
C -2.32288 -4.63718 -4.16635
H -2.18406 -5.58954 -4.68891
C -2.84169 -3.52327 -4.84442
H -3.11007 -3.60274 -5.90400
C -3.02241 -2.29998 -4.17250
H -3.43137 -1.44449 -4.71978
C 2.49780 -3.88238 0.81703
H 2.63610 -2.85603 0.45985
C 3.24100 -4.94517 0.27407
H 3.95549 -4.75467 -0.53483
C 3.06903 -6.24794 0.76971
H 3.63954 -7.08158 0.34658
C 2.15836 -6.47071 1.81743
H 2.01687 -7.48267 2.21338
C 1.42661 -5.40221 2.35923
H 0.71557 -5.59118 3.17190
C -2.76711 -4.17922 1.73765
H -3.27552 -4.70394 0.92127
C -1.38439 -3.95239 1.66391
H -0.82561 -4.31152 0.79226
C -3.49766 -3.74492 2.86029
H -4.57531 -3.92658 2.92666
C -2.81702 -3.10060 3.90636
H -3.36349 -2.78121 4.80123
C -1.42765 -2.88056 3.82499
H -0.92263 -2.38911 4.66249
K -1.74782 -0.63431 1.50520
K -0.60783 1.77905 -1.14018
K 0.60776 -1.77864 -1.14081
K 1.74781 0.63377 1.50537

3K′
SCF (BP86) Energy = -3072.03104857
Enthalpy 0K = -3070.741579
Enthalpy 298K = -3070.643660
Free Energy 298K = -3070.887748
Lowest Frequency = 10.9292 cm^{-1}
Second Frequency = 11.9384 cm^{-1}
SCF (BP86-D3BJ) Energy = -3072.51651476
SCF (C6H6) Energy = -3072.04062863
SCF (BS2) Energy = -6501.86777634

Si 5.48725 1.98435 2.74835
Si 3.32274 -4.77097 -2.79292
Si -3.32404 -4.76046 2.80907
Si -5.48626 1.97569 -2.75602
O 1.60704 1.26908 1.05536
O 1.08956 -1.31692 -1.56146
O -1.08943 -1.31164 1.56558
O -1.60663 1.26585 -1.05932
C 2.61220 2.12274 1.50053
C 2.16787 2.88606 2.78551
C 2.88418 3.09624 0.31864
C 1.50880 -2.22122 -2.53222
C 2.43603 -1.53684 -3.58047
C 0.20073 -2.75547 -3.18375
C -1.50901 -2.21240 2.53954
C -2.43607 -1.52413 3.58534
C -0.20113 -2.74478 3.19291
C -2.61181 2.11793 -1.50748
C -2.88449 3.09495 -0.32866
C -2.16704 2.87742 -2.79456
C 2.35510 -5.61796 -4.20259
H 1.39689 -6.03699 -3.85409
H 2.13427 -4.91941 -5.02584
H 2.95526 -6.44657 -4.61823
C 3.70322 -6.09137 -1.46079
H 2.77722 -6.53101 -1.05048
H 4.30785 -6.91587 -1.87704
H 4.27020 -5.65588 -0.61915
C 4.98150 -4.11932 -3.46892
H 4.83136 -3.34408 -4.23756
H 5.59034 -3.67917 -2.66012
H 5.56669 -4.94045 -3.91873
C 2.36397 -3.37078 -1.86894
H 1.73468 -3.85255 -1.09294
H 3.15813 -2.83565 -1.30829
C -2.36456 -3.36400 1.88022
H -1.73540 -3.84881 1.10601
H -3.15843 -2.83051 1.31761
C -2.35693 -5.60299 4.22178
H -1.39886 -6.02365 3.87485
H -2.13589 -4.90162 5.04258
H -2.95751 -6.42985 4.64026
C -3.70515 -6.08531 1.48154
H -2.77938 -6.52682 1.07272
H -4.31017 -6.90808 1.90064
H -4.27193 -5.65245 0.63841
C -4.98259 -4.10573 3.48263
H -4.83232 -3.32786 4.24859
H -5.59117 -3.66821 2.67221
H -5.56812 -4.92509 3.93522
C -6.82609 0.63501 -2.49199
H -6.49845 -0.34017 -2.89350
H -7.05362 0.49645 -1.42048
H -7.76789 0.90455 -3.00089
C -5.20848 2.17749 -4.63151
H -6.14154 2.49159 -5.13128
H -4.43358 2.93083 -4.84738
H -4.88920 1.22464 -5.08822
C -3.89341 1.28660 -1.90558
H -4.20968 0.70962 -1.01253
H -3.52131 0.52916 -2.62590
C -6.14433 3.61929 -2.04470
H -7.08147 3.89547 -2.55963
H -6.35940 3.55006 -0.96592
H -5.42733 4.44351 -2.18994
C 5.21031 2.19228 4.62329
H 4.43539 2.94618 4.83706

H	4.89146	1.24089	5.08333
H	6.14358	2.50820	5.12149
C	6.82677	0.64260	2.48824
H	6.49902	-0.33121	2.89294
H	7.05407	0.50056	1.41713
H	7.76873	0.91358	2.99610
C	6.14512	3.62555	2.03132
H	7.08291	3.90315	2.54430
H	6.35891	3.55288	0.95251
H	5.42847	4.45040	2.17477
C	3.89408	1.29277	1.90060
H	4.21004	0.71314	1.00916
H	3.52229	0.53744	2.62329
C	-2.44440	-3.42852	-4.04173
H	-3.45280	-3.69160	-4.37781
C	-1.72829	-4.28726	-3.19194
H	-2.17748	-5.23032	-2.86029
C	-0.42512	-3.95454	-2.77432
H	0.10593	-4.64701	-2.11450
C	-1.83389	-2.23565	-4.47543
H	-2.36727	-1.56128	-5.15473
C	-0.53827	-1.90802	-4.04731
H	-0.07970	-0.97457	-4.39059
C	2.45695	-1.85240	-4.95516
H	1.76550	-2.60557	-5.34722
C	3.34352	-1.20708	-5.83656
H	3.32985	-1.46337	-6.90194
C	4.24223	-0.24094	-5.35784
H	4.93485	0.25933	-6.04294
C	4.24548	0.07328	-3.98751
H	4.95068	0.81511	-3.59600
C	3.34667	-0.56332	-3.11777
H	3.34389	-0.32570	-2.04749
C	4.02049	3.67071	-1.78828
H	4.85748	3.50788	-2.47670
C	3.04729	4.63752	-2.08462
H	3.11285	5.23125	-3.00210
C	1.99305	4.84295	-1.17393
H	1.23152	5.60324	-1.38140
C	3.93857	2.91445	-0.60327
H	4.71454	2.17007	-0.40202
C	1.91452	4.08006	0.00105
H	1.08226	4.24297	0.69376
C	-1.41386	2.16253	-3.75109
H	-1.17783	1.11342	-3.53650
C	-0.99347	2.75722	-4.95089
H	-0.41986	2.17338	-5.68024
C	-1.31109	4.10076	-5.21879
H	-0.97892	4.57367	-6.14902
C	-2.06418	4.82609	-4.28238
H	-2.32217	5.87287	-4.47917
C	-2.49256	4.21793	-3.08792
H	-3.07990	4.80044	-2.37054
C	-1.91473	4.07940	-0.01332
H	-1.08181	4.23978	-0.70583
C	-1.99393	4.84593	1.15924
H	-1.23230	5.60660	1.36498
C	-3.04899	4.64367	2.06969
H	-3.11511	5.24026	2.98528
C	-4.02228	3.67629	1.77554
H	-4.85987	3.51586	2.46380
C	-3.93967	2.91634	0.59294
H	-4.71573	2.17160	0.39335
C	1.41491	2.17410	3.74439
H	1.17863	1.12441	3.53295
C	0.99501	2.77238	4.94258
H	0.42155	2.19077	5.67382
C	1.31293	4.11665	5.20642
H	0.98113	4.59235	6.13537
C	2.06585	4.83910	4.26765
H	2.32407	5.88641	4.46128
C	2.49373	4.22737	3.07484
H	3.08098	4.80765	2.35559
C	-3.34649	-0.55201	3.11928
H	-3.34377	-0.31816	2.04817
C	-4.24510	0.08783	3.98684
H	-4.95010	0.82850	3.59279
C	-4.24188	-0.22163	5.35826
H	-4.93435	0.28120	6.04163
C	-3.34340	-1.18633	5.84030
H	-3.32978	-1.43893	6.90656
C	-2.45700	-1.83489	4.96111
H	-1.76571	-2.58686	5.35575
C	1.83372	-2.22104	4.48264
H	2.36735	-1.54444	5.15953
C	0.53816	-1.89454	4.05348
H	0.07991	-0.95974	4.39350
C	2.44384	-3.41562	4.05311
H	3.45219	-3.67781	4.39005
C	1.72741	-4.27714	3.20639
H	2.17629	-5.22151	2.87807
C	0.42433	-3.94548	2.78768
H	-0.10698	-4.64006	2.13029
K	1.56760	-1.34312	1.01520
K	0.99523	1.36078	-1.59232
K	-0.99463	1.36617	1.58775
K	-1.56758	-1.34639	-1.01074

3K·κ^1L

SCF (BP86) Energy = -3766.30464556
Enthalpy 0K = -3764.599333
Enthalpy 298K = -3764.478627
Free Energy 298K = -3764.773155
Lowest Frequency = 6.5972 cm^{-1}
Second Frequency = 10.8041 cm^{-1}
SCF (BP86-D3BJ) Energy = -3766.90867106
SCF (C6H6) Energy = -3766.31359417
SCF (BS2) Energy = -7196.30705660

Si	1.36262	5.46399	-2.36528
Si	0.65076	2.59105	4.85140
Si	-6.24366	-0.39931	2.97716
Si	-4.02801	-5.44165	-1.21401
O	-0.27853	1.63839	-1.31213
O	-0.07327	0.12481	2.17846
O	-3.56113	0.85307	0.78393
O	-1.51807	-1.96809	-0.83555
C	0.33897	2.48982	-2.22597
C	-0.66254	2.92260	-3.34889
C	1.53614	1.72774	-2.85944
C	0.38721	-0.18350	3.44964
C	1.94223	-0.19791	3.52963
C	-0.16667	-1.60765	3.79797
C	-4.65159	1.59951	1.20365
C	-5.86035	1.49824	0.22632
C	-4.17779	3.09281	1.26709
C	-1.99076	-3.06654	-1.55149

C	-2.46050	-2.51942	-2.92928
C	-0.89820	-4.16363	-1.71993
C	2.35414	2.51080	5.69761
H	2.31503	1.87661	6.59958
H	3.13211	2.10268	5.03346
H	2.66427	3.52266	6.01346
C	-0.56129	3.43324	6.06977
H	-0.65561	2.85418	7.00476
H	-0.21327	4.44567	6.33940
H	-1.57155	3.53011	5.63459
C	0.76938	3.74081	3.32426
H	1.31515	3.27920	2.48489
H	-0.23050	4.06784	2.98570
H	1.31729	4.65753	3.60568
C	-0.10562	0.84877	4.54435
H	-0.01718	0.39066	5.55268
H	-1.19241	1.00083	4.38180
C	-5.17952	1.16275	2.62904
H	-5.80308	1.97521	3.05910
H	-4.29443	1.08490	3.29296
C	-8.01532	-0.27051	2.29249
H	-8.49952	0.66354	2.62411
H	-8.03811	-0.28625	1.19139
H	-8.62304	-1.11435	2.66450
C	-6.36231	-0.49365	4.88451
H	-6.83889	0.41054	5.30129
H	-6.96442	-1.36310	5.20123
H	-5.36517	-0.58893	5.34919
C	-5.47282	-2.04947	2.39220
H	-5.22766	-2.04056	1.31721
H	-4.56832	-2.30002	2.97598
H	-6.19079	-2.87161	2.55821
C	-5.68060	-5.44563	-0.25033
H	-5.51143	-5.29305	0.82986
H	-6.35509	-4.64430	-0.59927
H	-6.21099	-6.40598	-0.37375
C	-2.97889	-6.90758	-0.59377
H	-3.50033	-7.86318	-0.77828
H	-1.99907	-6.94890	-1.09646
H	-2.79917	-6.82914	0.49255
C	-3.17005	-3.76622	-0.76388
H	-3.96790	-3.01136	-0.60775
H	-2.75235	-3.96554	0.24432
C	-4.42943	-5.70238	-3.06138
H	-4.97199	-6.65636	-3.18552
H	-5.06358	-4.89846	-3.46917
H	-3.51654	-5.75458	-3.67581
C	-0.15038	6.42745	-3.01340
H	-0.65199	5.89288	-3.83632
H	-0.89212	6.58445	-2.21125
H	0.15620	7.42086	-3.38548
C	2.17628	6.50319	-0.97973
H	1.47832	6.65956	-0.13858
H	3.07736	6.00748	-0.57822
H	2.47906	7.49768	-1.35136
C	2.62021	5.29899	-3.79282
H	2.81489	6.29921	-4.21913
H	3.58580	4.88385	-3.46107
H	2.23861	4.65757	-4.60343
C	0.79983	3.82155	-1.51033
H	1.55200	3.55220	-0.74133
H	-0.09417	4.15259	-0.94252
C	-1.33003	-4.20584	4.07103
H	-1.76878	-5.20266	4.18323
C	-1.87215	-3.10627	4.75448
H	-2.73886	-3.24148	5.41165
C	-1.29390	-1.82784	4.62150
H	-1.72915	-0.99494	5.18221
C	-0.19679	-4.01068	3.25862
H	0.25237	-4.86200	2.73512
C	0.36564	-2.73280	3.12335
H	1.25236	-2.59698	2.49552
C	2.66194	-0.89448	4.52372
H	2.11879	-1.50715	5.25267
C	4.06451	-0.82618	4.58348
H	4.60264	-1.39012	5.35385
C	4.77973	-0.03499	3.66684
H	5.87480	0.01339	3.70190
C	4.06886	0.67724	2.68398
H	4.60838	1.31549	1.97441
C	2.66956	0.57588	2.60629
H	2.10012	1.10816	1.83627
C	3.92522	1.31650	-3.26032
H	4.96651	1.63033	-3.12233
C	3.63087	0.17700	-4.02280
H	4.43444	-0.40248	-4.48974
C	2.28552	-0.20591	-4.18594
H	2.03586	-1.09255	-4.78038
C	2.88972	2.07767	-2.68444
H	3.15065	2.96827	-2.10674
C	1.26159	0.55473	-3.60461
H	0.21843	0.25528	-3.74972
C	-0.05713	-4.42844	-0.61916
H	-0.16624	-3.80096	0.27263
C	0.87762	-5.47515	-0.65052
H	1.50044	-5.68027	0.22822
C	1.01889	-6.26457	-1.80626
H	1.75256	-7.07711	-1.83647
C	0.20842	-5.99632	-2.92004
H	0.31110	-6.59678	-3.83099
C	-0.74570	-4.96354	-2.87164
H	-1.37819	-4.77779	-3.74576
C	-1.48750	-2.06057	-3.85146
H	-0.43008	-2.24716	-3.63617
C	-1.84583	-1.38163	-5.02536
H	-1.06757	-1.05157	-5.72240
C	-3.20001	-1.12072	-5.31051
H	-3.48246	-0.58719	-6.22361
C	-4.18088	-1.56578	-4.41158
H	-5.24083	-1.38482	-4.62295
C	-3.81337	-2.25955	-3.24144
H	-4.60309	-2.60269	-2.56649
C	-1.97864	3.26077	-2.96652
H	-2.23530	3.21724	-1.90240
C	-2.93825	3.67323	-3.90420
H	-3.94942	3.93869	-3.57318
C	-2.60033	3.75462	-5.26699
H	-3.34505	4.07009	-6.00532
C	-1.29363	3.43361	-5.66594
H	-1.01270	3.49758	-6.72339
C	-0.33659	3.02705	-4.71753
H	0.67565	2.77956	-5.05220
C	-5.92850	0.41771	-0.67208
H	-5.11156	-0.31194	-0.64585
C	-7.01889	0.26042	-1.54539
H	-7.05449	-0.59624	-2.22837
C	-8.06407	1.19874	-1.54051
H	-8.91293	1.08619	-2.22335

C	-8.01052	2.28350	-0.64777
H	-8.82054	3.02144	-0.63435
C	-6.92267	2.42729	0.22790
H	-6.89145	3.28055	0.91547
C	-3.42798	5.09612	0.04430
H	-3.33046	5.63304	-0.90570
C	-4.01766	3.82257	0.06498
H	-4.38691	3.38424	-0.86889
C	-2.97211	5.68572	1.23807
H	-2.51979	6.68274	1.22868
C	-3.13137	4.98567	2.44530
H	-2.80730	5.43887	3.38915
C	-3.72700	3.70925	2.45663
H	-3.85134	3.19593	3.41521
K	-1.17373	2.20493	1.09983
K	0.84888	-0.68935	-0.38429
K	-2.56782	0.37586	-1.74671
K	-2.32067	-1.29508	1.57575
N	7.35242	-0.89646	-0.36353
N	8.66854	-0.19184	2.57563
N	10.16369	-0.65049	-1.50968
N	3.65924	-2.16718	-0.48284
C	7.31564	0.29739	0.50673
H	6.50205	0.17749	1.24219
H	7.06937	1.20432	-0.09140
C	7.62882	-0.59895	-1.77112
H	7.54736	-1.54786	-2.33264
C	6.20248	-1.80433	-0.18403
H	6.22320	-2.15715	0.86319
H	6.38417	-2.69176	-0.81923
C	4.79590	-1.21248	-0.49437
H	4.56836	-0.42860	0.25011
H	4.79592	-0.72676	-1.48581
C	3.68213	-3.07926	-1.63744
H	3.70071	-2.48830	-2.56832
H	2.76798	-3.69639	-1.63394
H	4.55280	-3.77488	-1.64490
C	3.57434	-2.92141	0.77753
H	4.39775	-3.65763	0.91964
H	2.62785	-3.48971	0.79267
H	3.58527	-2.22384	1.63241
C	8.60039	0.57962	1.31693
H	9.49554	0.40779	0.67716
H	8.58023	1.64938	1.59809
C	9.57444	0.44319	3.53547
H	9.57219	-0.13145	4.47828
H	9.23145	1.46809	3.75943
H	10.63219	0.50153	3.17895
C	9.08657	-1.58021	2.34179
H	8.44827	-2.03731	1.57178
H	8.99709	-2.15643	3.27969
H	10.14166	-1.65593	1.98489
C	8.99671	0.04649	-2.05804
H	9.06128	0.15112	-3.17282
H	9.01108	1.07749	-1.65446
C	11.39133	0.04441	-1.89332
H	11.56814	0.05992	-2.99742
H	12.25979	-0.44192	-1.41694
H	11.35165	1.09122	-1.54423
C	10.21576	-2.06398	-1.87755
H	11.13190	-2.51265	-1.45746
H	10.22738	-2.23868	-2.98206
H	9.35329	-2.59437	-1.44372
H	6.87336	0.09243	-2.22439

3K·**2**κ^1L

SCF (BP86) Energy = -4460.58446124
Enthalpy 0K = -4458.463908
Enthalpy 298K = -4458.319977
Free Energy 298K = -4458.670132
Lowest Frequency = 5.5109 cm^{-1}
Second Frequency = 7.3487 cm^{-1}
SCF (BP86-D3BJ) Energy = -4461.29118801
SCF (C6H6) Energy = -4460.59428319
SCF (BS2) Energy = -7890.75514488

Si	-4.27101	-6.19865	-2.04337
Si	-1.40653	-0.44405	-5.56050
Si	-5.45030	4.19566	-0.00372
Si	0.77018	1.96325	5.44792
O	-2.00051	-3.11958	-0.08451
O	-0.24229	-0.39664	-1.98743
O	-3.16502	0.57549	0.29939
O	0.02730	-0.45076	1.96937
C	-2.46817	-4.42903	-0.17186
C	-3.56178	-4.70327	0.91549
C	-1.26205	-5.38670	0.03091
C	0.51819	-0.12102	-3.11016
C	1.19245	-1.39875	-3.70482
C	1.65585	0.87455	-2.69563
C	-4.36778	1.27520	0.27962
C	-5.07471	1.21564	1.66889
C	-5.22601	0.61275	-0.83732
C	0.49806	-0.28206	3.26974
C	-0.18863	-1.37281	4.14312
C	2.05187	-0.39567	3.33931
C	-0.34786	-1.36619	-6.84761
H	0.42146	-0.70558	-7.28208
H	0.16621	-2.23693	-6.41062
H	-0.99021	-1.72206	-7.67270
C	-2.39147	0.91570	-6.47962
H	-1.71573	1.64671	-6.95662
H	-3.02413	0.48007	-7.27259
H	-3.05101	1.47182	-5.79040
C	-2.69807	-1.68174	-4.88019
H	-2.24753	-2.45479	-4.23519
H	-3.52162	-1.17389	-4.34701
H	-3.16026	-2.20759	-5.73452
C	-0.33455	0.51849	-4.28471
H	0.33923	1.07007	-4.97444
H	-0.98474	1.29020	-3.82506
C	-4.10960	2.80524	-0.00917
H	-3.55354	2.87565	-0.96583
H	-3.38413	3.11931	0.76782
C	-7.09716	3.75385	-0.86059
H	-6.94649	3.42179	-1.90071
H	-7.63650	2.95295	-0.32995
H	-7.75196	4.64306	-0.87883
C	-4.67655	5.65966	-0.96501
H	-4.45531	5.38576	-2.01148
H	-5.35314	6.53177	-0.98214
H	-3.73176	5.98117	-0.49218
C	-5.80601	4.78656	1.77376
H	-6.24074	3.98162	2.38812
H	-4.87951	5.12531	2.26905
H	-6.51263	5.63493	1.76904
C	-0.39957	3.43858	5.79487

H	-0.38707	4.16905	4.96741
H	-1.44368	3.10629	5.93076
H	-0.09826	3.97286	6.71299
C	2.53841	2.64789	5.24460
H	2.86427	3.16020	6.16691
H	3.26119	1.84570	5.02413
H	2.58593	3.38166	4.42125
C	0.15856	1.17255	3.79064
H	-0.94059	1.30689	3.73080
H	0.56810	1.84651	3.01157
C	0.72190	0.82808	6.98151
H	1.04258	1.40254	7.86875
H	-0.28896	0.43639	7.17984
H	1.40061	-0.03321	6.87708
C	-6.03159	-5.99164	-1.33900
H	-6.03276	-6.01354	-0.23708
H	-6.47541	-5.03273	-1.65873
H	-6.69000	-6.80154	-1.69929
C	-4.38801	-6.16620	-3.95293
H	-4.78295	-5.19909	-4.31032
H	-3.39925	-6.31621	-4.42065
H	-5.05856	-6.95991	-4.32607
C	-3.62402	-7.91695	-1.52049
H	-4.35455	-8.68607	-1.82867
H	-2.65692	-8.16674	-1.98551
H	-3.50081	-7.99024	-0.42802
C	-3.17818	-4.67378	-1.56358
H	-2.42608	-4.51601	-2.36284
H	-3.89676	-3.83442	-1.66463
C	3.74059	2.60652	-1.79047
H	4.55204	3.26515	-1.46335
C	2.69183	3.10368	-2.57802
H	2.67705	4.16057	-2.86798
C	1.66419	2.24799	-3.02011
H	0.87327	2.66298	-3.65251
C	3.74088	1.24340	-1.44165
H	4.56124	0.82995	-0.84444
C	2.71114	0.40014	-1.88126
H	2.74774	-0.66480	-1.62671
C	2.29712	-1.33722	-4.58211
H	2.73130	-0.36250	-4.83230
C	2.85271	-2.50344	-5.13173
H	3.71161	-2.42995	-5.80861
C	2.30902	-3.76273	-4.82076
H	2.73914	-4.67313	-5.25211
C	1.20885	-3.83930	-3.95037
H	0.77739	-4.81308	-3.69263
C	0.66489	-2.66691	-3.39585
H	-0.19240	-2.71834	-2.71442
C	0.39955	-7.02783	-0.73880
H	0.78411	-7.66603	-1.54254
C	1.00911	-7.04494	0.52384
H	1.86645	-7.69753	0.71953
C	0.50085	-6.20936	1.53683
H	0.96268	-6.21300	2.53136
C	-0.72121	-6.20942	-0.97778
H	-1.18627	-6.23352	-1.96707
C	-0.60499	-5.38487	1.28511
H	-0.99268	-4.74447	2.08419
C	2.79815	0.18619	2.29391
H	2.25493	0.61509	1.44531
C	4.20027	0.21923	2.32917
H	4.74850	0.71545	1.52041
C	4.90432	-0.37596	3.39232
H	6.00090	-0.38100	3.39500
C	4.17531	-0.98279	4.42781
H	4.70193	-1.45981	5.26230
C	2.76763	-0.97805	4.40760
H	2.22030	-1.44427	5.23326
C	0.19816	-2.72721	3.99659
H	1.06815	-2.96344	3.37442
C	-0.48820	-3.76442	4.64525
H	-0.14499	-4.79883	4.53076
C	-1.61289	-3.48504	5.44356
H	-2.15319	-4.29380	5.94568
C	-2.02419	-2.15228	5.59190
H	-2.89155	-1.91123	6.21690
C	-1.31510	-1.11439	4.95637
H	-1.65134	-0.08424	5.10734
C	-4.54773	-3.71333	1.11996
H	-4.50717	-2.80908	0.50258
C	-5.57792	-3.87500	2.05907
H	-6.33330	-3.09026	2.18495
C	-5.64554	-5.04788	2.83244
H	-6.44239	-5.17957	3.57201
C	-4.68295	-6.04978	2.63630
H	-4.72545	-6.97285	3.22568
C	-3.65803	-5.88088	1.68637
H	-2.91911	-6.67682	1.55214
C	-4.25763	1.27107	2.81863
H	-3.17268	1.33038	2.67035
C	-4.80604	1.27016	4.11063
H	-4.14698	1.33425	4.98422
C	-6.19915	1.19437	4.28546
H	-6.63230	1.18394	5.29127
C	-7.02799	1.13872	3.15412
H	-8.11588	1.08159	3.27352
C	-6.47120	1.15891	1.86195
H	-7.13652	1.12053	0.99334
C	-6.45899	-1.37080	-1.62954
H	-6.94321	-2.32621	-1.39900
C	-5.85269	-0.63485	-0.59921
H	-5.87663	-1.02627	0.42288
C	-6.45304	-0.88084	-2.94842
H	-6.93030	-1.44678	-3.75501
C	-5.83868	0.35535	-3.20955
H	-5.83783	0.76247	-4.22706
C	-5.23543	1.08682	-2.16927
H	-4.76802	2.04689	-2.40769
K	-2.70960	-1.14691	-1.69095
K	0.60012	-2.20184	-0.03076
K	-2.48229	-1.44818	1.95377
K	-0.62320	1.39077	0.08838
N	7.23265	-2.17048	-0.43532
N	8.72168	-0.83736	2.16231
N	9.96950	-2.31482	-1.75398
N	3.53720	-3.37007	-0.02950
C	7.26766	-0.79549	0.10280
H	6.48300	-0.69154	0.87192
H	7.02561	-0.06120	-0.69924
C	7.42452	-2.24903	-1.88663
H	7.29084	-3.30615	-2.18144
C	6.09048	-2.97416	0.04016
H	6.16156	-3.02645	1.14154
H	6.23677	-4.00433	-0.33556
C	4.67047	-2.46982	-0.35423
H	4.47789	-1.50858	0.15632
H	4.62889	-2.27935	-1.44213

```
C   3.52396 -4.57596 -0.87211
H   3.50101 -4.28440 -1.93540
H   2.61542 -5.16275 -0.65703
H   4.39736 -5.24878 -0.70818
C   3.49257 -3.72405  1.39912
H   4.32634 -4.38634  1.72524
H   2.55285 -4.26674  1.60648
H   3.51950 -2.80814  2.01371
C   8.59477 -0.36721  0.76710
H   9.45369 -0.69678  0.13969
H   8.59900  0.73874  0.79888
C   9.66213 -0.00634  2.91721
H   9.70417 -0.35699  3.96336
H   9.32095  1.04333  2.92268
H  10.70362 -0.03073  2.51185
C   9.13892 -2.24424  2.22756
H   8.47569 -2.85770  1.60072
H   9.08676 -2.59766  3.27263
H  10.18114 -2.39929  1.85849
C   8.78472 -1.74397 -2.40171
H   8.78540 -1.92997 -3.50761
H   8.84155 -0.64508 -2.27842
C  11.18467 -1.77768 -2.36359
H  11.29889 -2.04785 -3.44282
H  12.07070 -2.15371 -1.82377
H  11.18230 -0.67583 -2.29126
C   9.97714 -3.77624 -1.75485
H  10.90685 -4.13289 -1.27981
H   9.92536 -4.22307 -2.77875
H   9.13035 -4.15428 -1.16042
H   6.65690 -1.66979 -2.45829
N   0.03505  7.52824  0.09472
N   3.26156  8.36901 -0.40203
N  -0.58367  9.07763 -2.64033
N   0.08581  4.33686  0.59088
C   0.99830  8.44340  0.71235
H   1.56492  7.88418  1.48437
H   0.41782  9.21012  1.25832
C  -1.22211  8.13174 -0.34652
H  -1.85189  7.32785 -0.76867
C   0.60987  6.49584 -0.78121
H   1.68224  6.41579 -0.53239
H   0.58674  6.79697 -1.85195
C  -0.05837  5.10327 -0.67084
H  -1.14288  5.17816 -0.87938
H   0.37802  4.48291 -1.47877
C   1.48690  4.22738  1.02248
H   2.10789  3.85463  0.19014
H   1.55961  3.50647  1.85609
H   1.92135  5.18892  1.37781
C  -0.75275  4.85288  1.68682
H  -0.44374  5.85831  2.03788
H  -0.71215  4.14577  2.53509
H  -1.80117  4.91843  1.34863
C   2.01830  9.14530 -0.23013
H   1.55067  9.24866 -1.22498
H   2.24008 10.17709  0.14420
C   4.15292  8.51064  0.75050
H   5.03818  7.86486  0.61979
H   3.64250  8.19514  1.67548
H   4.50770  9.56078  0.89843
C   3.95933  8.74818 -1.63201
H   3.29835  8.58440 -2.49963
H   4.85772  8.11898 -1.75844
H   4.28981  9.81576 -1.64386
C  -1.18784  9.33535 -1.32370
H  -2.23778  9.71643 -1.42256
H  -0.61195 10.15401 -0.85300
C  -0.35501 10.33745 -3.35455
H  -1.29578 10.90389 -3.55994
H   0.13469 10.13325 -4.32224
H   0.31187 10.98794 -2.76295
C  -1.40843  8.18407 -3.45864
H  -0.90888  8.00424 -4.42572
H  -2.42427  8.59981 -3.66630
H  -1.53566  7.20909 -2.96138
H  -1.77092  8.49269  0.54804
```

3K’’

SCF (BP86) Energy = -3072.02093353
Enthalpy 0K = -3070.731179
Enthalpy 298K = -3070.633471
Free Energy 298K = -3070.875368
Lowest Frequency = 9.5841 cm^{-1}
Second Frequency = 11.1263 cm^{-1}
SCF (BP86-D3BJ) Energy = -3072.52197208
SCF (C6H6) Energy = -3072.02948882
SCF (BS2) Energy = -6501.85514724

```
Si -5.56825 -2.20067 -1.93358
Si -3.45614  4.57540  0.72168
Si  1.10540  1.64355  5.09977
Si  6.43349 -0.17924 -0.68654
O  -1.55848 -1.21840 -0.75528
O  -0.62880  2.37851 -0.00775
O   0.26892 -0.35971  2.70775
O   2.14320 -0.32074 -0.66624
C  -2.46837 -1.89198 -1.57084
C  -2.42489 -3.43016 -1.31541
C  -2.05998 -1.55668 -3.03372
C  -0.61894  3.75147 -0.21600
C  -1.11116  4.14959 -1.63935
C   0.86767  4.21925 -0.04579
C  -0.09762 -0.91192  3.93319
C   0.40497 -2.38413  4.09359
C  -1.65502 -0.87167  4.00364
C   3.38099 -0.75361 -1.14189
C   3.36952 -2.30544 -1.04318
C   3.60897 -0.29419 -2.61593
C  -4.11805  5.64962 -0.70482
H  -3.66650  6.65596 -0.67921
H  -3.90395  5.21159 -1.69245
H  -5.21162  5.77075 -0.60909
C  -4.00234  5.39199  2.36315
H  -3.57990  6.40666  2.46503
H  -5.10139  5.48233  2.41480
H  -3.67166  4.80388  3.23708
C  -4.30304  2.85986  0.65472
H  -3.95725  2.25672 -0.20118
H  -4.14217  2.29946  1.59368
H  -5.39452  2.98913  0.54856
C  -1.53711  4.52971  0.80651
H  -1.26520  5.60669  0.80761
H  -1.28943  4.15741  1.81994
C   0.53446 -0.18051  5.19382
H   1.48549 -0.69573  5.43433
H  -0.09323 -0.33826  6.09169
```

C	1.33088	2.25143	6.89798
H	0.36700	2.28975	7.43444
H	2.00078	1.58038	7.46279
H	1.76955	3.26433	6.92413
C	-0.05903	2.89000	4.23983
H	-0.98899	3.03398	4.81586
H	0.43666	3.87431	4.16958
H	-0.34107	2.56846	3.22414
C	2.84015	1.69237	4.27171
H	2.97266	0.84350	3.57971
H	3.03323	2.63932	3.73612
H	3.62286	1.59190	5.04376
C	7.30999	0.33818	0.93384
H	6.96241	1.32920	1.27503
H	7.11431	-0.38279	1.74684
H	8.40373	0.39785	0.79648
C	6.87978	1.09167	-2.03631
H	7.97127	1.11585	-2.20104
H	6.39362	0.85219	-2.99602
H	6.56368	2.10734	-1.74170
C	4.54267	-0.10867	-0.29105
H	4.40439	-0.42465	0.76320
H	4.32271	0.97870	-0.30202
C	7.10703	-1.88799	-1.20451
H	8.19706	-1.81864	-1.36812
H	6.93017	-2.65858	-0.43660
H	6.64943	-2.23945	-2.14341
C	-6.00744	-3.79849	-0.98972
H	-5.24362	-4.57942	-1.13532
H	-6.09162	-3.60634	0.09408
H	-6.97741	-4.19859	-1.33344
C	-6.93677	-0.90962	-1.58583
H	-6.98817	-0.66339	-0.51073
H	-6.75472	0.03025	-2.13575
H	-7.92876	-1.28735	-1.88916
C	-5.56789	-2.56690	-3.80659
H	-6.56141	-2.94296	-4.10854
H	-5.35060	-1.66815	-4.40662
H	-4.82432	-3.33508	-4.07360
C	-3.93872	-1.42859	-1.23819
H	-3.98990	-0.33200	-1.39623
H	-4.03199	-1.56599	-0.14146
C	3.66464	4.69429	0.31655
H	4.73420	4.88493	0.45241
C	2.75313	4.97156	1.34828
H	3.10960	5.38945	2.29702
C	1.37625	4.73950	1.16554
H	0.68827	4.98005	1.98110
C	3.17745	4.19418	-0.90510
H	3.86885	3.99044	-1.73004
C	1.80374	3.96218	-1.07592
H	1.43753	3.59686	-2.04226
C	-0.83449	5.40900	-2.21411
H	-0.21456	6.12681	-1.66462
C	-1.32957	5.75091	-3.48231
H	-1.09555	6.73199	-3.91076
C	-2.12308	4.84122	-4.20313
H	-2.51031	5.10802	-5.19222
C	-2.41701	3.58938	-3.63876
H	-3.04457	2.87498	-4.18308
C	-1.90903	3.25107	-2.37194
H	-2.15072	2.28923	-1.90564
C	-2.15882	-0.13282	-5.03855
H	-2.67976	0.63953	-5.61544

C	-0.97618	-0.70025	-5.53716
H	-0.56432	-0.37514	-6.49798
C	-0.33579	-1.70684	-4.78906
H	0.58220	-2.17028	-5.16743
C	-2.69063	-0.55636	-3.80511
H	-3.61522	-0.09512	-3.44571
C	-0.87047	-2.12035	-3.55966
H	-0.36023	-2.90059	-2.98547
C	3.18113	1.00140	-2.97519
H	2.71297	1.61727	-2.19972
C	3.37012	1.50790	-4.27053
H	3.03661	2.52313	-4.51648
C	3.99196	0.71512	-5.25156
H	4.13644	1.10056	-6.26640
C	4.43093	-0.57394	-4.91133
H	4.92084	-1.20226	-5.66382
C	4.24672	-1.06907	-3.60707
H	4.59651	-2.07732	-3.36296
C	2.51254	-3.03374	-1.90606
H	1.99347	-2.49575	-2.70625
C	2.31984	-4.41590	-1.76336
H	1.65812	-4.94848	-2.45571
C	2.98019	-5.12024	-0.73830
H	2.83713	-6.19975	-0.62517
C	3.83475	-4.41906	0.12539
H	4.37132	-4.95405	0.91718
C	4.02297	-3.03137	-0.02499
H	4.69707	-2.51510	0.66444
C	-2.26328	-3.87003	0.01494
H	-2.16846	-3.10387	0.79205
C	-2.24402	-5.23391	0.34531
H	-2.12699	-5.54322	1.39042
C	-2.37858	-6.20216	-0.66541
H	-2.35682	-7.26866	-0.41758
C	-2.54629	-5.78477	-1.99516
H	-2.65540	-6.52799	-2.79313
C	-2.57564	-4.41467	-2.31431
H	-2.70730	-4.11112	-3.35792
C	1.60684	-2.75624	3.45701
H	2.11673	-1.99761	2.85064
C	2.15973	-4.03658	3.62585
H	3.09766	-4.29469	3.12224
C	1.51262	-4.98149	4.43978
H	1.93502	-5.98362	4.56921
C	0.32473	-4.62073	5.09667
H	-0.18381	-5.34334	5.74510
C	-0.21625	-3.33439	4.93223
H	-1.13679	-3.06634	5.46252
C	-3.81798	-1.72635	3.18219
H	-4.37586	-2.50179	2.64563
C	-2.42347	-1.83563	3.30694
H	-1.91453	-2.70295	2.87359
C	-4.49578	-0.63509	3.75677
H	-5.58364	-0.54705	3.66975
C	-3.75635	0.32433	4.46986
H	-4.26969	1.16666	4.94756
C	-2.35999	0.20118	4.59258
H	-1.81158	0.95558	5.16267
K	-1.95980	0.50223	1.20386
K	0.04161	0.56641	-2.01665
K	0.56802	-2.16761	0.51606
K	1.60450	1.39957	1.23727

3K''-TS(c-d)

SCF (BP86) Energy = -3071.97049975

Enthalpy 0K = -3070.681936

Enthalpy 298K = -3070.584995

Free Energy 298K = -3070.823791

Lowest Frequency = -390.2283 cm^{-1}

Second Frequency = 9.2381 cm^{-1}

SCF (BP86-D3BJ) Energy = -3072.47273720

SCF (C6H6) Energy = -3071.98051166

SCF (BS2) Energy = -6501.80385326

Si	4.73658	1.62364	3.37990
Si	1.10085	5.42370	-1.86605
Si	0.43926	-2.76118	-3.59269
Si	-5.94001	-3.10357	-0.16848
O	1.30846	0.06163	1.30499
O	-0.64505	2.24846	-1.09655
O	1.41149	-2.05594	-2.34493
O	-2.15002	-1.09767	0.13606
C	2.08276	0.24237	2.45452
C	2.72951	-1.09879	2.91303
C	1.11061	0.81443	3.52507
C	-1.23217	3.38611	-1.64220
C	-1.77073	4.37007	-0.56147
C	-2.43298	2.88393	-2.51389
C	2.98452	-2.00119	-3.24074
C	3.82660	-3.08930	-2.57649
C	3.44414	-0.58680	-2.95436
C	-3.20319	-1.84560	0.68039
C	-2.53946	-3.08301	1.34897
C	-4.01403	-1.00066	1.71148
C	0.36231	7.03005	-1.15956
H	-0.32516	7.49952	-1.88352
H	-0.19697	6.86033	-0.22616
H	1.17210	7.75170	-0.95157
C	2.15559	5.90364	-3.38795
H	1.53717	6.38118	-4.16755
H	2.94893	6.61872	-3.10876
H	2.64354	5.02383	-3.84244
C	2.29137	4.66324	-0.57546
H	1.75505	4.22319	0.28149
H	2.94327	3.89805	-1.03365
H	2.95836	5.44945	-0.18002
C	-0.23437	4.21539	-2.54630
H	-0.81331	4.86451	-3.23694
H	0.29853	3.49126	-3.19525
C	2.67313	-2.29398	-4.61084
H	3.01265	-3.24873	-5.01898
H	2.61693	-1.47999	-5.33929
C	0.70166	-4.50753	-4.35899
H	0.97232	-4.44050	-5.42436
H	1.52753	-5.02724	-3.84326
H	-0.20714	-5.12936	-4.25734
C	-0.35441	-1.54413	-4.87284
H	0.01659	-1.76544	-5.88693
H	-1.46018	-1.62183	-4.89792
H	-0.07962	-0.49177	-4.66732
C	-1.09357	-3.26306	-2.47248
H	-1.48787	-2.57409	-1.70242
H	-1.94043	-3.53519	-3.13349
H	-0.82824	-4.19276	-1.93276
C	-6.44793	-3.79143	-1.87967
H	-6.47266	-2.99104	-2.64002
H	-5.74112	-4.56349	-2.23035
H	-7.45220	-4.24838	-1.84475
C	-7.25291	-1.82825	0.36879
H	-8.24831	-2.30143	0.43503
H	-7.01703	-1.38937	1.35185
H	-7.32433	-1.00289	-0.36076
C	-4.22892	-2.24981	-0.44553
H	-3.68262	-2.83534	-1.20966
H	-4.49202	-1.29427	-0.94449
C	-5.93718	-4.55002	1.07576
H	-6.94390	-5.00199	1.11888
H	-5.22371	-5.34064	0.79191
H	-5.67722	-4.21457	2.09283
C	6.04998	0.24382	3.33089
H	5.64622	-0.71476	3.69475
H	6.41933	0.08497	2.30295
H	6.91692	0.51211	3.95975
C	5.53299	3.22963	2.71121
H	5.86373	3.10572	1.66505
H	4.82409	4.07546	2.74065
H	6.41746	3.51388	3.30753
C	4.21929	1.93303	5.18980
H	5.10630	2.22123	5.78125
H	3.47624	2.74219	5.27878
H	3.78861	1.03011	5.65170
C	3.28501	1.22283	2.16465
H	2.86755	2.19148	1.81978
H	3.79906	0.79021	1.28194
C	-4.50569	1.54761	-3.96187
H	-5.30169	1.04260	-4.51870
C	-3.32614	1.95086	-4.61089
H	-3.19801	1.76201	-5.68268
C	-2.30859	2.61114	-3.89555
H	-1.40457	2.91982	-4.42966
C	-4.65948	1.82918	-2.59092
H	-5.58186	1.54579	-2.07199
C	-3.63808	2.48513	-1.88493
H	-3.77783	2.70869	-0.82188
C	-2.75285	5.34413	-0.84409
H	-3.18623	5.39607	-1.84939
C	-3.19376	6.23797	0.14471
H	-3.96339	6.97920	-0.09790
C	-2.65321	6.18572	1.44121
H	-2.99773	6.88214	2.21292
C	-1.66203	5.23463	1.73330
H	-1.21971	5.18978	2.73481
C	-1.23343	4.33831	0.73886
H	-0.43871	3.61396	0.94537
C	-0.03959	2.70103	4.61113
H	-0.08616	3.77634	4.81811
C	-0.99794	1.83794	5.16492
H	-1.80116	2.22896	5.79763
C	-0.89872	0.45717	4.90803
H	-1.62926	-0.23445	5.34215
C	0.99756	2.19462	3.80450
H	1.73020	2.89574	3.39416
C	0.13542	-0.03861	4.09919
H	0.19803	-1.11374	3.90311
C	-4.29103	0.34579	1.38721
H	-3.91401	0.73187	0.43279
C	-5.04567	1.17059	2.23636
H	-5.25198	2.20855	1.95049
C	-5.54415	0.66192	3.44887
H	-6.12955	1.30034	4.11882
C	-5.29033	-0.67735	3.78384

H	-5.67993	-1.09101	4.72088
C	-4.53992	-1.49883	2.92232
H	-4.35881	-2.54164	3.20139
C	-1.79769	-2.90847	2.54378
H	-1.84246	-1.94210	3.05666
C	-1.00951	-3.93646	3.08442
H	-0.45687	-3.76752	4.01555
C	-0.93044	-5.18313	2.43495
H	-0.31890	-5.98940	2.85295
C	-1.66194	-5.37996	1.25300
H	-1.62308	-6.34690	0.73880
C	-2.45617	-4.34560	0.72285
H	-3.00537	-4.53066	-0.20369
C	3.25367	-1.95229	1.91741
H	3.17294	-1.64298	0.86853
C	3.89631	-3.15668	2.24360
H	4.30509	-3.78385	1.44362
C	4.01809	-3.54638	3.58920
H	4.51340	-4.48760	3.85064
C	3.50652	-2.71040	4.59373
H	3.59854	-2.99805	5.64724
C	2.87690	-1.49751	4.25826
H	2.49153	-0.85684	5.05788
C	3.34801	-3.98209	-1.59997
H	2.31136	-3.87245	-1.27609
C	4.16207	-5.01341	-1.09451
H	3.75233	-5.71382	-0.35757
C	5.48481	-5.15422	-1.53808
H	6.12029	-5.95441	-1.14394
C	5.98147	-4.26398	-2.50668
H	7.00943	-4.36652	-2.87142
C	5.15863	-3.25548	-3.02647
H	5.53900	-2.58581	-3.80522
C	4.95425	0.99177	-1.80949
H	5.73395	1.16392	-1.05921
C	4.42047	-0.30140	-1.96599
H	4.80013	-1.11503	-1.34133
C	4.51174	2.05085	-2.62017
H	4.94840	3.05002	-2.52347
C	3.50539	1.79685	-3.57664
H	3.14423	2.60732	-4.21983
C	2.97578	0.50801	-3.73002
H	2.20966	0.33388	-4.49113
K	1.77926	1.30552	-0.91205
K	-1.20788	1.05706	1.35993
K	0.38731	-2.23095	0.28572
K	-1.75807	-0.05993	-2.21680

3K''-d

SCF (BP86) Energy = -3072.06152013

Enthalpy 0K = -3070.773871

Enthalpy 298K = -3070.674375

Free Energy 298K = -3070.929231

Lowest Frequency = 5.8863 cm^{-1}

Second Frequency = 7.3648 cm^{-1}

SCF (BP86-D3BJ) Energy = -3072.53068517

SCF (C6H6) Energy = -3072.07244708

SCF (BS2) Energy = -6501.90441458

Si	3.99199	0.00111	3.84087
Si	0.80948	5.49536	-0.25884
Si	0.75135	-0.80704	-3.99274
Si	-6.39927	-2.71910	-2.10010
O	0.69210	-0.52002	1.14180
O	-1.13071	2.43064	-0.35212
O	0.20593	-0.43864	-2.48566
O	-2.80717	-1.11596	-0.37905
C	1.39262	-0.83947	2.30207
C	1.98945	-2.27716	2.21448
C	0.35718	-0.71200	3.45537
C	-1.67678	3.69033	-0.54095
C	-2.15509	4.35136	0.78725
C	-2.91931	3.50317	-1.47828
C	7.31789	-0.36487	-2.78701
C	7.55692	-1.54391	-1.90289
C	6.73261	0.87452	-2.19425
C	-3.87771	-2.00675	-0.37439
C	-3.23717	-3.41886	-0.25079
C	-4.85092	-1.70810	0.80553
C	0.25770	6.85543	0.95404
H	-0.38575	7.59540	0.44852
H	-0.30679	6.44798	1.80744
H	1.14033	7.39116	1.34607
C	1.88967	6.32306	-1.60433
H	1.31821	7.08518	-2.16196
H	2.76534	6.82608	-1.15813
H	2.26248	5.58604	-2.33755
C	1.92278	4.24549	0.66996
H	1.32965	3.55795	1.29454
H	2.54544	3.65883	-0.02994
H	2.62180	4.78445	1.33313
C	-0.65763	4.69636	-1.20802
H	-1.20234	5.57227	-1.61969
H	-0.21694	4.17950	-2.08484
C	7.63715	-0.41481	-4.10581
H	8.01361	-1.33308	-4.56562
H	7.53007	0.45970	-4.75415
C	1.34842	-2.62685	-4.13976
H	2.17964	-2.82960	-3.43990
H	0.53027	-3.33568	-3.91289
H	1.70991	-2.85906	-5.15745
C	2.22022	0.30021	-4.54526
H	3.07637	0.19859	-3.85367
H	2.57746	0.03288	-5.55596
H	1.92542	1.36575	-4.56964
C	-0.61533	-0.57984	-5.33104
H	-1.49721	-1.21339	-5.11980
H	-0.95324	0.47256	-5.38651
H	-0.25018	-0.85443	-6.33692
C	-6.62446	-2.53904	-3.99193
H	-6.59349	-1.47863	-4.29835
H	-5.83061	-3.07060	-4.54543
H	-7.59399	-2.95210	-4.32066
C	-7.84699	-1.82455	-1.24024
H	-8.81146	-2.27661	-1.53078
H	-7.76049	-1.87664	-0.14282
H	-7.87812	-0.75883	-1.52606
C	-4.72384	-1.84575	-1.69699
H	-4.04361	-2.02219	-2.55540
H	-4.97333	-0.76566	-1.73899
C	-6.47197	-4.57748	-1.67308
H	-7.43300	-4.99823	-2.01813
H	-5.66100	-5.14682	-2.15597
H	-6.40177	-4.75184	-0.58715
C	5.27455	-1.33129	3.37737
H	4.82228	-2.33618	3.35352
H	5.71304	-1.13244	2.38435

H	6.10058	-1.34498	4.10991
C	4.87452	1.69824	3.84336
H	5.26516	1.93901	2.83918
H	4.18896	2.51085	4.14070
H	5.72665	1.70525	4.54516
C	3.35599	-0.33970	5.60769
H	4.20769	-0.35324	6.31060
H	2.64766	0.43203	5.95076
H	2.84706	-1.31462	5.67984
C	2.61987	0.13484	2.48687
H	2.22679	1.17142	2.50717
H	3.18356	0.05921	1.53432
C	-5.09402	2.73692	-3.16875
H	-5.93025	2.45198	-3.81555
C	-3.93362	3.31069	-3.71412
H	-3.86134	3.48031	-4.79470
C	-2.86527	3.69064	-2.87814
H	-1.97929	4.14685	-3.33083
C	-5.17669	2.56093	-1.77397
H	-6.08206	2.13609	-1.32663
C	-4.10533	2.93783	-0.94969
H	-4.18898	2.80757	0.13511
C	-3.05578	5.43788	0.81223
H	-3.46742	5.81782	-0.13009
C	-3.44417	6.02852	2.02497
H	-4.15058	6.86625	2.01996
C	-2.93267	5.54901	3.24370
H	-3.23626	6.00930	4.18996
C	-2.02655	4.47615	3.23386
H	-1.61034	4.09976	4.17537
C	-1.64998	3.88531	2.01484
H	-0.92521	3.06472	1.98584
C	-0.86224	0.63821	5.11407
H	-0.92265	1.55051	5.71843
C	-1.86177	-0.34172	5.22128
H	-2.70837	-0.20235	5.90130
C	-1.74916	-1.51534	4.45153
H	-2.51311	-2.29691	4.53111
C	0.22995	0.45238	4.24524
H	0.99240	1.23457	4.18699
C	-0.66126	-1.68967	3.58235
H	-0.59579	-2.60250	2.98086
C	-5.09092	-0.35592	1.13071
H	-4.57677	0.40957	0.53809
C	-5.97556	0.00833	2.15749
H	-6.15127	1.06754	2.37938
C	-6.63918	-0.98523	2.89912
H	-7.32426	-0.70858	3.70752
C	-6.41779	-2.33550	2.58637
H	-6.93055	-3.12070	3.15345
C	-5.53923	-2.69108	1.54628
H	-5.38129	-3.75006	1.31696
C	-2.67788	-3.81867	0.98883
H	-2.86513	-3.20257	1.87446
C	-1.89109	-4.97485	1.10600
H	-1.48496	-5.25988	2.08310
C	-1.62395	-5.76983	-0.02527
H	-1.01162	-6.67318	0.06239
C	-2.17050	-5.39444	-1.26291
H	-1.98790	-6.00853	-2.15217
C	-2.96753	-4.23846	-1.36968
H	-3.37927	-3.97554	-2.34849
C	2.51317	-2.69665	0.97223
H	2.46345	-1.99455	0.13131
C	3.09638	-3.96340	0.80867
H	3.50508	-4.25600	-0.16533
C	3.16009	-4.85371	1.89615
H	3.60446	-5.84710	1.77344
C	2.64986	-4.45202	3.14073
H	2.69513	-5.13385	3.99749
C	2.07848	-3.17586	3.29801
H	1.68823	-2.88051	4.27747
C	6.62643	-1.90611	-0.90078
H	5.72994	-1.29465	-0.75558
C	6.82864	-3.04364	-0.10566
H	6.08704	-3.30824	0.65529
C	7.97330	-3.83756	-0.28816
H	8.13599	-4.72129	0.33770
C	8.91352	-3.48356	-1.26968
H	9.81717	-4.08692	-1.40804
C	8.70903	-2.34762	-2.06566
H	9.45755	-2.05793	-2.81066
C	6.59698	2.49971	-0.36413
H	6.90638	2.81918	0.63676
C	7.09401	1.29997	-0.89387
H	7.78675	0.68983	-0.30573
C	5.71969	3.29823	-1.11810
H	5.34023	4.24010	-0.70863
C	5.33807	2.88005	-2.40493
H	4.65477	3.49358	-3.00249
C	5.83409	1.67903	-2.93404
H	5.51432	1.34680	-3.92703
K	1.17379	1.23148	-0.73519
K	-1.80935	0.48878	1.45435
K	-0.34030	-2.23005	-0.62255
K	-2.17396	0.60771	-2.23838

Hypothetical Heterometallic Clusters

$Li_3K(OR)_4$
SCF (BP86) Energy = -3009.65753335
Enthalpy 0K = -3008.359454
Enthalpy 298K = -3008.265660
Free Energy 298K = -3008.494165
Lowest Frequency = 10.3602 cm^{-1}
Second Frequency = 10.8840 cm^{-1}
SCF (BP86-D3BJ) Energy = -3010.17172101
SCF (C6H6) Energy = -3009.66704207
SCF (BS2) Energy = -4724.52767284

Si -5.64311 -1.74834 -0.78785
Si -3.01585 4.16038 1.56243
Si 2.32485 0.58091 4.49157
Si 5.95092 -0.52538 -1.30466
O -1.46755 -0.77868 -0.65315
O -0.29527 2.07895 0.22449
O 0.13784 -0.39020 1.79469
O 1.71330 -0.36539 -0.66250
C -2.58248 -1.39959 -1.26279
C -2.45804 -2.94713 -1.20788
C -2.60008 -0.87235 -2.72645
C -0.21163 3.46616 0.43392
C -0.67032 4.27147 -0.82002
C 1.28794 3.81851 0.70776
C -0.07289 -0.78529 3.12991
C 0.65702 -2.10984 3.46711
C -1.61729 -0.95074 3.33653
C 2.85638 -0.91084 -1.29670
C 2.73818 -2.45844 -1.26709
C 2.89587 -0.38595 -2.76993
C -3.49822 5.76347 0.65076
H -2.96638 6.63444 1.07074
H -3.26683 5.71450 -0.42527
H -4.58124 5.94948 0.76057
C -3.54681 4.35095 3.39046
H -3.03205 5.20096 3.87119
H -4.63277 4.53294 3.46940
H -3.31501 3.44717 3.98069
C -3.98941 2.70090 0.81969
H -3.74132 2.54664 -0.24253
H -3.79840 1.76171 1.36591
H -5.07354 2.90164 0.88568
C -1.10472 3.93589 1.63736
H -0.77535 4.94379 1.96476
H -0.88699 3.25799 2.48665
C 0.45452 0.30642 4.13521
H -0.00606 0.14071 5.13024
H 0.07285 1.28708 3.78932
C 3.12357 -0.79241 5.54262
H 2.54571 -0.98323 6.46272
H 3.20255 -1.74347 4.99283
H 4.14030 -0.48255 5.84371
C 2.40372 2.19954 5.50786
H 1.83517 2.10937 6.44983
H 3.44620 2.44972 5.77128
H 1.98630 3.05095 4.94368
C 3.32863 0.80334 2.89057
H 3.20718 -0.07398 2.23483
H 3.02182 1.71418 2.34575
H 4.40527 0.91182 3.10964
C 7.09976 -1.14779 0.09237
H 7.01924 -0.50524 0.98613
H 6.85623 -2.17881 0.40308
H 8.15466 -1.14055 -0.23283
C 6.50931 1.23809 -1.78036
H 7.55856 1.24419 -2.12304
H 5.88592 1.64187 -2.59619
H 6.43190 1.92430 -0.91921
C 4.15615 -0.42521 -0.57087
H 4.17438 -0.89796 0.42827
H 3.99983 0.65210 -0.37273
C 6.18523 -1.64665 -2.83031
H 7.25896 -1.69320 -3.08491
H 5.83401 -2.67622 -2.65386
H 5.64500 -1.25241 -3.70587
C -5.82324 -3.47222 0.00431
H -5.16771 -4.21532 -0.47750
H -5.55918 -3.43351 1.07477
H -6.86515 -3.82907 -0.07499
C -6.84145 -0.55576 0.10436
H -6.57332 -0.45785 1.17031
H -6.81538 0.45407 -0.34062
H -7.88211 -0.92007 0.04978
C -6.16696 -1.84435 -2.62171
H -7.20640 -2.21194 -2.68723
H -6.12627 -0.86087 -3.11822
H -5.53184 -2.53670 -3.19758
C -3.88028 -1.02452 -0.47173
H -3.97494 0.07854 -0.46787
H -3.66544 -1.29596 0.58090
C 4.06499 4.35348 1.08584
H 5.12896 4.56774 1.22967
C 3.15848 4.50942 2.14334
H 3.51177 4.84230 3.12522
C 1.78886 4.24296 1.95554
H 1.10630 4.38237 2.79845
C 3.58730 3.92759 -0.16643
H 4.27936 3.81308 -1.00809
C 2.22083 3.66716 -0.34486
H 1.85361 3.37698 -1.33493
C -0.25658 5.60192 -1.04817
H 0.43670 6.07742 -0.34591
C -0.70911 6.32146 -2.16531
H -0.36757 7.35073 -2.32153
C -1.59299 5.72786 -3.08276
H -1.94389 6.28810 -3.95579
C -2.02702 4.41113 -2.86134
H -2.72959 3.93808 -3.55662
C -1.56710 3.69597 -1.74127
H -1.93706 2.68448 -1.54253
C -3.24086 0.81942 -4.39533
H -3.87505 1.66929 -4.67188
C -2.31321 0.30651 -5.31604
H -2.21106 0.75235 -6.31090
C -1.53142 -0.80334 -4.94703
H -0.81016 -1.22753 -5.65398
C -3.37800 0.23875 -3.12090
H -4.10982 0.66228 -2.42745
C -1.67564 -1.37843 -3.67372
H -1.06438 -2.24715 -3.40836
C 2.97678 1.01320 -2.97627
H 3.05619 1.66902 -2.10291
C 2.96314 1.57251 -4.26410
H 3.03958 2.65912 -4.38758

C	2.86938	0.73931	-5.39433
H	2.86387	1.16927	-6.40139
C	2.80336	-0.64969	-5.21137
H	2.74919	-1.31473	-6.08065
C	2.81915	-1.20416	-3.91684
H	2.78628	-2.29124	-3.80328
C	1.52628	-3.05666	-1.68592
H	0.71592	-2.41511	-2.05151
C	1.33632	-4.44663	-1.65798
H	0.38055	-4.86926	-1.98405
C	2.36521	-5.28589	-1.19821
H	2.22398	-6.37132	-1.17077
C	3.57150	-4.71486	-0.76855
H	4.38270	-5.35422	-0.40247
C	3.75301	-3.31963	-0.80298
H	4.70213	-2.90674	-0.45156
C	-1.93347	-3.53225	-0.03660
H	-1.60790	-2.87557	0.77730
C	-1.86126	-4.92502	0.11730
H	-1.45255	-5.34813	1.04133
C	-2.30774	-5.77302	-0.91029
H	-2.24872	-6.86077	-0.79708
C	-2.83379	-5.20873	-2.08343
H	-3.18813	-5.85550	-2.89388
C	-2.91405	-3.81155	-2.22587
H	-3.33665	-3.39321	-3.14480
C	1.60357	-2.65275	2.57861
H	1.81672	-2.12573	1.64204
C	2.30082	-3.83431	2.89149
H	3.02678	-4.23342	2.17540
C	2.06122	-4.49609	4.10501
H	2.59807	-5.41914	4.34881
C	1.12579	-3.96005	5.00745
H	0.93167	-4.46344	5.96117
C	0.43758	-2.77939	4.69264
H	-0.29149	-2.37665	5.40467
C	-3.65689	-2.31920	3.45378
H	-4.12019	-3.31189	3.44192
C	-2.26329	-2.20592	3.30984
H	-1.66517	-3.11538	3.20414
C	-4.44951	-1.17440	3.62608
H	-5.53281	-1.26208	3.75891
C	-3.83007	0.08620	3.63624
H	-4.42952	0.99226	3.77689
C	-2.43710	0.19241	3.48797
H	-1.97689	1.18604	3.52618
K	-0.04110	0.91349	-2.25252
Li	1.21823	0.84736	0.76871
Li	0.21025	-1.37362	0.08291
Li	-1.29933	0.52985	0.77609

$Li_2K_2(OR)_4$
SCF (BP86) Energy = -3030.44560781
Enthalpy 0K = -3029.150102
Enthalpy 298K = -3029.055203
Free Energy 298K = -3029.287529
Lowest Frequency = 8.3640 cm^{-1}
Second Frequency = 10.2508 cm^{-1}
SCF (BP86-D3BJ) Energy = -3030.95427479
SCF (C6H6) Energy = -3030.45530854
SCF (BS2) Energy = -5316.96973032

Si	6.13704	0.70798	-0.49922
Si	1.01424	-5.35902	1.60200
Si	-3.14739	0.11955	4.30337
Si	-5.36377	1.85112	-1.88012
O	1.83843	0.71796	-0.54222
O	-0.30455	-2.27080	-0.04451
O	-0.30846	0.31517	1.93011
O	-1.37153	0.71727	-0.77655
C	3.07484	1.11005	-1.07232
C	3.33071	2.63290	-0.89053
C	3.03439	0.70635	-2.57539
C	-0.90789	-3.52932	-0.05408
C	-0.42279	-4.40922	-1.24306
C	-2.45118	-3.30360	-0.15980
C	-0.23075	0.49294	3.31999
C	-0.43382	1.97189	3.74459
C	1.20805	0.05356	3.76843
C	-2.29259	1.56610	-1.43370
C	-1.91840	3.03590	-1.10306
C	-2.21006	1.30489	-2.97526
C	1.11086	-6.96209	0.57977
H	0.17558	-7.54158	0.65861
H	1.29362	-6.76502	-0.48838
H	1.93119	-7.59654	0.95978
C	0.89904	-5.84507	3.44781
H	-0.01232	-6.43410	3.64921
H	1.76557	-6.45720	3.75277
H	0.87003	-4.95444	4.09973
C	2.67688	-4.42661	1.38793
H	2.78312	-3.97582	0.38641
H	2.82186	-3.65869	2.16904
H	3.50466	-5.14826	1.50494
C	-0.58904	-4.34632	1.26652
H	-1.37481	-5.11639	1.41779
H	-0.69985	-3.64306	2.11565
C	-1.29972	-0.37458	4.08119
H	-0.96839	-0.52422	5.12983
H	-1.29589	-1.38214	3.61817
C	-3.38179	1.46351	5.63381
H	-2.86736	1.19752	6.57307
H	-2.99434	2.44015	5.30332
H	-4.45641	1.57864	5.86177
C	-3.99007	-1.47603	4.94388
H	-3.52943	-1.82577	5.88438
H	-5.06192	-1.30309	5.14395
H	-3.91531	-2.29866	4.21113
C	-4.01744	0.66924	2.70781
H	-3.54948	1.58389	2.31069
H	-3.97176	-0.11046	1.92934
H	-5.08218	0.88637	2.90430
C	-6.61803	2.31095	-0.51008
H	-6.77561	1.46593	0.18204
H	-6.27404	3.17053	0.09138
H	-7.59713	2.57960	-0.94365
C	-6.10677	0.41829	-2.90030
H	-7.06471	0.71672	-3.36066
H	-5.42120	0.11331	-3.70886
H	-6.29456	-0.46335	-2.26441
C	-3.75543	1.23205	-0.98578
H	-3.84335	1.49144	0.08407
H	-3.83162	0.12837	-1.01908
C	-5.18403	3.34631	-3.05353
H	-6.18344	3.62842	-3.43014
H	-4.74645	4.22783	-2.55851

```
H  -4.55248  3.09898 -3.92186
C   6.66790  2.25165  0.48210
H   6.18806  3.16480  0.09503
H   6.39746  2.15315  1.54768
H   7.76218  2.38762  0.42685
C   6.97914 -0.82029  0.28713
H   6.65182 -0.96181  1.33217
H   6.73978 -1.74447 -0.26812
H   8.07764 -0.71207  0.29439
C   6.75089  0.85430 -2.29914
H   7.85234  0.93380 -2.30650
H   6.47128 -0.02212 -2.90630
H   6.34781  1.75006 -2.79802
C   4.24070  0.38208 -0.29744
H   4.10419 -0.71426 -0.41065
H   4.04877  0.59985  0.77256
C  -5.23458 -2.70374 -0.35718
H  -6.30633 -2.49294 -0.43250
C  -4.61548 -2.80464  0.89826
H  -5.20184 -2.66889  1.81350
C  -3.24422 -3.09933  0.99207
H  -2.79332 -3.19513  1.98509
C  -4.46098 -2.88635 -1.51521
H  -4.92778 -2.81074 -2.50333
C  -3.09029 -3.17399 -1.41417
H  -2.50992 -3.33652 -2.32866
C  -1.12049 -5.56159 -1.66802
H  -2.06160 -5.83228 -1.17695
C  -0.63736 -6.35614 -2.71927
H  -1.20378 -7.23887 -3.03612
C   0.56820 -6.02718 -3.36360
H   0.94601 -6.64866 -4.18220
C   1.28449 -4.89612 -2.94085
H   2.23383 -4.63520 -3.42245
C   0.78499 -4.09855 -1.89584
H   1.34421 -3.22460 -1.54409
C   3.33117 -0.95461 -4.36977
H   3.77335 -1.89797 -4.70981
C   2.54829 -0.18828 -5.24726
H   2.36686 -0.52768 -6.27232
C   2.01503  1.03308 -4.79433
H   1.40276  1.64698 -5.46294
C   3.56890 -0.51061 -3.05490
H   4.18933 -1.13005 -2.40043
C   2.25435  1.46677 -3.48109
H   1.82920  2.41806 -3.14598
C  -2.45572 -0.01048 -3.44109
H  -2.72328 -0.78739 -2.71487
C  -2.38411 -0.33485 -4.80557
H  -2.59395 -1.35989 -5.13359
C  -2.06554  0.65594 -5.75251
H  -2.01624  0.40947 -6.81841
C  -1.83166  1.96666 -5.31170
H  -1.59980  2.75552 -6.03642
C  -1.90477  2.28594 -3.94213
H  -1.73580  3.31931 -3.62686
C  -0.58573  3.46273 -1.31621
H   0.14135  2.74926 -1.72127
C  -0.17246  4.77161 -1.02454
H   0.86728  5.06330 -1.20358
C  -1.08849  5.69672 -0.49492
H  -0.77323  6.72060 -0.26795
C  -2.40770  5.28814 -0.25196
H  -3.13331  5.99255  0.17044
C  -2.81368  3.97397 -0.55078
H  -3.84676  3.68510 -0.34140
C   2.90722  3.24188  0.30804
H   2.35878  2.63278  1.03404
C   3.17630  4.59201  0.57891
H   2.83669  5.03518  1.52137
C   3.86880  5.37488 -0.36099
H   4.07467  6.43130 -0.15837
C   4.29031  4.78782 -1.56477
H   4.82549  5.38639 -2.31040
C   4.03031  3.42955 -1.82139
H   4.37130  2.98870 -2.76361
C  -1.01667  2.89398  2.85691
H  -1.30952  2.55199  1.85846
C  -1.24032  4.22788  3.24395
H  -1.69289  4.92348  2.52978
C  -0.87723  4.66204  4.52821
H  -1.04471  5.70189  4.82924
C  -0.29335  3.75009  5.42495
H  -0.00401  4.07626  6.43029
C  -0.07713  2.41989  5.03564
H   0.38369  1.71955  5.74186
C   3.62801  0.50743  3.76785
H   4.45168  1.20454  3.57830
C   2.30650  0.91077  3.52021
H   2.11178  1.91961  3.14289
C   3.89469 -0.77832  4.27443
H   4.92316 -1.09223  4.48115
C   2.81917 -1.64226  4.53819
H   3.00467 -2.63745  4.95808
C   1.49557 -1.22818  4.29012
H   0.67619 -1.91676  4.51836
K   1.77650 -1.29983  1.17091
K   0.25509 -0.74152 -2.26064
Li -1.31445 -0.66670  0.57548
Li  0.19516  1.30200  0.26835
```

$LiK_3(OR)_4$

SCF (BP86) Energy = -3051.23667425
Enthalpy 0K = -3049.944264
Enthalpy 298K = -3049.847951
Free Energy 298K = -3050.084589
Lowest Frequency = 10.8931 cm^{-1}
Second Frequency = 13.1183 cm^{-1}
SCF (BP86-D3BJ) Energy = -3051.74347435
SCF (C6H6) Energy = -3051.24510540
SCF (BS2) Energy = -5909.41628095

```
Si  3.69895  4.40507 -1.79330
Si  5.08914 -1.90317  2.46314
Si -3.40828  0.38474  4.34874
Si -4.90800 -3.43941 -1.35802
O   0.78651  1.35381 -0.97517
O   1.80701 -1.77588  0.88364
O  -0.81759  0.95225  1.83932
O  -1.53826 -0.85318 -0.72551
C   1.40670  2.25728 -1.84654
C   0.45353  3.41445 -2.25867
C   1.85913  1.41889 -3.07769
C   2.40310 -2.94423  1.33312
C   3.26965 -3.64203  0.24205
C   1.23164 -3.90473  1.73397
C  -1.04510  1.79126  2.92825
```

C	-2.06673	2.92566	2.63610
C	0.33222	2.44500	3.30223
C	-2.67962	-1.23895	-1.44526
C	-3.54407	0.01950	-1.72119
C	-2.22681	-1.91605	-2.78307
C	6.38628	-3.05749	1.68205
H	6.39334	-4.03795	2.18788
H	6.19807	-3.23439	0.61137
H	7.39395	-2.61841	1.78913
C	5.60978	-1.58462	4.27593
H	5.63908	-2.52355	4.85540
H	6.61520	-1.13151	4.32559
H	4.90807	-0.90033	4.78452
C	5.13858	-0.21037	1.56929
H	4.69450	-0.26012	0.56125
H	4.62735	0.56982	2.16206
H	6.18652	0.11861	1.45602
C	3.34473	-2.70400	2.57782
H	3.54754	-3.66853	3.08999
H	2.77350	-2.09248	3.30561
C	-1.59341	0.99903	4.18091
H	-1.44696	1.61047	5.09579
H	-0.94692	0.10777	4.32344
C	-4.65553	1.78991	4.64542
H	-4.33686	2.43567	5.48087
H	-4.77832	2.42786	3.75615
H	-5.64051	1.36352	4.90606
C	-3.40874	-0.73389	5.90209
H	-3.10330	-0.17293	6.80238
H	-4.41587	-1.14427	6.09199
H	-2.71644	-1.58761	5.79088
C	-3.99253	-0.67583	2.87314
H	-3.75931	-0.19056	1.91166
H	-3.53392	-1.68172	2.88903
H	-5.08438	-0.83002	2.92307
C	-6.30975	-3.49336	-0.05633
H	-5.92967	-3.83532	0.92224
H	-6.77057	-2.50151	0.09423
H	-7.11035	-4.18746	-0.36610
C	-4.22623	-5.21335	-1.55711
H	-5.00682	-5.90243	-1.92342
H	-3.39140	-5.23406	-2.27809
H	-3.85680	-5.60844	-0.59429
C	-3.49387	-2.32018	-0.63618
H	-3.88298	-1.83133	0.27690
H	-2.73995	-3.05324	-0.28659
C	-5.65611	-2.91144	-3.03040
H	-6.49723	-3.58292	-3.27853
H	-6.03866	-1.87833	-3.01250
H	-4.91323	-2.98172	-3.84090
C	2.81600	6.07127	-1.52096
H	1.87900	6.13296	-2.09730
H	2.56636	6.21680	-0.45559
H	3.46606	6.90891	-1.82892
C	5.28447	4.38833	-0.72093
H	5.03989	4.46075	0.35338
H	5.86348	3.46019	-0.87128
H	5.94479	5.23718	-0.97004
C	4.23039	4.26048	-3.61972
H	4.92103	5.08583	-3.86777
H	4.74971	3.31083	-3.82787
H	3.36911	4.32851	-4.30325
C	2.62866	2.94195	-1.11955
H	3.34222	2.14744	-0.81537

H	2.20600	3.34137	-0.17594
C	-1.17296	-5.33399	2.32778
H	-2.08838	-5.88937	2.55653
C	-0.42030	-4.74405	3.35662
H	-0.74491	-4.84256	4.39903
C	0.76624	-4.04355	3.06118
H	1.33955	-3.60767	3.88546
C	-0.71407	-5.22711	1.00026
H	-1.27311	-5.70414	0.18759
C	0.46557	-4.52271	0.71510
H	0.81889	-4.46086	-0.32040
C	3.64016	-5.00161	0.32072
H	3.28278	-5.60962	1.15995
C	4.44576	-5.59064	-0.66668
H	4.71408	-6.65018	-0.58825
C	4.90681	-4.82866	-1.75453
H	5.53426	-5.28872	-2.52526
C	4.55743	-3.47106	-1.83971
H	4.91937	-2.86090	-2.67508
C	3.74405	-2.89196	-0.84995
H	3.48554	-1.82816	-0.88601
C	3.49492	-0.01628	-4.22997
H	4.52540	-0.37942	-4.31702
C	2.51546	-0.45479	-5.13440
H	2.76802	-1.16742	-5.92648
C	1.20616	0.04859	-5.01787
H	0.42622	-0.28109	-5.71206
C	3.16796	0.90577	-3.21748
H	3.95756	1.22987	-2.53321
C	0.88853	0.96709	-4.00544
H	-0.13495	1.34774	-3.92961
C	-1.38105	-3.04993	-2.70415
H	-1.10575	-3.43671	-1.71509
C	-0.90357	-3.70011	-3.85324
H	-0.26218	-4.58386	-3.75434
C	-1.26178	-3.22858	-5.12945
H	-0.89627	-3.73369	-6.02966
C	-2.10594	-2.11300	-5.22963
H	-2.40756	-1.74109	-6.21555
C	-2.58335	-1.46845	-4.07228
H	-3.25437	-0.61131	-4.17721
C	-2.91686	1.15075	-2.29451
H	-1.84970	1.09398	-2.53605
C	-3.62317	2.33438	-2.55287
H	-3.10273	3.18779	-2.99914
C	-4.98749	2.42604	-2.22272
H	-5.54592	3.34630	-2.42564
C	-5.62157	1.32723	-1.62552
H	-6.67984	1.38647	-1.34729
C	-4.90671	0.13955	-1.38180
H	-5.42925	-0.69685	-0.90971
C	-0.44007	3.92043	-1.29297
H	-0.47949	3.43678	-0.31103
C	-1.27030	5.01817	-1.56689
H	-1.95374	5.38338	-0.79271
C	-1.23426	5.63051	-2.83170
H	-1.88397	6.48406	-3.05339
C	-0.35995	5.13141	-3.81084
H	-0.32732	5.59161	-4.80485
C	0.47849	4.03936	-3.52345
H	1.15392	3.66484	-4.29941
C	-2.92427	2.84456	1.52494
H	-2.85074	1.97849	0.85961
C	-3.88279	3.84280	1.27083

H	-4.53571	3.74730	0.39691
C	-3.99539	4.94755	2.12963
H	-4.73704	5.72931	1.93286
C	-3.14659	5.04081	3.24686
H	-3.22353	5.89806	3.92512
C	-2.19739	4.03867	3.49650
H	-1.53743	4.12702	4.36744
C	2.10975	4.05900	2.75587
H	2.44197	4.92865	2.17811
C	0.81387	3.55282	2.56377
H	0.15342	4.03257	1.83487
C	2.97445	3.46588	3.69471
H	3.98100	3.86606	3.85501
C	2.51360	2.37113	4.44528
H	3.16111	1.91300	5.20171
C	1.21098	1.87355	4.25148
H	0.87439	1.03210	4.86528
K	1.92333	0.77952	1.33226
K	0.90854	-1.24202	-1.66992
Li	-0.89842	0.90896	-0.13352
K	-0.91343	-1.66390	1.66117

3Na Disaggregation Species

(κ^2-Me$_6$Tren) Na(μ-OR) Na$_3$(OR)$_3$
SCF (BP86) Energy = -3653.79856849
Enthalpy 0K = -3652.090852
Enthalpy 298K = -3651.971463
Free Energy 298K = -3652.261496
Lowest Frequency = 7.3521 cm^{-1}
Second Frequency = 9.1979 cm^{-1}
SCF (BP86-D3BJ) Energy = -3654.40660630
SCF (C6H6) Energy = -3653.81540830
SCF (BS2) Energy = -5445.54637348

Si 1.57717 5.37229 1.79987
Si 3.49676 -1.95872 2.94338
Si -3.66870 -3.77718 3.07452
Si -6.21940 -2.23356 -3.70498
O -0.27406 1.86372 0.22419
O 2.13575 -1.48547 0.09698
O -1.66621 -0.86093 1.79750
O -3.37177 -0.44474 -1.04053
C 0.05289 3.20549 0.08135
C -1.19603 4.10938 0.36621
C 0.52430 3.47187 -1.37754
C 2.07355 -2.82402 0.46544
C 3.19927 -3.67142 -0.23501
C 0.77195 -3.50701 -0.08768
C -2.05501 -1.09393 3.12115
C -3.44696 -0.46852 3.45601
C -1.01622 -0.42354 4.08622
C -4.34610 -0.30874 -2.02541
C -5.37166 0.74312 -1.51980
C -3.73100 0.15924 -3.37992
C 5.26493 -2.10336 2.22877
H 5.94411 -1.46895 2.82660
H 5.64901 -3.13560 2.25955
H 5.32367 -1.76860 1.18035
C 3.57636 -2.67061 4.71434
H 3.90916 -3.72348 4.70346
H 4.28412 -2.10079 5.34119
H 2.58813 -2.63250 5.20135
C 3.08548 -0.09928 3.03547
H 3.14880 0.36636 2.03714
H 2.07870 0.07995 3.45013
H 3.79856 0.42012 3.70020
C 2.13367 -2.95716 2.02079
H 2.16640 -4.00710 2.37151
H 1.17709 -2.56175 2.41886
C -2.16035 -2.63722 3.44249
H -2.09989 -2.78705 4.54091
H -1.25568 -3.12518 3.02741
C -5.13584 -3.42822 4.23491
H -4.81353 -3.40496 5.28965
H -5.61601 -2.46270 4.00975
H -5.89277 -4.22600 4.13148
C -3.02277 -5.54084 3.43711
H -2.64622 -5.62387 4.47142
H -3.82340 -6.29052 3.31124
H -2.19643 -5.80856 2.75593
C -4.35352 -3.81356 1.28445
H -4.73455 -2.83453 0.94507
H -3.63151 -4.22250 0.55309
H -5.21664 -4.50265 1.26748
C -6.96532 -3.90147 -3.12872
H -6.17302 -4.64033 -2.91486
H -7.56415 -3.77742 -2.20938
H -7.62456 -4.33273 -3.90221
C -5.26675 -2.55430 -5.32668
H -5.94583 -2.93782 -6.10836
H -4.79019 -1.63547 -5.70463
H -4.47446 -3.30790 -5.17308
C -5.00607 -1.71885 -2.29277
H -5.45280 -2.05673 -1.33637
H -4.13969 -2.39264 -2.45770
C -7.67699 -1.05299 -4.05850
H -8.34749 -1.51551 -4.80462
H -8.27071 -0.84383 -3.15400
H -7.33898 -0.08709 -4.46624
C 0.22039 6.01262 2.97866
H -0.71574 6.23586 2.44180
H -0.01206 5.27217 3.76344
H 0.55913 6.93691 3.47962
C 3.19038 5.15633 2.80488
H 3.04108 4.45064 3.64112
H 3.99148 4.75330 2.15886
H 3.53273 6.11576 3.23107
C 1.88769 6.73272 0.49213
H 2.00962 7.70337 1.00530
H 2.80234 6.55339 -0.09732
H 1.04342 6.82666 -0.20984
C 1.15847 3.60396 1.14082
H 2.10861 3.11579 0.84204
H 0.84172 3.07155 2.06004
C -1.45164 -4.76912 -1.33919
H -2.29344 -5.26567 -1.83327
C -0.79943 -5.38353 -0.25327
H -1.14370 -6.35828 0.10981
C 0.30506 -4.76401 0.35479
H 0.81914 -5.27820 1.17369
C -0.99643 -3.51724 -1.79446
H -1.48594 -3.03012 -2.64615
C 0.09095 -2.88821 -1.15675
H 0.45810 -1.90746 -1.48118
C 4.00061 -4.63167 0.41596
H 3.87240 -4.81462 1.48643
C 4.96534 -5.37865 -0.28419
H 5.57091 -6.11644 0.25414
C 5.14552 -5.19582 -1.66315
H 5.88487 -5.78957 -2.21059
C 4.34319 -4.25771 -2.33664
H 4.44561 -4.12425 -3.41970
C 3.38827 -3.51185 -1.62900
H 2.72314 -2.83020 -2.17356
C 2.06734 4.36275 -3.07167
H 2.99921 4.89138 -3.30724
C 1.20809 3.95965 -4.10375
H 1.45948 4.16804 -5.14980
C 0.01347 3.29030 -3.77630
H -0.66856 2.95366 -4.56480
C 1.72710 4.11892 -1.72549
H 2.40094 4.46713 -0.93853
C -0.31086 3.04305 -2.43551
H -1.23561 2.50977 -2.19507
C -2.39416 -0.17229 -3.66962
H -1.81745 -0.66924 -2.88257
C -1.81635 0.14767 -4.90919
H -0.77728 -0.13348 -5.11668

```
C  -2.56247  0.83522 -5.88157
H  -2.11443  1.08963 -6.84849
C  -3.88802  1.20273 -5.59454
H  -4.47704  1.75604 -6.33484
C  -4.46378  0.86413 -4.35849
H  -5.49423  1.16539 -4.14351
C  -4.96728  2.09734 -1.44003
H  -3.98917  2.38575 -1.84364
C  -5.79642  3.08250 -0.88533
H  -5.44411  4.11816 -0.83341
C  -7.06950  2.73889 -0.39584
H  -7.72505  3.50535  0.03153
C  -7.49033  1.40287 -0.46734
H  -8.48286  1.11898 -0.09825
C  -6.64712  0.41853 -1.01703
H  -6.99930 -0.61651 -1.05077
C  -2.03388  3.76782  1.44954
H  -1.76027  2.89454  2.05121
C  -3.16238  4.53166  1.78373
H  -3.78634  4.23900  2.63552
C  -3.48544  5.67209  1.02732
H  -4.36586  6.27309  1.27924
C  -2.66366  6.03157 -0.05287
H  -2.90317  6.91762 -0.65214
C  -1.53273  5.26031 -0.37789
H  -0.90916  5.55541 -1.22761
C  -4.36877 -0.16821  2.43258
H  -4.11332 -0.35728  1.38141
C  -5.63797  0.36247  2.73166
H  -6.32545  0.59536  1.91144
C  -6.01001  0.60267  4.06341
H  -6.99505  1.02161  4.29658
C  -5.10327  0.30271  5.09470
H  -5.37883  0.48490  6.13974
C  -3.83978 -0.22607  4.79159
H  -3.13926 -0.44448  5.60530
C  -0.03598  1.68026  4.91118
H  -0.00025  2.77468  4.87526
C  -0.91060  0.98627  4.06426
H  -1.57248  1.54771  3.39930
C   0.77026  0.97428  5.82227
H   1.44276  1.50979  6.50079
C   0.69286 -0.42487  5.85378
H   1.31011 -0.99175  6.55972
C  -0.18272 -1.11364  4.99088
H  -0.22245 -2.20540  5.05034
Na  0.46826 -0.08446  1.06186
Na  3.83193 -0.74624 -1.12838
Na -2.37089  1.03232  0.33275
Na -2.45813 -2.08305  0.09791
N   6.01369  0.55054 -1.88485
N   5.79450  3.42219  0.70376
N   8.58686 -2.17319 -1.17068
N   3.38886  0.02201 -3.43729
C   6.48344  1.54957 -0.88437
H   7.28668  2.17840 -1.33253
H   6.94423  0.99835 -0.04772
C   7.11852 -0.41643 -2.16017
H   6.87836 -0.92743 -3.10768
C   5.60260  1.23247 -3.15378
H   6.07264  0.70727 -4.00309
H   6.00968  2.26423 -3.18427
C   4.08734  1.32574 -3.39683
H   3.60416  1.93325 -2.61114
H   3.93568  1.88927 -4.34822
C   1.92852  0.24843 -3.51390
H   1.58478  0.85649 -2.66120
H   1.40631 -0.72259 -3.48646
H   1.63450  0.78259 -4.44261
C   3.81416 -0.79240 -4.58863
H   3.64438 -0.27131 -5.55888
H   3.23984 -1.73324 -4.60128
H   4.88288 -1.05176 -4.51701
C   5.36891  2.45778 -0.31385
H   4.88742  3.03076 -1.12748
H   4.57890  1.82411  0.13882
C   6.65908  4.48993  0.20246
H   6.74334  5.27644  0.97219
H   6.20565  4.94405 -0.69524
H   7.69422  4.16581 -0.05805
C   6.30685  2.81857  1.93322
H   5.60199  2.04720  2.28746
H   6.37547  3.59502  2.71446
H   7.31619  2.35264  1.83565
C   7.30216 -1.47315 -1.05613
H   7.28809 -0.98482 -0.06548
H   6.43704 -2.18514 -1.07528
C   8.66500 -3.00010 -2.37943
H   7.86807 -3.78117 -2.42492
H   9.64428 -3.50626 -2.41016
H   8.58991 -2.37512 -3.28436
C   8.83768 -2.99055  0.02054
H   9.83401 -3.45770 -0.05833
H   8.08462 -3.80167  0.16431
H   8.82930 -2.35240  0.92023
H   8.08725  0.11220 -2.30703
```

C^{Na} (κ^2)

SCF (BP86) Energy = -1434.15069446
Enthalpy 0K = -1433.412812
Enthalpy 298K = -1433.366440
Free Energy 298K = -1433.495230
Lowest Frequency = 10.8573 cm^{-1}
Second Frequency = 16.3627 cm^{-1}
SCF (BP86-D3BJ) Energy = -1434.34304647
SCF (C6H6) Energy = -1434.15904490
SCF (BS2) Energy = -1882.21907441

```
Si  3.76081 -1.68066 -0.43999
O   0.86556 -0.18238  0.05178
C   1.84748  0.63425 -0.48400
C   2.59207  1.47345  0.60125
C   1.10158  1.66706 -1.40993
C   4.35129 -1.36234  1.34203
H   4.99233 -0.46719  1.39933
H   3.50065 -1.20346  2.02276
H   4.93375 -2.22728  1.70655
C   5.31305 -2.03826 -1.50746
H   6.01681 -1.18777 -1.48715
H   5.85275 -2.92792 -1.13800
H   5.04650 -2.22486 -2.56287
C   2.67490 -3.24883 -0.49007
H   1.81441 -3.17646  0.19454
H   2.28854 -3.42503 -1.51006
H   3.26722 -4.13634 -0.20361
C   2.89795 -0.19071 -1.29442
H   3.69046  0.45569 -1.72156
```

```
H   2.37979 -0.65082 -2.15983
C  -0.64241  3.33014 -2.95391
H  -1.30334  3.97252 -3.54572
C   0.20602  2.40590 -3.58222
H   0.21474  2.32634 -4.67607
C   1.06625  1.58967 -2.82001
H   1.72951  0.89010 -3.33823
C  -0.60201  3.44536 -1.54939
H  -1.22782  4.18952 -1.04256
C   0.25731  2.63071 -0.79655
H   0.29911  2.74081  0.29342
C   3.65566  2.34771  0.29103
H   3.98727  2.45456 -0.74878
C   4.29041  3.09680  1.29399
H   5.11814  3.76624  1.03243
C   3.86423  2.99457  2.63011
H   4.35989  3.57857  3.41353
C   2.79674  2.14031  2.94773
H   2.45430  2.05575  3.98618
C   2.16832  1.39007  1.93911
H   1.33647  0.71079  2.15197
Na -1.11586  0.11324 -0.80971
N  -3.05773 -1.11476  0.28767
N  -0.35440 -3.31193  1.95713
N  -4.17462  1.87181  2.37145
N  -2.28647 -1.04501 -2.68123
C  -2.43331 -1.97352  1.33490
H  -3.20417 -2.65481  1.76391
H  -2.10983 -1.30591  2.15130
C  -4.01102 -0.16717  0.92749
H  -4.72107  0.17106  0.15332
C  -3.71432 -1.92369 -0.77220
H  -4.61125 -1.38021 -1.11622
H  -4.08453 -2.89135 -0.36627
C  -2.82786 -2.23780 -1.99384
H  -1.96531 -2.85055 -1.68343
H  -3.43046 -2.86630 -2.69390
C  -1.27391 -1.45426 -3.67420
H  -0.46775 -2.01615 -3.17423
H  -0.83085 -0.55641 -4.13488
H  -1.70240 -2.09354 -4.47957
C  -3.34051 -0.26145 -3.34858
H  -3.88324 -0.85249 -4.12217
H  -2.88542  0.61638 -3.83562
H  -4.07755  0.10507 -2.61578
C  -1.19181 -2.78694  0.87681
H  -1.51213 -3.64785  0.25826
H  -0.53081 -2.14543  0.26219
C  -1.00326 -4.29730  2.81453
H  -0.24211 -4.78351  3.45043
H  -1.47698 -5.07995  2.19515
H  -1.78265 -3.87963  3.49763
C   0.35180 -2.27202  2.70800
H   0.77704 -1.54212  1.99381
H   1.17231 -2.73444  3.28494
H  -0.29215 -1.72353  3.43964
C  -3.29532  1.05899  1.52850
H  -2.45473  0.71845  2.15909
H  -2.84294  1.66493  0.69735
C  -5.21132  2.55831  1.59746
H  -4.79430  3.25190  0.82539
H  -5.84447  3.15056  2.27890
H  -5.86493  1.83106  1.08871
C  -3.40075  2.83549  3.15907
H  -4.07754  3.38826  3.83238
H  -2.85482  3.58199  2.53281
H  -2.66012  2.30339  3.77915
H  -4.60873 -0.65774  1.72725
```

TS(C-D)^{Na} (κ^2)

SCF (BP86) Energy = -1434.11698845
Enthalpy 0K = -1433.381701
Enthalpy 298K = -1433.335250
Free Energy 298K = -1433.464785
Lowest Frequency = -380.8572 cm^{-1}
Second Frequency = 10.5526 cm^{-1}
SCF (BP86-D3BJ) Energy = -1434.31132013
SCF (C6H6) Energy = -1434.12703663
SCF (BS2) Energy = -1882.18804657

```
Si  0.98254  1.51239  2.57950
N  -2.47949 -0.71338 -0.91636
N  -2.67488  2.83156 -2.32211
N  -0.29859 -2.69108 -1.85496
N  -5.30710 -0.78456  1.69236
C  -2.80844  0.39775 -1.84700
H  -3.88958  0.41671 -2.10312
H  -2.27119  0.19862 -2.79032
C  -2.72186 -2.02012 -1.57599
H  -2.84025 -2.78181 -0.78478
C  -3.19078 -0.61426  0.38804
H  -2.82796  0.29361  0.89727
H  -2.86993 -1.47004  1.01161
C  -4.73639 -0.62957  0.34754
H  -5.12520  0.28650 -0.16355
H  -5.07221 -1.49385 -0.25482
C  -5.13032  0.42016  2.51127
H  -4.06218  0.65677  2.64182
H  -5.55516  0.24429  3.51364
H  -5.63425  1.31845  2.07664
C  -6.72625 -1.13999  1.61765
H  -7.35643 -0.35490  1.13264
H  -7.11825 -1.30511  2.63569
H  -6.84950 -2.07742  1.04845
C   3.44739  0.71591  2.21332
H   4.07640  1.61042  2.19872
H   3.70195 -0.03820  2.96354
C  -0.94576  1.76872  2.49065
H  -1.26739  2.22645  1.53823
H  -1.28191  2.43240  3.30942
H  -1.48111  0.80816  2.61561
C   1.66443  3.30117  2.53402
H   0.88470  4.03468  2.81073
H   2.01735  3.54749  1.51832
H   2.51982  3.41873  3.21796
C   1.12952  0.71674  4.32337
H   0.97948 -0.37727  4.27951
H   0.34853  1.12179  4.99464
H   2.11772  0.89643  4.77256
Na  0.01775 -0.83661 -0.16535
C  -2.40272  1.78744 -1.32669
H  -2.99461  2.02822 -0.42660
H  -1.33098  1.77651 -1.00285
C  -1.68620  2.84586 -3.40259
H  -1.96167  3.61621 -4.14234
H  -1.66506  1.87762 -3.92931
H  -0.65098  3.06667 -3.04341
```

C -2.75595 4.15454 -1.69452
H -3.54509 4.15569 -0.92404
H -3.02256 4.90621 -2.45701
H -1.80135 4.47682 -1.21462
C -1.59133 -2.44007 -2.52981
H -1.92534 -3.33916 -3.09982
H -1.42696 -1.64396 -3.27794
C   0.79296 -2.71714 -2.85135
H   0.65263 -3.52161 -3.60868
H   1.75255 -2.88110 -2.33570
H   0.84201 -1.74956 -3.37873
C -0.31845 -3.97948 -1.13041
H   0.64882 -4.13103 -0.62430
H -0.50249 -4.83808 -1.81633
H -1.10913 -3.98159 -0.36214
H -3.67279 -2.02473 -2.15418
O   1.12481 0.56757 1.13072
C   2.82766 0.28816 0.98712
C   2.86235 -1.22050 0.78257
C   2.26426 -2.07647 1.74930
C   3.54493 -1.84016 -0.29456
C   2.39347 -3.47064 1.67175
H   1.72232 -1.62268 2.58388
C   3.67146 -3.23798 -0.37158
H   4.01307 -1.21598 -1.06186
C   3.10209 -4.06687 0.61107
H   1.94145 -4.09777 2.44935
H   4.22982 -3.68059 -1.20511
H   3.21384 -5.15498 0.55663
C   3.23564 1.09442 -0.24716
C   4.61319 1.24373 -0.53205
C   2.31523 1.68833 -1.13557
C   5.05131 1.93959 -1.66919
H   5.33725 0.81041 0.16557
C   2.75050 2.39508 -2.27016
H   1.25189 1.63627 -0.88396
C   4.12021 2.51809 -2.54802
H   6.12519 2.03999 -1.86286
H   2.01263 2.86425 -2.93199
H   4.46023 3.07117 -3.43036

D^{Na} (κ^2)
SCF (BP86) Energy = -1434.18221374
Enthalpy 0K = -1433.445911
Enthalpy 298K = -1433.397920
Free Energy 298K = -1433.536226
Lowest Frequency = 6.3008 cm^{-1}
Second Frequency = 9.4925 cm^{-1}
SCF (BP86-D3BJ) Energy =
-1434.36120119
SCF (C6H6) Energy = -1434.19203424
SCF (BS2) Energy = -1882.25812053

Si 1.37128 -3.38372 -0.03672
N   2.56662 1.50414 -0.13195
N   1.18770 0.63251 -3.57598
N   0.14142 2.50689 1.46550
N   6.20368 0.49971 0.61425
C   2.43077 1.57292 -1.61483
H   3.33044 1.16147 -2.10735
H   2.36319 2.63319 -1.92487
C   2.48074 2.81448 0.53744
H   2.90652 2.70619 1.55142
C   3.72643 0.69038 0.30810
H   3.62484 -0.29850 -0.17033
H   3.64011 0.51837 1.39792
C   5.11952 1.30660 0.04016
H   5.26426 1.47877 -1.05638
H   5.16143 2.30177 0.52073
C   6.43120 -0.73914 -0.13801
H   5.52865 -1.37018 -0.14383
H   7.23524 -1.31732 0.34761
H   6.73324 -0.55292 -1.19815
C   7.44357 1.27428 0.69687
H   7.83290 1.59900 -0.29948
H   8.22663 0.66607 1.18125
H   7.28368 2.17692 1.31170
C -3.47819 -2.17304 -0.73744
H -3.88000 -2.35614 -1.73819
H -2.75292 -2.89456 -0.35257
C   3.28749 -3.40660 -0.23367
H   3.59675 -2.85305 -1.13935
H   3.68980 -4.43252 -0.31816
H   3.76982 -2.93178 0.64058
C   0.67405 -4.36275 -1.53532
H   1.08285 -5.38863 -1.58067
H   0.92604 -3.86137 -2.48712
H -0.42633 -4.44312 -1.48420
C   0.99975 -4.43880 1.52721
H   1.46130 -3.98690 2.42421
H   1.39161 -5.46812 1.43486
H -0.08692 -4.51032 1.71252
Na 0.58738 0.13926 0.72333
C   1.21300 0.77208 -2.12055
H   1.24864 -0.24414 -1.67572
H   0.27094 1.26157 -1.74007
C   0.89497 1.89257 -4.25864
H   0.90175 1.73093 -5.34990
H   1.66378 2.64978 -4.03176
H -0.10315 2.31739 -3.98169
C   0.23970 -0.40793 -3.98625
H   0.48147 -1.35415 -3.47651
H   0.31405 -0.56550 -5.07615
H -0.82114 -0.15096 -3.74803
C   1.03852 3.34346 0.63421
H   1.06547 4.39383 1.01030
H   0.60561 3.38236 -0.38241
C -1.26574 2.88644 1.23400
H -1.47198 3.94476 1.51415
H -1.92730 2.23442 1.82873
H -1.51399 2.75775 0.16680
C   0.44911 2.64376 2.90225
H -0.23146 1.99800 3.48128
H   0.33046 3.69220 3.26056
H   1.48294 2.32471 3.11192
H   3.08772 3.59874 0.03039
O   0.77051 -1.86194 0.06863
C -3.85502 -1.08314 -0.02025
C -3.25484 -0.80230 1.31862
C -1.94495 -1.24475 1.63715
C -3.99858 -0.13685 2.32325
C -1.42206 -1.04870 2.92637
H -1.29937 -1.72820 0.88350
C -3.46479 0.06823 3.60546
H -5.01487 0.20327 2.10033
C -2.17441 -0.39131 3.91759
H -0.42288 -1.43972 3.15467
H -4.06839 0.57293 4.36782

H	-1.76562	-0.25179	4.92445
C	-4.89845	-0.16112	-0.56457
C	-6.03640	-0.67576	-1.22788
C	-4.76564	1.24369	-0.46697
C	-6.99395	0.18016	-1.79061
H	-6.17181	-1.76106	-1.28055
C	-5.72176	2.10075	-1.03046
H	-3.89458	1.66193	0.04777
C	-6.84014	1.57288	-1.69649
H	-7.86969	-0.24279	-2.29444
H	-5.59234	3.18569	-0.95104
H	-7.59001	2.24193	-2.13134

Section 3. References

[1] N. Davison, P. G. Waddell, C. Dixon, C. Wills, T. J. Penfold and E. Lu., *Dalton Trans.* **2022**, *51*, 10707-10713.

[2] S. E. Baillie, W. Clegg, P. García-Álvarez, E. Hevia, A. R. Kennedy, J. Klett and L. Russo, *Chem. Commun.* **2011**, *47*, 388-390.

[3] J. R. Lynch, A. R. Kennedy, J. Barker, J. Reid and R. E. Mulvey, *Helv. Chim. Acta* **2022**, *105*, e202200082.

[4] D. E. Anderson, A. Tortajada and E. Hevia, *Angew. Chem. Int. Ed.* **2023**, *62*, e202218498.

[5] N. Davison, C. L. McMullin, L. Zhang, S. X. Hu, P. G. Waddell, C. Wills, C. Dixon, and E. Lu, *J. Am. Chem. Soc.* **2023**, *145*, 6562-6576.

[6] Y. Bai, Z. Lin, Z. Ye, D. Dong, J. Wang, L. Chen, F. Xie, Y. Li, P. H. Dixneuf and M. Zhang, *Org. Lett.* **2022**, *24*, 7988-7992.

[7] L. Feng, Y. Tuo, Z. Wu, W. Zhang, C. Li, B. Yang, L. Liu, J. Gong, G. Jiang, W. Hu and B. Z. Tang, *J. Am. Chem. Soc*. **2024**, *146*, 32582-32594.

[8] N. Alotaibi, R. Babaahmadi, S. Das, E. Richards, T. Wirth, M. Pramanik and R. L. Melen, *Chem. Eur. J.* **2024**, e202404236.

[9] C. Y. Huang and A. G. Doyle, *J. Am. Chem. Soc.* **2015**, *137*, 5638-5641.

[10] J. C. Wu, L. B. Gong, Y. Xia, R. J. Song, Y. X. Xie and J. H. Li, *Angew. Chem. In. Ed.* **2012**, *51*, 9909-9913.

[11] S. Z. Tasker, A. C. Gutierrez and T. F. Jamison, *Angew. Chem. In. Ed.* **2014**, *53*, 1858-1861.

[12] I. MacLean, M. J. García, S. Cabrera, L. Marzo and J. Alemán, *Green Chem.*, **2024**, *26*, 6553-6558.

[13] J. Templ and M. Schnürch, *Angew. Chem. In. Ed.* **2024**, *63*, e202411536.

[14] G. Tan, F. Paulus, Á. Rentería-Gómez, R. F. Lalisse, C. G. Daniliuc, O. Gutierrez and F. Glorius, *J. Am. Chem. Soc.* **2023**, *144*, 21664-21673.

[15] J. Aragón, S. Sun, S. Fernández and J. Lloret-Fillol, *Angew. Chem. In. Ed.* **2024**, *63*, e202405580.

[16] J. Tang, D. Hackenberger and L. J. Goossen, *Angew. Chem. In. Ed.* **2024**, *55*, 11296-11299.

[17] M. Golfmann, L. Glagow, A. Giakoumidakis, C. Golz and J. C. Walker, *Chem. Eur. J.* **2023**, *29*, e202202373.

[18] K. H. Lee, B. Lee, K. R. Lee, M. H. Yi and N. H. Hur, *Chem. Commun.* **2012**, *48*, 4414-4416.

[19] Z. Zhang, D. Li and C. Xi, *Org. Lett.* **2023**, *25*, 698-702.

[20] F. Mäsing, A. Mardyukov, C. Doerenkamp, H. Eckert, U. Malkus, H. Nüsse, J. Klingauf and A. Studer, *Angew. Chem. In. Ed.* **2015**, *54*,12612-12617.

[21] S. Patra, I. Mosiagin, R. Giri, T. Nauser, and D. Katayev, *Angew. Chem. In. Ed.* **2023**, *62*, e202300533.

[22] L. Zhou, R. Huang, S. Lu, B. Liu, M. Gao and B. Xu, *Org. Lett.* **2023**, *25*, 1415-1419.

[23] M. L. Rao and R. J. Dhanorkar, *Eur. J. Org. Chem.* **2014***,* 5214–5228.

[24] D. Cao, S. Xia, L. Li, H. Zeng and C. J. Li, *Org. Lett.*, **2024**, 26(30),6418-6423.

[25] G. Zhang, Y. Wang, X. Wen, C. Ding and Y. Li, *Chem. Commun.*, **2012**, *48*, 2979-2981.

[26] A. Modak, A. Deb, T. Patra, S. Rana, S. Maity and D. Maiti, *Chem. Commun.*, **2012**, *48*, 4253-4255.

[27] Gaussian 16 Rev. C.01, Frisch M. J., Trucks G. W., Schlegel H. B., Scuseria G. E., Robb M. A., Cheeseman J. R., Scalmani G., Barone V., Petersson G. A., Nakatsuji H., Li X., Caricato M., Marenich A. V., Bloino J., Janesko B. G., Gomperts R., Mennucci B., Hratchian H. P., Ortiz J. V., Izmaylov A. F., Sonnenberg J. L., Williams, Ding F., Lipparini F., Egidi F., Goings J., Peng B., Petrone A., Henderson T., Ranasinghe D., Zakrzewski V. G., Gao J., Rega N., Zheng G., Liang W., Hada M., Ehara M., Toyota K., Fukuda R., Hasegawa

J., Ishida M., Nakajima T., Honda Y., Kitao O., Nakai H., Vreven T., Throssell K., J. A. Montgomery Jr., Peralta J. E., Ogliaro F., Bearpark M. J., Heyd J. J., Brothers E. N., Kudin K. N., Staroverov V. N., Keith T. A., Kobayashi R., Normand J., Raghavachari K., Rendell A. P., Burant J. C., Iyengar S. S., Tomasi J., Cossi M., Millam J. M., Klene M., Adamo C., Cammi R., Ochterski J. W., Martin R. L., Morokuma K., Farkas O., Foresman J. B. and Fox D. J., Wallingford, CT (2016).

[28] Andrae D., Häußermann U., Dolg M., Stoll H. and Preuß H., Energy-adjusted ab initio pseudopotentials for the second and third row transition elements. *Theor. Chim. Acta*, **1990**, 77, 123-141.

[29] a) Hariharan P. C. and Pople J. A., The influence of polarization functions on molecular orbital hydrogenation energies. *Theor. Chim. Acta*, **1973**, 28, 213-222. b) Hehre W. J., Ditchfield R. and Pople J. A., Self—consistent molecular orbital methods. XII. Further extensions of Gaussian—type basis sets for use in molecular orbital studies of organic molecules. *J. Chem. Phys.*, **1972**, 56, 2257-2261.

[30] a) Becke A. D., Density-functional exchange-energy approximation with correct asymptotic behavior. *Phys. Rev. A*, **1988**, 38, 3098-3100. b) Perdew J. P., Density-functional approximation for the correlation energy of the inhomogeneous electron gas. *Phys. Rev. B*, **1986**, 33, 8822-8824.

[31] Tomasi J., Mennucci B. and Cammi R., Quantum Mechanical Continuum Solvation Models. *Chem. Rev.*, **2005**, 105, 2999-3094.

[32] Grimme S., Ehrlich S. and Goerigk L., Effect of the damping function in dispersion corrected density functional theory. *J. Comp. Chem.*, **2011**, 32, 1456-1465.
